# Developmental responses of bread wheat to changes in ambient temperature following deletion of a locus that includes *FLOWERING LOCUS T1*

**DOI:** 10.1111/pce.13130

**Published:** 2018-02-07

**Authors:** Laura E. Dixon, Alba Farré, E. Jean Finnegan, Simon Orford, Simon Griffiths, Scott A. Boden

**Affiliations:** ^1^ John Innes Centre Norwich NR4 7UH UK; ^2^ CSIRO, Agriculture and Food Canberra Australia

**Keywords:** *FLOWERING LOCUS T*, germination, inflorescence, temperature, wheat

## Abstract

FLOWERING LOCUS T (FT) is a central integrator of environmental signals that regulates the timing of vegetative to reproductive transition in flowering plants. In model plants, these environmental signals have been shown to include photoperiod, vernalization, and ambient temperature pathways, and in crop species, the integration of the ambient temperature pathway remains less well understood. In hexaploid wheat, at least 5 FT‐like genes have been identified, each with a copy on the A, B, and D genomes. Here, we report the characterization of FT‐B1 through analysis of FT‐B1 null and overexpression genotypes under different ambient temperature conditions. This analysis has identified that the FT‐B1 alleles perform differently under diverse environmental conditions; most notably, the FT‐B1 null produces an increase in spikelet and tiller number when grown at lower temperature conditions. Additionally, absence of FT‐B1 facilitates more rapid germination under both light and dark conditions. These results provide an opportunity to understand the FT‐dependent pathways that underpin key responses of wheat development to changes in ambient temperature. This is particularly important for wheat, for which development and grain productivity are sensitive to changes in temperature.

## Abstract 2

The physiology and development of wheat respond dramatically to increases in ambient growth temperature. To combat the adverse effects of this response, we need to understand its genetic and molecular basis. Here, we present the characterization of the FLOWERING LOCUS T (FT)‐B1 mediated temperature responses to flowering, plant architecture, germination, and gene expression. We show that FT‐B1 alleles respond differently under diverse temperatures and that the absence of FT‐B1 enhances germination and increases spikelet production at lower temperatures.

## 1 INTRODUCTION

Flowering and the subsequent production of gametes enable the introduction and maintenance of genetic diversity to a plant species. The timing of flowering has evolved to best suit individual plant species, with respect to coincidence with pollinators and the local environment. Utilizing this naturally highly regulated response has been essential in enabling the production of high yielding crops and has been crucial for the adaptation of crops to geographically diverse regions of the world. Therefore, understanding the mechanisms by which flowering time is controlled is important for our sustained agricultural production. Research conducted in numerous plant species has identified photoperiod and temperature as key environmental triggers, whose signals converge on a few genes that control the transition from vegetative to reproductive development (Jung & Müller, 2009). One of the central and final genetic regulators in this process is *FLOWERING LOCUS T* (*FT;* Pin & Nilsson, 2012). The *FT* gene encodes a protein that has sequence homology to phosphatidylethanolamine binding proteins or RAF kinase inhibitor proteins and has been shown to bind proteins and lipids (Nakamura et al., 2014) but has not yet been observed to directly bind DNA. Genes with high sequence similarity to *FT* from Arabidopsis have been identified across the angiosperm species, and these form relatively large families of *FT*‐like genes, many of which have yet to be characterized (Faure, Higgins, Turner, & Laurie, 2007). The conservation of the *FT*‐like genes is also observed through a central role in flowering time regulation and its action in forming at the least part of florigen, the mobile flowering signal (Putterill & Varkonyi‐Gasic, 2016). In support of this, it has been demonstrated in Arabidopsis and rice that *FT* is expressed in the leaf phloem vasculature and that *FT* protein is then transported to the shoot apical meristem where it exerts its effect as the florigen signal, triggering the change from vegetative to reproductive development (Jaeger & Wigge, 2007; Mathieu, Warthmann, Kuttner, & Schmid, 2007; Tamaki, Matsuo, Wong, Yokoi, & Shimamoto, 2007).

In addition to the conserved roles of *FT*, it is becoming clear that function of *FT* shows distinct roles in different species, as well as context dependent functional diversity. *FT*‐like genes have been identified to act as both floral activators (Kardailsky et al., 1999; Kojima et al., 2002) and repressors (Pin et al., 2010) and as regulators of seed germination (Chen et al., 2014). *FT*‐like genes also play important roles in tuber formation (Navarro et al., 2011), the breaking of bud dormancy (Cooke, Eriksson, & Junttila, 2012; and references within), the regulation of branching (Tsuji et al., 2015), stomatal aperture (Kinoshita et al., 2011), ambient temperature signalling (Balasubramanian, Sureshkumar, Lempe, & Weigel, 2006; Blázquez, Ahn, & Weigel, 2003; Capovilla, Pajoro, Immink, & Schmid, 2015; Hsu et al., 2011), and a role in gibberellin signalling (Hisamatsu & King, 2008; Pearce, Vanzetti, & Dubcovsky, 2013; and references within).

In the monocot crop plants, such as wheat and barley, multiple *FT*‐like genes have been identified and shown to have distinct expression patterns and specific interactions with FD‐like and 14‐3‐3 proteins (Li, Lin, & Dubcovsky, 2015; Taoka et al., 2011). This enables the formation of protein complexes containing *FT* which bind specific DNA sequences and mediate regulation of gene expression in an *FT*‐dependent manner (Faure et al., 2007; Kikuchi, Kawahigashi, Ando, Tonooka, & Handa, 2009; Li et al., 2015; Li & Dubcovsky, 2008). Although this regulation is very similar to that observed in Arabidopsis and rice, other aspects of *FT* regulation differ between the monocots and dicots. In particular, in barley, the expression of *HvFT1*‐like is not directly regulated by high temperature (Hemming, Walford, Fieg, Dennis, & Trevaskis, 2012).

In hexaploid wheat, at least five *FT*‐like genes, with a copy on each the A, B, and D genomes within wheat, have been identified (Lv et al., 2014). However, the distinct roles of the *FT*s in flowering regulation remain largely unknown. *FT‐B1* on Chromosome 7B is the most characterized wheat *FT* gene, mainly because it was identified to have a central role in the vernalization pathway (*FT‐B1* is denoted *VRN3* in the vernalization pathway; Yan et al., 2006). The notion of *FT‐B1* as the central *FT1* was subsequently supported through homologue specific gene expression analysis which identified that *FT‐B1* was expressed at a higher level than either *FT‐A1* or *FT‐D1* in the Paragon cultivar (Shaw, Turner, & Laurie, 2012). Transgenic overexpression of *FT1* bypassed all signals of flowering repression to rapidly promote flowering, with the transformed wheat plants flowering whilst still in the callus stage (Lv et al., 2014). An allele that causes higher expression of *FT‐B1*, first identified in the Hope cultivar, also led to an early flowering phenotype under long days (LD) and bypassing the vernalization requirement (Yan et al., 2006). Notably, this earlier flowering did not lead to any significant decrease in yield (Nitcher, Pearce, Tranquilli, Zhang, & Dubcovsky, 2014). Development of *FT1* RNAi lines in Brachypodium further supported the central role of *FT1* in flowering regulation in grasses, as *FT1* RNAi lines showed dramatic flowering delays. Unfortunately, wheat *FT1* RNAi lines were infertile and so prevented confirmation of this result (Lv et al., 2014). Subsequently, an *FT‐B1* null has been identified and confirmed to be late flowering, this genotype also showed an altered spikelet architecture with the formation of paired spikelets (Boden et al., 2015).

Here, we report the identification of a late flowering hexaploid wheat line which contained a deletion across the *FT‐B1* region. In addition, we have generated a *FT‐B1* overexpressor, using the Hope allele, in a highly similar genetic background (Paragon cultivar). This provided the opportunity to further understand the role of *FT‐B1* in flowering regulation and to identify whether *FT‐B1* in wheat fulfilled similar roles, specifically relating to temperature responses and development, to those identified in other plant species. Understanding this will provide valuable information for the application of *FT‐B1* alleles into an agricultural environment and provide important information for the identification of other genes involved in temperature dependent regulation of development and flowering.

## 2 MATERIALS AND METHODS

### 2.1 Plant material

*FT‐B1* null, hereafter referred to as *ft‐b1*, was identified from a cross between the elite UK spring hexaploid wheat, Paragon (Triticum aestivum L.; *Vrn‐A1a* and *Vrn‐B1c*), and a hexaploid landrace wheat from the Watkins collection, denoted W352 (Wingen et al., 2014). An initial cross was conducted between these parents, and the resulting plant was allowed to self‐fertilize. From these seeds, a population of 94 individuals was developed through single seed descent to, at least, the F_5_ generation (Wingen et al., 2017). All experiments reported in this paper used F_5_ or subsequent self‐fertilized generations. The *ft‐b1* line was identified during a detailed phenotyping experiment at the F_5_ stage. The *ft‐b1* line was single nucleotide polymorphism (SNP)genotyped using the iSelect 90K array (Wang et al., 2014) and identified to contain a near equal genetic contribution from both parents.

To generate the *ft‐b1* near isogenic line (NIL), hereafter referred to as the *ft‐b1* NIL, a backcross was made using F_6_ *ft‐b1* to the recurrent parent, Paragon, and the *ft‐b1* allele was selected using Taqman copy number assay (Díaz, Zikhali, Turner, Isaac, & Laurie, 2012) by iDna (iDna Genetics Ltd.) for three successive backcrosses to the recurrent parent to generate BC_4_ NILs, with BC_4_F_2_ lines being used in the reported experiments. The Taqman assay was conducted on genomic DNA extracted from leaf tissue samples and could clearly distinguish *ft‐b1*, heterozygotes carrying the *ft‐b1*, and homozygotes for *FT‐B1*.

*FT‐B1* overexpressor NIL, hereafter referred to as *FT‐B1* OX, BC_3_F_3_ was generated by backcrossing the cultivar Hope (T. aestivum), carrying the dominant allele associated with the insertion of a transposable element in the *FT‐B1* promoter, to the recurrent parent Paragon to produce BC_1_ plants. BC_1_ plants were backcrossed into Paragon two more times to produce BC_3_ heterozygous plants which were self‐pollinated, and homozygous plants carrying the insertion were selected. These plants were grown and self‐pollinated to obtain the BC_3_F_3_ lines. The lines were selected at each stage using two sequence tagged site markers from Yan et al. (2006) for marker assisted backcrossing.

### 2.2 Plant growth conditions

**Figure 1:**
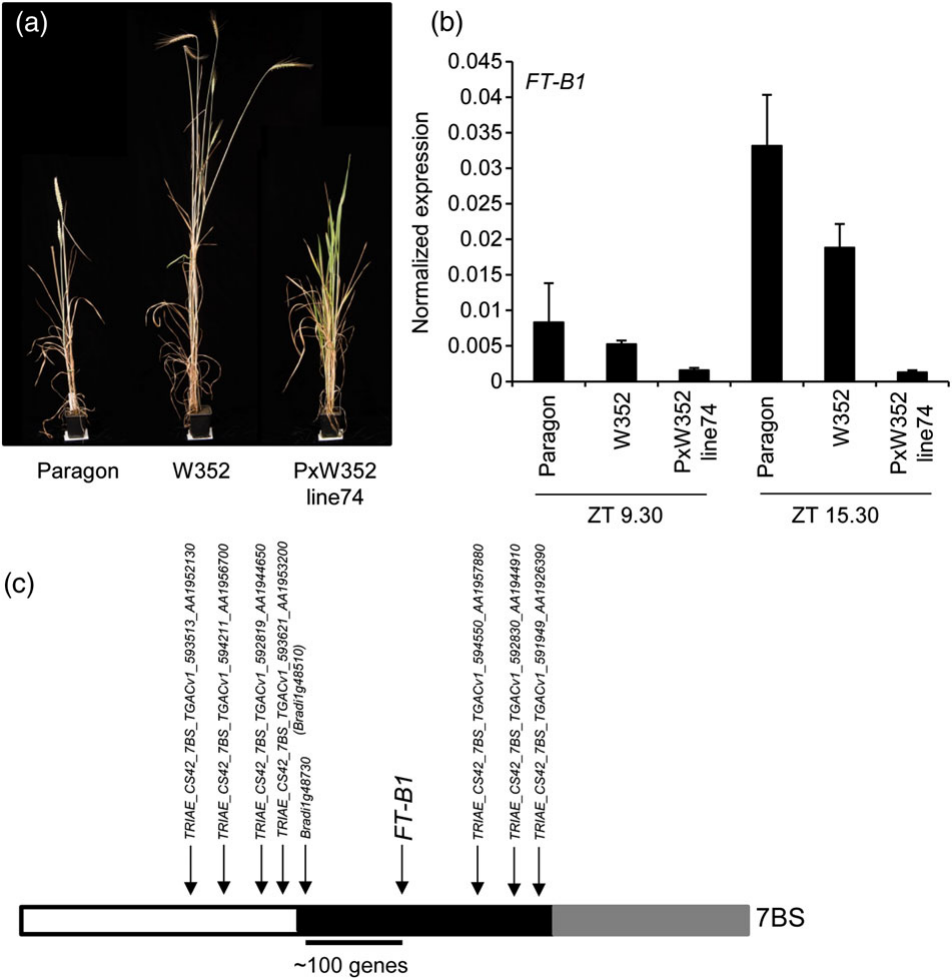
A late flowering hexaploid wheat line was identified to carry a genomic deletion which included *FLOWERING LOCUS T* (*FT*)*‐B1*. (a) Example images showing that flowering was severely delayed in the line carrying the *FT‐B1* deletion, PxW352 Line74, compared to parental genotypes, Paragon, and W352. (b) Gene expression of *FT‐B1* normalized to glyceraldehyde 3‐phosphate dehydrogenase expression at Zeitgeber time (ZT) 9.30 and ZT 15.30 showed that PxW352 Line74 did not express *FT‐B1*, whereas both parental lines did. Error is shown as standard error of the mean (SEM) of three biological replicates. (c) Schematic showing the chromosomal region of 7BS containing the deletion. White indicates chromosome present, black deleted, and grey unknown. Markers have been developed for the genes marked, with gene names corresponding to Ensembl plant annotation [Colour figure can be viewed at http://wileyonlinelibrary.com]

For the gene expression experiments (Figures 1, 5, S1 and S2) and the representative image in Figure 1, plants were grown in 7 × 7 cm plastic pots in cereal mix soil in Gallenkamp controlled‐environment chambers under either high temperature (HT) 24 °C during the light period and 19 °C during the dark period or low temperature (LT) 18 °C during the light period and 13 °C during the dark period. Both rooms were LD photoperiod (16 hr light/8 hr dark) and humidity set to 70%. Two independent flowering time experiments were conducted, with flowering‐time recorded when plants reached Zadok scale 59.

For the phenotyping (Figure 3, including the representative images), *ft‐b1 NIL*, *FT‐B1* OX NIL, and background genotype Paragon were germinated in Petri dishes before being transferred to peat and sand mix in 5 × 5 cm pots and subsequently to cereal mix 11 × 11 cm pots. Data are the average of five plants from each genotype grown in controlled‐environment chambers with the same LD photoperiod and humidity as above and temperature regime of either HT 24 °C during the light period and 19 °C during the dark period or LT 20 °C during the light period and 15 °C during the dark period. Flowering time was recorded as described above.

NILs were developed under LD glasshouse conditions.

### 2.3 Molecular markers and sequence information

Initial polymerase chain reaction (PCR) analysis suggested that *ft‐b1* was a gene deletion. To assess how far this deletion extended, PCR primers were designed based on synteny alignment of 7BS with Brachypodium and rice genomes. Oligonucleotide sequences and annealing temperatures are provided in Table S1. Nucleotide sequences of amplicons were confirmed by Sanger sequencing. Using the markers described, the deletion region of Chromosome 7BS was delimited (details provided in Table S2).

Additional sequence detail on the *FT* homologues and paralogs was obtained using exome capture data (Bian, Tyrrell, & Davey, 2017, https://grassroots.tools) provided in reference list (Figure S4).

### 2.4 Germination assay

Paragon, *ft‐b1* NIL, and *FT‐B1* OX were grown under natural photoperiods in glasshouse conditions (Paragon and *FT‐B1* OX) or controlled photoperiods in growth cabinets (Paragon and *ft‐b1*), and inflorescences harvested at maturity. Threshed seeds were placed at 37 °C for at least 4 weeks to ensure that they were fully after‐ripened. The germination assays were conducted in Snijder Micro Clima Series, Economic Lux Chamber at either a constant 20 °C HT or a constant 10 °C LT with either constant light or constant dark (except for when phenotype scores were taken). The assay was conducted as per Barrero et al. (2015) with up to 18 seeds per genotype per Petri dish. Three separate Petri dishes were phenotyped, with one of these being conducted in an independent experiment.

### 2.5 Apex characterization (short day [SD] and LD)

Paragon, W352, *ft‐b1*, and *FT‐B1* OX were grown under either SD (8 hr light/16 hr dark) or LD photoperiods at a constant 20 °C and humidity set to 70% in Gallenkamp‐controlled environment chambers. Two plants were dissected per genotype for the most time‐points every 3 days once the most developmentally advanced genotype had started floral apex development. A second independent experiment was performed to confirm the result (data not included). The sampling frequency was much less for the SD cabinet due to the slower developmental rate. Apex length was measured in ImageJ from the base of the spikelet forming region of the apex to its tip, and error is shown as standard deviation between two samples.

### 2.6 Quantitative real‐time PCR (qRT‐PCR)

For the identification of *ft‐b1* (Figure S2), leaf tissue samples were taken from Paragon, W352, and PxW352 line74, and RNA was extracted using Tri Reagent (Sigma‐Aldrich) and DNase I treated (NEB) before cDNA was synthesized using Superscript II reverse transcriptase (Invitrogen) and random and Oligo dT primers. For the expression analysis of *FT‐B1* NILs (Figure 5), the fifth leaf was harvested at Zeitgeber time (ZT) 13 hr for Paragon, *ft‐b1* NIL, and *FT‐B1* OX NIL. RNA was extracted using the Spectrum Plant RNA extraction kit (Sigma‐Aldrich) before being treated with DNase I, and cDNA synthesized as described above and diluted (1:10) for use in qRT‐PCR. For the expression analysis of *FT‐B1* NILs (Figure S3), Paragon, *ft‐b1*, and *FT‐B1* OX were germinated in 5 × 5 cm pots containing peat and sand mix under SD conditions. Leaf samples were collected from six plants at the third leaf stage (SD sample), and the remaining plants were transferred to LD conditions for 7 days when the third leaf was then collected (LD sample). This stage was used because it is when the meristem is at the transition apex stage and is therefore competent to receive the flowering signal. RNA extraction and cDNA synthesis were performed as described above for NILs. For the expression analysis from seeds (Figure 4), RNA was extracted from imbibed seeds. Seeds were placed on Whatman filter paper with 5 ml distilled water in 9 cm diameter Petri dishes and incubated at 20 °C overnight (for *ft‐b1* and some Paragon) and for a further 24 hr (remaining Paragon and *FT‐B1* OX) until the seeds had broken dormancy and the definition of the embryo could be identified. The radicle and the plumule of the embryo were dissected and pooled (with three individuals per replicate) and flash frozen in liquid nitrogen. RNA extraction and cDNA synthesis were performed as described above. Oligonucleotide sequences used for qRT‐PCR are given in Table S3 with all data being expressed as normalized product according to 2^^‐ΔCT^. The identity of the normalization gene for each experiment is provided in the respective figure legends.

## 3 RESULTS

3.1

#### 3.1.1. A late flowering line showing altered environmental responses carries a deletion spanning the *FT‐B1* region in wheat

A parental cross between a landrace wheat (W352) and an elite spring wheat cultivar, Paragon, was used to develop a population of 94 genetically unique individuals (see Section 2 for details). From this population, one individual (Line 74) showed a particularly late flowering phenotype with the inflorescence emerging after the other lines had reached maturity and senesced (Figure 1a). Interestingly, the degree of flowering delay depended on the temperature of the growth conditions. Under lower ambient temperatures (18 °C light/13 °C dark), LD conditions flowering of Line 74 was delayed by an average of 30 days, relative to both Paragon and W352. However, under higher ambient temperature conditions (24 °C light/19 °C dark), the delay in flowering of Line 74 was greatly reduced and was observed to be in the range of 10–15 days later than either of the two parents (Figure S1).

As the flowering delay was not observed in either of the parents or the rest of the population, it indicated that the phenotype arose from an independent mutation that occurred spontaneously during the crossing process. Although mutations in multiple genes are known to influence flowering time in plants, relatively few have been identified that delay flowering. We therefore assessed expression of candidate genes in which mutations had previously been reported to show significant flowering delays (Figures 1b and S2). Gene expression was measured at ZT 9.30 hr and ZT 15.30 hr under LD conditions to maximize the likelihood of capturing the genes being expressed, as many of the flowering genes show peaks in transcript levels during hours between the end of the SD and LD photoperiods. Using this approach, we established that *FT‐B1* was not expressed in the late flowering line at either of the time‐points tested (Figure 1b) but was expressed in the two parental lines. However, this absence of expression could have been anticipated due to the late flowering nature of the line, as *FT‐B1* expression increases with transition to flowering. Therefore, to investigate further if this was an output of a signalling cascade or due to a non‐functional *FT‐B1*, we tested if the late flowering line carried a copy of *FT‐B1* through Taqman copy number assay. This assay confirmed both parent lines to contain one haploid copy of *FT‐B1*, and the progeny PxW352 Line 74 was null for *FT‐B1*. An absence of *FT‐B1* would strongly suggest the cause of the observed late flowering phenotype, but this does not exclude the possibility that other important genes in the flowering response may have also been lost in Line 74. To assess the extent of the deletion, gene and genome specific markers were developed. Using synteny with Brachypodium and rice, initial gene order was formed either side of *FT‐B1*, which was subsequently confirmed through the aligned wheat sequence. Using the order supported by synteny, we identified that Line 74 carries a deletion which spans the *FT‐B1* region, with one break point between *Bradi1g48510* (present) and *Bradi1g48730* (absent) and the other break point extending to the distal end of the chromosome (Figure 1c). Using recent advancements in wheat genome assembly, it is evident that the deletion contains more genes than identified through synteny with Brachypodium and rice, and the deletion is estimated to cover a region containing 408 predicted gene models (listed in Table S2), and so a possible contribution of other genes to the flowering‐time phenotype cannot be discounted.

#### 3.1.2. Lines carrying an *FT‐B1* deletion have altered developmental rate and plant architecture

**Figure 2:**
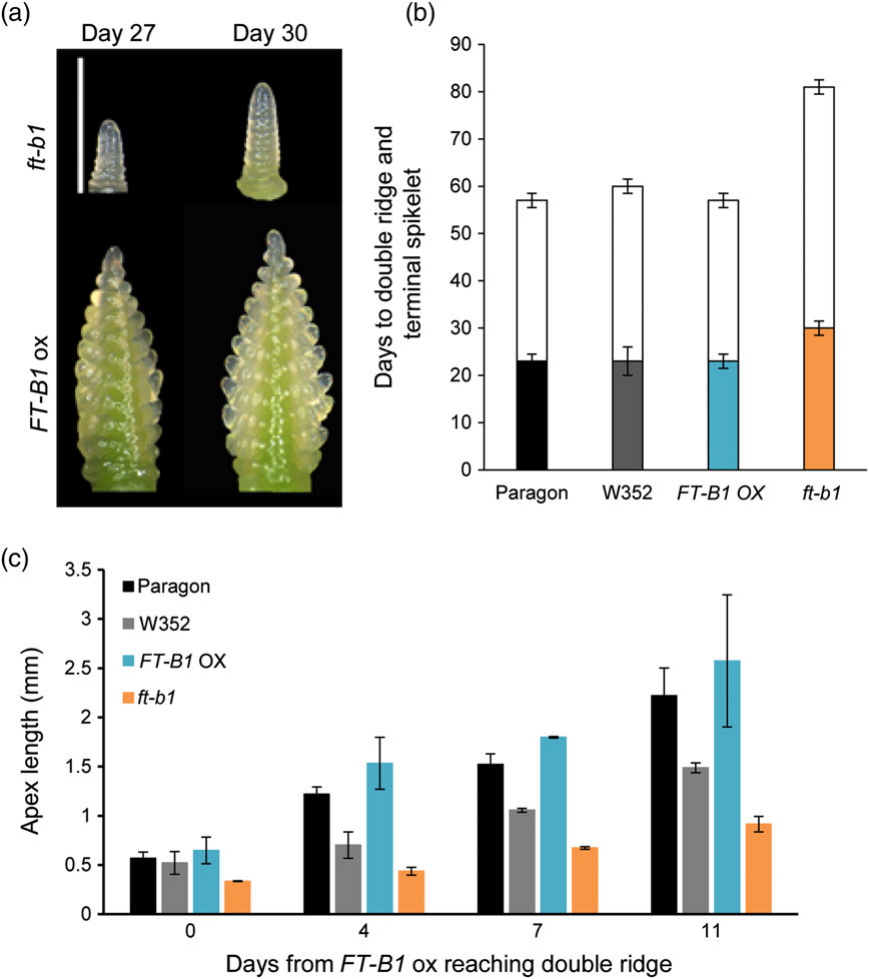
*FLOWERING LOCUS T* (*FT*)*‐B1* affects the timing and rate of vegetative to inflorescence meristem transition. (a) Example images from apex dissection of *FT‐B1* overexpressor and *ft‐b1* (PxW352 Line 74) grown under long‐day (LD) conditions. Scale bar showing 1 mM. (b) Days taken to reach the double ridge (coloured bars) and terminal spikelet (white bars) stage under LD conditions, with error bars as the interval between sampling. (c) Apex length from plants grown under LD conditions, for each genotype for the first four time‐points, the stage at which Paragon and *FT‐B1* overexpressor reached terminal spikelet [Colour figure can be viewed at http://wileyonlinelibrary.com]

The flowering delay observed in plants carrying the *ft‐b1* genotype demonstrates that flowering was affected but provides no indication of the developmental stage that this delay is established. In many plant species, *FT* forms part of the mobile florigen signal that travels from the leaf to the shoot apical meristem to signal the vegetative to reproductive transition. We hypothesized that this conserved function for FT in wheat would result in altered rate of apex development from vegetative to reproductive stages in genotypes with different *FT‐B1* levels. To assess this hypothesis, we conducted an apex dissection time‐course under LD conditions. This time‐course comprised of *ft‐b1*, both parent cultivars, Paragon and W352, and a high‐expression allele of *FT‐B1* in Paragon (described in Section 2 and referred to as *FT‐B1* OX). The developing wheat apices were dissected from all genotypes at 2–3‐day intervals once the first genotype (*FT‐B1* OX) had reached the first visible stage of transitioning to reproductive development, the double ridge stage. This identified that the *ft‐b1* genotype reached double ridge stage ~7 days after the *FT‐B1* OX, showing that inflorescence development was already delayed at this stage (Figure 2a,b, filled bars). The *FT‐B1* OX had reached the terminal spikelet stage (Figure 2b, white bars) at this time‐point. The parent genotypes reached terminal spikelet stage at a similar point to *FT‐B1* OX (Figure 2). The rate of delay in apex development can be quantified through measurement of apex length (Figure 2c) and then used to calculate the rate of growth. The rate of inflorescence growth for the *FT‐B1 OX* line from Days 0 to 11 is on average 0.18 mm day^−1^, whereas *ft‐b1* grew at 0.05 mm day^−1^ over the same period. Although this analysis assumes a linear growth rate which may be misleading, it does allow a quantification of delay in apex development in *ft‐b1* which can be used as an indication to the importance of *FT‐B1* in this process. Under SD conditions, a similar developmental pattern during apex development was observed (data not included), but plants did not complete flowering. The contribution of *FT‐B1* to apex development under SD photoperiods is supported by detection of *FT‐B1* expression under 8 hr photoperiods (Figure S3).

**Figure 3:**
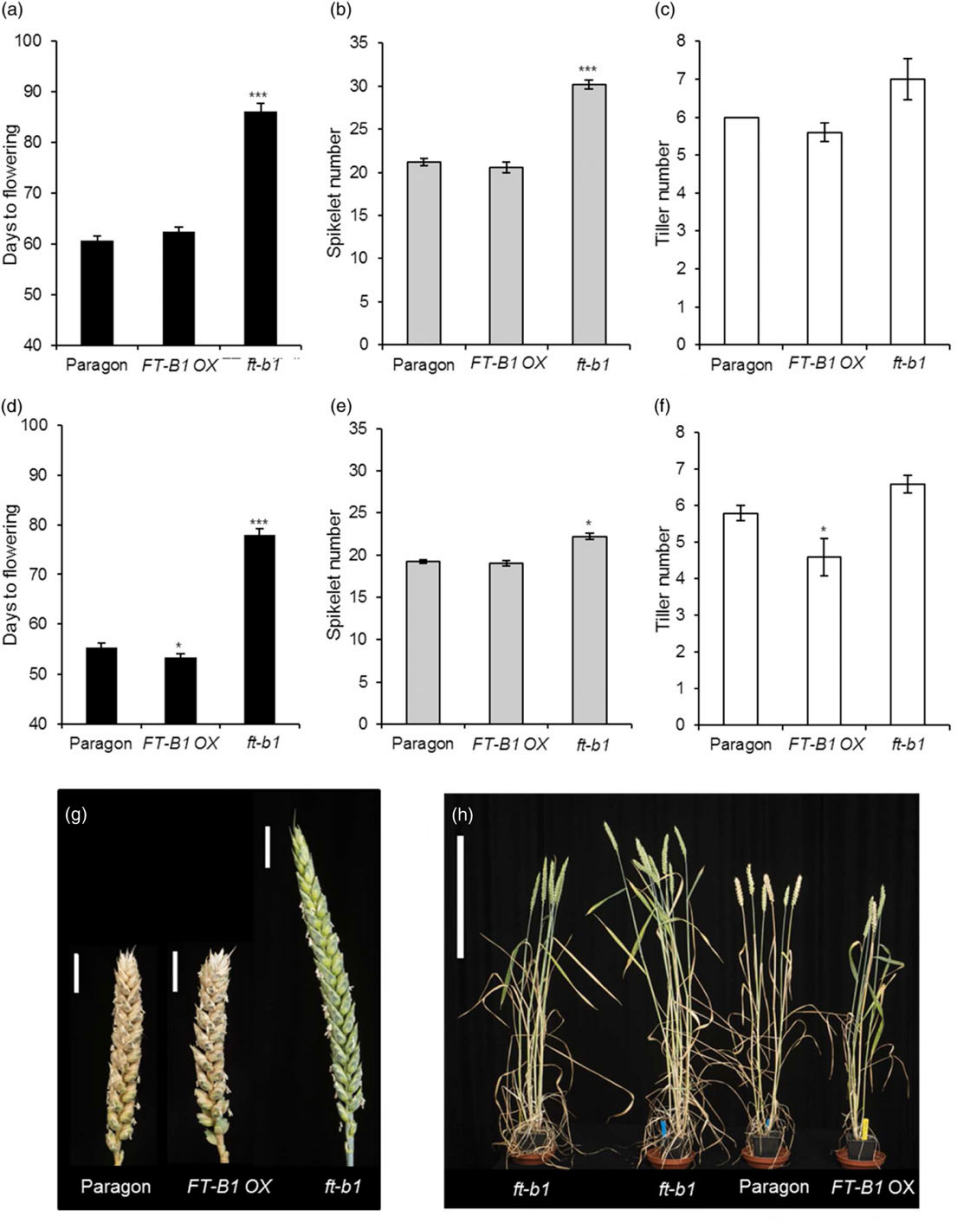
*FLOWERING LOCUS T* (*FT*)*‐B1* near isogenic lines identify distinct regulation via *FT‐B1* on flowering time, tiller, and spikelet numbers under different temperatures. Plants were grown at either a lower temperature of 20 °C light: 15 °C night, long‐day (LD) photoperiod and (a) flowering time, (b) spikelet number, (c) tiller number, measured or under warmer conditions of 24 °C light: 19 °C dark, LD photoperiod and (d) flowering time, (e) spikelet number, (f) tiller number, measured. Values are means with *N* = 4 or 5 and standard error of the mean error bars, with a similar biologically independent experiment repeated. (g) Example images of spikes from lower temperature conditions to highlight the increased branching under these conditions, scale bar showing 2 cm. (h) Example image of whole plants with two replicates of *ft‐b1* NIL shown to demonstrate range of tiller numbers displayed by this genotype; scale bar, 40 cm. Significance determined by Student's *t*‐test between Paragon and either *FT‐1B* overexpressor (OX) or *ft‐b1* NIL (* *p* < .05, *** *p* < .001) [Colour figure can be viewed at http://wileyonlinelibrary.com]

To further understand *ft‐b1* and its role on hexaploid wheat development and flowering regulation, NILs were developed. Using Paragon as the recurrent parent, we generated a *ft‐b1* NIL and a *FT‐B1* OX NIL (using the Hope allele) to understand the role of the *FT‐B1* region in influencing certain agronomic traits; flowering time, plant architecture, and spikelet number. Paragon, *ft‐b1*, and *FT‐B1* OX NILs were grown under the high and low ambient temperature conditions of 24 °C day temperature/19 °C night temperature and 20 °C/15 °C, respectively. *FT‐B1* OX and Paragon flowered at similar times under both temperature conditions. Both lines showed earlier flowering under the high‐temperature condition, which is consistent with previous results showing that spring wheat flowers earlier under warmer conditions (Greenup, Peacock, Dennis, & Trevaskis, 2009). The *ft‐b1* NIL was significantly delayed in flowering time compared to Paragon and *FT‐B1* OX (Figure 3a), but the degree of this delay was similar under both temperature conditions, 23.6 days under higher temperatures (Figure 3a) and 24.6 days under lower temperatures (Figure 3d). As previous reports had shown that *FT* has additional roles in regulating plant architecture, we also assessed spikelet and mature tiller number (Figure 3b,c,e,f; Boden et al., 2015; Tsuji et al., 2015). We observed that *FT‐B1* does influence plant architecture in wheat and that this response is under environmental control (Figure 3b,c,e,f). At the lower temperature, there was no significant difference between Paragon and the *FT‐B1* OX NIL for spikelet number, on average, Paragon formed 21.2 spikelets, and *FT‐B1* OX formed 20.6 spikelets. In *ft‐b1* NIL*,* the spikelet number significantly increased to 30.2/inflorescence, with on average one more tiller (Figure 3g,h). A similar pattern was observed under the higher temperatures, but the response was not as substantial, with an increase in spikelet number, 22.2 *ft‐b1* NIL versus 19.2 in Paragon and 19 in *FT‐B1* OX NIL. However, under the warmer conditions, the role of *FT‐B1* on regulating tiller branching is more distinguishable between the NILs, with the *FT‐B1* OX showing a slight decrease in tiller number (4.6 tillers/plant), and *ft‐b1* NIL showing a slight increase (6.6 tillers/plant), relative to Paragon (5.8 tillers/plant Figure 3c,f).

#### 3.1.3. *FT‐B1* influences wheat seed germination

**Figure 4:**
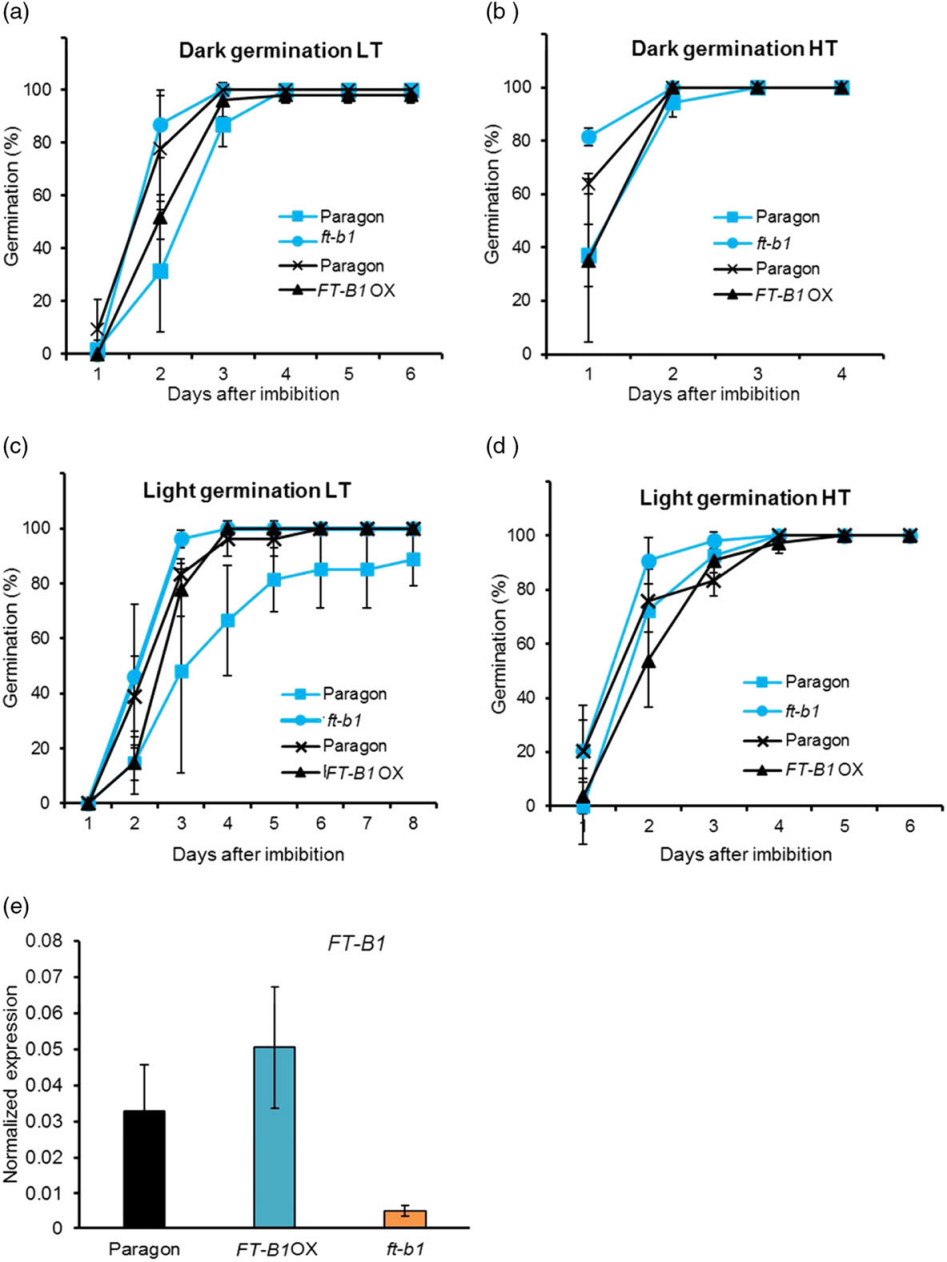
*FLOWERING LOCUS T* (*FT*)*‐B1* is involved in regulating wheat germination. Germination was assessed under continuous darkness at (a) 10 °C low temperature (LT) or (b) 20 °C high temperature (HT) and under continuous light at (c) 10 °C (LT) or (d) 20 °C (HT). Germination is a mean value from three combined independent experiments, each with *N* = 18 and standard error of the mean error bars. For each experiment, the near isogenic lines (NILs) are compared to Paragon which was grown and ripened under the same conditions. Paragon and *ft‐b1* NIL are highlighted in blue with the Paragon and *FT‐B1* overexpressor (OX) shown in black. (e). *FT‐B1* expression was assessed in radicles and plumules for Paragon, *ft‐b1* NIL, and *FT‐B1* OX NIL with expression normalized against *TRIAE_CS42_5AS_TGACv1_392916_AA1266200, n* = 3 for each genotype, with each sample containing radicles and plumules of three dark germinated seeds, error shown as standard error of the mean of these biological replicates [Colour figure can be viewed at http://wileyonlinelibrary.com]

*FT* and *FT*‐family genes have also been identified as key regulators in controlling the dormancy and germination of seeds. In wheat, a homologue of *FT*, *MOTHER OF FT,* was identified to increase the dormancy of seeds under lower temperatures (Nakamara et al., 2011). To investigate the possibility of *FT‐B1* being involved in regulating seed germination, we used the *ft‐b1*, *FT‐B1* OX NILs, and Paragon in seed germination assays. The assays were conducted under two temperatures because *FT1* is known to be involved in aspects of temperature regulation of seed germination in Arabidopsis (Chen et al., 2014). Seed germination is promoted by warm, dark conditions, and under these conditions, *ft‐b1* NIL showed a higher percentage of germinated seeds on the first day compared to Paragon and the *FT‐B1* OX, with all genotypes showing a near 100% germination by the second day (Figure 4b). A very similar pattern was observed under the dark low‐temperature conditions but with a delay of a day before the seeds began to germinate (Figure 4a). Under constant light conditions, *ft‐b1* NIL also showed faster germination which was more pronounced under the higher temperature conditions (Figure 4c,d). Although we cannot discount that other genes within the 7BS deletion contribute to the germination phenotype of the *ft‐b1* NIL, a role for *FT‐B1* in seed germination was supported by detection of *FT‐B1* transcripts in the radicle and plumule of germinating seeds (Figure 4e). This suggests that *FT‐B1* has a developmental role beyond flowering‐time regulation.

#### 3.1.4. The role of *FT‐B1* in temperature regulated gene expression

**Figure 5:**
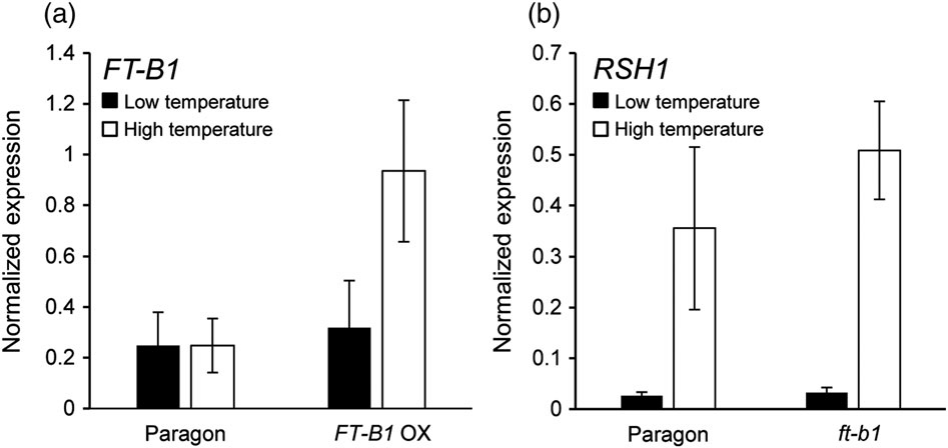
Gene expression responses to ambient temperature in Paragon and near isogenic line. (a) *FLOWERING LOCUS T* (*FT‐B1*) expression from fifth leaf stage leaf tips, normalized to *TRIAE_CS42_6DS_TGACv1_ 544038_AA1746170* in Paragon and *FT‐B1* overexpressor (OX) under high ambient temperature (black bars, 24 °C light/19 °C dark) and low ambient temperature (white bars, 18 °C light/13 °C dark). (b) *RNase‐S*‐like (*RSH1*) expression from fifth leaf stage leaf tips, normalized to *TRIAE_CS42_6DS_TGACv1_ 544038_AA1746170* in Paragon and *ft‐b1* NIL under high ambient temperature (black bars, 24 °C light/19 °C dark) and low ambient temperature (white bars, 18 °C light/13 °C dark). Values are means from two independent experiments with standard error of the mean error bars

The physiological analysis of the role of *FT* in apex transition, germination, and plant architecture (Figures 2, 3, and 4) indicated that *FT* fulfils different functions at different stages of plant development. The data suggest that the temperature dependency of apex transition and flowering acceleration was occurring at least semi‐independently of *FT*. To try to identify any role of *FT‐B1* in the temperature dependent signalling, we looked at gene expression at the fifth leaf stage under the two ambient temperature conditions, as this is a stage when stem elongation starts, and which has been shown to be a more temperature dependent process (Kiss et al., 2017). At this stage, a low, very similar level of expression is observed under both ambient temperature conditions in Paragon (Figure 5a), and in the overexpressor, an increased level of *FT‐B1* is measured which is substantially higher at elevated ambient temperatures. Interestingly, this higher level of *FT‐B1* at the fifth leaf stage under higher ambient temperatures does not lead to an earlier flowering‐time, which may indicate that the levels of *FT‐B1* at this stage are not important in determining to final flowering time. The gene expression of *FT‐B1* under standard regulation (Paragon) suggested that *FT‐B1* was not involved in the ambient temperature response of wheat plants. If this was the case, we could anticipate that the expression of genes known to be involved in the temperature response in cereals would be similar with or without *FT‐B1*. To test this, we measured the gene expression of *RNase‐S*‐like (*RSH1)* which has been shown to respond to different ambient temperatures (Hemming et al*.,* 2012). The expression of *RSH1* was extremely similar in Paragon and *ft‐b1* NIL, identifying that both genotypes could still respond to ambient temperature signals (Figure 5b).

To determine if altered levels of *FT‐B1* activity affects the expression of *FT1* homologues or *FT* paralogs, we measured transcript levels of *FT‐A1* and *FT‐D1 and FT2, FT3, FT4, FT5, and FT6* (sequences provided in Figure S4). Paragon, *ft‐b1* NIL, and *FT‐B1* OX plants were grown under SD conditions until the third leaf stage, before being transferred to LD conditions for 7 days when leaves were harvested for RNA extraction. Using qRT‐PCR, we were not able to detect transcripts for *FT‐A1*, *FT‐D1*, *FT5*, and *FT6* in all three genotypes. Transcripts for *FT2*, *FT3*, and *FT4* were detected; however, there was no significant difference in levels identified between the three genotypes (Figure S3b). Based on these results, we conclude that of the *FT*‐like genes in wheat, *FT‐B1* uniquely contributes to the flowering‐time phenotype of the *ft‐b1* and *FT‐B1* OX NILs at this stage.

## 4 DISCUSSION

The *FT* and *FT*‐like genes are known to be central in the regulation of vegetative to reproductive transition in plants; however, it is becoming apparent that these genes also have central roles in the regulation of a number of other processes, many of which are key to agricultural productivity (Pin & Nilsson, 2012). Here, we have examined regulatory roles for *FT‐B1* during processes including flowering, germination, and tiller development through genetic characterization of the *FT‐B1* locus in a spring, photoperiod sensitive wheat. By growing genotypes that differ in *FT‐B1* activity under different ambient growth temperatures, we have also increased our understanding of the contribution for *FT* to thermally responsive developmental processes in wheat.

Our results show that the absence of *FT‐B1* significantly delays flowering under both high and low ambient temperatures (Figure 3), which confirms that *FT* has a key role during the vegetative to reproductive transition in wheat. Absence of *FT‐B1* was also found to delay inflorescence development and increase spikelet number, indicating that *FT‐B1* has an important role in promoting inflorescence development between the double ridge and terminal spikelet stages. Interestingly, spikelet number, but not flowering time, was responsive to changes in ambient temperature in the *ft‐b1* NIL (Figure 3), suggesting that there are *FT‐*independent thermally responsive factors that promote inflorescence development in between the double ridge and terminal spikelet stages. Taken together with data showing that *FT‐B1* transcript levels were not significantly different between low and high ambient growth temperatures in either Paragon or the *FT‐B1* OX NIL (Figure 5), our results suggest that the accelerated flowering of domesticated wheat under increased ambient temperatures is largely independent of *FT*. This conclusion is consistent with results from barley, which showed that genes exclusively from the vernalization pathway, excluding *FT*, mediate the ambient temperature flowering response (Hemming et al., 2012). Interestingly, Paragon and the *FT‐B1* OX allele flowered at very similar times (Figure 3). This indicates that expression levels of *FT‐B1* may not be limiting in the Paragon genetic background or that increased expression of this allele in the *FT‐B1* OX NIL, which is still under native regulation, may be occurring too late in development to significantly affect flowering time. Observations from wheat‐carrying transgenic, constitutive overexpression of *FT1* from the Ubiquitin promoter, demonstrate that *FT1* is a potent activator of flowering, causing the transgenic plants to flower whilst still in callus stage (Lv et al., 2014). This suggests that expression from the Hope allele is not constitutive, which is supported by gene expression analysis shown here (Figure 5) and by previous reports showing that transcript levels of the *FT‐B1 OX* allele and its effect on flowering‐time are dependent on the developmental age of the plant and the genetic background, respectively (Nitcher et al., 2014). Similarly, our characterization of flowering‐time in the *ft‐b1* NILs shows that the *FT‐B1* locus is not singularly responsible for integrating the environmental and developmental information required to trigger transition from vegetative to floral development in hexaploid wheat and that some redundancy either with other *FT* genes or an *FT*‐independent pathway exists to ensure floral progression without *FT‐B1*, as previously reported (Boden et al., 2015; Hemming et al., 2012; Lv et al., 2014).

The characterization of the *FT‐B1* NILs also provides insights into the contribution of *FT‐B1* to plant architecture and development beyond regulation of flowering‐time. We observed that tiller numbers were lower in the *FT‐B1* OX NIL, that this effect was significant under high but not low growth temperatures and associated with accelerated flowering and reduced spikelet numbers. Given that tillers develop from vegetative nodes, this result may indicate that a higher level of *FT‐B1* accelerates development and thus ultimately results in fewer tillers as the plants reach maturity faster. This result is consistent with the observation that the *ft‐b1* generally had a higher tiller number, indicating that without *FT‐B1* more tillers could grow out, possibly at a slower rate. Interestingly, however, the phenotypic result is distinct from reports in rice that showed the *FT* homologue, *Hd3a*, promotes tiller development such that in an *Hd3a* RNAi line tiller number is decreased even though the plant flowers later (Tsuji et al., 2015). This result may reflect a different developmental strategy or greater developmental dependency on *Hd3a* for integration of flowering signals. Tissue specification of *FT‐B1* responses is further supported through the observation that under all conditions investigated and absence of *FT‐B1* accelerate the rate of germination (Figure 4). A role of *FT‐B1* in this process is further supported through its presence in radicles and plumules of germinating seeds (Figure 4e). This suggests that *FT‐B1* is important for multiple stages of development in wheat, where it represses germination but then accelerates apex transition and time to flowering.

Results presented here provide insights that could be used to further understand the flowering response of wheat to increased ambient temperatures and to identify genes that underpin these responses. The data presented show that *FT‐B1*’s role in regulating flowering‐time is largely exerted through acting early in inflorescence development and therefore highlights the importance of considering early developmental stages, in addition to the later stages of inflorescence emergence, anthesis, and grain development, when considering the effects of increased growth temperatures on reproductive development in wheat. Our observation that the flowering time delay was greatly decreased by elevated ambient temperatures when the *ft‐b1* allele was in a background with a higher proportion of landrace genetic material (Figure S1) suggests that genetic resources such as the Watkin's population (Wingen et al*.,* 2014) could be useful for identifying genes that are crucial for ambient temperature responses in wheat. This approach could be especially useful for identifying flowering‐time genes whose effect has been lost from modern wheat cultivars or is masked by key regulators, such as *VERNALIZATION1* and *PHOTOPERIOD1*. Furthermore, the *ft‐b1* allele could potentially be used as an alternative vernalization allele in areas which do not always meet vernalization requirement due to warming climate. In a spring background, the *ft‐b1* allele shows a developmental delay which could allow winter drilling but proceeds fast enough, with the additional benefit of higher spikelet number, to still flower within a standard season. Field trials would be required to ascertain the most suited environmental conditions to utilize this allele.

The research presented here identifies new applications for a well‐known gene in flowering regulation and links these applications to the role key environmental parameter, of ambient temperature. It uncovers differences in *FT‐B1* function to that of *FT*'s in other plant species and highlights the need to study the genes destined for agricultural application in as close to agricultural genetic material as possible, exampled through the differences we observed in the *ft‐b1* response between landrace and elite wheat cultivars. Identifying and understanding the role of *FT*‐dependent and independent pathways will be critical for our ability to regulate growth under warmer climates, something which will be required to develop a robust wheat yield.

## Supporting information

Figure S1: Flowering time under different ambient temperatures
Figure S2: Gene expression of activators in flowering regulation
Figure S3: Gene expression of *FT* genes in *FT‐B1* NILs
Figure S4: *FT* alleles in the Paragon cultivar
Table S1: Oligonucleotides used for determining the extent of the *FT‐B1* deletion
Table S2: List of all predicted genes and gene models within the deleted region of 7BS from the IWGSC alignment
Table S3: Oligonucleotides used in Q‐PCR analysis
