# Supplementary material for: Developmental responses of bread wheat to changes in ambient temperature following deletion of a locus that includes FLOWERING LOCUS T1
Source: Plant Cell Environ. 2018 Feb 7;41(7):1715–25. doi: 10.1111/pce.13130 (PMC6033019; doi:10.1111/pce.13130)
Supplement: Supplementary file 1 — Figure S1: Flowering time under different ambient temperatures Figure S2: Gene expression of activators in flowering regulation Figure S3: Gene expression of FT genes in FT‐B1 NILs Figure S4: FT alleles in the Paragon cultivar Table S1: Oligonucleotides used for determining the extent of the FT‐B1 deletion Table S2: List of all predicted genes and gene models within the deleted region of 7BS from the IWGSC alignment Table S3: Oligonucleotides used in Q‐PCR analysis [file PCE-41-1715-s001.zip › Supplementary Figure 4.docx]

***FT-A1 - TRIAE_CS42_7AS_TGACv1_569837_AA1825000***

TCCCCCGTACGACCCATCCGAGGCTGTGTGATCTTGCTCTCCCTCCCCGTCGTCACCATGCACGTGAAACGGGCGGGCGGGCTTTCGACACCTTCCCAGC
||||||||||||||||||||||||||||||||||||||||||||||||||||||||||||||||||||||||||||||||||||||||||||||||||||
TCCCCCGTACGACCCATCCGAGGCTGTGTGATCTTGCTCTCCCTCCCCGTCGTCACCATGCACGTGAAACGGGCGGGCGGGCTTTCGACACCTTCCCAGC
TACGGCCGGCGGCAGCTGATGAAGCTTACATCAAATCGAGCCAAGGAAGCATGCACCCCAGTCACCGTCTCGCACTAGCTAATTGGCAGACATTCCCTGT
||||||||||||||||||||||||||||||||||||||||||||||||||||||||||||||||||||||||||||||||||||||||||||||||||||
TACGGCCGGCGGCAGCTGATGAAGCTTACATCAAATCGAGCCAAGGAAGCATGCACCCCAGTCACCGTCTCGCACTAGCTAATTGGCAGACATTCCCTGT
GCCCGCTTGCCGGCCGGCCGCGGCGTGACCGCCGGTCGGCCCAGAGCCCCGGTCGCAACGCAAACCTACACGCCAGCAGGAGCAGGCAGCCAGGCACGGC
||||||||||||||||||||||||||||||||||||||||||||||||||||||||||||||||||||||||||||||||||||||||||||||||||||
GCCCGCTTGCCGGCCGGCCGCGGCGTGACCGCCGGTCGGCCCAGAGCCCCGGTCGCAACGCAAACCTACACGCCAGCAGGAGCAGGCAGCCAGGCACGGC
CTAGAAGCCACCATTAATTTGCGTGGTGATCATGATCAGGAGCTTATTACGGCAGACAGATGCATCCATCGGTCTCGCTTCTGCCTGTGGGGGTCAAAAG
||||||||||||||||||||||||||||||||||||||||||||||||||||||||||||||||||||||||||||||||||||||||||||||||||||
CTAGAAGCCACCATTAATTTGCGTGGTGATCATGATCAGGAGCTTATTACGGCAGACAGATGCATCCATCGGTCTCGCTTCTGCCTGTGGGGGTCAAAAG
CGCTGCCGGTTGTACCACGTCCACAGAACCAATTCAATAGAGAGAGGCGACGAGATTCCGTGGCCACGCCAGCTCGGCAGCGCCAAGGAGTGCTAGAGCG
||||||||||||||||||||||||||||||||||||||||||||||||||||||||||||||||||||||||||||||||||||||||||||||||||||
CGCTGCCGGTTGTACCACGTCCACAGAACCAATTCAATAGAGAGAGGCGACGAGATTCCGTGGCCACGCCAGCTCGGCAGCGCCAAGGAGTGCTAGAGCG
GAGAGCAGCGGCTGAACTGGTCTGGACATGGACATGGACATGGAACATGCCAGGCTGAGCTTTTCGGCCCTATATAAAGTGGCCACCGGCCGTGGGGCAA
||||||||||||||||||||||||||||||||||||||||||||||||||||||||||||||||||||||||||||||||||||||||||||||||||||
GAGAGCAGCGGCTGAACTGGTCTGGACATGGACATGGACATGGAACATGCCAGGCTGAGCTTTTCGGCCCTATATAAAGTGGCCACCGGCCGTGGGGCAA
CACTCATCATCACCACTTCCTCAATTCACAGCTTACGCTTACTCTTGCTCCCTCTGCTGCTAGCTAGCCGGTCGATCTACACTAGGAAGAAGGAAGGGGA
||||||||||||||||||||||||||||||||||||||||||||||||||||||||||||||||||||||||||||||||||||||||||||||||||||
CACTCATCATCACCACTTCCTCAATTCACAGCTTACGCTTACTCTTGCTCCCTCTGCTGCTAGCTAGCCGGTCGATCTACACTAGGAAGAAGGAAGGGGA
AATGGCCGGGAGGGACAGGGACCCGCTGGTGGTTGGCAGGGTTGTGGGAGACGTGCTGGACCCCTTTGTCCGGACCACCAACCTCAGGGTGACCTTCGGG
||||||||||||||||||||||||||||||||||||||||||||||||||||||||||||||||||||||||||||||||||||||||||||||||||||
AATGGCCGGGAGGGACAGGGACCCGCTGGTGGTTGGCAGGGTTGTGGGAGACGTGCTGGACCCCTTTGTCCGGACCACCAACCTCAGGGTGACCTTCGGG
AACAGGACCGTGTCCAACGGCTGCGAGCTCAAGCCGTCCATGGTCGCCCAGCAGCCCAGGGTTGAGGTGGGCGGCAATGAGATGAGGACCTTCTACACAC
||||||||||||||||||||||||||||||||||||||||||||||||||||||||||||||||||||||||||||||||||||||||||||||||||||
AACAGGACCGTGTCCAACGGCTGCGAGCTCAAGCCGTCCATGGTCGCCCAGCAGCCCAGGGTTGAGGTGGGCGGCAATGAGATGAGGACCTTCTACACAC
TCGTACGTACACAGTCACTATCTAATGCCTATATATATGTTAAGCTCTGAAAGTGCTCGCCACACGCACATGATCGATCGAGCTCTATATATAGTACGTG
||||||||||||||||||||||||||||||||||||||||||||||||||||||||||||||||||||||||||||||||||||||||||||||||||||
TCGTACGTACACAGTCACTATCTAATGCCTATATATATGTTAAGCTCTGAAAGTGCTCGCCACACGCACATGATCGATCGAGCTCTATATATAGTACGTG
TGGGAAGATGATTCTCGATGCTTCTGTTCACAGCATGTTTGTCTTGGCAGGCACATGACTAATGCTCCATCTTGCATATGGCTCTGTGCTAGCTCTCTGG
||||||||||||||||||||||||||||||||||||||||||||||||||||||||||||||||||||||||||||||||||||||||||||||||||||
TGGGAAGATGATTCTCGATGCTTCTGTTCACAGCATGTTTGTCTTGGCAGGCACATGACTAATGCTCCATCTTGCATATGGCTCTGTGCTAGCTCTCTGG
TGTTCATCATGATTTTCTATGCTTCTTTTCTATTCGGGGAACATTGATTTTCGATGCTTCTGTTGACATGTTTTATGTTTGTTCTGGCAAGCACACGACT
||||||||||||||||||||||||||||||||||||||||||||||||||||||||||||||||||||||||||||||||||||||||||||||||||||
TGTTCATCATGATTTTCTATGCTTCTTTTCTATTCGGGGAACATTGATTTTCGATGCTTCTGTTGACATGTTTTATGTTTGTTCTGGCAAGCACACGACT
AATTAAAGCTCGATCTTAAATATATGCTTATGCACGTAGTACTCTCTACGTCTCTAGTATTGATCATGATGTGCACGCGTGTACTGCCTGCAGGTGATGG
|||||||||||||||||||||||||||||||||||||||||||||||||||||||||||||||||||||||||||||||||||||||| |||||||||||
AATTAAAGCTCGATCTTAAATATATGCTTATGCACGTAGTACTCTCTACGTCTCTAGTATTGATCATGATGTGCACGCGTGTACTGCCCGCAGGTGATGG
TAGACCCAGATGCTCCAAGTCCAAGCGATCCCAACCTTAGGGAGTATCTCCACTGGTAAGTAAATTTGTAGCTCAGTTGAATAATTTCTCTTTCCCTAGA
||||||||||||||||||||||||||||||||||||||||||||||||||||||||||||||||||||||||||||||||||||||||||||||||||||
TAGACCCAGATGCTCCAAGTCCAAGCGATCCCAACCTTAGGGAGTATCTCCACTGGTAAGTAAATTTGTAGCTCAGTTGAATAATTTCTCTTTCCCTAGA
TATACACACTAGCTCATGTGTGTGTGTGTGTGTGCGCGCGTGTGCATCTACATGTGTGTGCAGGCTTGTGACAGATATCCCCGGTACAACTGGTGCCTCG
||||||||||||||||||||||||||||||||||||||||||||||||||||||||||||||||||||||||||||||||||||||||||||||||||||
TATACACACTAGCTCATGTGTGTGTGTGTGTGTGCGCGCGTGTGCATCTACATGTGTGTGCAGGCTTGTGACAGATATCCCCGGTACAACTGGTGCCTCG
TTCGGGCAGGAAGTGATGTGCTATGAGAGCCCTCGTCCGACCATGGGGATCCACCGCTTCGTGCTCGTGCTCTTCCAGCAGCTCGGCCGGCAGACGGTGT
||||||||||||||||||||||||||||||||||||||||||||||||||||||||||||||||||||||||||||||||||||||||||||||||||||
TTCGGGCAGGAAGTGATGTGCTATGAGAGCCCTCGTCCGACCATGGGGATCCACCGCTTCGTGCTCGTGCTCTTCCAGCAGCTCGGCCGGCAGACGGTGT
ACGCCCCCGGGTGGCGCCAGAACTTCAACACCAGGGACTTCGCCGAGCTCTACAACCTTGGCCCGCCCGTCGCCGCCGTCTACTTCAACTGCCAGCGTGA
|||||||||||||||||||||||||||||||||||||||||||||||||||||||||| |||||||||||||||||||||||||||||||||||||||||
ACGCCCCCGGGTGGCGCCAGAACTTCAACACCAGGGACTTCGCCGAGCTCTACAACCTCGGCCCGCCCGTCGCCGCCGTCTACTTCAACTGCCAGCGTGA
GGCCGGCTCCGGTGGCAGGAGGATGTACAATTGACCTACCCATGGCCCACGTACGCCGCCCGCAAAGTCAGCAAATTATCCAACGTGGCTAGTTTACTAG
||||||||||||||||||||||||||||||||||||||||||||||||||||||||||||||||||||||||||||||||||||||||||||||||||||
GGCCGGCTCCGGTGGCAGGAGGATGTACAATTGACCTACCCATGGCCCACGTACGCCGCCCGCAAAGTCAGCAAATTATCCAACGTGGCTAGTTTACTAG
TATATAGTTTGTCATAAGAAGCCAGCCACGAATTAATTAAGCATTATCTATATATTGGCAACACATACACTACATATATGCATACTATGATCGATGTAAA
||||||||||||||||||||||||||||||||||||||||||||||||||||||||||||||||||||||||||||||||||||||||||||||||||||
TATATAGTTTGTCATAAGAAGCCAGCCACGAATTAATTAAGCATTATCTATATATTGGCAACACATACACTACATATATGCATACTATGATCGATGTAAA
ACTAGCCGTACGCATATATGCATATCAACGGCTAATTAATTAATTAAGGGGGGATGAACCCTAGATCAATGGCTTGGTACTGCACTATATATATAGTCTG
||||||||||||||||||||||||||||||||||||||||||||||||||||||||||||||||||||||||||||||||||||||||||||||||||||
ACTAGCCGTACGCATATATGCATATCAACGGCTAATTAATTAATTAAGGGGGGATGAACCCTAGATCAATGGCTTGGTACTGCACTATATATATAGTCTG
CAATAAACTGATGCCAATAGTATACAGCACACAATATTGGAGGAGCTACACGCCATGTGCAACTTAGAGCTACCTGGTACATATCTGCAGGTTGGTCTTG
||||||||||||||||||||||||||||||||||||||||||||||||||||||||||||||||||||||||||||||||||||||||||||||||||||
CAATAAACTGATGCCAATAGTATACAGCACACAATATTGGAGGAGCTACACGCCATGTGCAACTTAGAGCTACCTGGTACATATCTGCAGGTTGGTCTTG
ACTCTTGTGTGTTCACTTATGCGTGCATGAACATCAGTCAATCATATAGACATAGTTATGCATGGGAGACAAACATGTAATTGACAGATACTGCTACAAG
||||||||||||||||||||||||||||||||||||||||||||||||||||||||||||||||||||||||||||||||||||||||||||||||||||
ACTCTTGTGTGTTCACTTATGCGTGCATGAACATCAGTCAATCATATAGACATAGTTATGCATGGGAGACAAACATGTAATTGACAGATACTGCTACAAG
ACACAGTGATATGTACACACTTAGCGCAGTAGCAAAGCACATGCATGTGTTGGGCCTTGTACAACTTCCTCATGATGATGGCAATATAATCAAGTGTCAA
||||||||||||||||||||||||||||||||||||||||||||||||||||||||||||||||||||||||||||||||||||||||||||||||||||
ACACAGTGATATGTACACACTTAGCGCAGTAGCAAAGCACATGCATGTGTTGGGCCTTGTACAACTTCCTCATGATGATGGCAATATAATCAAGTGTCAA
GTTATGTATGAGACCGGTGGCTTCTCTGTGATGATGAACAATATTAATGTCTGGTGCGCTAGCTCATCAATTTGTGTCTTATACTAGATGCTTCAGCTGC
||||||||||||||||||||||||||||||||||||||||||||||||||||||||||||||||||||||||||||||||||||||||||||||||||||
GTTATGTATGAGACCGGTGGCTTCTCTGTGATGATGAACAATATTAATGTCTGGTGCGCTAGCTCATCAATTTGTGTCTTATACTAGATGCTTCAGCTGC
CTTTGCGTCGATCAGCAGAAGACACACCTTTTTTTTTTAGAAACAGCAGAAGACACACCTTTTTGTTTTTGAGTGAAGCAGAAGAAGACACACCTAAACA
||||||||||||||||||||||||||||||||||||||||||||||||||||||||||||||||||||||||||||||||||||||||||||||||||||
CTTTGCGTCGATCAGCAGAAGACACACCTTTTTTTTTTAGAAACAGCAGAAGACACACCTTTTTGTTTTTGAGTGAAGCAGAAGAAGACACACCTAAACA
TATGTTTAGGTGTTCCTAAACATATGTTCCAGTAATGGGCTTCCGCCAGCATGTTTCATAGCCATGGGCTTCTCATACTTGCGGCCTGACGGGCCCCAAA
||||||||||||||||||||||||||||||||||||||||||||||||||||||||||||||||||||||||||||||||||||||||||||||||||||
TATGTTTAGGTGTTCCTAAACATATGTTCCAGTAATGGGCTTCCGCCAGCATGTTTCATAGCCATGGGCTTCTCATACTTGCGGCCTGACGGGCCCCAAA
TTGGACGTGAACTACGCTGAAACGTGGTCACCCTGCAAACTGGCCCAGATAGTGGAACGGCTAACAAAGTTGGGCCGGCCGGGAAACGGGCACCCTATTT
||||||||||||||||||||||||||||||||||||||||||||||||||||||||||||||||||||||||||||||||||||||||||||||||||||
TTGGACGTGAACTACGCTGAAACGTGGTCACCCTGCAAACTGGCCCAGATAGTGGAACGGCTAACAAAGTTGGGCCGGCCGGGAAACGGGCACCCTATTT
CTCGCGTTCAACGAGACGAGAGGATGCGCTCGCTTCTGGGGGCGACCCAGATGGGCCAGCCCAGCCGCGGGAGCCACAGCCAACTTTTTTCTGTTTTCAT
||||||||||||||||||||||||||||||||||||||||||||||||||||||||||||||||||||||||||||||||||||||||||||||||||||
CTCGCGTTCAACGAGACGAGAGGATGCGCTCGCTTCTGGGGGCGACCCAGATGGGCCAGCCCAGCCGCGGGAGCCACAGCCAACTTTTTTCTGTTTTCAT
TTTTACTTTATTTTTGTACTTTTTTTATACTTCTAAATATTCTACATATATACATATATATTACAAAAATACTTCAAAAAAACATTTGAAAAATTGTTGA
||||||||||||||||||||||||||||||||||||||||||||||||||||||||||||||||||||||||||||||||||||||||||||||||||||
TTTTACTTTATTTTTGTACTTTTTTTATACTTCTAAATATTCTACATATATACATATATATTACAAAAATACTTCAAAAAAACATTTGAAAAATTGTTGA
ACAAGTATTAGAAAATGTTTACCAAGGATTTGAAAAATGTTGAAGAAGTATTTGAAAATGTTGAACAA
||||||||||||||||||||||||||||||||||||||||||||||||||||||||||||||||||||
ACAAGTATTAGAAAATGTTTACCAAGGATTTGAAAAATGTTGAAGAAGTATTTGAAAATGTTGAACAA

***FT-B1 - TRIAE_CS42_7BS_TGACv1_595178_AA1958810***

CAAGAATGAAACCTGTCCTCCAATGATCTCCCCCGTACGACCCATCCGAGGCTGTGTGATCTTGCTCTCCCTCCCCGTCGTCACCATGCACGTGAAACGG
||||||||||||||||||||||||||||||||||||||||||||||||||||||||||||||||||||||||||||||||||||||||||||||||||||
CAAGAATGAAACCTGTCCTCCAATGATCTCCCCCGTACGACCCATCCGAGGCTGTGTGATCTTGCTCTCCCTCCCCGTCGTCACCATGCACGTGAAACGG
CCGGGCTTTCGACACCTTCCCAGCTACGGCCGGCGGCAGCTGATGAAGCTTACATCAAATCGAGCCAAGGAAGCCTGCACCCCAGTCACCGTCGGCCGCT
||||||||||||||||||||||||||||||||||||||||||||||||||||||||||||||||||||||||||||||||||||||||||||||||||||
CCGGGCTTTCGACACCTTCCCAGCTACGGCCGGCGGCAGCTGATGAAGCTTACATCAAATCGAGCCAAGGAAGCCTGCACCCCAGTCACCGTCGGCCGCT
AGCTAATTGGCAGACATTCCCTGTGCCCGCTTGCCGGCCGGCCGCGGCGTGACCGCCGGTCGGCCCAGAGCCCCGGACGCAACGCAAACCTACACCCCAG
||||||||||||||||||||||||||||||||||||||||||||||||||||||||||||||||||||||||||||||||||||||||||||||||||||
AGCTAATTGGCAGACATTCCCTGTGCCCGCTTGCCGGCCGGCCGCGGCGTGACCGCCGGTCGGCCCAGAGCCCCGGACGCAACGCAAACCTACACCCCAG
CAGGCACGGACAGAAACCACCATTAATTTGCGTGGTGATCATGATCAGGAGCTTATTACGGCAGACAGATGCATCCATCGGTCTCGCTTCTGCCTGTGGG
||||||||||||||||||||||||||||||||||||||||||||||||||||||||||||||||||||||||||||||||||||||||||||||||||||
CAGGCACGGACAGAAACCACCATTAATTTGCGTGGTGATCATGATCAGGAGCTTATTACGGCAGACAGATGCATCCATCGGTCTCGCTTCTGCCTGTGGG
GGTCAAAAGCGCTGCCGGTTACACCACATCCACAGAACCAATTGTACAGAGGGAGGCGACGAGATTCCGTGGCCACGCCAGCTCGGCAGCGCCAAGGAGT
||||||||||||||||||||||||||||||||||||||||||||||||||||||||||||||||||||||||||||||||||||||||||||||||||||
GGTCAAAAGCGCTGCCGGTTACACCACATCCACAGAACCAATTGTACAGAGGGAGGCGACGAGATTCCGTGGCCACGCCAGCTCGGCAGCGCCAAGGAGT
ACTAGAGCGGCGAGCAGCGGCTGAACTGGTCTGGACATGGACATGTACCCTGCGTGAGCTTTTCGGCCCTATATAAAGTGGCCACCGGCCGTGGGGCAAC
||||||||||||||||||||||||||||||||||||||||||||||||||||||||||||||||||||||||||||||||||||||||||||||||||||
ACTAGAGCGGCGAGCAGCGGCTGAACTGGTCTGGACATGGACATGTACCCTGCGTGAGCTTTTCGGCCCTATATAAAGTGGCCACCGGCCGTGGGGCAAC
ACTCATCATCACCACTTCCTCAATTCACAGCTTACTCCTGCTCCAGAGAACTTCTGCTTGCTGCCTCGTACCCTAGCTAGCAAGGCAAGCTAGCCGGTCG
||||||||||||||||||||||||||||||||||||||||||||||||||||||||||||||||||||||||||||||||||||||||||||||||||||
ACTCATCATCACCACTTCCTCAATTCACAGCTTACTCCTGCTCCAGAGAACTTCTGCTTGCTGCCTCGTACCCTAGCTAGCAAGGCAAGCTAGCCGGTCG
ATCTATACTAGGAAGGAAGGGCTAATGGCCGGTAGGGATAGGGACCCGCTGGTGGTTGGCAGGGTTGTGGGGGACGTGCTGGACCCCTTCGTCCGGACCA
||||||||||||||||||||||||||||||||||||||||||||||||||||||||||||||||||||||||||||||||||||||||||||||||||||
ATCTATACTAGGAAGGAAGGGCTAATGGCCGGTAGGGATAGGGACCCGCTGGTGGTTGGCAGGGTTGTGGGGGACGTGCTGGACCCCTTCGTCCGGACCA
CCAACCTCAGGGTGACCTTCGGGAACAGGACCGTGTCCAACGGCTGCGAGCTCAAGCCGTCCATGGTCGCCCAGCAGCCCAGGGTTGAGGTGGGCGGCAA
||||||||||||||||||||||||||||||||||||||||||||||||||||||||||||||||||||||||||||||||||||||||||||||||||||
CCAACCTCAGGGTGACCTTCGGGAACAGGACCGTGTCCAACGGCTGCGAGCTCAAGCCGTCCATGGTCGCCCAGCAGCCCAGGGTTGAGGTGGGCGGCAA
TGAGATGAGGACCTTCTACACACTCGTACGTACACAGTCACTATCTAATGCCAATTTATCTCTGAAAGTGCTCACCACACGCACATGATCGATCGAGCTC
||||||||||||||||||||||||||||||||||||||||||||||||||||||||||||||||||||||||||||||||||||||||||||||||||||
TGAGATGAGGACCTTCTACACACTCGTACGTACACAGTCACTATCTAATGCCAATTTATCTCTGAAAGTGCTCACCACACGCACATGATCGATCGAGCTC
GATCTATAGTACGTGAGGGAAATTGATTTTCGATGCTTCTGTTCACATGTTTGCCTCAGCAAGCACATGACTAATGCTCCATCTTGCATATGTCTCTGTG
||||||||||||||||||||||||||||||||||||||||||||||||||||||||||||||||||||||||||||||||||||||||||||||||||||
GATCTATAGTACGTGAGGGAAATTGATTTTCGATGCTTCTGTTCACATGTTTGCCTCAGCAAGCACATGACTAATGCTCCATCTTGCATATGTCTCTGTG
CCCTCTGGTGTTGATCATGATTTTTCTATGCTTCTTCTATGTTCGGGGAGCATTTATTTTTTATGCTTCTCTTGACATGTTTCATGTTTGTCCTAGCAAG
||||||||||||||||||||||||||||||||||||||||||||||||||||||||||||||||||||||||||||||||||||||||||||||||||||
CCCTCTGGTGTTGATCATGATTTTTCTATGCTTCTTCTATGTTCGGGGAGCATTTATTTTTTATGCTTCTCTTGACATGTTTCATGTTTGTCCTAGCAAG
CACACGAGTAATTAAAGCTCGATCTTAAATACTCTCTCCGTCCGAATAAATGTACTTCTAGCTTTTGTCTTAAGTCAAAGTTTTAAAATTTTGACCAACT
||||||||||||||||||||||||||||||||||||||||||||||||||||||||||||||||||||||||||||||||||||||||||||||||||||
CACACGAGTAATTAAAGCTCGATCTTAAATACTCTCTCCGTCCGAATAAATGTACTTCTAGCTTTTGTCTTAAGTCAAAGTTTTAAAATTTTGACCAACT
TTATAGGAAAAAGTAGCAGCATTTATGACACTAAATTAGTATCACTAGATTCGTTTTGAAATGTATTTTCATAATATATCAATTTGATATTATATATGTT
||||||||||||||||||||||||||||||||||||||||||||||||||||||||||||||||||||||||||||||||||||||||||||||||||||
TTATAGGAAAAAGTAGCAGCATTTATGACACTAAATTAGTATCACTAGATTCGTTTTGAAATGTATTTTCATAATATATCAATTTGATATTATATATGTT
ACTACTTATTTGTATATAGTTGGTCAAAGTTTTAAAACTTTGACTTAGGATAAAAACTAGAAGTACACTTATTCGTGGACGGAGGGAGTATATGCTTATG
||||||||||||||||||||||||||||||||||||||||||||||||||||||||||||||||||||||||||||||||||||||||||||||||||||
ACTACTTATTTGTATATAGTTGGTCAAAGTTTTAAAACTTTGACTTAGGATAAAAACTAGAAGTACACTTATTCGTGGACGGAGGGAGTATATGCTTATG
TAGGTAGTACTCTCTACTTTGATCATGATGTGCACGCGTTTACTGCCCGCAGGTGATGGTAGACCCAGATGCTCCAAGTCCAAGCGATCCCAACCTTAGG
||||||||||||||||||||||||||||||||||||||||||||||||||||||||||||||||||||||||||||||||||||||||||||||||||||
TAGGTAGTACTCTCTACTTTGATCATGATGTGCACGCGTTTACTGCCCGCAGGTGATGGTAGACCCAGATGCTCCAAGTCCAAGCGATCCCAACCTTAGG
GAGTATCTCCACTGGTAAGTACTAAATTTGTAACTCAGTTGAATAATTTCTCTGTCCCTAGATATACACACTAGCTCATGTGTGCGTGTGTGTGTCTACA
||||||||||||||||||||||||||||||||||||||||||||||||||||||||||||||||||||||||||||||||||||||||||||||||||||
GAGTATCTCCACTGGTAAGTACTAAATTTGTAACTCAGTTGAATAATTTCTCTGTCCCTAGATATACACACTAGCTCATGTGTGCGTGTGTGTGTCTACA
TGTGTGTGCAGGCTTGTGACAGATATCCCCGGTACAACTGGTGCGTCGTTCGGGCAGGAGGTGATGTGCTACGAGAGCCCTCGTCCGACCATGGGGATCC
||||||||||||||||||||||||||||||||||||||||||||||||||||||||||||||||||||||||||||||||||||||||||||||||||||
TGTGTGTGCAGGCTTGTGACAGATATCCCCGGTACAACTGGTGCGTCGTTCGGGCAGGAGGTGATGTGCTACGAGAGCCCTCGTCCGACCATGGGGATCC
ACCGCTTCGTGCTCGTACTCTTCCAGCAGCTCGGGCGGCAGACGGTGTACGCCCCCGGGTGGCGCCAGAACTTCAACACCAGGGACTTCGCCGAGCTCTA
||||||||||||||||||||||||||||||||||||||||||||||||||||||||||||||||||||||||||||||||||||||||||||||||||||
ACCGCTTCGTGCTCGTACTCTTCCAGCAGCTCGGGCGGCAGACGGTGTACGCCCCCGGGTGGCGCCAGAACTTCAACACCAGGGACTTCGCCGAGCTCTA
CAACCTCGGCCCGCCTGTCGCCGCCGTCTACTTCAACTGCCAGCGTGAGGCCGGCTCCGGCGGCAGGAGGATGTACAATTGATCTACCCACGGCCCTCGT
||||||||||||||||||||||||||||||||||||||||||||||||||||||||||||||||||||||||||||||||||||||||||||||||||||
CAACCTCGGCCCGCCTGTCGCCGCCGTCTACTTCAACTGCCAGCGTGAGGCCGGCTCCGGCGGCAGGAGGATGTACAATTGATCTACCCACGGCCCTCGT
ACGCCACCAGCCCGCCGCCAAGTCAGCAAATTATCCAACGTGGCTAGTTTACTAGTATATAGTTTGTGATAAGAAGCCAGCCACGAATTAAGCATTACCT
||||||||||||||||||||||||||||||||||||||||||||||||||||||||||||||||||||||||||||||||||||||||||||||||||||
ACGCCACCAGCCCGCCGCCAAGTCAGCAAATTATCCAACGTGGCTAGTTTACTAGTATATAGTTTGTGATAAGAAGCCAGCCACGAATTAAGCATTACCT
ATATATTGGCAACACATACACTACATATATGCATACTATGATCGATGTATAACTAGCCGCATGCATATATGCAATCAACGGCTAATTAAGGGGGGGTGAA
||||||||||||||||||||||||||||||||||||||||||||||||||||||||||||||||||||||||||||||||||||||||||||||||||||
ATATATTGGCAACACATACACTACATATATGCATACTATGATCGATGTATAACTAGCCGCATGCATATATGCAATCAACGGCTAATTAAGGGGGGGTGAA
CCCTAGATCAATGGCTTGGTACTGCACTATATAAATATAGTCTGCAATAAACTGATGCCAATAGTATACAGCACACAAATATTGGAGGAGCTACACGCCA
||||||||||||||||||||||||||||||||||||||||||||||||||||||||||||||||||||||||||||||||||||||||||||||||||||
CCCTAGATCAATGGCTTGGTACTGCACTATATAAATATAGTCTGCAATAAACTGATGCCAATAGTATACAGCACACAAATATTGGAGGAGCTACACGCCA
TGTGCAACTTAGTGCTACCTGGTACATATCTGCAGGTTGGTCTTGTGCGTTCACTTATGCGTGCATGAACATCAGTCAATCATATAGACATAGTTATGCA
||||||||||||||||||||||||||||||||||||||||||||||||||||||||||||||||||||||||||||||||||||||||||||||||||||
TGTGCAACTTAGTGCTACCTGGTACATATCTGCAGGTTGGTCTTGTGCGTTCACTTATGCGTGCATGAACATCAGTCAATCATATAGACATAGTTATGCA
TGGGAGACAAACATGTAACTGACAGCGACTGCTACAAGACACAGTGATATGTACGCACTTAGCGCAGTAGCAAAGCACATGCATGTGTTGGGTCTTGTAC
||||||||||||||||||||||||||||||||||||||||||||||||||||||||||||||||||||||||||||||||||||||||||||||||||||
TGGGAGACAAACATGTAACTGACAGCGACTGCTACAAGACACAGTGATATGTACGCACTTAGCGCAGTAGCAAAGCACATGCATGTGTTGGGTCTTGTAC
AACTTCCTCATGATTATGGCAATATAATCAAGTGTCATGTTATGTATGAGACCGGTGGCTTCTCTCTGTGATTATGAGCAATATTAATGTCTGGTGCGCT
||||||||||||||||||||||||||||||||||||||||||||||||||||||||||||||||||||||||||||||||||||||||||||||||||||
AACTTCCTCATGATTATGGCAATATAATCAAGTGTCATGTTATGTATGAGACCGGTGGCTTCTCTCTGTGATTATGAGCAATATTAATGTCTGGTGCGCT
CATCAATCTGTGTCTTATGCTATATGCTAGCTAGGTAGCGTCTCTTTCAGTTGAGTTCCACGGTCTGCCGTCCTAGGTTTCAGAAATATCTTGTAGTATC
||||||||||||||||||||||||||||||||||||||||||||||||||||||||||||||||||||||||||||||||||||||||||||||||||||
CATCAATCTGTGTCTTATGCTATATGCTAGCTAGGTAGCGTCTCTTTCAGTTGAGTTCCACGGTCTGCCGTCCTAGGTTTCAGAAATATCTTGTAGTATC
ATGCTTCAGAAATATCTTTCAGAAGTTTCTTGCTTCCCAGCACCCAAAGTGCAGATAATAATCGGTTCATCGTCAGCGCTTTGCGCCTTTTCGAACTTCA
||||||||||||||||||||||||||||||||||||||||||||||||||||||||||||||||||||||||||||||||||||||||||||||||||||
ATGCTTCAGAAATATCTTTCAGAAGTTTCTTGCTTCCCAGCACCCAAAGTGCAGATAATAATCGGTTCATCGTCAGCGCTTTGCGCCTTTTCGAACTTCA
AAGTGAGTATATATCGACAACTTCTTTAACTTTTTCCTGGCCTTGGCTTGATTGGACTTTCTCAGCTCTTGCCATTCAGCTTCCTCTCTCCTCTACCCGC
||||||||||||||||||||||||||||||||||||||||||||||||||||||||||||||||||||||||||||||||||||||||||||||||||||
AAGTGAGTATATATCGACAACTTCTTTAACTTTTTCCTGGCCTTGGCTTGATTGGACTTTCTCAGCTCTTGCCATTCAGCTTCCTCTCTCCTCTACCCGC
-AAAAAAAAAAAAGCTTCCTCTCTCCTCTCTCAACTTGTCTTTCGGTAGATGGCATTAGGAAGTAGGGCTCCATGCTTAGAAGCAAGTACTCCCTCTGTA
 |||||||||||||||||||||||||||||||||||||||||||||||||||||||||||||||||||||||||||||||||||||||||||||||||||
AAAAAAAAAAAAAGCTTCCTCTCTCCTCTCTCAACTTGTCTTTCGGTAGATGGCATTAGGAAGTAGGGCTCCATGCTTAGAAGCAAGTACTCCCTCTGTA
AACTAATATAAGAGCATTTAGATCACTAAAGTAGTGTTCTAAACGCTCTTATATTAGTTTACGGAGGGAGTAGTTTCGATCCTTGTGTTTTATTCTACTA
||||||||||||||||||||||||||||||||||||||||||||||||||||||||||||||||||||||||||||||||||||||||| ||||||||||
AACTAATATAAGAGCATTTAGATCACTAAAGTAGTGTTCTAAACGCTCTTATATTAGTTTACGGAGGGAGTAGTTTCGATCCTTGTGTTCTATTCTACTA
CAGTATTATATGAACTCAACGAAAATAAACCTACACGCGTAAGGATGTTATTATTCCTGGGTAAGGACTAAGGAGAGGTAGCTAGAGCATCAACCTTTGG
||||||||||||||||||||||||||||||||||||||||||||||||||||||||||||||||||||||||||||||||||||||||||||||||||||
CAGTATTATATGAACTCAACGAAAATAAACCTACACGCGTAAGGATGTTATTATTCCTGGGTAAGGACTAAGGAGAGGTAGCTAGAGCATCAACCTTTGG
TGCACGATTTGTGGGCGCAG
||||||||||||||||||||
TGCACGATTTGTGGGCGCAG

***FT-D1 - TRIAE_CS42_7DS_TGACv1_621674_AA2022890***

TAGGGTAGTAAAAGATGCGCTCAAAGAGAACCAGGGCAACAAAAGTGGAGGGTATATTAAAAAGAAATTCAGAAGAAGAAAAAAACAAAGAGTTTATTTC
||||||||||||||||||||||||||||||||||||||||||||||||||||||||||||||||||||||||||||||||||||||||||||||||||||
TAGGGTAGTAAAAGATGCGCTCAAAGAGAACCAGGGCAACAAAAGTGGAGGGTATATTAAAAAGAAATTCAGAAGAAGAAAAAAACAAAGAGTTTATTTC
GAGCCGAGGGACAGGTGGAGCATTATCGTCGTCCTTGAGCCTGGCCCGGCACTGTACATATATTCTCGCGGGAAAATGATCGTGGGGGGTCTTTTTTCCT
||||||||||||||||||||||||||||||||||||||||||||||||||||||||||||||||||||||||||||||||||||||||||||||||||||
GAGCCGAGGGACAGGTGGAGCATTATCGTCGTCCTTGAGCCTGGCCCGGCACTGTACATATATTCTCGCGGGAAAATGATCGTGGGGGGTCTTTTTTCCT
AATACGGCACGCGTCCCGTTTAAGCTAACGTTTCGGGAAAGTCCGTCTTCTTCATCCTTTCCGACCAACACACAGAGCTAGCCCCGCGCCCACAGCTGAA
||||||||||||||||||||||||||||||||||||||||||||||||||||||||||||||||||||||||||||||||||||||||||||||||||||
AATACGGCACGCGTCCCGTTTAAGCTAACGTTTCGGGAAAGTCCGTCTTCTTCATCCTTTCCGACCAACACACAGAGCTAGCCCCGCGCCCACAGCTGAA
AGAATAACCCTGCCCTCCAATGATCTCCCCCGTACGACCCATCCGAGGCTGTGTGATCTTGCTCTCCCTCCCCGTCGTCACCATGCACGTGAAACAGGTC
||||||||||||||||||||||||||||||||||||||||||||||||||||||||||||||||||||||||||||||||||||||||||||||||||||
AGAATAACCCTGCCCTCCAATGATCTCCCCCGTACGACCCATCCGAGGCTGTGTGATCTTGCTCTCCCTCCCCGTCGTCACCATGCACGTGAAACAGGTC
GGCTTTCGACACCTGCCCGGCTACGGCCGGCGGCAGCTGATGAAGCTTACATCAAATCGAGCCAAGGAAGCATGCACCCCGGTCACCGTCTCGCGCTAGC
||||||||||||||||||||||||||||||||||||||||||||||||||||||||||||||||||||||||||||||||||||||||||||||||||||
GGCTTTCGACACCTGCCCGGCTACGGCCGGCGGCAGCTGATGAAGCTTACATCAAATCGAGCCAAGGAAGCATGCACCCCGGTCACCGTCTCGCGCTAGC
TAATTGGCAGACATTCCCTGTGCCCGCCTGCCGGCCGGCCGCGGCGTGACCGCCGGTCGGCCCACGGCCCCAGACGCAACGCCAACCTACACCCGAGCAC
||||||||||||||||||||||||||||||||||||||||||||||||||||||||||||||||||||||||||||||||||||||||||||||||||||
TAATTGGCAGACATTCCCTGTGCCCGCCTGCCGGCCGGCCGCGGCGTGACCGCCGGTCGGCCCACGGCCCCAGACGCAACGCCAACCTACACCCGAGCAC
GCACCGCCTGGAAACCACCATTAATTTGCGTGGTGATCATCATCAGGAGCTTATATTACGGCAGACAGATCCATCCATCGGTCTCTCGCTGCTGCCTGTG
||||||||||||||||||||||||||||||||||||||||||||||||||||||||||||||||||||||||||||||||||||||||||||||||||||
GCACCGCCTGGAAACCACCATTAATTTGCGTGGTGATCATCATCAGGAGCTTATATTACGGCAGACAGATCCATCCATCGGTCTCTCGCTGCTGCCTGTG
GCTGTGGGGGTCAAAAGCGCTGCCGGTTGCACCACGTCCACAGAAGCAATTCTACAGAGGGAGGCGACGAGATTCCGTGGCCACGCCAGCTCGGCAGCGC
||||||||||||||||||||||||||||||||||||||||||||||||||||||||||||||||||||||||||||||||||||||||||||||||||||
GCTGTGGGGGTCAAAAGCGCTGCCGGTTGCACCACGTCCACAGAAGCAATTCTACAGAGGGAGGCGACGAGATTCCGTGGCCACGCCAGCTCGGCAGCGC
CAAGGAGTACTAGAGCGGCGAGCAGCGGCTGAACTGGTCTGGACATGGACATGTACCCTGCCTGAGCTTTTCGGTCCTATATAAAGTGGCCACCGGCCGT
||||||||||||||||||||||||||||||||||||||||||||||||||||||||||||||||||||||||||||||||||||||||||||||||||||
CAAGGAGTACTAGAGCGGCGAGCAGCGGCTGAACTGGTCTGGACATGGACATGTACCCTGCCTGAGCTTTTCGGTCCTATATAAAGTGGCCACCGGCCGT
GGGGCAACACTCATCATCACCACCTCCTCAATTCACAGCTTACTCCTGCTCCAGAGAACCTCTGCTGCTTGCTCCCTCGTATCCTAGCTAGCAGGCCGGT
||||||||||||||||||||||||||||||||||||||||||||||||||||||||||||||||||||||||||||||||||||||||||||||||||||
GGGGCAACACTCATCATCACCACCTCCTCAATTCACAGCTTACTCCTGCTCCAGAGAACCTCTGCTGCTTGCTCCCTCGTATCCTAGCTAGCAGGCCGGT
CGATCTATACTAAGAAGGAAGGGGGAATGGCCGGGAGGGACAGAGACCCGCTGGTGGTTGGCAGGGTTGTGGGGGACGTGCTGGACCCCTTCATCCGGAC
||||||||||||||||||||||||||||||||||||||||||||||||||||||||||||||||||||||||||||||||||||||||||||||||||||
CGATCTATACTAAGAAGGAAGGGGGAATGGCCGGGAGGGACAGAGACCCGCTGGTGGTTGGCAGGGTTGTGGGGGACGTGCTGGACCCCTTCATCCGGAC
CACCAACCTCAGGGTGACCTTCGGGAACAGGACCGTGTCCAACGGCTGCGAGCTCAAGCCGTCCATGGTCGCCCAGCAGCCCAGGGTTGAGGTGGGCGGC
||||||||||||||||||||||||||||||||||||||||||||||||||||||||||||||||||||||||||||||||||||||||||||||||||||
CACCAACCTCAGGGTGACCTTCGGGAACAGGACCGTGTCCAACGGCTGCGAGCTCAAGCCGTCCATGGTCGCCCAGCAGCCCAGGGTTGAGGTGGGCGGC
AATGAGATGAGGACCTTCTACACACTCGTACGTACACAGTCACTATCTAATGCCTATATGTTTAGCTCTGAAAGTGCTCGCCACACGCACATGATCGATC
||||||||||||||||||||||||||||||||||||||||||||||||||||||||||||||||||||||||||||||||||||||||||||||||||||
AATGAGATGAGGACCTTCTACACACTCGTACGTACACAGTCACTATCTAATGCCTATATGTTTAGCTCTGAAAGTGCTCGCCACACGCACATGATCGATC
GAGCTCTATATATATAGTACGTGTGGGAAGATGATTCTCGATGCTTCTGTTCACAGAATGTTTGTCTTGGCAGGCACATGACTAATGCTCCATCTTGCAT
||||||||||||||||||||||||||||||||||||||||||||||||||||||||||||||||||||||||||||||||||||||||||||||||||||
GAGCTCTATATATATAGTACGTGTGGGAAGATGATTCTCGATGCTTCTGTTCACAGAATGTTTGTCTTGGCAGGCACATGACTAATGCTCCATCTTGCAT
ATGGCTCTGTGCTAGCTCTCTGGTGTTGATCATGATTTTCTATGCTTGTTTTCTTTTCTTTTCGGGGAACATTGATTTTCGATGCTTCTATTCACATGTT
||||||||||||||||||||||||||||||||||||||||||||||||||||||||||||||||||||||||||||||||||||||||||||||||||||
ATGGCTCTGTGCTAGCTCTCTGGTGTTGATCATGATTTTCTATGCTTGTTTTCTTTTCTTTTCGGGGAACATTGATTTTCGATGCTTCTATTCACATGTT
TCGTTCATGTTTGTCCTAGCAAGCACACGACTAATTAAAGCTCGGTCTTAAATACATATGCTTATTTACGGTAGTACTCTCTACTTCTCTAGTATTGATC
||||||||||||||||||||||||||||||||||||||||||||||||||||||||||||||||||||||||||||||||||||||||||||||||||||
TCGTTCATGTTTGTCCTAGCAAGCACACGACTAATTAAAGCTCGGTCTTAAATACATATGCTTATTTACGGTAGTACTCTCTACTTCTCTAGTATTGATC
ATGATGTGCACGCGTGTACTGCCCGCAGGTGATGGTAGACCCAGATGCTCCAAGTCCAAGCGATCCCAACCTTAGGGAGTATCTCCACTGGTAAGTACTA
||||||||||||||||||||||||||||||||||||||||||||||||||||||||||||||||||||||||||||||||||||||||||||||||||||
ATGATGTGCACGCGTGTACTGCCCGCAGGTGATGGTAGACCCAGATGCTCCAAGTCCAAGCGATCCCAACCTTAGGGAGTATCTCCACTGGTAAGTACTA
AATTTGTAACTCAGTTGAATAATTTCTCTGTCCCTAGATATACACAGTAGCTCATGTGTGTGTGTCTACATGTGTGTGCAGGCTTGTGACAGATATCCCC
||||||||||||||||||||||||||||||||||||||||||||||||||||||||||||||||||||||||||||||||||||||||||||||||||||
AATTTGTAACTCAGTTGAATAATTTCTCTGTCCCTAGATATACACAGTAGCTCATGTGTGTGTGTCTACATGTGTGTGCAGGCTTGTGACAGATATCCCC
GGTACAACTGGTGCATCCTTCGGGCAGGAGGTGATGTGCTACGAGAGCCCTCGTCCGACCATGGGGATCCATCGCTTCGTGCTCGTGCTCTTCCAGCAGC
|||||||||||||||||||||||||||||||||||||||||||||||||||||||||||||| |||||||||||||||||||||||||||||||||||||
GGTACAACTGGTGCATCCTTCGGGCAGGAGGTGATGTGCTACGAGAGCCCTCGTCCGACCAT-GGGATCCATCGCTTCGTGCTCGTGCTCTTCCAGCAGC
TCGGCCGGCAGACCGTGTACGCTCCCGGGTGGCGCCAGAACTTCAACACCAGGGACTTCGCCGAGCTCTACAACCTCGGCCCGCCTGTCGCCGCCGTCTA
||||||||||||||||||||||||||||||||||||||||||||||||||||||||||||||||||||||||||||||||||||||||||||||||||||
TCGGCCGGCAGACCGTGTACGCTCCCGGGTGGCGCCAGAACTTCAACACCAGGGACTTCGCCGAGCTCTACAACCTCGGCCCGCCTGTCGCCGCCGTCTA
CTTCAACTGCCAGCGTGAGGCCGGCTCCGGCGGCAGGAGGATGTACAATTGATCTACCCATGGCCCTCGTACGCCACCCGCCGCCAAGTCAGCAAATTAT
||||||||||||||||||||||||||||||||||||||||||||||||||||||||||||||||||||||||||||||||||||||||||||||||||||
CTTCAACTGCCAGCGTGAGGCCGGCTCCGGCGGCAGGAGGATGTACAATTGATCTACCCATGGCCCTCGTACGCCACCCGCCGCCAAGTCAGCAAATTAT
CCAACGTGGCTAGTTTACTAGTATATAGTTTGTCATAAGAAGCCAGCCACGAATTAATTAAGCAGTATCTCTATATTGGCAACACATACACTACATATAT
||||||||||||||||||||||||||||||||||||||||||||||||||||||||||||||||||||||||||||||||||||||||||||||||||||
CCAACGTGGCTAGTTTACTAGTATATAGTTTGTCATAAGAAGCCAGCCACGAATTAATTAAGCAGTATCTCTATATTGGCAACACATACACTACATATAT
GCATACTATATGATCGATGTATAACTAGCCGCATGCATATATGCAATCAACGGCTAAGTAAGGGGGGATGAAACCCTAGATCAATGGCTTGGTACTGCAC
||||||||||||||||||||||||||||||||||||||||||||||||||||||||||||||||||||||||||||||||||||||||||||||||||||
GCATACTATATGATCGATGTATAACTAGCCGCATGCATATATGCAATCAACGGCTAAGTAAGGGGGGATGAAACCCTAGATCAATGGCTTGGTACTGCAC
TATATATGTAGTCTGCAATAAACTGATGCCAATAGTATACAGCACACAATATTGGAGGAGCTACACGCCATGTGCAACTTAGTGCTATCTGGTACATATC
||||||||||||||||||||||||||||||||||||||||||||||||||||||||||||||||||||||||||||||||||||||||||||||||||||
TATATATGTAGTCTGCAATAAACTGATGCCAATAGTATACAGCACACAATATTGGAGGAGCTACACGCCATGTGCAACTTAGTGCTATCTGGTACATATC
TGCAGGTTGGTCTTGTGTGTTCACTTATGCGTGCATGAACATCAGTCAACATATACACATAGTTATGCATGGGAGACAAACATGTAATTGACAGCGACTG
||||||||||||||||||||||||||||||||||||||||||||||||||||||||||||||||||||||||||||||||||||||||||||||||||||
TGCAGGTTGGTCTTGTGTGTTCACTTATGCGTGCATGAACATCAGTCAACATATACACATAGTTATGCATGGGAGACAAACATGTAATTGACAGCGACTG
CTACAAGACACGGTGATATGTACACACTTAGCGCAGTAGCAAAGCACATGCATATGTTGGGTCTTGTACAACGCCCTCATGATTATGGCAATATAATCAA
||||||||||||||||||||||||||||||||||||||||||||||||||||||||||||||||||||||||||||||||||||||||||||||||||||
CTACAAGACACGGTGATATGTACACACTTAGCGCAGTAGCAAAGCACATGCATATGTTGGGTCTTGTACAACGCCCTCATGATTATGGCAATATAATCAA
GTGTCAAGTTGTGTATGAGACCGGTGGCTTCTCTGTGATGGTGAACAATATTAATCTCTGGTGCGCTCATCAATCTGTGTTTTATACTAGATGCTAGCTA
||||||||||||||||||||||||||||||||||||||||||||||||||||||||||||||||||||||||||||||||||||||||||||||||||||
GTGTCAAGTTGTGTATGAGACCGGTGGCTTCTCTGTGATGGTGAACAATATTAATCTCTGGTGCGCTCATCAATCTGTGTTTTATACTAGATGCTAGCTA
GGTAGCGTCTCTTTCAGTTGAGTTCCACGGTCTGCCGTCCTCGGTTTCAGAAATATATTCGTAGTATCGTGCTTCAGAAATATCTTTCAGAAGTTTCTTG
||||||||||||||||||||||||||||||||||||||||||||||||||||||||||||||||||||||||||||||||||||||||||||||||||||
GGTAGCGTCTCTTTCAGTTGAGTTCCACGGTCTGCCGTCCTCGGTTTCAGAAATATATTCGTAGTATCGTGCTTCAGAAATATCTTTCAGAAGTTTCTTG
CTTCCCAGCACCCAAAGTGCGTATAATGATCGGTTCATCATCAGCACTTTGCGCCTTTCGAACTTCAAAGTGAGCATGCATATATCGACAACCTTTTTAA
||||||||||||||||||||||||||||||||||||||||||||||||||||||||||||||||||||||||||||||||||||||||||||||||||||
CTTCCCAGCACCCAAAGTGCGTATAATGATCGGTTCATCATCAGCACTTTGCGCCTTTCGAACTTCAAAGTGAGCATGCATATATCGACAACCTTTTTAA
CTCTTTCCAGGCCTGGGCTTGGTTCGACTTTGTCAGCTCTTGTCATTCAGCTTCCTCTCTCCTCTCTCATCTTGTCTTTCAGTAGATGGCATTAAGAAGT
||||||||||||||||||||||||||||||||||||||||||||||||||||||||||||||||||||||||||||||||||||||||||||||||||||
CTCTTTCCAGGCCTGGGCTTGGTTCGACTTTGTCAGCTCTTGTCATTCAGCTTCCTCTCTCCTCTCTCATCTTGTCTTTCAGTAGATGGCATTAAGAAGT
AGGGCAGCAAGTAGTACTCCCTCCATCCCATAATGTAGGACGTTTTTAAGTGTCATAAAAACGTCCTACATTATGGGACGGAGGGAGTAGTTCCGATCCT
||||||||||||||||||||||||||||||||||||||||||||||||||||||||||||||||||||||||||||||||||||||||||||||||||||
AGGGCAGCAAGTAGTACTCCCTCCATCCCATAATGTAGGACGTTTTTAAGTGTCATAAAAACGTCCTACATTATGGGACGGAGGGAGTAGTTCCGATCCT
TGTGTTCTATTCTACTACAGTATTATATGAAGTCAATGGAAAAAAATCTACACACGTAAAGATGTTATTATTCCTGGGTAAGGAGAGGTAGCAAGAGCAC
||||||||||||||||||||||||||||||||||||||||||||||||||||||||||||||||||||||||||||||||||||||||||||||||||||
TGTGTTCTATTCTACTACAGTATTATATGAAGTCAATGGAAAAAAATCTACACACGTAAAGATGTTATTATTCCTGGGTAAGGAGAGGTAGCAAGAGCAC
CAACCTTTGCCGTGCGATTGGAGGGCGCAGCAGCTGCCTTTTTTCACCGGCCTAGACGCGAGATTCACCATGTGCAAAAATTATCGTAGCAGTGGGTAAA
||||||||||||||||||||||||||||||||||||||||||||||||||||||||||||||||||||||||||||||||||||||||||||||||||||
CAACCTTTGCCGTGCGATTGGAGGGCGCAGCAGCTGCCTTTTTTCACCGGCCTAGACGCGAGATTCACCATGTGCAAAAATTATCGTAGCAGTGGGTAAA
GTTGCTTTACTGATGTACCACCACT
|||||||||||||||||||||||||
GTTGCTTTACTGATGTACCACCACT

***FT-A2 - TRIAE_CS42_3AS_TGACv1_211205_AA0686640***

ATGCCGTGCCGTGCCCCTAACCTATACTACCATATATCCAATCTAAATTCAGATTTATTGCCTCAGGCAGATATTTGTATACAGTTGACGGGGCCAAATA
|||||||||||||||||||||||||||||||||||||||||| |||||||||||||||||||||||||||||||||||||||||||||||||||||||||
ATGCCGTGCCGTGCCCCTAACCTATACTACCATATATCCAATGTAAATTCAGATTTATTGCCTCAGGCAGATATTTGTATACAGTTGACGGGGCCAAATA
GGATCTTTCCGCCAAAAAGGCCTCCGATCATTCGATGGGATTAATCAGGGATAAAACGATAGCTCACTCTCTCACGTGGTGTGGTGGAGGCATGGGTGAG
||||||||||||||||||||||||||||||||||||||||||||||||||||||||||||||||||||||||||||||||||||||||||||||||||||
GGATCTTTCCGCCAAAAAGGCCTCCGATCATTCGATGGGATTAATCAGGGATAAAACGATAGCTCACTCTCTCACGTGGTGTGGTGGAGGCATGGGTGAG
TTCAATCAAATTCTTGACTTCTTGTGCACCCGACCTCCAAGTACATGCTAGGGCCCTAGCGGATTCCAAAACCAAAGATAAAAGTAAATAAACGAGAAGG
||||||||||||||||||||||||||||||||||||||||||||||||||||||||||||||||||||||||||||||||||||||||||||||||||||
TTCAATCAAATTCTTGACTTCTTGTGCACCCGACCTCCAAGTACATGCTAGGGCCCTAGCGGATTCCAAAACCAAAGATAAAAGTAAATAAACGAGAAGG
AATGGCTTCTTCTTGATTTTATGCGCGGAGTGGCTAAATAAGATTAGATTCTTATTGAGTTATGTGTATATGATAATAGATTGGCATACATAGTTTAGGG
||||||||||||||||||||||||||||||||||||||||||||||||||||||||||||||||||||||||||||||||||||||||||||||||||||
AATGGCTTCTTCTTGATTTTATGCGCGGAGTGGCTAAATAAGATTAGATTCTTATTGAGTTATGTGTATATGATAATAGATTGGCATACATAGTTTAGGG
GAATACGCATTTAGACGCACAATAATACAATTAAGCAACTAATGTGCCTATAAAAGAGTTCCACTATGTTTTTAAATCATCTCACCAGTTAGTAACTTCG
||||||||||||||||||||||||||||||||||||||||||||||||||||||||||||||||||||||||||||||||||||||||||||||||||||
GAATACGCATTTAGACGCACAATAATACAATTAAGCAACTAATGTGCCTATAAAAGAGTTCCACTATGTTTTTAAATCATCTCACCAGTTAGTAACTTCG
AAATTATCTTATATAGTCCTATATATGGAAAAGAAAGCATGTTATCTCGGCCCATGCATGGGGGGCGCATGTACTTAGGAGAAGGAAAAATTATAAAGAA
||||||||||||||||||||||||||||||||||||||||||||||||||||||||||||||||||||||||||||||||||||||||||||||||||||
AAATTATCTTATATAGTCCTATATATGGAAAAGAAAGCATGTTATCTCGGCCCATGCATGGGGGGCGCATGTACTTAGGAGAAGGAAAAATTATAAAGAA
AGAAACACCAACATTTGCAGCACACACACACACACACACACACATACAGCTATTCTAATGCACAAAGGTATTAAACTAAAGCTAAAAGTACGAAAATTCA
||||||||||||||||||||||||||||||||||||||||||||||||||||||||||||||||||||||||||||||||||||||||||||||||||||
AGAAACACCAACATTTGCAGCACACACACACACACACACACACATACAGCTATTCTAATGCACAAAGGTATTAAACTAAAGCTAAAAGTACGAAAATTCA
AGATATACCAAATTACTGCTCCCTCTGTTCCATATTAGTTGTCACTGGTTTAGTATAAAGCTAAAAGTACTACTCCCTCCATTCCTAGATATAGGGTGTA
||||||||||||||||||||||||||||||||||||||||||||||||||||||||||||||||||||||||||||||||||||||||||||||||||||
AGATATACCAAATTACTGCTCCCTCTGTTCCATATTAGTTGTCACTGGTTTAGTATAAAGCTAAAAGTACTACTCCCTCCATTCCTAGATATAGGGTGTA
TAGTTTTTGGCACAAAAATTTAAAAACCCGTGTGGAGGGAAAATTTCACAAGTTTTGGGCAAGATTAAACTTGACTAATTGACATGAGGAAATAGAGGAG
||||||||||||||||||||||||||||||||||||||||||||||||||||||||||||||||||||||||||||||||||||||||||||||||||||
TAGTTTTTGGCACAAAAATTTAAAAACCCGTGTGGAGGGAAAATTTCACAAGTTTTGGGCAAGATTAAACTTGACTAATTGACATGAGGAAATAGAGGAG
CTTGCCCGATATAAGGAACTGTAATCAAATTCCTAAAAAATATATCCAAACGAGTGGTGCAATGCAATACACCTTATACTTTCGGACTTTTTCTCAAAAA
||||||||||||||||||||||||||||||||||||||||||||||||||||||||||||||||||||||||||||||||||||||||||||||||||||
CTTGCCCGATATAAGGAACTGTAATCAAATTCCTAAAAAATATATCCAAACGAGTGGTGCAATGCAATACACCTTATACTTTCGGACTTTTTCTCAAAAA
TCTATACACCTTACATCAAGGAACGGGGGGAGTAATATGGATGGAGGGCGTATGAAATAGGAAACTTGTTTGCATTGTACAATTAGAATCAAACCGGAAT
||||||||||||||||||||||||||||||||||||||||||||||||||||||||||||||||||||||||||||||||||||||||||||||||||||
TCTATACACCTTACATCAAGGAACGGGGGGAGTAATATGGATGGAGGGCGTATGAAATAGGAAACTTGTTTGCATTGTACAATTAGAATCAAACCGGAAT
AACCGGGACAATTATATCTTTGCTAATTAGGGTTATCACATTATGGGTTTATATTAGCTTATGTTAAACATAAAATAGGCATATGCAACAATTAAACTAG
||||||||||||||||||||||||||||||||||||||||||||||||||||||||||||||||||||||||||||||||||||||||||||||||||||
AACCGGGACAATTATATCTTTGCTAATTAGGGTTATCACATTATGGGTTTATATTAGCTTATGTTAAACATAAAATAGGCATATGCAACAATTAAACTAG
AACAAAGGTAATTACACCATAAATGGGATTAAAAAAATCAATGCTAAGTTAACTAGAACTTCTAGTGAAGTAAAGCAAGCGAAAAGCATAAATTTAAGGA
||||||||||||||||||||||||||||||||||||||||||||||||||||||||||||||||||||||||||||||||||||||||||||||||||||
AACAAAGGTAATTACACCATAAATGGGATTAAAAAAATCAATGCTAAGTTAACTAGAACTTCTAGTGAAGTAAAGCAAGCGAAAAGCATAAATTTAAGGA
AAAATGAAAGGTTAGGATTGCTAGAGGTAGCAAATTGTTTTTTGTTCTATGGGAAGATGATATGTCTCATCAGTCCGTTACTAATAGAACAAGAATGACA
||||||||||||||||||||||||||||||||||||||||||||||||||||||||||||||||||||||||||||||||||||||||||||||||||||
AAAATGAAAGGTTAGGATTGCTAGAGGTAGCAAATTGTTTTTTGTTCTATGGGAAGATGATATGTCTCATCAGTCCGTTACTAATAGAACAAGAATGACA
TTTTACGAGTTTGCATTAAATTATATGCTCTAGTTCGCACATTATTAACTAGACTTGTAATGAAATGTATATTTTTAATAAAAAAACTATATCCTTTGAT
||||||||||||||||||||||||||||||||||||||||||||||||||||||||||||||||||||||||||||||||||||||||||||||||||||
TTTTACGAGTTTGCATTAAATTATATGCTCTAGTTCGCACATTATTAACTAGACTTGTAATGAAATGTATATTTTTAATAAAAAAACTATATCCTTTGAT
AAGATGTATCCTACATATATTTAGCTAAAGATTTAAGGGGAGGGGAGGGGGGGGGGGGGTCCTTATTCATTGTTTTGAGTTTGAACTATATATACCGTAC
|||||||||||||||||||||||||||||||||||||||||||||||||||||||| |||||||||||||||||||||||||||||||||||||||||
AAGATGTATCCTACATATATTTAGCTAAAGATTTAAGGGGAGGGGAGGGGGGGGGG---TCCTTATTCATTGTTTTGAGTTTGAACTATATATACCGTAC
AAGGCTACAAAATAATTGCTTCGCTTTTGCAAGTTTTAGAAGAGAAGAAAAATTCTCACAAGGTCTTACAACATTAAGCCAAACAAAATAAAGAATCGGT
||||||||||||||||||||||||||||||||||||||||||||||||||||||||||||||||||||||||||||||||||||||||||||||||||||
AAGGCTACAAAATAATTGCTTCGCTTTTGCAAGTTTTAGAAGAGAAGAAAAATTCTCACAAGGTCTTACAACATTAAGCCAAACAAAATAAAGAATCGGT
TGAGGACATTAATGTTATCTCTTATGCAATAGTATTTTCGTGGTGTGATCAGGAAACAATGCATAAGTGGAAGAAAAAAAAAGAGTCATCATCTAAAAAA
||||||||||||||||||||||||||||||||||||||||||||||||||||||||||||||||||||||||||||||||||||||||||||||||||||
TGAGGACATTAATGTTATCTCTTATGCAATAGTATTTTCGTGGTGTGATCAGGAAACAATGCATAAGTGGAAGAAAAAAAAAGAGTCATCATCTAAAAAA
ACTAATATATAGTGCAACATGCCAATAAATGCATATCTCATGCTTTATTCAGTTTTTATTTTTCAAATAAAACTTGTGCAGCACAAAAATTGGCTTCGGC
||||||||||||||||||||||||||||||||||||||||||||||||||||||||||||||||||||||||||||||||||||||||||||||||||||
ACTAATATATAGTGCAACATGCCAATAAATGCATATCTCATGCTTTATTCAGTTTTTATTTTTCAAATAAAACTTGTGCAGCACAAAAATTGGCTTCGGC
GAGAAATATTTATTTTAACACCATCAATTACTTAATTCCTGTGACGCGATATAAAAATCTAGAACACCGGCATGGAAGGCAATGTTTGCTTTGCACTTAT
||||||||||||||||||||||||||||||||||||||||||||||||||||||||||||||||||||||||||||||||||||||||||||||||||||
GAGAAATATTTATTTTAACACCATCAATTACTTAATTCCTGTGACGCGATATAAAAATCTAGAACACCGGCATGGAAGGCAATGTTTGCTTTGCACTTAT
ATTGAGGCAGGGGGCGCCAAATTTGTATCCCTCTTTACATGCCCATGCTGGGGCTGCTTGACAACCAATTCGCAAATACCTGGATGGCCTACTCATAGCC
||||||||||||||||||||||||||||||||||||||||||||||||||||||||||||||||||||||||||||||||||||||||||||||||||||
ATTGAGGCAGGGGGCGCCAAATTTGTATCCCTCTTTACATGCCCATGCTGGGGCTGCTTGACAACCAATTCGCAAATACCTGGATGGCCTACTCATAGCC
TAAAAGGGTCACCCCAAAGGTCCAAACTGCAGAGGTTTCCTGAGACCAGAATCTCCTGCACAAACTTAATCATCAATTTCACTGAAGACTACTCATCTGC
||||||||||||||||||||||||||||||||||||||||||||||||||||||||||||||||||||||||||||||||||||||||||||||||||||
TAAAAGGGTCACCCCAAAGGTCCAAACTGCAGAGGTTTCCTGAGACCAGAATCTCCTGCACAAACTTAATCATCAATTTCACTGAAGACTACTCATCTGC
TCCATCTGTAAAGAAATATAAAAACGTTTAGATCATTATTTTATTATATTTCTTTACGGAGGGAGTGCTTTTTTGTAATTAAGTGCTGAACCAATATTAA
||||||||||||||||||||||||||||||||||||||||||||||||||||||||||||||||||||||||||||||||||||||||||||||||||||
TCCATCTGTAAAGAAATATAAAAACGTTTAGATCATTATTTTATTATATTTCTTTACGGAGGGAGTGCTTTTTTGTAATTAAGTGCTGAACCAATATTAA
TGTGATCTACCGATGCAGGCTGGTCACCGACATCCCGGGGACGACAGGAGTATCTTTCGGTACAGGAGAAGAAAACTGATCTCACGTCTCACCACTTTAT
||||||||||||||||||||||||||||||||||||||||||||||||||||||||||||||||||||||||||||||||||||||||||||||||||||
TGTGATCTACCGATGCAGGCTGGTCACCGACATCCCGGGGACGACAGGAGTATCTTTCGGTACAGGAGAAGAAAACTGATCTCACGTCTCACCACTTTAT
TATACAGAACTCAGCGTCGCTCTGGGCTCTAAATCTAATGGAACTTTTCGTACGGATGCATGTCCAGGGACCGAGGTGGTGTGCTACGAGGGCCCGCGGC
||||||||||||||||||||||||||||||||||||||||||||||||||||||||||||||||||||||||||||||||||||||||||||||||||||
TATACAGAACTCAGCGTCGCTCTGGGCTCTAAATCTAATGGAACTTTTCGTACGGATGCATGTCCAGGGACCGAGGTGGTGTGCTACGAGGGCCCGCGGC
CGGTGCTCGGGATCCACCGGCTGGTGTTCCTGCTCTTCCAGCAGCTGGGCCGCCAGACGGTGTACGCGCCGGGGTGGCGGCAGAACTTCAGCACCCGCGA
||||||||||||||||||||||||||||||||||||||||||||||||||||||||||||||||||||||||||||||||||||||||||||||||||||
CGGTGCTCGGGATCCACCGGCTGGTGTTCCTGCTCTTCCAGCAGCTGGGCCGCCAGACGGTGTACGCGCCGGGGTGGCGGCAGAACTTCAGCACCCGCGA
CTTTGCCGAGCTCTACAACCTCGGCCTGCCCGTCGCCGCCGTCTACTTCAACTGCCAGAGGGAGACCGGAACCGGCGGGAGGAGGATGTGATCATGATCA
||||||||||||||||||||||||||||||||||||||||||||||||||||||||||||||||||||||||||||||||||||||||||||||||||||
CTTTGCCGAGCTCTACAACCTCGGCCTGCCCGTCGCCGCCGTCTACTTCAACTGCCAGAGGGAGACCGGAACCGGCGGGAGGAGGATGTGATCATGATCA
CCAGCTCCTTGTAGTACGATACTAGTAGTATATGTGACCACAGGATATGACGATGATGATGATGGTGGTGAGGATGAAGTCGATCGTATGGGTGGTTTGA
||||||||||||||||||||||||||||||||||||||||||||||||||||||||||||||||||||||||||||||||||||||||||||||||||||
CCAGCTCCTTGTAGTACGATACTAGTAGTATATGTGACCACAGGATATGACGATGATGATGATGGTGGTGAGGATGAAGTCGATCGTATGGGTGGTTTGA
TTATACAGGGCGAAATGGAGAAAGCATTTGTAATGTTCGAGATAATAACTATGCGTGCGACATTTTTGGTCCGATGCTCGTGCATGCTACGTACTTCTGA
||||||||||||||||||||||||||||||||||||||||||||||||||||||||||||||||||||||||||||||||||||||||||||||||||||
TTATACAGGGCGAAATGGAGAAAGCATTTGTAATGTTCGAGATAATAACTATGCGTGCGACATTTTTGGTCCGATGCTCGTGCATGCTACGTACTTCTGA
ACCACACAGATTGAAAAGATGTTGGGATCCAACGGGCCAGCCGGGAGATCGTACGGAAATCAGATGGAAATTGTCGTGCGGGTAGCGTCGCTGTATTTGT
||||||||||||||||||||||||||||||||||||||||||||||||||||||||||||||||||||||||||||||||||||||||||||||||||||
ACCACACAGATTGAAAAGATGTTGGGATCCAACGGGCCAGCCGGGAGATCGTACGGAAATCAGATGGAAATTGTCGTGCGGGTAGCGTCGCTGTATTTGT
AATGTATATGTGTGAACTGTCTTTATACTTTGTAGAAATGTATTATTGGGTGCTTGTGGTTGTTCATTTGCCACCCCACCTCCTCTCTACCGGAAGTAAT
||||||||||||||||||||||||||||||||||||||||||||||||||||||||||||||||||||||||||||||||||||||||||||||||||||
AATGTATATGTGTGAACTGTCTTTATACTTTGTAGAAATGTATTATTGGGTGCTTGTGGTTGTTCATTTGCCACCCCACCTCCTCTCTACCGGAAGTAAT
TTTCTTACAAGACGGCTATCTCTTCTGGTCCCAATATTGTTTTCACGTCTTAAGAAATGTAACAGGTTATTTCTACGGCTTTCTATTTTGTGTTTAGCAC
||||||||||||||||||||||||||||||||||||||||||||||||||||||||||||||||||||||||||||||||||||||||||||||||||||
TTTCTTACAAGACGGCTATCTCTTCTGGTCCCAATATTGTTTTCACGTCTTAAGAAATGTAACAGGTTATTTCTACGGCTTTCTATTTTGTGTTTAGCAC
AAGTAGTTTTGTGTAATCATTTGTTCTGAAATAAGCCAACATAATGAAGAATAAGAATAAACAAGTACTATTTGTTGAATCCAAGACACGATCTCAATAC
||||||||||||||||||||||||||||||||||||||||||||||||||||||||||||||||||||||||||||||||||||||||||||||||||||
AAGTAGTTTTGTGTAATCATTTGTTCTGAAATAAGCCAACATAATGAAGAATAAGAATAAACAAGTACTATTTGTTGAATCCAAGACACGATCTCAATAC
TCTAGAACCGCGAATAAAGACATCATTTTGCATTTGTTTTCTTATCTTTGTAAAACGTTCTTGTAGCAACAACTTGCATCTCGCGTGTTCCTTTAACAAG
||||||||||||||||||||||||||||||||||||||||||||||||||||||||||||||||||||||||||||||||||||||||||||||||||||
TCTAGAACCGCGAATAAAGACATCATTTTGCATTTGTTTTCTTATCTTTGTAAAACGTTCTTGTAGCAACAACTTGCATCTCGCGTGTTCCTTTAACAAG
GAAAGACATTCAAATATGATTCAATGGTTGTGAAAACACATGTATCTATCATTTATATTGTTGGAAGCAGATAACAAACATTGTTCATGAGGCACGATTG
||||||||||||||||||||||||||||||||||||||||||||||||||||||||||||||||||||||||||||||||||||||||||||||||||||
GAAAGACATTCAAATATGATTCAATGGTTGTGAAAACACATGTATCTATCATTTATATTGTTGGAAGCAGATAACAAACATTGTTCATGAGGCACGATTG
CGGCCGAGGAAGAGGGCTACTCAAGAACCTAGAATACAAATATCTATTTTTTTCATGTTTCTTCTTGTAGATCAAAGCTTTGTGTAGTCTAAACGTTACT
||||||||||||||||||||||||||||||||||||||||||||||||||||||||||||||||||||||||||||||||||||||||||||||||||||
CGGCCGAGGAAGAGGGCTACTCAAGAACCTAGAATACAAATATCTATTTTTTTCATGTTTCTTCTTGTAGATCAAAGCTTTGTGTAGTCTAAACGTTACT
TTCCTGTTCTCCCTCTCCCAAGGCCCTAGATAGCCATCACCGCTCCCTGCCAGCAAGGCCATAGATCCACTCCTCCTCCACCGGCTGCTCCGATGTAGGC
||||||||||||||||||||||||||||||||||||||||||||||||||||||||||||||||||||||||||||||||||||||||||||||||||||
TTCCTGTTCTCCCTCTCCCAAGGCCCTAGATAGCCATCACCGCTCCCTGCCAGCAAGGCCATAGATCCACTCCTCCTCCACCGGCTGCTCCGATGTAGGC
ACTGCGGTGGTGATGGTGGCACCATGCAGATTAAGAATAAAAGGTTTTCTTGACTCCTGGTCCGCCTCGTCAGCGGGGGTCTCGACAACGAAGTAAAGGA
||||||||||||||||||||||||||||||||||||||||||||||||||||||||||||||||||||||||||||||||||||||||||||||||||||
ACTGCGGTGGTGATGGTGGCACCATGCAGATTAAGAATAAAAGGTTTTCTTGACTCCTGGTCCGCCTCGTCAGCGGGGGTCTCGACAACGAAGTAAAGGA
GGGCGGCAAATTGTATGTTACTACTCTTTTGGAGTGGTGGATCTGGTTATGTCTTCATGTTATGCTTTTAGCAGAGTGAGTGCATTGTGGCGCTTCGTGT
||||||||||||||||||||||||||||||||||||||||||||||||||||||||||||||||||||||||||||||||||||||||||||||||||||
GGGCGGCAAATTGTATGTTACTACTCTTTTGGAGTGGTGGATCTGGTTATGTCTTCATGTTATGCTTTTAGCAGAGTGAGTGCATTGTGGCGCTTCGTGT
CTTGGTCCTATGGCTCCTCCAACCGACGATGT
||||||||||||||||||||||||||||||||
CTTGGTCCTATGGCTCCTCCAACCGACGATGT

***FT-B2 - TRIAE_CS42_3B_TGACv1_223754_AA0786660***

GCGTTGGGGAGGGGTGACAAGCCGAAAAAGGACTGACCGAACCGAGACGTGGTGTATTACCCCACTCAGAATTTAAACCGCAATAAAAGATTATTTCTAT
||||||||||||||||||||||||||||||||||||||||||||||||||||||||||||||||||||||||||||||||||||||||||||||||||||
GCGTTGGGGAGGGGTGACAAGCCGAAAAAGGACTGACCGAACCGAGACGTGGTGTATTACCCCACTCAGAATTTAAACCGCAATAAAAGATTATTTCTAT
TCTTTTGTAAAAGAAAACTTACTTGCATATCACTTGAATTTACTTGAAAAGAAGCGACTCCACTCAAATTCGTTCCTCCACCTAAATTTTCCTCTTGGTC
||||||||||||||||||||||||||||||||||||||||||||||||||||||||||||||||||||||||||||||||||||||||||||||||||||
TCTTTTGTAAAAGAAAACTTACTTGCATATCACTTGAATTTACTTGAAAAGAAGCGACTCCACTCAAATTCGTTCCTCCACCTAAATTTTCCTCTTGGTC
TACCCCGCACCCAAGCTCATTAATTTCTTCTTCTTCTTTTTCTGCGAATCTCATTAATTTCGCAGTAATACAACTGTTAAGAGCTCAACCGTGTGCTAGC
||||||||||||||||||||||||||||||||||||||||||||||||||||||||||||||||||||||||||||||||||||||||||||||||||||
TACCCCGCACCCAAGCTCATTAATTTCTTCTTCTTCTTTTTCTGCGAATCTCATTAATTTCGCAGTAATACAACTGTTAAGAGCTCAACCGTGTGCTAGC
TAATCAATTTAGTTTGTGGTGCGAACTGAGCGTAGTCCTGATGGTTAGGTTTCTTGTGGGATCGATACCTCGGAGGTGTTTATAGAGATAAGGTGTGCGT
||||||||||||||||||||||||||||||||||||||||||||||||||||||||||||||||||||||||||||||||||||||||||||||||||||
TAATCAATTTAGTTTGTGGTGCGAACTGAGCGTAGTCCTGATGGTTAGGTTTCTTGTGGGATCGATACCTCGGAGGTGTTTATAGAGATAAGGTGTGCGT
GCATATGTTTTTAGGGATGAGTGCACAATTGTGTTGTATTAACAAAATTTTAGTTTGAGAGAACACATTTTTTTTATAAAAAGCATCTTTATTAACTCAA
||||||||||||||||||||||||||||||||||||||||||||||||||||||||||||||||||||||||||||||||||||||||||||||||||||
GCATATGTTTTTAGGGATGAGTGCACAATTGTGTTGTATTAACAAAATTTTAGTTTGAGAGAACACATTTTTTTTATAAAAAGCATCTTTATTAACTCAA
TATGTAGTATCGAGTCGATACAAAGCATAGAGAACCCATAGAAAACATCGAGGTGATATAGACTGTTGTATATTCGCTCTCGGCCACTCTCATGCGCCGC
||||||||||||||||||||||||||||||||||||||||||||||||||||||||||||||||||||||||||||||||||||||||||||||||||||
TATGTAGTATCGAGTCGATACAAAGCATAGAGAACCCATAGAAAACATCGAGGTGATATAGACTGTTGTATATTCGCTCTCGGCCACTCTCATGCGCCGC
ACGTACCGCCAGCACGGGCACTTCTCCTTTCCTAGACGGGCAATAGGCCATTTGACCCACCCACTCCCCCTTTCCCAAACGCGACTCTTCCTCCTCCTCG
||||||||||||||||||||||||||||||||||||||||||||||||||||||||||||||||||||||||||||||||||||||||||||||||||||
ACGTACCGCCAGCACGGGCACTTCTCCTTTCCTAGACGGGCAATAGGCCATTTGACCCACCCACTCCCCCTTTCCCAAACGCGACTCTTCCTCCTCCTCG
CCATAGGACTCTAGGAGGCAGAGGAATGGACTTGGTGGCCGGCGATCCATAGATTCCCGTCGACAGCAACCATCGGATCGGACCGGTCAGGGCAGCCTGA
||||||||||||||||||||||||||||||||||||||||||||||||||||||||||||||||||||||||||||||||||||||||||||||||||||
CCATAGGACTCTAGGAGGCAGAGGAATGGACTTGGTGGCCGGCGATCCATAGATTCCCGTCGACAGCAACCATCGGATCGGACCGGTCAGGGCAGCCTGA
CGCACGCAGGACACCTCGTGATCCAGCAGCTATAGCTAGCTAGAGGCGTCGCGCTGGAGGAGCGAGCGAGCTATTCCAGAGATCTCTCTCTCTCCTCTCC
||||||||||||||||||||||||||||||||||||||||||||||||||||||||||||||||||||||||||||||||||||||||||||||||||||
CGCACGCAGGACACCTCGTGATCCAGCAGCTATAGCTAGCTAGAGGCGTCGCGCTGGAGGAGCGAGCGAGCTATTCCAGAGATCTCTCTCTCTCCTCTCC
CCCCTCTTCTATAAGTACGTCAGCTCGTCGTGGTGCAGCAGCAACAACGACGGAAGCAGCAAAACCACACACACACGCAGCTAGAGCTAATAGAGCCGGC
||||||||||||||||||||||||||||||||||||||||||||||||||||||||||||||||||||||||||||||||||||||||||||||||||||
CCCCTCTTCTATAAGTACGTCAGCTCGTCGTGGTGCAGCAGCAACAACGACGGAAGCAGCAAAACCACACACACACGCAGCTAGAGCTAATAGAGCCGGC
CGCCTTCATTAACATATCGCCACTTGCGCCGGCGGCCGGCGGAGAAGGGCGCCAGTGGTGACAGGAGGAAGAAGATGGTGGGGAGCGGCATGCATGCCCA
||||||||||||||||||||||||||||||||||||||||||||||||||||||||||||||||||||||||||||||||||||||||||||||||||||
CGCCTTCATTAACATATCGCCACTTGCGCCGGCGGCCGGCGGAGAAGGGCGCCAGTGGTGACAGGAGGAAGAAGATGGTGGGGAGCGGCATGCATGCCCA
GCGCGGGGACCCGCTGGTGGTGGGGCGCGTGATCGGCGACGTGGTGGACCCGTTCGTGCGGCGGGTGGCGCTGCGGGTCGGCTACGCGTCCAGGGACGTG
||||||||||||||||||||||||||||||||||||||||||||||||||||||||||||||||||||||||||||||||||||||||||||||||||||
GCGCGGGGACCCGCTGGTGGTGGGGCGCGTGATCGGCGACGTGGTGGACCCGTTCGTGCGGCGGGTGGCGCTGCGGGTCGGCTACGCGTCCAGGGACGTG
GCCAACGGCTGCGAGCTGAGGCCGTCCGCCATCGCCGACCCGCCGCGCGTCGAGGTCGGCGGCCCGGACATGCGCACCTTCTACACGCTGGTGAGTTCCG
||||||||||||||||||||||||||||||||||||||||||||||||||||||||||||||||||||||||||||||||||||||||||||||||||||
GCCAACGGCTGCGAGCTGAGGCCGTCCGCCATCGCCGACCCGCCGCGCGTCGAGGTCGGCGGCCCGGACATGCGCACCTTCTACACGCTGGTGAGTTCCG
CCTCCGCCGTACGTACGTCTGTACGTGCCATGCTCGCGCGCTGCTTAATTACTCCTCCTAGATTACTTACTCCGGTGAGTGCGTGCGCGCAGGTGATGGT
||||||||||||||||||||||||||||||||||||||||||||||||||||||||||||||||||||||||||||||||||||||||||||||||||||
CCTCCGCCGTACGTACGTCTGTACGTGCCATGCTCGCGCGCTGCTTAATTACTCCTCCTAGATTACTTACTCCGGTGAGTGCGTGCGCGCAGGTGATGGT
GGATCCGGATGCTCCAAGTCCCAGCGATCCCAGCCTTAGGGAGTACTTGCACTGGTGAGAGCAGAGCATGCACCAACAAAACAAAACAAAACAAAACAAA
||||||||||||||||||||||||||||||||||||||||||||||||||||||||||||||||||||||||||||||||||||||||||||||||||||
GGATCCGGATGCTCCAAGTCCCAGCGATCCCAGCCTTAGGGAGTACTTGCACTGGTGAGAGCAGAGCATGCACCAACAAAACAAAACAAAACAAAACAAA
AGATTTCCCTCCTACTCCCAGCGGCTTCCCTAGCTAGCTAGCTTGCTTGCTTGCTTGGAAATATCCCCACCAACATGCCGTGCTCCTGACCTAGCACAGG
||||||||||||||||||||||||||||||||||||||||||||||||||||||||||||||||||||||||||||||||||||||||||||||||||||
AGATTTCCCTCCTACTCCCAGCGGCTTCCCTAGCTAGCTAGCTTGCTTGCTTGCTTGGAAATATCCCCACCAACATGCCGTGCTCCTGACCTAGCACAGG
CAGAGTCTCTACTAGGACTACATAAATTCAGATTTATATATTGCCGCAGGCAGATATTTGTATACAGTTGACGGGGCCAAATAGGATCTTTCAGCCGAAA
||||||||||||||||||||||||||||||||||||||||||||||||||||||||||||||||||||||||||||||||||||||||||||||||||||
CAGAGTCTCTACTAGGACTACATAAATTCAGATTTATATATTGCCGCAGGCAGATATTTGTATACAGTTGACGGGGCCAAATAGGATCTTTCAGCCGAAA
AACGCATTGAATACACTTCCAAGAGATTAACGAGGGATTGGTGGATAGCTCACTCTCTCACATAGTGTGGTGGAGACATGTATGGGTGAATGGCCCACTC
||||||||||||||||||||||||||||||||||||||||||||||||||||||||||||||||||||||||||||||||||||||||||||||||||||
AACGCATTGAATACACTTCCAAGAGATTAACGAGGGATTGGTGGATAGCTCACTCTCTCACATAGTGTGGTGGAGACATGTATGGGTGAATGGCCCACTC
ACCACCTGAGTTCAATCAAATTCTTGTGCACCCAACCTCCCCTCCAAGTACTTCATGCTAGGGCTCTAGCAGATTTCAAAACCAAAGATAAAAAGTACTC
||||||||||||||||||||||||||||||||||||||||||||||||||||||||||||||||||||||||||||||||||||||||||||||||||||
ACCACCTGAGTTCAATCAAATTCTTGTGCACCCAACCTCCCCTCCAAGTACTTCATGCTAGGGCTCTAGCAGATTTCAAAACCAAAGATAAAAAGTACTC
CCTCTGTAAATGAATATAAGAGTCCTTAGATCACTAAAGTAGTGATCTAAACGCTCTTATATCTCGTTACGGAGGGAGTAAATACTAAATGAAAAGGCAT
||||||||||||||||||||||||||||||||||||||||||||||||||||||||||||||||||||||||||||||||||||||||||||||||||||
CCTCTGTAAATGAATATAAGAGTCCTTAGATCACTAAAGTAGTGATCTAAACGCTCTTATATCTCGTTACGGAGGGAGTAAATACTAAATGAAAAGGCAT
GACTTATTGATTTTATGCGCAGAGTGACTGATTCACATATAAATAGGATAAATTTTTGTGTCTTTTTTTGTGCATGATGCTACATTGACATATACAGTTT
||||||||||||||||||||||||||||||||||||||||||||||||||||||||||||||||||||||||||||||||||||||||||||||||||||
GACTTATTGATTTTATGCGCAGAGTGACTGATTCACATATAAATAGGATAAATTTTTGTGTCTTTTTTTGTGCATGATGCTACATTGACATATACAGTTT
AGTGTTGAGGGAATGCACGTTTAGATGCCCAATAATATGATTAAGCAACTAGTGTACCTACAAAAGAGTTCAAGTGTGTTTTTAAATCATCTCATCAATC
||||||||||||||||||||||||||||||||||||||||||||||||||||||||||||||||||||||||||||||||||||||||||||||||||||
AGTGTTGAGGGAATGCACGTTTAGATGCCCAATAATATGATTAAGCAACTAGTGTACCTACAAAAGAGTTCAAGTGTGTTTTTAAATCATCTCATCAATC
AGTAGCTTCAAAATTATCTTATGTAATCCTACATATGGACATCAAAGCATATTATCTAGACCCATGCATATGCATGGGGGCGGATGTACTTAGGAGTGGA
||||||||||||||||||||||||||||||||||||||||||||||||||||||||||||||||||||||||||||||||||||||||||||||||||||
AGTAGCTTCAAAATTATCTTATGTAATCCTACATATGGACATCAAAGCATATTATCTAGACCCATGCATATGCATGGGGGCGGATGTACTTAGGAGTGGA
AAACTTTATAAAGAAATAAATACGCGTGCACGCGCACACACACAACTTTTCTAATGCAAAAAATATATTCAACAAAATTAAAAGGAAAAAAATTCAAGAC
||||||||||||||||||||||||||||||||||||||||||||||||||||||||||||||||||||||||||||||||||||||||||||||||||||
AAACTTTATAAAGAAATAAATACGCGTGCACGCGCACACACACAACTTTTCTAATGCAAAAAATATATTCAACAAAATTAAAAGGAAAAAAATTCAAGAC
ATACCAAATTACTATGAAATAGGCAAGTTGTTTGCATTGTACAACTAGAATCGAACTGAAATAGCCAGGACAATGTGTCTTTACTAATTAGCATTATCAC
||||||||||||||||||||||||||||||||||||||||||||||||||||||||||||||||||||||||||||||||||||||||||||||||||||
ATACCAAATTACTATGAAATAGGCAAGTTGTTTGCATTGTACAACTAGAATCGAACTGAAATAGCCAGGACAATGTGTCTTTACTAATTAGCATTATCAC
ATTATGGGTTTATATTTGCTTACGTTGAAAATAAAAATAGGTTCATGTGCAACAATTAAACTAGAACAAAGGTAATTGCACCATAAATGCGATTACAACT
||||||||||||||||||||||||||||||||||||||||||||||||||||||||||||||||||||||||||||||||||||||||||||||||||||
ATTATGGGTTTATATTTGCTTACGTTGAAAATAAAAATAGGTTCATGTGCAACAATTAAACTAGAACAAAGGTAATTGCACCATAAATGCGATTACAACT
TCTTGCTAAGTTTACTAGAACTTCCAGTGAAGTAAGGCAAACGAAGAACATAAATTTAGGTAAAAATGAAAGGTGGGTTTGCTAGACATAGCATATTGTT
||||||||||||||||||||||||||||||||||||||||||||||||||||||||||||||||||||||||||||||||||||||||||||||||||||
TCTTGCTAAGTTTACTAGAACTTCCAGTGAAGTAAGGCAAACGAAGAACATAAATTTAGGTAAAAATGAAAGGTGGGTTTGCTAGACATAGCATATTGTT
TTCGGTTCTATAGGAAGATACATGTACCGTTAGTCCGTTACTGATAAAAAAAGATATTTTACGGCTTTGTATTGAATTATATGCTCTAGTCTGTAGATTA
||||||||||||||||||||||||||||||||||||||||||||||||||||||||||||||||||||||||||||||||||||||||||||||||||||
TTCGGTTCTATAGGAAGATACATGTACCGTTAGTCCGTTACTGATAAAAAAAGATATTTTACGGCTTTGTATTGAATTATATGCTCTAGTCTGTAGATTA
TTAACTAGACTTGCAATGAAATGTATATTACTACTCCCTCTGTTCACTTTTGTAAGACGTTTTAGACAGCTGAAATTGAATTGTTTTAGGTGTTGTCTTA
||||||||||||||||||||||||||||||||||||||||||||||||||||||||||||||||||||||||||||||||||||||||||||||||||||
TTAACTAGACTTGCAATGAAATGTATATTACTACTCCCTCTGTTCACTTTTGTAAGACGTTTTAGACAGCTGAAATTGAATTGTTTTAGGTGTTGTCTTA
AATGTCTAAAACGTCTTAGAAAAGTGAACGGAGGGAGTAATAAAAATCTCCATCCTTGATAATATGTATTTGGCTAAAGATTTAAGGGGGGGGGGGGGGG
||||||||||||||||||||||||||||||||||||||||||||||||||||||||||||||||||||||||||||||||||||| |||||||||||||
AATGTCTAAAACGTCTTAGAAAAGTGAACGGAGGGAGTAATAAAAATCTCCATCCTTGATAATATGTATTTGGCTAAAGATTTAA--GGGGGGGGGGGGG
GGTCCTTTTGATTTGAGTTTGACCTATTTAATGTTTTAAGTTTGAACTATATGTACCATAC
|||||||||||||||||||||||||||||||||||||||||||||||||||||||||||||
GGTCCTTTTGATTTGAGTTTGACCTATTTAATGTTTTAAGTTTGAACTATATGTACCATAC

***FT-D2 - TRIAE_CS42_3DS_TGACv1_272258_AA0917880***

AATTCAAGGTCGCACGGGTGTGGGGGAGGGGTGACAAGCCGAAAAAGGACTGACCGAACCGAGACGTGGTGTATTACCCCACTCAGAATTTAAATTGCAA
||||||||||||||||||||||||||||||||||||||||||||||||||||||||||||||||||||||||||||||||||||||||||||||||||||
AATTCAAGGTCGCACGGGTGTGGGGGAGGGGTGACAAGCCGAAAAAGGACTGACCGAACCGAGACGTGGTGTATTACCCCACTCAGAATTTAAATTGCAA
TAAAAGATTATTTCTGTTCTTTTGTAAAAGAAAACTTGCTTACAGATCACTTGAATTTACTTGAAAAGAAGCGACTCCACTCAAATTCGTTCCTCCACCT
||||||||||||||||||||||||||||||||||||||||||||||||||||||||||||||||||||||||||||||||||||||||||||||||||||
TAAAAGATTATTTCTGTTCTTTTGTAAAAGAAAACTTGCTTACAGATCACTTGAATTTACTTGAAAAGAAGCGACTCCACTCAAATTCGTTCCTCCACCT
AAATTTTCCTCTTGGTCTACCCCGCACCCAAGCTCATTAATTTCTTCTTCCTTTTTTTGCAAATCTCATTAATTTCGCAGTAATACAACTGTTAAGAGCT
||||||||||||||||||||||||||||||||||||||||||||||||||||||||||||||||||||||||||||||||||||||||||||||||||||
AAATTTTCCTCTTGGTCTACCCCGCACCCAAGCTCATTAATTTCTTCTTCCTTTTTTTGCAAATCTCATTAATTTCGCAGTAATACAACTGTTAAGAGCT
CAACCATGTGCTAGCTAATCAATTTAGTTTGTGGTGTGAACTGAGAGTAGTCCTGATGGTTAGGTTCCTTGTGGGCTCGATACCTCGGAGGTGCTTATAG
||||||||||||||||||||||||||||||||||||||||||||||||||||||||||||||||||||||||||||||||||||||||||||||||||||
CAACCATGTGCTAGCTAATCAATTTAGTTTGTGGTGTGAACTGAGAGTAGTCCTGATGGTTAGGTTCCTTGTGGGCTCGATACCTCGGAGGTGCTTATAG
AGATAGGGTGAGCGTGCATATGTTTTTAGGGATGAGTGCACAATTATGTTGTATTAACAAAATTTTAGTTTGAGAGAACACATCTTTTTTATAAAAAGTA
||||||||||||||||||||||||||||||||||||||||||||||||||||||||||||||||||||||||||||||||||||||||||||||||||||
AGATAGGGTGAGCGTGCATATGTTTTTAGGGATGAGTGCACAATTATGTTGTATTAACAAAATTTTAGTTTGAGAGAACACATCTTTTTTATAAAAAGTA
TTTTTATTAACTTAAAATGTAGTATCGAGTCAATACAAAGCATAGAGAATACGTAGAAAACATCGAGGTGATATAGACTGTTGTATATTCGCTCTCGGCC
||||||||||||||||||||||||||||||||||||||||||||||||||||||||||||||||||||||||||||||||||||||||||||||||||||
TTTTTATTAACTTAAAATGTAGTATCGAGTCAATACAAAGCATAGAGAATACGTAGAAAACATCGAGGTGATATAGACTGTTGTATATTCGCTCTCGGCC
ACTCTCATGCGCCGCACGTACCGCCAGCACGGGCACCTCTCCTTTCCTAGACGGGCAATAGGCCATTTGACCCACCAACTCCCCCTTTCCCAAACGCGAC
||||||||||||||||||||||||||||||||||||||||||||||||||||||||||||||||||||||||||||||||||||||||||||||||||||
ACTCTCATGCGCCGCACGTACCGCCAGCACGGGCACCTCTCCTTTCCTAGACGGGCAATAGGCCATTTGACCCACCAACTCCCCCTTTCCCAAACGCGAC
TCTTCCTCCTCCTCGCCACAGGAGGCAGAGGAATGGACTTGGTGGCCGGCCGATCCATAGATTCCCGTCGACAGCAACCATCGGATCGGACCGGTCAGGG
||||||||||||||||||||||||||||||||||||||||||||||||||||||||||||||||||||||||||||||||||||||||||||||||||||
TCTTCCTCCTCCTCGCCACAGGAGGCAGAGGAATGGACTTGGTGGCCGGCCGATCCATAGATTCCCGTCGACAGCAACCATCGGATCGGACCGGTCAGGG
CAGCCTGACGCACGCAGGACACCTCGTGATCCAGCAGCTATAGCTAGCTAGAGGCGTCGCGCTGGAGGAGCGAGCTAGCTAGCCCAGAGATCTGTCTCTC
||||||||||||||||||||||||||||||||||||||||||||||||||||||||||||||||||||||||||||||||||||||||||||||||||||
CAGCCTGACGCACGCAGGACACCTCGTGATCCAGCAGCTATAGCTAGCTAGAGGCGTCGCGCTGGAGGAGCGAGCTAGCTAGCCCAGAGATCTGTCTCTC
CTCTCCCCCTCTTCTATAAGTACGTCCGCTCGTCGTGGTGCAGGTGCAGCAGCAACAACGACGGGAGCAGCAAAACCACACACACGCAGACGCAGCTAGA
||||||||||||||||||||||||||||||||||||||||||||||||||||||||||||||||||||||||||||||||||||||||||||||||||||
CTCTCCCCCTCTTCTATAAGTACGTCCGCTCGTCGTGGTGCAGGTGCAGCAGCAACAACGACGGGAGCAGCAAAACCACACACACGCAGACGCAGCTAGA
GCTAATAGAGCTGGCCGCCTTCATCAACATACCGCCACTTGTGCCGGCGGCCGGCGGAGAAGGACCAGCGGTGACAGGAGGAAGAAGATGGTGGGGAGCG
||||||||||||||||||||||||||||||||||||||||||||||||||||||||||||||||||||||||||||||||||||||||||||||||||||
GCTAATAGAGCTGGCCGCCTTCATCAACATACCGCCACTTGTGCCGGCGGCCGGCGGAGAAGGACCAGCGGTGACAGGAGGAAGAAGATGGTGGGGAGCG
GCATGCATGCGCAGCGCGGGGACCCGCTGGTGGTGGGTCGCGTGATCGGCGACGTGGTGGACCCGTTCGTGCGGCGGGTGGCGCTGCGGGTCGGCTACGC
||||||||||||||||||||||||||||||||||||||||||||||||||||||||||||||||||||||||||||||||||||||||||||||||||||
GCATGCATGCGCAGCGCGGGGACCCGCTGGTGGTGGGTCGCGTGATCGGCGACGTGGTGGACCCGTTCGTGCGGCGGGTGGCGCTGCGGGTCGGCTACGC
GTCCAGGGACGTGGCCAACGGCTGCGAGCTCAGGCCGTCCGCCATCGCCGACCCGCCGCGCGTCGAGGTCGGCGGCCCGGACATGCGCACTTTCTACACG
||||||||||||||||||||||||||||||||||||||||||||||||||||||||||||||||||||||||||||||||||||||||||||||||||||
GTCCAGGGACGTGGCCAACGGCTGCGAGCTCAGGCCGTCCGCCATCGCCGACCCGCCGCGCGTCGAGGTCGGCGGCCCGGACATGCGCACTTTCTACACG
CTGGTGAGTTCCACTCCGTCCGTACGTGCCATGCTCGCGCGCTCCTGCTTAATTGCTCCTCCCAGTCCTAGATTACTTACTCTAGTGGGTGCGCGCAGGT
||||||||||||||||||||||||||||||||||||||||||||||||||||||||||||||||||||||||||||||||||||||||||||||||||||
CTGGTGAGTTCCACTCCGTCCGTACGTGCCATGCTCGCGCGCTCCTGCTTAATTGCTCCTCCCAGTCCTAGATTACTTACTCTAGTGGGTGCGCGCAGGT
GATGGTGGATCCGGATGCTCCAAGCCCCAGCGATCCCAGCCTTAGGGAGTACTTGCACTGGTGAGAGCAGAGCATGCACCAACAAACAAAACAAAACAAA
||||||||||||||||||||||||||||||||||||||||||||||||||||||||||||||||||||||||||||||||||||||||||||||||||||
GATGGTGGATCCGGATGCTCCAAGCCCCAGCGATCCCAGCCTTAGGGAGTACTTGCACTGGTGAGAGCAGAGCATGCACCAACAAACAAAACAAAACAAA
AGATTTCCCTCCTACTTCCCTAGCTAGCTTGCTTGGAAATATCCCCACCAACATGCCGTTCCCCTGACCTAGCACCAGCAGAGTCTCTATATAAATTCAG
||||||||||||||||||||||||||||||||||||||||||||||||||||||||||||||||||||||||||||||||||||||||||||||||||||
AGATTTCCCTCCTACTTCCCTAGCTAGCTTGCTTGGAAATATCCCCACCAACATGCCGTTCCCCTGACCTAGCACCAGCAGAGTCTCTATATAAATTCAG
ATTTATATATTGCCCCAGGCAGATATTTGTATACAGCTGACCGGGCCAAATAGGATCTTTCAGCCCAAAAAGCCTTCGAAGGGATTAATGAGGATAGCTC
||||||||||||||||||||||||||||||||||||||||||||||||||||||||||||||||||||||||||||||||||||||||||||||||||||
ATTTATATATTGCCCCAGGCAGATATTTGTATACAGCTGACCGGGCCAAATAGGATCTTTCAGCCCAAAAAGCCTTCGAAGGGATTAATGAGGATAGCTC
ACTCACTCTCTCACGTGGTGTGGTGGAGACATATATGGCTGAATGGCCCACTCATCACCACCTGAGTTCAATCAAATTCTTGTGCGCCCAACCTCCCCTC
||||||||||||||||||||||||||||||||||||||||||||||||||||||||||||||||||||||||||||||||||||||||||||||||||||
ACTCACTCTCTCACGTGGTGTGGTGGAGACATATATGGCTGAATGGCCCACTCATCACCACCTGAGTTCAATCAAATTCTTGTGCGCCCAACCTCCCCTC
CAACTCAAAAGTACTTCATGCTAGGGCTCTAGCAGATTTCAAAGCCAAAGATAAAAGTGCTAAATAAATGAAAAGGAACAGCTTCTTGATTTTATGCGCA
||||||||||||||||||||||||||||||||||||||||||||||||||||||||||||||||||||||||||||||||||||||||||||||||||||
CAACTCAAAAGTACTTCATGCTAGGGCTCTAGCAGATTTCAAAGCCAAAGATAAAAGTGCTAAATAAATGAAAAGGAACAGCTTCTTGATTTTATGCGCA
GAACAACTAAATGATACACAAATAAGTAAATACTCCCTCTGTAAACAAATGTATGACATTTTAGATCACTACTGCCCCCCTAAACAAAATATAAGACGTT
||||||||||||||||||||||||||||||||||||||||||||||||||||||||||||||||||||||||||||||||||||||||||||||||||||
GAACAACTAAATGATACACAAATAAGTAAATACTCCCTCTGTAAACAAATGTATGACATTTTAGATCACTACTGCCCCCCTAAACAAAATATAAGACGTT
TTTTATGCCCTATGCAGAACCAAAAAACGTCTTACATTTGTTTACAGAGGTAGTAATTAAATGCTAAGGTATATTTCTTACGTATTTTTTTGTGCATGAT
||||||||||||||||||||||||||||||||||||||||||||||||||||||||||||||||||||||||||||||||||||||||||||||||||||
TTTTATGCCCTATGCAGAACCAAAAAACGTCTTACATTTGTTTACAGAGGTAGTAATTAAATGCTAAGGTATATTTCTTACGTATTTTTTTGTGCATGAT
GCTAGATTGACATATACGTACAGTTTAGTGGTCAGGGAATGCACTTTTAGATGCCCAATAATACGATTAAGCAACTATATGTACCTATAAAAGAGTTTAT
||||||||||||||||||||||||||||||||||||||||||||||||||||||||||||||||||||||||||||||||||||||||||||||||||||
GCTAGATTGACATATACGTACAGTTTAGTGGTCAGGGAATGCACTTTTAGATGCCCAATAATACGATTAAGCAACTATATGTACCTATAAAAGAGTTTAT
GTATGCTTTTAAATCATCTCACCGATTAGTAACTTCAGAATTATCTTAGGTAATCCTATATATGCACATCATCAAAGCATATTATCTAGACCCGTGCATG
||||||||||||||||||||||||||||||||||||||||||||||||||||||||||||||||||||||||||||||||||||||||||||||||||||
GTATGCTTTTAAATCATCTCACCGATTAGTAACTTCAGAATTATCTTAGGTAATCCTATATATGCACATCATCAAAGCATATTATCTAGACCCGTGCATG
AGAGGCGCATCTACTTAGGAGTGGAAAATTTTATAAAAAAAATAAACATCAACACGCACGCATGCACACATACACCTATTCTAATGCATAAAGCATATTA
||||||||||||||||||||||||||||||||||||||||||||||||||||||||||||||||||||||||||||||||||||||||||||||||||||
AGAGGCGCATCTACTTAGGAGTGGAAAATTTTATAAAAAAAATAAACATCAACACGCACGCATGCACACATACACCTATTCTAATGCATAAAGCATATTA
AACAAAAGTTAAAAGGACAAATATTCAAGGCATACCAAATTACTATGAAATCGCAAGTTGTTTGCATTGTACAACTAGAATCAGACTGAAATAGCCATGA
||||||||||||||||||||||||||||||||||||||||||||||||||||||||||||||||||||||||||||||||||||||||||||||||||||
AACAAAAGTTAAAAGGACAAATATTCAAGGCATACCAAATTACTATGAAATCGCAAGTTGTTTGCATTGTACAACTAGAATCAGACTGAAATAGCCATGA
CAATTATATATTTGATAATTAGTGTTATCATATTATGGGTTTATATTTGCTTGCATTAAAAATAAAAATAGTTCGTGTGCAACAATTTAACAAGGACAAA
||||||||||||||||||||||||||||||||||||||||||||||||||||||||||||||||||||||||||||||||||||||||||||||||||||
CAATTATATATTTGATAATTAGTGTTATCATATTATGGGTTTATATTTGCTTGCATTAAAAATAAAAATAGTTCGTGTGCAACAATTTAACAAGGACAAA
GCTAGTTGCACCATAAATGCGATTACAAATTCATTGCTAAGTTTACTAGAACTCAACTTCCAATGAACTAAAGCAAGCGAAGCACATAAATTTAAGTAAA
||||||||||||||||||||||||||||||||||||||||||||||||||||||||||||||||||||||||||||||||||||||||||||||||||||
GCTAGTTGCACCATAAATGCGATTACAAATTCATTGCTAAGTTTACTAGAACTCAACTTCCAATGAACTAAAGCAAGCGAAGCACATAAATTTAAGTAAA
AATGAAAGGTTGGAGTTGCTAGATGTAGCAAATTGTTTTCGGTTCTATAGAAGATGCATGTACCGTTAGCCCGTCACTGATAAAACAAAATGACATTTTA
||||||||||||||||||||||||||||||||||||||||||||||||||||||||||||||||||||||||||||||||||||||||||||||||||||
AATGAAAGGTTGGAGTTGCTAGATGTAGCAAATTGTTTTCGGTTCTATAGAAGATGCATGTACCGTTAGCCCGTCACTGATAAAACAAAATGACATTTTA
CGGCTTTGTATTACTCCCTCCGTCCCAAAATAAGTGTCTTGAGCTCAAGACACTTATTTTGGAACGGAGGGAGTAAATTATATGCTCTAGTCTGTAGATT
||||||||||||||||||||||||||||||||||||||||||||||||||||||||||||||||||||||||||||||||||||||||||||||||||||
CGGCTTTGTATTACTCCCTCCGTCCCAAAATAAGTGTCTTGAGCTCAAGACACTTATTTTGGAACGGAGGGAGTAAATTATATGCTCTAGTCTGTAGATT
ATTAACTAGACTTGCAATGAAATGTATATTACTAATAAAAAACTCCATCCTTGATAATACGTATTTAGCTAAAGATTTTGTGGGGGGGGG
||||||||||||||||||||||||||||||||||||||||||||||||||||||||||||||||||||||||||||||||||||||||||
ATTAACTAGACTTGCAATGAAATGTATATTACTAATAAAAAACTCCATCCTTGATAATACGTATTTAGCTAAAGATTTTGTGGGGGGGGG

***FT-A3 - TRIAE_CS42_1AL_TGACv1_002602_AA0043540***

CACCCATGTGAAGAAGGAACAAGTGGATTCTATTTTGCTATTGCGGAGCAGTAAGTGGATTCTACTTTGCTATCCCAGGGCAGTAAAAAGAATCCTTATC

||||||||||||||||||||||||||||||||||||||||||||||||||||||||||||||||||||||||||||||||||||||||||||||||||||

CACCCATGTGAAGAAGGAACAAGTGGATTCTATTTTGCTATTGCGGAGCAGTAAGTGGATTCTACTTTGCTATCCCAGGGCAGTAAAAAGAATCCTTATC

ATCAAATGAATACACGTAGCAGTCAACTTTGTAAAGTTTCTACAAAACTTTCCATATGCCATATAAATAATATCCGCAGGTAGTTCAGAAATCGTGCTAT

||||||||||||||||||||||||||||||||||||||||||||||||||||||||||||||||||||||||||||||||||||||||||||||||||||

ATCAAATGAATACACGTAGCAGTCAACTTTGTAAAGTTTCTACAAAACTTTCCATATGCCATATAAATAATATCCGCAGGTAGTTCAGAAATCGTGCTAT

AGTCCTGTAGGGATGCAAGTGAACGGACCCACGGGCTGTTTTATCTAACTAATGGTATACTTCTACTTAGCTGATACTTCTGCAGGTACCTAACCAATAG

||||||||||||||||||||||||||||||||||||||||||||||||||||||||||||||||||||||||||||||||||||||||||||||||||||

AGTCCTGTAGGGATGCAAGTGAACGGACCCACGGGCTGTTTTATCTAACTAATGGTATACTTCTACTTAGCTGATACTTCTGCAGGTACCTAACCAATAG

TTTACTTTGTGCACAATAGCAAATAGCTAGATGGGTTGTTGGTTGGCCATCAGCCCAAACTTGCATCCTTATGTATGGATAGGCGAGCCTATCGAATAAA

||||||||||||||||||||||||||||||||||||||||||||||||||||||||||||||||||||||||||||||||||||||||||||||||||||

TTTACTTTGTGCACAATAGCAAATAGCTAGATGGGTTGTTGGTTGGCCATCAGCCCAAACTTGCATCCTTATGTATGGATAGGCGAGCCTATCGAATAAA

ACTGAATGGTTCCACTAGAAAAAGAATATAACAGAATTGTATTGTAGAAAACTGGCAAAAGAAATACTAAAATACATGATGGCCTACTGCGCTGGATAAC

||||||||||||||||||||||||||||||||||||||||||||||||||||||||||||||||||||||||||||||||||||||||||||||||||||

ACTGAATGGTTCCACTAGAAAAAGAATATAACAGAATTGTATTGTAGAAAACTGGCAAAAGAAATACTAAAATACATGATGGCCTACTGCGCTGGATAAC

TATTTTTAAGGCACGGTTAAAAATAAAGCCAACACAAAAGCAAACTACCTTAAATAAGCAATATCAGCAACACAGTGAAGAGATGCAATGATTAAGCTAG

||||||||||||||||||||||||||||||||||||||||||||||||||||||||||||||||||||||||||||||||||||||||||||||||||||

TATTTTTAAGGCACGGTTAAAAATAAAGCCAACACAAAAGCAAACTACCTTAAATAAGCAATATCAGCAACACAGTGAAGAGATGCAATGATTAAGCTAG

TGCTAGTCTAGACAAACTACCTTAGATCTTTGCTAAAAGTTGCACAGAACATAACAGTGGCTTCAAGAAGATGCATGCAGCTCATTTTTTCGGCAATAAG

||||||||||||||||||||||||||||||||||||||||||||||||||||||||||||||||||||||||||||||||||||||||||||||||||||

TGCTAGTCTAGACAAACTACCTTAGATCTTTGCTAAAAGTTGCACAGAACATAACAGTGGCTTCAAGAAGATGCATGCAGCTCATTTTTTCGGCAATAAG

TAGAAGAGTGATGGCCCTGTTCCAACATAGAGTATTGCTGCAACTTGGCAGGTCACAGTAGAAAAAATTCACTTTGTAAACAACAGTGACATGAGCAGGC

||||||||||||||||||||||||||||||||||||||||||||||||||||||||||||||||||||||||||||||||||||||||||||||||||||

TAGAAGAGTGATGGCCCTGTTCCAACATAGAGTATTGCTGCAACTTGGCAGGTCACAGTAGAAAAAATTCACTTTGTAAACAACAGTGACATGAGCAGGC

TCCTGGCCTGAGTGCAATTAAGTGGGTTGCAAGCATATGTTTTCCATTCCTACTAGTCCATTTTATGTAATCCCTGATTTTGAATAAAATAATGACCCTA

|||||||||||||||||||||||||||||||||||||||||||| |||||||||||||||||||||||||||||||||||||||||||||||||||||||

TCCTGGCCTGAGTGCAATTAAGTGGGTTGCAAGCATATGTTTTCTATTCCTACTAGTCCATTTTATGTAATCCCTGATTTTGAATAAAATAATGACCCTA

AGCTATAAGGAATATGAGTTTTCAGAAGGGAAAAAATCTTTTAATGCTGTGCAAATGCATTACATGCCAACAAGAAAACAATGTCAGCAGATATCTAAAA

||||||||||||||||||||||||||||||||||||||||||||||||||||||||||||||||||||||||||||||||||||||||||||||||||||

AGCTATAAGGAATATGAGTTTTCAGAAGGGAAAAAATCTTTTAATGCTGTGCAAATGCATTACATGCCAACAAGAAAACAATGTCAGCAGATATCTAAAA

AAAATACAAGGGAAAGTACTTAATACCAGTAAGCATGCAGTTGAAATGAAGAACCTAAGCATGTTAAGTACACCTACATGTTCAGCGTCACTACATTGTT

||||||||||||||||||||||||||||||||||||||||| ||||||||||||||||||||||||||||||||||||||||||||||||||||||||||

AAAATACAAGGGAAAGTACTTAATACCAGTAAGCATGCAGT-GAAATGAAGAACCTAAGCATGTTAAGTACACCTACATGTTCAGCGTCACTACATTGTT

AGGTTCAGAAATATGGTACCCATTATCTCCAAAGAGGTTGTGACCAAAGGAATGCGCTAACTATCGTTTACAATTCACAAGCTCGTCCCAGTATACCACA

||||||||||||||||||||||||||||||||||||||||||||||||||||||||||||||||||||||||||||||||||||||||||||||||||||

AGGTTCAGAAATATGGTACCCATTATCTCCAAAGAGGTTGTGACCAAAGGAATGCGCTAACTATCGTTTACAATTCACAAGCTCGTCCCAGTATACCACA

GCCATGCTAATAATAGGATGGCTTTAGCATGCATGTCAAAATATCTACGGCCCGACCACTCCATAAAGTACAGGATCTAATCGACAAATATCAATGACGT

||||||||||||||||||||||||||||||||||||||||||||||||||||||||||||||||||||||||||||||||||||||||||||||||||||

GCCATGCTAATAATAGGATGGCTTTAGCATGCATGTCAAAATATCTACGGCCCGACCACTCCATAAAGTACAGGATCTAATCGACAAATATCAATGACGT

TACGTACAGGTGACGCACAACATGACGCTAGTATCAAAACTTTTGAAAGGTGAATAAGACAACCCTCATCGCACAATACAAGTAGTTCGTGCAAGTAGGT

||||||||||||||||||||||||||||||||||||||||||||||||||||||||||||||||||||||||||||||||||||||||||||||||||||

TACGTACAGGTGACGCACAACATGACGCTAGTATCAAAACTTTTGAAAGGTGAATAAGACAACCCTCATCGCACAATACAAGTAGTTCGTGCAAGTAGGT

CATACATAGACACAATTTGTTTCAGGTCTGTAGAGAGCTAGGCACAATAACAGACAAAGGCTAAGGCTGTTAACTGGTAGTCCTCCAGTATATGTCGGCA

||||||||||||||||||||||||||||||||||||||||||||||||||||||||||||||||||||||||||||||||||||||||||||||||||||

CATACATAGACACAATTTGTTTCAGGTCTGTAGAGAGCTAGGCACAATAACAGACAAAGGCTAAGGCTGTTAACTGGTAGTCCTCCAGTATATGTCGGCA

GCGGATCCATTGGTTGTGGCCCATGTTCTACAAGATGTGCTTGATCCATTTACATCAACTGTTCCACTCAGGATAGCTTACAACAATAGGCTAGTCCTGG

||||||||||||||||||||||||||||||||||||||||||||||||||||||||||||||||||||||||||||||||||||||||||||||||||||

GCGGATCCATTGGTTGTGGCCCATGTTCTACAAGATGTGCTTGATCCATTTACATCAACTGTTCCACTCAGGATAGCTTACAACAATAGGCTAGTCCTGG

CAGGTGCTGAGCTAAGACCATCTGCAATTGTAAGCAAGCCACGAGTTGATATCAGTGGCAGTGACATGAGAGTTCTCTACACCCTGGTAAGCTTCTAACT

||||||||||||||||||||||||||||||||||||||||||||||||||||||||||||||||||||||||||||||||||||||||||||||||||||

CAGGTGCTGAGCTAAGACCATCTGCAATTGTAAGCAAGCCACGAGTTGATATCAGTGGCAGTGACATGAGAGTTCTCTACACCCTGGTAAGCTTCTAACT

CTAGTAGTGGACAATATATGCAAATCCTTTGGTTATTTCACTCTTTTACGTGGTTTGTTAGTATAACTGTGAAGGAAGTCCATAGAAGGAAAATGTCTCA

||||||||||||||||||||||||||||||||||||||||||||||||||||||||||||||||||||||||||||||||||||||||||||||||||||

CTAGTAGTGGACAATATATGCAAATCCTTTGGTTATTTCACTCTTTTACGTGGTTTGTTAGTATAACTGTGAAGGAAGTCCATAGAAGGAAAATGTCTCA

TAGATGATTAGTTTACTCCTAGCAAACTCAAACGAATCATATGTTAACTTTTTTGAGTTTGTCTTTGACTTCAAAAGATATTGGTGGATCCAGACGCCCC

||||||||||||||||||||||||||||||||||||||||||||||||||||||||||||||||||||||||||||||||||||||||||||||||||||

TAGATGATTAGTTTACTCCTAGCAAACTCAAACGAATCATATGTTAACTTTTTTGAGTTTGTCTTTGACTTCAAAAGATATTGGTGGATCCAGACGCCCC

AAGCCCAAGTCACCCATCACTAAGGGAGTACTTGCACTGGTAAATCAAATGCAACATGTTCTTTCTCGCTAATTCGATTCCAAACCCTCCACCCATCTGT

||||||||||||||||||||||||||||||||||||||||||||||||||||||||||||||||||||||||||||||||||||||||||||||||||||

AAGCCCAAGTCACCCATCACTAAGGGAGTACTTGCACTGGTAAATCAAATGCAACATGTTCTTTCTCGCTAATTCGATTCCAAACCCTCCACCCATCTGT

TTTCTGCCCCGAAACTTTTGATATATGCTTCCTATTTAAGTTGCCCTGATTACGATATATCTCCACTCCTGTGTAGGATGGTGTCAGACATCCCTGCAAC

||||||||||||||||||||||||||||||||||||||||||||||||||||||||||||||||||||||||||||||||||||||||||||||||||||

TTTCTGCCCCGAAACTTTTGATATATGCTTCCTATTTAAGTTGCCCTGATTACGATATATCTCCACTCCTGTGTAGGATGGTGTCAGACATCCCTGCAAC

AACTGGTGCCAGCTTTGGTATGATGTCATGCATAACAATTTGTTCAATGTGATTTTTGGTTTTAGCTGAATTTTGTGGATGCAGAAATCAGGCCATGTTA

||||||||||||||||||||||||||||||||||||||||||||||||||||||||||||||||||||||||||||||||||||||||||||||||||||

AACTGGTGCCAGCTTTGGTATGATGTCATGCATAACAATTTGTTCAATGTGATTTTTGGTTTTAGCTGAATTTTGTGGATGCAGAAATCAGGCCATGTTA

AGTTCTTTCCCTCAAATGACTCCATACAAGTTAATTCTAATATAGCCCATCGTTAACACCAAGATATCTTAAACCATGACAAAGAATAACTATAAACAGG

||||||||||||||||||||||||||||||||||||||||||||||||||||||||||||||||||||||||||||||||||||||||||||||||||||

AGTTCTTTCCCTCAAATGACTCCATACAAGTTAATTCTAATATAGCCCATCGTTAACACCAAGATATCTTAAACCATGACAAAGAATAACTATAAACAGG

GTAAAATGACATTTGTATGTACAGCCTAAAGAAGCAAGAACTCTTAAACAGTGGTATACTCATGAATTTCCTTTTTCTATCTATAATATTTATACTTGAT

||||||||||||||||||||||||||||||||||||||||||||||||||||||||||||||||||||||||||||||||||||| ||||||||||||||

GTAAAATGACATTTGTATGTACAGCCTAAAGAAGCAAGAACTCTTAAACAGTGGTATACTCATGAATTTCCTTTTTCTATCTATACTATTTATACTTGAT

TTATTTTTCACGTGTACAAACACAAAATTCATGCGATCAATTAATATCATTCAATCTTATACTGGTTTTGAAGAGTTTTATGATATATTTTTCTACCAAC

||||||||||||||||||||||||||||||||||||||||||||||||||||||||||||||||||||||||||||||||||||||||||||||||||||

TTATTTTTCACGTGTACAAACACAAAATTCATGCGATCAATTAATATCATTCAATCTTATACTGGTTTTGAAGAGTTTTATGATATATTTTTCTACCAAC

TAACATATACAGCAGAACAAAGGTTACCCCCTATACATGAAGATTATATTAGAAGACACCTATTTGCCTAGAACTGAACAAAGTAGACTACGTTGCAGAT

||||||||||||||||||||||||||||||||||||||||||||||||||||||||||||||||||||||||||||||||||||||||||||||||||||

TAACATATACAGCAGAACAAAGGTTACCCCCTATACATGAAGATTATATTAGAAGACACCTATTTGCCTAGAACTGAACAAAGTAGACTACGTTGCAGAT

AAATAACTAGATATTTCCACCAAGTTGAATTACTTTTGAAACTCTAAACTAATTAATATTAGGGTGCTCTTGCAGGCCAAGAGCTTGTAGTTTATGAAAG

||||||||||||||||||||||||||||||||||||||||||||||||||||||||||||||||||||||||||||||||||||||||||||||||||||

AAATAACTAGATATTTCCACCAAGTTGAATTACTTTTGAAACTCTAAACTAATTAATATTAGGGTGCTCTTGCAGGCCAAGAGCTTGTAGTTTATGAAAG

ACCAGAACCCAGATCTGGTATCCATCGGATGGTATTTGTGCTGTTCCAGCAACTAGGCAGGGGTACAGTTTTTGCACCAGACGTGCGACACAACTTCAGC

||||||||||||||||||||||||||||||||||||||||||||||||||||||||||||||||||||||||||||||||||||||||||||||||||||

ACCAGAACCCAGATCTGGTATCCATCGGATGGTATTTGTGCTGTTCCAGCAACTAGGCAGGGGTACAGTTTTTGCACCAGACGTGCGACACAACTTCAGC

TGCAGGAACTTTGCACGGCAGCACCACCTCAATATTGTGGCTGTCTCATATTTCAACTGTCAAAGGGAAGGTGGATCAGGTGGAAGAAGGTTTAGGCCAG

||||||||||||||||||||||||||||||||||||||||||||||||||||||||||||||||||||||||||||||||||||||||||||||||||||

TGCAGGAACTTTGCACGGCAGCACCACCTCAATATTGTGGCTGTCTCATATTTCAACTGTCAAAGGGAAGGTGGATCAGGTGGAAGAAGGTTTAGGCCAG

AAAGTTCTCAAGGGGAGTAGAGATTAGATACTACAGAGTAAAGACCATTGTATGCTGCACCGTAGTGTTGCATCACAAATAATGTGTAGCATATAGAATA

||||||||||||||||||||||||||||||||||||||||||||||||||||||||||||||||||||||||||||||||||||||||||||||||||||

AAAGTTCTCAAGGGGAGTAGAGATTAGATACTACAGAGTAAAGACCATTGTATGCTGCACCGTAGTGTTGCATCACAAATAATGTGTAGCATATAGAATA

CCCTGTATCATCTAAATATGCAGCATTATATGCATCTTCAGCTATGATGAGCTTGATGGGATATATCTCAGTCATTTGTTAATCATCTGATCACGGAGAG

||||||||||||||||||||||||||||||||||||||||||||||||||||||||||||||||||||||||||||||||||||||||||||||||||||

CCCTGTATCATCTAAATATGCAGCATTATATGCATCTTCAGCTATGATGAGCTTGATGGGATATATCTCAGTCATTTGTTAATCATCTGATCACGGAGAG

TATTCTTGTGGGAATACAAAAGATAAACTCAGGCTTTTACAATATTAGTTAAGCTCAACTAGTTTATAGTTCTGAGCACCTGCTTTGCATTTTAACAAGT

||||||||||||||||||||||||||||||||||||||||||||||||||||||||||||||||||||||||||||||||||||||||||||||||||||

TATTCTTGTGGGAATACAAAAGATAAACTCAGGCTTTTACAATATTAGTTAAGCTCAACTAGTTTATAGTTCTGAGCACCTGCTTTGCATTTTAACAAGT

TCTATTGAATAAATCAACACACTGTAAATCATCAAAGGTATTTTTGAAAGAAAGCCCAAGATATTTTAGAGCCCACAAGTGGCAAAGTAAACCCCAAGGT

||||||||||||||||||||||||||||||||||||||||||||||||||||||||||||||||||||||||||||||||||||||||||||||||||||

TCTATTGAATAAATCAACACACTGTAAATCATCAAAGGTATTTTTGAAAGAAAGCCCAAGATATTTTAGAGCCCACAAGTGGCAAAGTAAACCCCAAGGT

ATGTCCAATGTTCGCATTATTCCTTATTCAAAGCAACAGGGGTGCTGGACATTCACATCATTACATCAAACACTTAAAAGTGAAAACATAATATTACAAG

||||||||||||||||||||||||||||||||||||||||||||||||||||||||||||||||||||||||||||||||||||||||||||||||||||

ATGTCCAATGTTCGCATTATTCCTTATTCAAAGCAACAGGGGTGCTGGACATTCACATCATTACATCAAACACTTAAAAGTGAAAACATAATATTACAAG

ACCCCAAGAGCAGTCACAACTTACAGCAGAAGCATATCATCGGGAGGAACCGATCACTTCTTCCCAAGGTGGCATACCTCCACCAGTTCCCAGGACTCCA

||||||||||||||||||||||||||||||||||||||||||||||||||||||||||||||||||||||||||||||||||||||||||||||||||||

ACCCCAAGAGCAGTCACAACTTACAGCAGAAGCATATCATCGGGAGGAACCGATCACTTCTTCCCAAGGTGGCATACCTCCACCAGTTCCCAGGACTCCA

AGCTTTTATCCTCAGTATCCGTTCGATAAAGTGCGAGCTCGGAGATCTCAAACTGCAGTCCAGAAATTTCACTGCCCATTTCCTCTACTTTCTTCCTTGC

||||||||||||||||||||||| ||||||||||||||||||||||||||||||||||||||||||||||||||||||||||||||||||||||||||||

AGCTTTTATCCTCAGTATCCGTTTGATAAAGTGCGAGCTCGGAGATCTCAAACTGCAGTCCAGAAATTTCACTGCCCATTTCCTCTACTTTCTTCCTTGC

TGCTTCCTTCTCTTCGTCTGTTAGATCCCCATACAGGAGACTTACATGTGGCATATATGCT

|||||||||||||||||||||||||||||||||||||||||||||||||||||||||||||

TGCTTCCTTCTCTTCGTCTGTTAGATCCCCATACAGGAGACTTACATGTGGCATATATGCT

***FT-B3 - TRIAE_CS42_1BL_TGACv1_030923_AA0103850***

GTGAAAAAGGAACAAGTGGATTTTACTTTGTTATCGTAGAGCAGTAAGTGGATTCTACTTTGCTATCTCAGAGCAGTAAATAGAATCCTTGTCATCAAAT
||||||||||||||||||||||||||||||||||||||||||||||||||||||||||||||||||||||||||||||||||||||||||||||||||||
GTGAAAAAGGAACAAGTGGATTTTACTTTGTTATCGTAGAGCAGTAAGTGGATTCTACTTTGCTATCTCAGAGCAGTAAATAGAATCCTTGTCATCAAAT
GAAAGCGCGTCGCAGTCAACTTTGTAAAGTTTCTATAAAACTTTCCATATGTCATACAAATAATATCCGCTTCAGAACTTGTGGTATAGTCCTGTAGGGA
||||||||||||||||||||||||||||||||||||||||||||||||||||||||||||||||||||||||||||||||||||||||||||||||||||
GAAAGCGCGTCGCAGTCAACTTTGTAAAGTTTCTATAAAACTTTCCATATGTCATACAAATAATATCCGCTTCAGAACTTGTGGTATAGTCCTGTAGGGA
TGCAAGTCAGCAAGTGGATGGACCCATGGGCTGTTTTATCCAACTAATGGTATACTTCTACTTAGCTGATAATTCTGCAGGTACCTAACCAATAGTTTAC
||||||||||||||||||||||||||||||||||||||||||||||||||||||||||||||||||||||||||||||||||||||||||||||||||||
TGCAAGTCAGCAAGTGGATGGACCCATGGGCTGTTTTATCCAACTAATGGTATACTTCTACTTAGCTGATAATTCTGCAGGTACCTAACCAATAGTTTAC
TTTGTGCACATAGCAAATAGCTGGATGGGTTGTTGCGTTGGCCCAATCAGCCCAAACTTGCATCCTTATGTATGGATAGGCGAGCCTATCGAATAAAACT
||||||||||||||||||||||||||||||||||||||||||||||||||||||||||||||||||||||||||||||||||||||||||||||||||||
TTTGTGCACATAGCAAATAGCTGGATGGGTTGTTGCGTTGGCCCAATCAGCCCAAACTTGCATCCTTATGTATGGATAGGCGAGCCTATCGAATAAAACT
GAATGGTTCCACTAGAAAAAAGAATATAACAGAATTGTATTGTAGAGAACTGGCAAAAAAAACCTAAAACACACGATGGCCTGCTGGGCTGGATAACTAC
||||||||||||||||||||||||||||||||||||||||||||||||||||||||||||||||||||||||||||||||||||||||||||||||||||
GAATGGTTCCACTAGAAAAAAGAATATAACAGAATTGTATTGTAGAGAACTGGCAAAAAAAACCTAAAACACACGATGGCCTGCTGGGCTGGATAACTAC
TAACGCACGGTTAAAACTAAAGCCAACACAAAAGCAGATTGCCTTAAATAAGCAATATCAGCAACACAATGAAGATATGCAATGATTAGGCTAGTGCTAG
||||||||||||||||||||||||||||||||||||||||||||||||||||||||||||||||||||||||||||||||||||||||||||||||||||
TAACGCACGGTTAAAACTAAAGCCAACACAAAAGCAGATTGCCTTAAATAAGCAATATCAGCAACACAATGAAGATATGCAATGATTAGGCTAGTGCTAG
TCTATAAACTACCTTAGATCTTAGCTAAAAGTTGCACAGAACAAAGAACATAACAATGGCTTCAAGAAGATGCATGCAGCTCACTTTTCGCATAAACAGA
||||||||||||||||||||||||||||||||||||||||||||||||||||||||||||||||||||||||||||||||||||||||||||||||||||
TCTATAAACTACCTTAGATCTTAGCTAAAAGTTGCACAGAACAAAGAACATAACAATGGCTTCAAGAAGATGCATGCAGCTCACTTTTCGCATAAACAGA
AGTGATGACCCTGTTCCAACATAGAGTAGCGTTGCAACTTGGCAGGTCACAGTAGAAAAAATCCACTTTGTAAACAACAGTAATGCTTGCGATTAAGTGG
||||||||||||||||||||||||||||||||||||||||||||||||||||||||||||||||||||||||||||||||||||||||||||||||||||
AGTGATGACCCTGTTCCAACATAGAGTAGCGTTGCAACTTGGCAGGTCACAGTAGAAAAAATCCACTTTGTAAACAACAGTAATGCTTGCGATTAAGTGG
GTTGCAAGCATATGTTTCTCATTTCTACTAGTCCATTTTATGCAATCCCTGAATTTGAATAAAATAATGGCCCTAAGCTATAAGGAATATGAGTTTTCAG
||||||||||||||||||||||||||||||||||||||||||||||||||||||||||||||||||||||||||||||||||||||||||||||||||||
GTTGCAAGCATATGTTTCTCATTTCTACTAGTCCATTTTATGCAATCCCTGAATTTGAATAAAATAATGGCCCTAAGCTATAAGGAATATGAGTTTTCAG
AAGGGAAAACATCTTCTAATGTTGTGCAAATGTGTTACATGCCAACAAGAAAACAACATCAGCAGATATCTCAAAAAAGTACAAGGGGAAGTACTTAATA
||||||||||||||||||||||||||||||||||||||||||||||||||||||||||||||||||||||||||||||||||||||||||||||||||||
AAGGGAAAACATCTTCTAATGTTGTGCAAATGTGTTACATGCCAACAAGAAAACAACATCAGCAGATATCTCAAAAAAGTACAAGGGGAAGTACTTAATA
CCACTAAGCATGCAGTTGAAATGAAGAACCTAAGTACGTAAAGTACAACTACATGTTTAGCTTCACTACATTGTTAATTAGGTTCAGAAATATGGTACCC
||||||||||||||||||||||||||||||||||||||||||||||||||||||||||||||||||||||||||||||||||||||||||||||||||||
CCACTAAGCATGCAGTTGAAATGAAGAACCTAAGTACGTAAAGTACAACTACATGTTTAGCTTCACTACATTGTTAATTAGGTTCAGAAATATGGTACCC
ATTATTTCCATAGAGGTTGTGATCTAAGGAATGCACTAACTATCGCCCACAATTCACAAGCTTGTTCCAGTATACCACAGCCATGCTAATGATCGGATGG
||||||||||||||||||||||||||||||||||||||||||||||||||||||||||||||||||||||||||||||||||||||||||||||||||||
ATTATTTCCATAGAGGTTGTGATCTAAGGAATGCACTAACTATCGCCCACAATTCACAAGCTTGTTCCAGTATACCACAGCCATGCTAATGATCGGATGG
CTTAAGCATGCATGTCGAAATATCTACGGCCCGACTACTCCATAAAGTTCAGGATCTAATCGACGAATATCAAAGACGTTACGTGTATACAGGTGACACA
||||||||||||||||||||||||||||||||||||||||||||||||||||||||||||||||||||||||||||||||||||||||||||||||||||
CTTAAGCATGCATGTCGAAATATCTACGGCCCGACTACTCCATAAAGTTCAGGATCTAATCGACGAATATCAAAGACGTTACGTGTATACAGGTGACACA
GAACATGACGCTAGTATCAAAACTTTTGAAAGGTGAATAAGACAACCCTCATCGCGCAATACAAGTAGTTCGTGCAAGTAGTTCATACATAGACACAGTT
||||||||||||||||||||||||||||||||||||||||||||||||||||||||||||||||||||||||||||||||||||||||||||||||||||
GAACATGACGCTAGTATCAAAACTTTTGAAAGGTGAATAAGACAACCCTCATCGCGCAATACAAGTAGTTCGTGCAAGTAGTTCATACATAGACACAGTT
TGTTTCAGGTCTCTAGAGAGCTAGGTTAACACAATAGCAGACAAGGACAAGGCTATTAATTGGTAGTCCTCCTCCAGTATATGTCGGCAGCGGATCCATT
||||||||||||||||||||||||||||||||||||||||||||||||||||||||||||||||||||||||||||||||||||||||||||||||||||
TGTTTCAGGTCTCTAGAGAGCTAGGTTAACACAATAGCAGACAAGGACAAGGCTATTAATTGGTAGTCCTCCTCCAGTATATGTCGGCAGCGGATCCATT
GGTTGTGGCTCATGTTTTACAAGATGTGCTTGATCCATTTACATCAACTGTTCCGCTCAGGATAGCCTACAACAATAGGCTAGTTCTGGCAGGTGCTGAG
||||||||||||||||||||||||||||||||||||||||||||||||||||||||||||||||||||||||||||||||||||||||||||||||||||
GGTTGTGGCTCATGTTTTACAAGATGTGCTTGATCCATTTACATCAACTGTTCCGCTCAGGATAGCCTACAACAATAGGCTAGTTCTGGCAGGTGCTGAG
CTAAGACCATCTGCAATTGTAAGCAAGCCACGAGTTGATATCGGTGGCAGTGACATGAGAGTCCTCTATACCCTGGTAAGCTTCTAACTCTAGTAGTGGA
||||||||||||||||||||||||||||||||||||||||||||||||||||||||||||||||||||||||||||||||||||||||||||||||||||
CTAAGACCATCTGCAATTGTAAGCAAGCCACGAGTTGATATCGGTGGCAGTGACATGAGAGTCCTCTATACCCTGGTAAGCTTCTAACTCTAGTAGTGGA
CAATATATGCAAATCCTTTGGTTATTTCACTCTTTTATGTGATTTGTTAGTATAATTGTGAAGGAATTCCATAGAAGAAAAATGTCTCATAGATGATTAA
||||||||||||||||||||||||||||||||||||||||||||||||||||||||||||||||||||||||||||||||||||||||||||||||||||
CAATATATGCAAATCCTTTGGTTATTTCACTCTTTTATGTGATTTGTTAGTATAATTGTGAAGGAATTCCATAGAAGAAAAATGTCTCATAGATGATTAA
TTTACTCCTAGCAAGCTCAAATGGATCATATGTTAACTTGTTTGAGTTTGTCTTTGACTTCAAAAGATATTGGTGGATCCAGACGCCCCAAGCCCAAGTC
||||||||||||||||||||||||||||||||||||||||||||||||||||||||||||||||||||||||||||||||||||||||||||||||||||
TTTACTCCTAGCAAGCTCAAATGGATCATATGTTAACTTGTTTGAGTTTGTCTTTGACTTCAAAAGATATTGGTGGATCCAGACGCCCCAAGCCCAAGTC
ACCCATCACTAAGGGAGTACTTGCACTGGTAAATCAAATGCAACATGTTCTTATTCTCGCCAATACGATCCCAAACCTCCACCCATCTGTTTTCTGCCCC
||||||||||||||||||||||||||||||||||||||||||||||||||||||||||||||||||||||||||||||||||||||||||||||||||||
ACCCATCACTAAGGGAGTACTTGCACTGGTAAATCAAATGCAACATGTTCTTATTCTCGCCAATACGATCCCAAACCTCCACCCATCTGTTTTCTGCCCC
CGAAACTTTTGATATATGCTTCCTATTTAATAGGATCTGATTATGATATATCTCCACTCCTGTGTAGGATGGTGTCCGACATCCCTGGAACAACTAGTGG
||||||||||||||||||||||||||||||||||||||||||||||||||||||||||||||||||||||||||||||||||||||||||||||||||||
CGAAACTTTTGATATATGCTTCCTATTTAATAGGATCTGATTATGATATATCTCCACTCCTGTGTAGGATGGTGTCCGACATCCCTGGAACAACTAGTGG
CAGCTTCGGTATGATGTCATGCATAACAATTTGTTCAATGTTATTTTTGGTTTTAGCTGAATTTTGCAGATGCATAAATCAGGTCATGTTAAGTTCTTTC
||||||| ||||||||||||||||||||||||||||||||||||||||||||||||||||||||||||||||||||||||||||||||||||||||||||
CAGCTTCAGTATGATGTCATGCATAACAATTTGTTCAATGTTATTTTTGGTTTTAGCTGAATTTTGCAGATGCATAAATCAGGTCATGTTAAGTTCTTTC
CCTCAAACGACTCGATACAAGCTAATTCCAATATAGTCCATCATTAACACCAAGATATCTTAAACCATGACAAAGAATAAATCTAAACAGGGTAAAATGA
||||||||||||||||||||||||||||||||||||||||||||||||||||||||||||||||||||||||||||||||||||||||||||||||||||
CCTCAAACGACTCGATACAAGCTAATTCCAATATAGTCCATCATTAACACCAAGATATCTTAAACCATGACAAAGAATAAATCTAAACAGGGTAAAATGA
CATTTGTATGTACAGCCTAAAGAAGAAAGAACTCTTAAAGCAGTGTGTACTTAAGAATTTCCTTTTTCTATCTACTATTTATACTTGATTTATTTTGCAT
||||||||||||||||||||||||||||||||||||||||||||||||||||||||||||||||||||||||||||||||||||||||||||||||||||
CATTTGTATGTACAGCCTAAAGAAGAAAGAACTCTTAAAGCAGTGTGTACTTAAGAATTTCCTTTTTCTATCTACTATTTATACTTGATTTATTTTGCAT
ATGTACAAACACAAAATTCATGCAATCAATTAATATCATTCAATCTTATACTGGTTTTGAAGAGTTTTATGGTACACTCCCTATACATATACAGCAGATT
||||||||||||||||||||||||||||||||||||||||||||||||||||||||||||||||||||||||||||||||||||||||||||||||||||
ATGTACAAACACAAAATTCATGCAATCAATTAATATCATTCAATCTTATACTGGTTTTGAAGAGTTTTATGGTACACTCCCTATACATATACAGCAGATT
ATATTAGAAGACTCCTTTTTGCCTAGAACTGAACAAAGTAGACCACATCGCAGATAAATGACTAAATGTTTCCACCAAGTTGAATTACTCTTTAAATTCT
||||||||||||||||||||||||||||||||||||||||||||||||||||||||||||||||||||||||||||||||||||||||||||||||||||
ATATTAGAAGACTCCTTTTTGCCTAGAACTGAACAAAGTAGACCACATCGCAGATAAATGACTAAATGTTTCCACCAAGTTGAATTACTCTTTAAATTCT
AAACTAATTAATACTAGGGTGCTCTCGCAGGCCAAGAGCTTGTAGTTTATGAAAGACCAGAACCCAGATCTGGTATTCACCGGATGGTATTTGTGCTGTT
||||||||||||||||||||||||||||||||||||||||||||||||||||||||||||||||||||||||||||||||||||||||||||||||||||
AAACTAATTAATACTAGGGTGCTCTCGCAGGCCAAGAGCTTGTAGTTTATGAAAGACCAGAACCCAGATCTGGTATTCACCGGATGGTATTTGTGCTGTT
CCAGCAACTAGGCAGGGGAACAGTTTTTGCACCAGATGTGCGACACAATTTCAGCTGCAGAAACTTTGCACGGCAGTACCACCTCAACATTGTGGCTGCC
||||||||||||||||||||||||||||||||||||||||||||||||||||||||||||||||||||||||||||||||||||||||||||||||||||
CCAGCAACTAGGCAGGGGAACAGTTTTTGCACCAGATGTGCGACACAATTTCAGCTGCAGAAACTTTGCACGGCAGTACCACCTCAACATTGTGGCTGCC
TCATATTTCAACTGTCAAAGGGAAGGTGGATCTGGCGGAAGAAGGTTTAGGCCAGAAAGTTCTCAAGGGGAGTAGAGATTAGATAGTACAGAGTAAAGAC
||||||||||||||||||||||||||||||||||||||||||||||||||||||||||||||||||||||||||||||||||||||||||||||||||||
TCATATTTCAACTGTCAAAGGGAAGGTGGATCTGGCGGAAGAAGGTTTAGGCCAGAAAGTTCTCAAGGGGAGTAGAGATTAGATAGTACAGAGTAAAGAC
CATTATATGCTGTACTGTAGTGTTGTACCACAAATAATGTGCAGTATATAGAATGTCTTGTATCATCTAAATATGCAGCATTATATGTATCTTCAGCTAT
||||||||||||||||||||||||||||||||||||||||||||||||||||||||||||||||||||||||||||||||||||||||||||||||||||
CATTATATGCTGTACTGTAGTGTTGTACCACAAATAATGTGCAGTATATAGAATGTCTTGTATCATCTAAATATGCAGCATTATATGTATCTTCAGCTAT
GATAAGCTTGATGGAATATATCTCAATCATTAGTTAATCACCTGATCATGGAAAGTATTCTTGAGGGAGTACAAAAGATAAACTCGGACTTTTACAATAT
||||||||||||||||||||||||||||||||||||||||||||||||||||||||||||||||||||||||||||||||||||||||||||||||||||
GATAAGCTTGATGGAATATATCTCAATCATTAGTTAATCACCTGATCATGGAAAGTATTCTTGAGGGAGTACAAAAGATAAACTCGGACTTTTACAATAT
TATTAGTTAAGCTCAACTAGTTTCTACTCAGCACCTGCTTTTCATTTTAATAGGTTCTACTGAATAAATCAACACACTGTAAATCATCAAAGTTATTTTG
||||||||||||||||||||||||||||||||||||||||||||||||||||||||||||||||||||||||||||||||||||||||||||||||||||
TATTAGTTAAGCTCAACTAGTTTCTACTCAGCACCTGCTTTTCATTTTAATAGGTTCTACTGAATAAATCAACACACTGTAAATCATCAAAGTTATTTTG
GAAAGTAAGCCCAAAATATTTTAGACGAAAGGCAATATTGGTCAACTTTGTAATAAAGCCCACAAGTGGCAAGGTAAAGCCCAAGGTAGGTCCAATGTTC
||||||||||||||||||||||||||||||||||||||||||||||||||||||||||||||||||||||||||||||||||||||||||||||||||||
GAAAGTAAGCCCAAAATATTTTAGACGAAAGGCAATATTGGTCAACTTTGTAATAAAGCCCACAAGTGGCAAGGTAAAGCCCAAGGTAGGTCCAATGTTC
ACATTATTCCTTATTCAAAGCAACAGGGGTGCCGGACATTCACATCATTACATCAAACACTTAAAAGTGAAAACATAATATTACAAGACCCCAAGAGCAG
||||||||||||||||||||||||||||||||||||||||||||||||||||||||||||||||||||||||||||||||||||||||||||||||||||
ACATTATTCCTTATTCAAAGCAACAGGGGTGCCGGACATTCACATCATTACATCAAACACTTAAAAGTGAAAACATAATATTACAAGACCCCAAGAGCAG
TCACAACTTACAGCAGAAGCATATCATCGGGAGGAATCGATCACTTCTTCCCAAGGTGGCATACCTCCACCAGTTCCCAGGACTCCAAGCTTTTATCCTC
||||||||||||||||||||||||||||||||||||||||||||||||||||||||||||||||||||||||||||||||||||||||||||||||||||
TCACAACTTACAGCAGAAGCATATCATCGGGAGGAATCGATCACTTCTTCCCAAGGTGGCATACCTCCACCAGTTCCCAGGACTCCAAGCTTTTATCCTC
AGTATCCGTTCGATAAAGTGCGAGCTCAGAGATCTCAAACTGCAGTCCAGATAGTTCACTGTCCATTTCCTCTACCTTCTTCCTTGCTGCTTCCTTCTCC
||||||||||||||||||||||||||||||||||||||||||||||||||||||||||||||||||||||||||||||||||||||||||||||||||||
AGTATCCGTTCGATAAAGTGCGAGCTCAGAGATCTCAAACTGCAGTCCAGATAGTTCACTGTCCATTTCCTCTACCTTCTTCCTTGCTGCTTCCTTCTCC
TCGTCTGTTAGATCCCCATACAGGAGGCTGACAT
||||||||||||||||||||||||||||||||||
TCGTCTGTTAGATCCCCATACAGGAGGCTGACAT

***FT-D3 - TRIAE_CS42_1DL_TGACv1_061682_AA0201670***

AAAGCATTTTCCTATTCAATCTTATGCCTTCTGGGAAGGGAACATTGTTATCTTGACAAAAGAGATGCGTGCTTCTCCTTTTATGTTTCAGTCACCGAGG
||||||||||||||||||||||||||||||||||||||||||||||||||||||||||||||||||||||||||||||||||||||||||||||||||||
AAAGCATTTTCCTATTCAATCTTATGCCTTCTGGGAAGGGAACATTGTTATCTTGACAAAAGAGATGCGTGCTTCTCCTTTTATGTTTCAGTCACCGAGG
ATATGTAAGGAGCACAAATCTTCTTTGCTCTGAGCATGGGAGTATCAAACCAGATATTTTGCCTGTATAGTGAACCACTAAAACCACAAGGGCATATACA
||||||||||||||||||||||||||||||||||||||||||||||||||||||||||||||||||||||||||||||||||||||||||||||||||||
ATATGTAAGGAGCACAAATCTTCTTTGCTCTGAGCATGGGAGTATCAAACCAGATATTTTGCCTGTATAGTGAACCACTAAAACCACAAGGGCATATACA
AGTACAATGGGTATATGTCAGCCTGCTTGTGGGGCCTCAGAAACGTGGGAATATAAAAAACCTATGATTTAAGTGGCATGTCCTCTTGAATCCTATAGGA
||||||||||||||||||||||||||||||||||||||||||||||||||||||||||||||||||||||||||||||||||||||||||||||||||||
AGTACAATGGGTATATGTCAGCCTGCTTGTGGGGCCTCAGAAACGTGGGAATATAAAAAACCTATGATTTAAGTGGCATGTCCTCTTGAATCCTATAGGA
TAGGAAAACTATGGGAATTCTGATCAAAGAATGTTTGAAAACACAGGAAAGACAAATAGAATGCAAATGAAACCTAAGAAAAATTCCTAAGGATTTCAAT
||||||||||||||||||||||||||||||||||||||||||||||||||||||||||||||||||||||||||||||||||||||||||||||||||||
TAGGAAAACTATGGGAATTCTGATCAAAGAATGTTTGAAAACACAGGAAAGACAAATAGAATGCAAATGAAACCTAAGAAAAATTCCTAAGGATTTCAAT
CCTGCGAATCAAGCAAAAATTGTAGATTTTTTTCCTACAGAAGAAAATCCCCCAAAGAGCCTATAAAATTCCTTTGAATCAAAGAAGCCCTATATGTTTT
||||||||||||||||||||||||||||||||||||||||||||||||||||||||||||||||||||||||||||||||||||||||||||||||||||
CCTGCGAATCAAGCAAAAATTGTAGATTTTTTTCCTACAGAAGAAAATCCCCCAAAGAGCCTATAAAATTCCTTTGAATCAAAGAAGCCCTATATGTTTT
TCTAGCATGTAACGTGATGTCTCTTCGGTGTCACTAGGTGATATGGAGGACAGCCTGAGGTAAACAAGGTATATATTCATATTTACCCCCACCCCCACCC
||||||||||||||||||||||||||||||||||||||||||||||||||||||||||||||||||||||||||||||||||||||||||||||||||||
TCTAGCATGTAACGTGATGTCTCTTCGGTGTCACTAGGTGATATGGAGGACAGCCTGAGGTAAACAAGGTATATATTCATATTTACCCCCACCCCCACCC
ATGTGAAGAAGGAACAAGTGGATTATATTTTGTTATCGCAGAGCAGTAAGTGGATTCTACTTTGCTATCGCAGGGCAGTAAATAGAATCCTTATCATCAA
||||||||||||||||||||||||||||||||||||||||||||||||||||||||||||||||||||||||||||||||||||||||||||||||||||
ATGTGAAGAAGGAACAAGTGGATTATATTTTGTTATCGCAGAGCAGTAAGTGGATTCTACTTTGCTATCGCAGGGCAGTAAATAGAATCCTTATCATCAA
ATGAATACACATAACAGTCAACTTTGTAAATTTTCTACAAAACTTTCCATATGCCATATAAATAATACCCGCAGGTAGTTCAGAAATTGTGCTACAGTCC
||||||||||||||||||||||||||||||||||||||||||||||||||||||||||||||||||||||||||||||||||||||||||||||||||||
ATGAATACACATAACAGTCAACTTTGTAAATTTTCTACAAAACTTTCCATATGCCATATAAATAATACCCGCAGGTAGTTCAGAAATTGTGCTACAGTCC
TGTAGGGATGCAAGTGGACGAACCCGCGGGCTGTTTTATCTAACTAATGGTATACTTCTACTTAGCTGATACTTCTGCAGGTACCTAACCAATAGTTTAC
||||||||||||||||||||||||||||||||||||||||||||||||||||||||||||||||||||||||||||||||||||||||||||||||||||
TGTAGGGATGCAAGTGGACGAACCCGCGGGCTGTTTTATCTAACTAATGGTATACTTCTACTTAGCTGATACTTCTGCAGGTACCTAACCAATAGTTTAC
TTTGTGCACATAGCAAATAGCTAGATGAGTTGTTGGGTTGGCCCAATCAGCCCAAACTTGCATCCTTATGTATGGATAGGCGAGCCTATCGAATAAAACT
||||||||||||||||||||||||||||||||||||||||||||||||||||||||||||||||||||||||||||||||||||||||||||||||||||
TTTGTGCACATAGCAAATAGCTAGATGAGTTGTTGGGTTGGCCCAATCAGCCCAAACTTGCATCCTTATGTATGGATAGGCGAGCCTATCGAATAAAACT
GAATGGTTCTACTAGAAAAAGATATAACAGAATTGTATTGTAGAAAACTGGCAAAAGAAATACTACAATACATGATGGCCTGCTGCGCTGGATAACTATT
||||||||| ||||||||||||||||||||||||||||||||||||||||||||||||||||||||||||||||||||||||||||||||||||||||||
GAATGGTTCCACTAGAAAAAGATATAACAGAATTGTATTGTAGAAAACTGGCAAAAGAAATACTACAATACATGATGGCCTGCTGCGCTGGATAACTATT
TTTAAGGCACGGTTAAAAATAAAGCCAACAACAAAAGCAAACTACCTTAAATAAGCAACATCAGCAACAAAGTGAAGAGATGCAATGATTAAGCTAGTGC
||||||||||||||||||||||||||||||||||||||||||||||||||||||||||||||||||||||||||||||||||||||||||||||||||||
TTTAAGGCACGGTTAAAAATAAAGCCAACAACAAAAGCAAACTACCTTAAATAAGCAACATCAGCAACAAAGTGAAGAGATGCAATGATTAAGCTAGTGC
TACTCTAGACAAACTACCTTAGATCTTTGCTAAAAGTTGCACAGAACATAACAATGGCTTCAAGAAGATGCATGCAGCTCACTTTTTCGGTGATAAACAG
||||||||||||||||||||||||||||||||||||||||||||||||||||||||||||||||||||||||||||||||||||||||||||||||||||
TACTCTAGACAAACTACCTTAGATCTTTGCTAAAAGTTGCACAGAACATAACAATGGCTTCAAGAAGATGCATGCAGCTCACTTTTTCGGTGATAAACAG
AAGAGTGATGGCTCTGTTCCAACATAGAGTATCGCTGCAACTTGGCAGGTCACAGTAGAAAAAGTTCACTTTGGAAACAACAGTAATGTCACCAGGCTCC
||||||||||||||||||||||||||||||||||||||||||||||||||||||||||||||||||||||||||||||||||||||||||||||||||||
AAGAGTGATGGCTCTGTTCCAACATAGAGTATCGCTGCAACTTGGCAGGTCACAGTAGAAAAAGTTCACTTTGGAAACAACAGTAATGTCACCAGGCTCC
TGCCCTGAGTGCAATTAAGTGGGTTGCAAGCATATGTGTTCCATTCCTGCTAGCCCATTTTATGTAATCCCTGAATTTGAATAAAATAATGACCCTAGGC
||||||||||||||||||||||||||||||||||||||||||||||||||||||||||||||||||||||||||||||||||||||||||||||||||||
TGCCCTGAGTGCAATTAAGTGGGTTGCAAGCATATGTGTTCCATTCCTGCTAGCCCATTTTATGTAATCCCTGAATTTGAATAAAATAATGACCCTAGGC
TATAAGGAGTATGAGTTTTCAGAAGGGAAACAAATCTTTTAATGCTATGCAAATGCATTACATGCCAACAATAAAACAATATCAGCAGATATCTCAAAAA
||||||||||||||||||||||||||||||||||||||||||||||||||||||||||||||||||||||||||||||||||||||||||||||||||||
TATAAGGAGTATGAGTTTTCAGAAGGGAAACAAATCTTTTAATGCTATGCAAATGCATTACATGCCAACAATAAAACAATATCAGCAGATATCTCAAAAA
AGTACAAGGGGAAGTACTTAATACCACTAAGCATGCAGTTGAAATGAAAAACCTAAGAACGTTAAGTACATCTACATGTTCAGCGTCACTACATTGTTAG
||||||||||||||||||||||||||||||||||||||||||||||||||||||||||||||||||||||||||||||||||||||||||||||||||||
AGTACAAGGGGAAGTACTTAATACCACTAAGCATGCAGTTGAAATGAAAAACCTAAGAACGTTAAGTACATCTACATGTTCAGCGTCACTACATTGTTAG
GTTCAGAAATATGGTACCTATTATCTCCAAAGAGGTTGTGACCGAAGGAATGCGCTAACTATCGCCCACAATTCACAAGTTTGTTCCAGTATACCACAGC
||||||||||||||||||||||||||||||||||||||||||||||||||||||||||||||||||||||||||||||||||||||||||||||||||||
GTTCAGAAATATGGTACCTATTATCTCCAAAGAGGTTGTGACCGAAGGAATGCGCTAACTATCGCCCACAATTCACAAGTTTGTTCCAGTATACCACAGC
CATGGTAATAATCGGATGGCTTAAGCATGCATGTCGAAATATCTACGGCCCGACTACTCCATAAAGTTCAGGATCTAATCGACAAATATCAATGATGTAA
||||||||||||||||||||||||||||||||||||||||||||||||||||||||||||||||||||||||||||||||||||||||||||||||||||
CATGGTAATAATCGGATGGCTTAAGCATGCATGTCGAAATATCTACGGCCCGACTACTCCATAAAGTTCAGGATCTAATCGACAAATATCAATGATGTAA
CGAGTATACAGGTGACGCACGACATGACGCTAGTATCAAAACTTTTGAAAGGTGAATAAGACAACCCTCATCGCGCAATACAAGTAGTTCGTGCAAGTAG
||||||||||||||||||||||||||||||||||||||||||||||||||||||||||||||||||||||||||||||||||||||||||||||||||||
CGAGTATACAGGTGACGCACGACATGACGCTAGTATCAAAACTTTTGAAAGGTGAATAAGACAACCCTCATCGCGCAATACAAGTAGTTCGTGCAAGTAG
TTCCTGCATAGACACAACTGGTTTCAGGTCTGTAGAGAGCTAGGTTATCACAATAACAGACAAGGCTAAGGCTGTTAATTGGTAGTCCTCCTCTAGTATA
||||||||||||||||||||||||||||||||||||||||||||||||||||||||||||||||||||||||||||||||||||||||||||||||||||
TTCCTGCATAGACACAACTGGTTTCAGGTCTGTAGAGAGCTAGGTTATCACAATAACAGACAAGGCTAAGGCTGTTAATTGGTAGTCCTCCTCTAGTATA
TGTCGGCAGCGGATCCATTGGTTGTGCATGTTATACAAGATGTGCTTGATCCATTTACATCAACTGTTCCACTCAGGATAGCCTACAACAACAGGCTAGT
||||||||||||||||||||||||||||||||||||||||||||||||||||||||||||||||||||||||||||||||||||||||||||||||||||
TGTCGGCAGCGGATCCATTGGTTGTGCATGTTATACAAGATGTGCTTGATCCATTTACATCAACTGTTCCACTCAGGATAGCCTACAACAACAGGCTAGT
TCTGGCAGGTGCTGAGCTAAGACCATCTGCAATTGTAAGCAAGCCGCGAGTTGATATCGGTGGCAGTGACATGAGAGTTCTCTACACCCTGGTAAGCTTC
||||||||||||||||||||||||||||||||||||||||||||||||||||||||||||||||||||||||||||||||||||||||||||||||||||
TCTGGCAGGTGCTGAGCTAAGACCATCTGCAATTGTAAGCAAGCCGCGAGTTGATATCGGTGGCAGTGACATGAGAGTTCTCTACACCCTGGTAAGCTTC
TAACTCTAGTAGTGGACAATATATGCAAATACTTTGGTTATTTCACTCTTTTACGTGGGTTGTTAGTATAATTGTGAAGGAATTCCATAGAAGGAAAAGG
||||||||||||||||||||||||||||||||||||||||||||||||||||||||||||||||||||||||||||||||||||||||||||||||||||
TAACTCTAGTAGTGGACAATATATGCAAATACTTTGGTTATTTCACTCTTTTACGTGGGTTGTTAGTATAATTGTGAAGGAATTCCATAGAAGGAAAAGG
TCTCATAGATGATTAATTTACTCCTAGCAAACTCAAACGAATCATATGTTAACTGGTTTGAGTTTGTCTTTGACTTCAAAAGATATTGGTGGATCCAGAC
||||||||||||||||||||||||||||||||||||||||||||||||||||||||||||||||||||||||||||||||||||||||||||||||||||
TCTCATAGATGATTAATTTACTCCTAGCAAACTCAAACGAATCATATGTTAACTGGTTTGAGTTTGTCTTTGACTTCAAAAGATATTGGTGGATCCAGAC
GCCCCAAGCCCAAGTCACCCATCACTAAGGGAGTACTTGCACTGGTAAATCAAATGCAAACATGTTCTTATTCTCACCAATACGATTCCAAACCCTCGAC
||||||||||||||||||||||||||||||||||||||||||||||||||||||||||||||||||||||||||||||||||||||||||||||||||||
GCCCCAAGCCCAAGTCACCCATCACTAAGGGAGTACTTGCACTGGTAAATCAAATGCAAACATGTTCTTATTCTCACCAATACGATTCCAAACCCTCGAC
CCATCTGTTTTCTGCCATGAAACTTTAGATGTATGTTCCATACTTAATTTGCTCTAATTATGATATATCTCCACTCCTGTGTAGGATGGTGTCAGACATC
||||||||||||||||||||||||||||||||||||||||||||||||||||||||||||||||||||||||||||||||||||||||||||||||||||
CCATCTGTTTTCTGCCATGAAACTTTAGATGTATGTTCCATACTTAATTTGCTCTAATTATGATATATCTCCACTCCTGTGTAGGATGGTGTCAGACATC
CCTGGAACAACTGGTGCCAGCTTCGGTATGATGCCATGCATAACAATTTGTTCAATGTGATTTTTTGTTTTAGCTGAATTTTGTGGACGCAGAAATCAGG
||||||||||||||||||||||||||||||||||||||||||||||||||||||||||||||||||||||||||||||||||||||||||||||||||||
CCTGGAACAACTGGTGCCAGCTTCGGTATGATGCCATGCATAACAATTTGTTCAATGTGATTTTTTGTTTTAGCTGAATTTTGTGGACGCAGAAATCAGG
CCATGTTAAGTTCTTTCCCTCAAACGACTCGATACAAGTTAATTCCAATATAGCCCATCGTTAACACCAAGACATCTTGAACCATGACAAAGAATAACAA
||||||||||||||||||||||||||||||||||||||||||||||||||||||||||||||||||||||||||||||||||||||||||||||||||||
CCATGTTAAGTTCTTTCCCTCAAACGACTCGATACAAGTTAATTCCAATATAGCCCATCGTTAACACCAAGACATCTTGAACCATGACAAAGAATAACAA
TAAACAGCGTAAAATGACATTAGTATGTTTTGCAAATGAAATGGAAAACATTAAGACAAGGCCACTGTACAGCCTAAAGAAGCAAGAACTCTTAAAACAG
||||||||||||||||||||||||||||||||||||||||||||||||||||||||||||||||||||||||||||||||||||||||||||||||||||
TAAACAGCGTAAAATGACATTAGTATGTTTTGCAAATGAAATGGAAAACATTAAGACAAGGCCACTGTACAGCCTAAAGAAGCAAGAACTCTTAAAACAG
TGGTATACTTAAGAATTTCCTTTTTCAATCTACTATTTATACTTTATTTCCACATGTACAAACACAAAATCCATGCAATCAATTAATATCATTCAATCTT
||||||||||||||||||||||||||||||||||||||||||||||||||||||||||||||||||||||||||||||||||||||||||||||||||||
TGGTATACTTAAGAATTTCCTTTTTCAATCTACTATTTATACTTTATTTCCACATGTACAAACACAAAATCCATGCAATCAATTAATATCATTCAATCTT
ATACTGGTTTTGAAGACTTTTATGATATATTGTGCTAGCAACTAACATATACAGCAGAACAAAGGTTACCCCTATACATGAAGATTATATTAGAATTACC
||||||||||||||||||||||||||||||||||||||||||||||||||||||||||||||||||||||||||||||||||||||||||||||||||||
ATACTGGTTTTGAAGACTTTTATGATATATTGTGCTAGCAACTAACATATACAGCAGAACAAAGGTTACCCCTATACATGAAGATTATATTAGAATTACC
TAGAACAAACAAAGTAGACTACGTTGCAGATAAATGACTAGATTGGTATGTTTCCACCAAGTTGAATTACTCTTTAAATTCTTAACTGGTGCTCTTGCAG
||||||||||||||||||||||||||||||||||||||||||||||||||||||||||||||||||||||||||||||||||||||||||||||||||||
TAGAACAAACAAAGTAGACTACGTTGCAGATAAATGACTAGATTGGTATGTTTCCACCAAGTTGAATTACTCTTTAAATTCTTAACTGGTGCTCTTGCAG
GCCAAGAGCTTTTAGTTTATGAAAGGCCAGAACCAAGATCTGGTATCCACCGGATGGTATTTGTGCTGTTCCAGCAACTAGGCAGGGGTACAGTTTTTGC
||||||||||||||||||||||||||||||||||||||||||||||||||||||||||||||||||||||||||||||||||||||||||||||||||||
GCCAAGAGCTTTTAGTTTATGAAAGGCCAGAACCAAGATCTGGTATCCACCGGATGGTATTTGTGCTGTTCCAGCAACTAGGCAGGGGTACAGTTTTTGC
ACCAGATGTGCGACACAACTTCAGCTGCAGGAACTTTGCACGACAGCACCACCTCAACATTGTGGCTGCCTCATATTTCAACTGTCAAAGGGAAGGTGGA
||||||||||||||||||||||||||||||||||||||||||||||||||||||||||||||||||||||||||||||||||||||||||||||||||||
ACCAGATGTGCGACACAACTTCAGCTGCAGGAACTTTGCACGACAGCACCACCTCAACATTGTGGCTGCCTCATATTTCAACTGTCAAAGGGAAGGTGGA
TCAGGCGGAAGAAGGTTTAGGCCAGAAAGTTCTCAAGGGGAGTAGGGACTAGATAGTACAGAGTACTGACCATTGTATGCGGTACTGTAGTGCTGCATCA
|||||||||||||||||||| |||||||||||||||||||||||||||||||||||||||||||||||||||||||||||||||||||||||||||||||
TCAGGCGGAAGAAGGTTTAGACCAGAAAGTTCTCAAGGGGAGTAGGGACTAGATAGTACAGAGTACTGACCATTGTATGCGGTACTGTAGTGCTGCATCA
CAAATAATGTGCAGTACATAGAATGTCTTGTGTCACCTAAATATGCAGCATTATATGCGTCTTCAGCTATGATGAGCTTGATGGGATATATCTCAGTCAT
||||||||||||||||||||||||||||||||||||||||||||||||||||||||||||||||||||||||||||||||||||||||||||||||||||
CAAATAATGTGCAGTACATAGAATGTCTTGTGTCACCTAAATATGCAGCATTATATGCGTCTTCAGCTATGATGAGCTTGATGGGATATATCTCAGTCAT
TAGTTAATCATCTGATCATGGAAAGTATTCTTGAGGGAATACAAAAGATAAACTCAGGCTTTTACAATGTTAGTTAAGCTCAACTAGTTTATAGTTCTGA
||||||||||||||||||||||||||||||||||||||||||||||||||||||||||||||||||||||||||||||||||||||||||||||||||||
TAGTTAATCATCTGATCATGGAAAGTATTCTTGAGGGAATACAAAAGATAAACTCAGGCTTTTACAATGTTAGTTAAGCTCAACTAGTTTATAGTTCTGA
GCACCTGCTTTGCATTTCAACAAGTTCTATTGAATAAATCAACACACTGTAAATCATCAAAGTTATTTTTGAAAGAAATCCCAAGATATATTTTAGACGA
||||||||||||||||||||||||||||||||||||||||||||||||||||||||||||||||||||||||||||||||||||||||||||||||||||
GCACCTGCTTTGCATTTCAACAAGTTCTATTGAATAAATCAACACACTGTAAATCATCAAAGTTATTTTTGAAAGAAATCCCAAGATATATTTTAGACGA
AAGGCAATATTGGTCAACTTTGTATTAAAACCCACAAGTGGCAAGGTAAGCCCCAAGGTAGGTCCAATGTTCACATTATTCCTTATTCAAAGCAACAGGG
||||||||||||||||||||||||||||||||||||||||||||||||||||||||||||||||||||||||||||||||||||||||||||||||||||
AAGGCAATATTGGTCAACTTTGTATTAAAACCCACAAGTGGCAAGGTAAGCCCCAAGGTAGGTCCAATGTTCACATTATTCCTTATTCAAAGCAACAGGG
GTGCTAGACATTCACATCATTACATCAAACACTTAAAGGTGAAAACATAATATTACAAGACCCCAAGAGCAGTCACAACTTACAGCAGAAGCATATCATC
||||||||||||||||||||||||||||||||||||||||||||||||||||||||||||||||||||||||||||||||||||||||||||||||||||
GTGCTAGACATTCACATCATTACATCAAACACTTAAAGGTGAAAACATAATATTACAAGACCCCAAGAGCAGTCACAACTTACAGCAGAAGCATATCATC
GGGAGGAACCGATCACTTCTTCCCAAGGTGGCATACCTCCACCAGTTCCCAGGACTCCAAGCTTTTATCCTCGGTGTCGGTTCGATAAAGTGCGAGCTCG
||||||||||||||||||||||||||||||||||||||||||||||||||||||||||||||||||||||||||||||||||||||||||||||||||||
GGGAGGAACCGATCACTTCTTCCCAAGGTGGCATACCTCCACCAGTTCCCAGGACTCCAAGCTTTTATCCTCGGTGTCGGTTCGATAAAGTGCGAGCTCG
GAGATCTCGAACTGCAGTCCAGAAATTTCACTGTCCATTTCCTCTACTTTCTTCCTTGCTGCTTCCTTCTCTTCGTCTGTTAGATCCCCATACAAGAGGC
||||||||||||||||||||||||||||||||||||||||||||||||||||||||||||||||||||||||||||||||||||||||||||||||||||
GAGATCTCGAACTGCAGTCCAGAAATTTCACTGTCCATTTCCTCTACTTTCTTCCTTGCTGCTTCCTTCTCTTCGTCTGTTAGATCCCCATACAAGAGGC
TTACATGTGGCATATATGCTGCAATCGTGTAAAAAAACATGAATCGATTGTCATCAACTTGTCAAT
||||||||||||||||||||||||||||||||||||||||||||||||||||||||||||||||||
TTACATGTGGCATATATGCTGCAATCGTGTAAAAAAACATGAATCGATTGTCATCAACTTGTCAAT

***FT-A4 - TRIAE_CS42_2AS_TGACv1_113639_AA0358780***

TAAACACCACATATGTTTTGTCCACTTGTACATGTCTACTCCCTGTCAGAGTTAAATTTAATGAGTATAACAGCACTCAATAATATATATATATATATAT
||||||||||||||||||||||||||||||||||||||||||||||||||||||||||||||||||||||||||||||||||||||||||||||||||||
TAAACACCACATATGTTTTGTCCACTTGTACATGTCTACTCCCTGTCAGAGTTAAATTTAATGAGTATAACAGCACTCAATAATATATATATATATATAT
ATATATATATATATATATATATATATATATATATATATATA----------------------------ACTGATTGTTCGCTGTAAAGAACCAAACAGA
||||||||||||||||||||||||||||||||||||||||| |||||||||||||||||||||||||||||||
ATATATATATATATATATATATATATATATATATATATATATATATATATATATATATATATATATATAACTGATTGTTCGCTGTAAAGAACCAAACAGA
TCGTAACTTGCGTAGAAGTACTATATCAAGCACTGGGGCCTGACACCGAGCTAGCCAAGTAATATATCGCCAATTGCCAATGATACCATGTAAAGTCAGA
||||||||||||||||||||||||||||||||||||||||||||||||||||||||||||||||||||||||||||||||||||||||||||||||||||
TCGTAACTTGCGTAGAAGTACTATATCAAGCACTGGGGCCTGACACCGAGCTAGCCAAGTAATATATCGCCAATTGCCAATGATACCATGTAAAGTCAGA
AGTACAGATCTTCCTTGTGGAATTTGATGCATAGAAGAACAAAAGATATATAATTCCATATAACTGATATGGAAAGGATATATGCGATTCCCAAACATTT
||||||||||||||||||||||||||||||||||||||||||||||||||||||||||||||||||||||||||||||||||||||||||||||||||||
AGTACAGATCTTCCTTGTGGAATTTGATGCATAGAAGAACAAAAGATATATAATTCCATATAACTGATATGGAAAGGATATATGCGATTCCCAAACATTT
GTTACATATGCTCTTATCGCGAGAAGGAGAGATGGCGCTGAGAACATTAGCAAGACGAAGGAATAATGTCTAAAATGCTAACAACTAGAAGGGCACCTTG
||||||||||||||||||||||||||||||||||||||||||||||||||||||||||||||||||||||||||||||||||||||||||||||||||||
GTTACATATGCTCTTATCGCGAGAAGGAGAGATGGCGCTGAGAACATTAGCAAGACGAAGGAATAATGTCTAAAATGCTAACAACTAGAAGGGCACCTTG
TGGAAAGTAGATCGACATAGTGATATTCTAATTATTAGAACGCGAATCACACGTGCATGTGTATGTCTGTCATCTTAGAGCTCTCTCAAAGACTATAAAT
||||||||||||||||||||||||||||||||||||||||||||||||||||||||||||||||||||||||||||||||||||||||||||||||||||
TGGAAAGTAGATCGACATAGTGATATTCTAATTATTAGAACGCGAATCACACGTGCATGTGTATGTCTGTCATCTTAGAGCTCTCTCAAAGACTATAAAT
AGTCCCCAAACCCATGTAATTTCTCAAGCTTTTGTAGTTTGATAGTGTATACACTTGCTCTGAGACTCCAAAATATTATGGCGAATGACTCCTTGACAAG
||||||||||||||||||||||||||||||||||||||||||||||||||||||||||||||||||||||||||||||||||||||||||||||||||||
AGTCCCCAAACCCATGTAATTTCTCAAGCTTTTGTAGTTTGATAGTGTATACACTTGCTCTGAGACTCCAAAATATTATGGCGAATGACTCCTTGACAAG
GGCACAGATAGTTGGAGATGTTCTAGACCCATTTGTTAGCTCGGTGCCTCTAACTGTGATGTATGATGGGAGGCCTGTGTTCAATGGCATGGAGTTTCGC
||||||||||||||||||||||||||||||||||||||||||||||||||||||||||||||||||||||||||||||||||||||||||||||||||||
GGCACAGATAGTTGGAGATGTTCTAGACCCATTTGTTAGCTCGGTGCCTCTAACTGTGATGTATGATGGGAGGCCTGTGTTCAATGGCATGGAGTTTCGC
TCACCAGCAGTCTCTCTGAAACCCAGCGTTGAGATAGGCGGTGATGATTTCCGTGTGGCCTATACTCTAGTAAGCCTACCACTTTGAACCCGTAAGAGTA
||||||||||||||||||||||||||||||||||||||||||||||||||||||||||||||||||||||||||||||||||||||||||||||||||||
TCACCAGCAGTCTCTCTGAAACCCAGCGTTGAGATAGGCGGTGATGATTTCCGTGTGGCCTATACTCTAGTAAGCCTACCACTTTGAACCCGTAAGAGTA
CTCCATTGATGGTAGGCCATACGTGTATGGCCATGGTGTGAACTTGAGAGAGCTTGGGAGACTAATGGCTTCACCAAACAAGCTCGCTGTTGCATGTTGC
||||||||||||||||||||||||||||||||||||||||||||||||||||||||||||||||||||||||||||||||||||||||||||||||||||
CTCCATTGATGGTAGGCCATACGTGTATGGCCATGGTGTGAACTTGAGAGAGCTTGGGAGACTAATGGCTTCACCAAACAAGCTCGCTGTTGCATGTTGC
TTGAACTTGTTTGTTGGTAGCCCATGTTGACCATGTGCTTGTGTAATTTTTATCCCCTAACATGTTGACCATGTGCTTGTGTAATTGTAAATCAAACAGG
||||||||||||||||||||||||||||||||||||||||||||||||||||||||||||||||||||||||||||||||||||||||||||||||||||
TTGAACTTGTTTGTTGGTAGCCCATGTTGACCATGTGCTTGTGTAATTTTTATCCCCTAACATGTTGACCATGTGCTTGTGTAATTGTAAATCAAACAGG
TTATGGTGGATCCTGATGCGCCTAATCCCAGCAACCCAACCCTGAGGGAGTACCTGCACTGGTAAGCCATTACTCTTACATGTTTTGTCATATTTTACCG
||||||||||||||||||||||||||||||||||||||||||||||||||||||||||||||||||||||||||||||||||||||||||||||||||||
TTATGGTGGATCCTGATGCGCCTAATCCCAGCAACCCAACCCTGAGGGAGTACCTGCACTGGTAAGCCATTACTCTTACATGTTTTGTCATATTTTACCG
CATGCACGTTTTTGTTGGGCCACAACTATATGTTGATGGACTGCTCTAATTTTATTTTACTTTGGGTATGTGCAGGATGGTGACTGATGTCCCATCATCA
||||||||||||||||||||||||||||||||||||||||||||||||||||||||||||||||||||||||||||||||||||||||||||||||||||
CATGCACGTTTTTGTTGGGCCACAACTATATGTTGATGGACTGCTCTAATTTTATTTTACTTTGGGTATGTGCAGGATGGTGACTGATGTCCCATCATCA
ACAAACGATAGCTTTGGTGAGTACATCAAAATTAGTTTTCCTAATACTCAGGTTTTCATTCAAACTTCTTGCCAAACGGTTCATGTTAGGTACTAGCTAG
||||||||||||||||||||||||||||||||||||||||||||||||||||||||||||||||||||||||||||||||||||||||||||||||||||
ACAAACGATAGCTTTGGTGAGTACATCAAAATTAGTTTTCCTAATACTCAGGTTTTCATTCAAACTTCTTGCCAAACGGTTCATGTTAGGTACTAGCTAG
TATCTCAGGTGATGTTCCATAGTCACGCCTAAGCTTATACTTAGTGATTAAACTGAGATATTGTATATTTTGATATATGTTTTTCACTTAATATCTTAAT
||||||||||||||||||||||||||||||||||||||||||||||||||||||||||||||||||||||||||||||||||||||||||||||||||||
TATCTCAGGTGATGTTCCATAGTCACGCCTAAGCTTATACTTAGTGATTAAACTGAGATATTGTATATTTTGATATATGTTTTTCACTTAATATCTTAAT
ATGCGATACACCTTAGTTAACAAAGCAGTAGATCTGTATAGTGTGTATAAGACGATGTTAAATCAAAATAAGTATGTGGATTAACTAAAAGTGAGTTCAT
||||||||||||||||||||||||||||||||||||||||||||||||||||||||||||||||||||||||||||||||||||||||||||||||||||
ATGCGATACACCTTAGTTAACAAAGCAGTAGATCTGTATAGTGTGTATAAGACGATGTTAAATCAAAATAAGTATGTGGATTAACTAAAAGTGAGTTCAT
ATGTTTATTATTAACACATAAATTTAATGATTTCATCTTTAGGATGTATGAATTGTATTGCTTTGTGATCATATTTGTAAAAAAAAGTATGATGCAAGGA
||||||||||||||||||||||||||||||||||||||||||||||||||||||||||||||||||||||||||||||||||||||||||||||||||||
ATGTTTATTATTAACACATAAATTTAATGATTTCATCTTTAGGATGTATGAATTGTATTGCTTTGTGATCATATTTGTAAAAAAAAGTATGATGCAAGGA
AATGGTTTCTTAATTATTGCAAAATATTTATCAAACACGGTTAGACATTTCACATCAACTAGTGCAATGTTGCAATGGTTGCATAAGGTACTTGCGAAAT
||||||||||||||||||||||||||||||||||||||||||||||||||||||||||||||||||||||||||||||||||||||||||||||||||||
AATGGTTTCTTAATTATTGCAAAATATTTATCAAACACGGTTAGACATTTCACATCAACTAGTGCAATGTTGCAATGGTTGCATAAGGTACTTGCGAAAT
GCTTCTGAAAAATATATTCTCTGCAAATAATTTGAGAGGTCTTTGTCATGTTGTTCATTTTTTCAGTACTTAATTACTCGTCAATATTGTTTTACACATA
||||||||||||||||||||||||||||||||||||||||||||||||||||||||||||||||||||||||||||||||||||||||||||||||||||
GCTTCTGAAAAATATATTCTCTGCAAATAATTTGAGAGGTCTTTGTCATGTTGTTCATTTTTTCAGTACTTAATTACTCGTCAATATTGTTTTACACATA
TGTATATTTAGTACCATGCGACTTTGCCAAATTAGCATACATTGGTTTTTCTACATGGCAAAACACATAATGTTGCCATATAGCAACTGCCATTTTAATG
||||||||||||||||||||||||||||||||||||||||||||||||||||||||||||||||||||||||||||||||||||||||||||||||||||
TGTATATTTAGTACCATGCGACTTTGCCAAATTAGCATACATTGGTTTTTCTACATGGCAAAACACATAATGTTGCCATATAGCAACTGCCATTTTAATG
CTCAAATAATACAACATTTTATGATAGACCTTACGTACTTGACGTCCATTTTCTGTGATTATAAGTAACCAACTACCAACTCGCCCGTGTTAATTAGGGT
||||||||||||||||||||||||||||||||||||||||||||||||||||||||||||||||||||||||||||||||||||||||||||||||||||
CTCAAATAATACAACATTTTATGATAGACCTTACGTACTTGACGTCCATTTTCTGTGATTATAAGTAACCAACTACCAACTCGCCCGTGTTAATTAGGGT
AGGAAGAGAAAATCTGGCATGTACTTCAATAGTAGCTTCAATCAGTGAACAAAATAATACATGCATGCACGCCCTTGAACCGCTTTATAGTTGCTATTTT
||||||||||||||||||||||||||||||||||||||||||||||||||||||||||||||||||||||||||||||||||||||||||||||||||||
AGGAAGAGAAAATCTGGCATGTACTTCAATAGTAGCTTCAATCAGTGAACAAAATAATACATGCATGCACGCCCTTGAACCGCTTTATAGTTGCTATTTT
CCACATATCCCACCTTACTGCAAAATAGTACAAATTAATGATACCAAGAAAGGACACAAATGCAGTAAAAACCACAAAAGCACATGCTATTTATTCCATT
||||||||||||||||||||||||||||||||||||||||||||||||||||||||||||||||||||||||||||||||||||||||||||||||||||
CCACATATCCCACCTTACTGCAAAATAGTACAAATTAATGATACCAAGAAAGGACACAAATGCAGTAAAAACCACAAAAGCACATGCTATTTATTCCATT
TTCCCGGGAAAGCGCACGCTTAACCTATCTGAATTCCCTTACTCTTTGATGAACTGGCAGGAAAAGAGATCGTGCCATACGAGAGCCCAAGCCCTACCAT
||||||||||||||||||||||||||||||||||||||||||||||||||||||||||||||||||||||||||||||||||||||||||||||||||||
TTCCCGGGAAAGCGCACGCTTAACCTATCTGAATTCCCTTACTCTTTGATGAACTGGCAGGAAAAGAGATCGTGCCATACGAGAGCCCAAGCCCTACCAT
GGGCATCCACCGCATGGTGCTGGTTCTGTACCAGCAGCTGGGGCGGGGGACGGTGTTCGCGCCGCAGGCACGCCAGAGCTTCAACTCGCGCAGCTTCGCG
||||||||||||||||||||||||||||||||||||||||||||||||||||||||||||||||||||||||||||||||||||||||||||||||||||
GGGCATCCACCGCATGGTGCTGGTTCTGTACCAGCAGCTGGGGCGGGGGACGGTGTTCGCGCCGCAGGCACGCCAGAGCTTCAACTCGCGCAGCTTCGCG
CGCCGCTTCAACCTCGGCAAGCCCGTCGCTGCTGTGTACTTCAACTGCCAGCGTCCCACGGGCACCGGTGGGAGGAGGTTCACCTGATGTGTCAAACTGA
||||||||||||||||||||||||||||||||||||||||||||||||||||||||||||||||||||||||||||||||||||||||||||||||||||
CGCCGCTTCAACCTCGGCAAGCCCGTCGCTGCTGTGTACTTCAACTGCCAGCGTCCCACGGGCACCGGTGGGAGGAGGTTCACCTGATGTGTCAAACTGA
TCGACCAGCAATGATCCTCAGCCCAGCCGACGGCAATGCTTGAGCTCACCGGTACCTGATACTGTGATAAAGATATCTAGAGAAGTTGGAATAAATGTAC
||||||||||||||||||||||||||||||||||||||||||||||||||||||||||||||||||||||||||||||||||||||||||||||||||||
TCGACCAGCAATGATCCTCAGCCCAGCCGACGGCAATGCTTGAGCTCACCGGTACCTGATACTGTGATAAAGATATCTAGAGAAGTTGGAATAAATGTAC
TGGAAGGTTTATGTCTGATCTATCTAGCTGGTTGATCAGCCATGGCCCAGCTCGAAGGCAGTAAGTTAAGCTCGAGCTTTGCCGGTACTTATGTCTGCGT
||||||||||||||||||||||||||||||||||||||||||||||||||||||||||||||||||||||||||||||||||||||||||||||||||||
TGGAAGGTTTATGTCTGATCTATCTAGCTGGTTGATCAGCCATGGCCCAGCTCGAAGGCAGTAAGTTAAGCTCGAGCTTTGCCGGTACTTATGTCTGCGT
GCTTACTAATACGTACACACATGATAGCCGCATCACACGGTACTTAGCTAGACGAGTTGGAATAAAGTACCTGTGTTTGTACTTGTAGACTCTTAAGTAA
||||||||||||||||||||||||||||||||||||||||||||||||||||||||||||||||||||||||||||||||||||||||||||||||||||
GCTTACTAATACGTACACACATGATAGCCGCATCACACGGTACTTAGCTAGACGAGTTGGAATAAAGTACCTGTGTTTGTACTTGTAGACTCTTAAGTAA
AGTGCCTTTGCAAGGATGCGTTGCTACTTATCAATAAGGAATGTGACATTATGCCACACCTTGGTAATGAGATATGTGCGGTTGGTTGTTCTGATCTTCA
||||||||||||||||||||||||||||||||||||||||||||||||||||||||||||||||||||||||||||||||||||||||||||||||||||
AGTGCCTTTGCAAGGATGCGTTGCTACTTATCAATAAGGAATGTGACATTATGCCACACCTTGGTAATGAGATATGTGCGGTTGGTTGTTCTGATCTTCA
GTTTGAGCAAAATTATTTTGTATTTTTGTGAGTTAGATTAGAACAACCATGAGCGTAATGTTGGCCGGCATAGAGATCAAAGTGATGACACGTGCACAAT
||||||||||||||||||||||||||||||||||||||||||||||||||||||||||||||||||||||||||||||||||||||||||||||||||||
GTTTGAGCAAAATTATTTTGTATTTTTGTGAGTTAGATTAGAACAACCATGAGCGTAATGTTGGCCGGCATAGAGATCAAAGTGATGACACGTGCACAAT
ATACCTCTATCATGCCACAAAAAATTGAAATCAAATTTTCGTCCAAAGATATAGAACTGCGAATTGTCCACACCGAATTTGAACTTGGTACATACAATCT
||||||||||||||||||||||||||||||||||||||||||||||||||||||||||||||||||||||||||||||||||||||||||||||||||||
ATACCTCTATCATGCCACAAAAAATTGAAATCAAATTTTCGTCCAAAGATATAGAACTGCGAATTGTCCACACCGAATTTGAACTTGGTACATACAATCT
ACCAATACATTTTTCCTTATTTGTTCTCATGGTGTGCATTGAATTTATTTTTGGACTCATGGCAAAACTTCTCGAAGACTCATGTCACGAGATGTAGTTT
||||||||||||||||||||||||||||||||||||||||||||||||||||||||||||||||||||||||||||||||||||||||||||||||||||
ACCAATACATTTTTCCTTATTTGTTCTCATGGTGTGCATTGAATTTATTTTTGGACTCATGGCAAAACTTCTCGAAGACTCATGTCACGAGATGTAGTTT
GGCGGATGCAGCTTGTAGGACACCGGTGGGCTACAAGTCGATGGGTTGGGGATCCCAAGGTAGATTTCGGTAAAGCAAAAACAGTCGTGTTATGAGACTT
||||||||||||||||||||||||||||||||||||||||||||||||||||||||||||||||||||||||||||||||||||||||||||||||||||
GGCGGATGCAGCTTGTAGGACACCGGTGGGCTACAAGTCGATGGGTTGGGGATCCCAAGGTAGATTTCGGTAAAGCAAAAACAGTCGTGTTATGAGACTT
AGGTCGAAACATTTAACAACAAGGTTCCCGGGCGCCCTTAAATTCCTTGGGAGACACCGTTGTGATGTTGTTTTCCTTTC
||||||||||||||||||||||||||||||||||||||||||||||||||||||||||||||||||||||||||||||||
AGGTCGAAACATTTAACAACAAGGTTCCCGGGCGCCCTTAAATTCCTTGGGAGACACCGTTGTGATGTTGTTTTCCTTTC

***FT-B4 - TRIAE_CS42_2BS_TGACv1_147607_AA0485540***

TGCATGCGTGAGTATACAGTTAAGAAGAAAATGATATACTGACAAACACTACTACCGAAAGCAATATTAAACGTGCATAGATAGAAGCAACAAATTGAAA
||||||||||||||||||||||||||||||||||||||||||||||||||||||||||||||||||||||||||||||||||||||||||||||||||||
TGCATGCGTGAGTATACAGTTAAGAAGAAAATGATATACTGACAAACACTACTACCGAAAGCAATATTAAACGTGCATAGATAGAAGCAACAAATTGAAA
AGCCTAAATACCACATATGTTTTGTCCACTGTACATGTCTATTCCCTGTCAGAGTTAAATTTAATGAGTATAACAGCACTCAATAATATATATAACCGGT
||||||||||||||||||||||||||||||||||||||||||||||||||||||||||||||||||||||||||||||||||||||||||||||||||||
AGCCTAAATACCACATATGTTTTGTCCACTGTACATGTCTATTCCCTGTCAGAGTTAAATTTAATGAGTATAACAGCACTCAATAATATATATAACCGGT
TGTTCGCTGTAAAGAACCAAACAGATCGTAACTTGCATAGAAGTACTATACCAAGCACTGGGGCCTGATCGACACCGAGCTAGCAAGCAATATATCGCCA
||||||||||||||||||||||||||||||||||||||||||||||||||||||||||||||||||||||||||||||||||||||||||||||||||||
TGTTCGCTGTAAAGAACCAAACAGATCGTAACTTGCATAGAAGTACTATACCAAGCACTGGGGCCTGATCGACACCGAGCTAGCAAGCAATATATCGCCA
ATTGCCAATGATACCATGTAAAGTCAGAAGTAGAGATCTTCCTCGTGGAATTTGATGCATAGAAGAACAAAAGATATATAATTCCATATAACTGATATGG
||||||||||||||||||||||||||||||||||||||||||||||||||||||||||||||||||||||||||||||||||||||||||||||||||||
ATTGCCAATGATACCATGTAAAGTCAGAAGTAGAGATCTTCCTCGTGGAATTTGATGCATAGAAGAACAAAAGATATATAATTCCATATAACTGATATGG
AAAGGATATATGCGATTCCCAAACATTTGTTACATATGCTCTTATCGCGAGAAGGAGAGATGGCGCTGAGAACCCTAGCAAGACGAAGGAATAATGTCTA
||||||||||||||||||||||||||||||||||||||||||||||||||||||||||||||||||||||||||||||||||||||||||||||||||||
AAAGGATATATGCGATTCCCAAACATTTGTTACATATGCTCTTATCGCGAGAAGGAGAGATGGCGCTGAGAACCCTAGCAAGACGAAGGAATAATGTCTA
AAATGCTAACAAGTAGAAGGGCATCTTGTGGAAAGTAGATCAACATAGTGATATTCTAATTATTAGAACGCGAATCACACGTGCATGTGTGTGTCATCTT
||||||||||||||||||||||||||||||||||||||||||||||||||||||||||||||||||||||||||||||||||||||||||||||||||||
AAATGCTAACAAGTAGAAGGGCATCTTGTGGAAAGTAGATCAACATAGTGATATTCTAATTATTAGAACGCGAATCACACGTGCATGTGTGTGTCATCTT
AGAGCTCTCTCAAAGACTATAAATAGTCCCCAAACCCATGTAATTTCTCAAGCTTTTGTAGTTCAATAGTGTATACACTTGCTCTGAGACTCCAAAATAT
||||||||||||||||||||||||||||||||||||||||||||||||||||||||||||||||||||||||||||||||||||||||||||||||||||
AGAGCTCTCTCAAAGACTATAAATAGTCCCCAAACCCATGTAATTTCTCAAGCTTTTGTAGTTCAATAGTGTATACACTTGCTCTGAGACTCCAAAATAT
TATGGCGAATGACTCCTTGACAAGGGCACAGATAGTTGGAGATGTTCTAGACCCATTTGTTAGCTTGGTGCCTCTAACTGTGATGTATGATGGGAGGCCT
||||||||||||||||||||||||||||||||||||||||||||||||||||||||||||||||||||||||||||||||||||||||||||||||||||
TATGGCGAATGACTCCTTGACAAGGGCACAGATAGTTGGAGATGTTCTAGACCCATTTGTTAGCTTGGTGCCTCTAACTGTGATGTATGATGGGAGGCCT
GTGTTCAATGGCATGGAGTTTCGCTCACCAGCAGTCTCTCTGAAACCCAGCGTTGAGATAGGCGGTGATGATTTTCGTGTGGCCTATACTCTAGTAAGCC
||||||||||||||||||||||||||||||||||||||||||||||||||||||||||||||||||||||||||||||||||||||||||||||||||||
GTGTTCAATGGCATGGAGTTTCGCTCACCAGCAGTCTCTCTGAAACCCAGCGTTGAGATAGGCGGTGATGATTTTCGTGTGGCCTATACTCTAGTAAGCC
TACCACTTTGAACCCGTAAGAGTACTCCATTGATGGAAGGCCATACGTGTATGGCCATGGTGTGAACTTGAGAGAGCTTGGGAGACTAATGGCTTCACCA
||||||||||||||||||||||||||||||||||||||||||||||||||||||||||||||||||||||||||||||||||||||||||||||||||||
TACCACTTTGAACCCGTAAGAGTACTCCATTGATGGAAGGCCATACGTGTATGGCCATGGTGTGAACTTGAGAGAGCTTGGGAGACTAATGGCTTCACCA
AACAAGCTCGTTGTTGCATGTTGCTTGAACTTGTTTGTTGGTAGCCCAATCCAAATTATAGCCAGGTTAATTTTTATCCCCTAACATGTTGACCATGTGC
||||||||||||||||||||||||||||||||||||||||||||||||||||||||||||||||||||||||||||||||||||||||||||||||||||
AACAAGCTCGTTGTTGCATGTTGCTTGAACTTGTTTGTTGGTAGCCCAATCCAAATTATAGCCAGGTTAATTTTTATCCCCTAACATGTTGACCATGTGC
TTGTGTAATTGTGAATCAAATAGGTTATGGTGGATCCTGATGCGCCTAATCCCAGCAACCCAACCCTGAGGGAGTACCTGCACTGGTAAGCCATTACTCT
||||||||||||||||||||||||||||||||||||||||||||||||||||||||||||||||||||||||||||||||||||||||||||||||||||
TTGTGTAATTGTGAATCAAATAGGTTATGGTGGATCCTGATGCGCCTAATCCCAGCAACCCAACCCTGAGGGAGTACCTGCACTGGTAAGCCATTACTCT
TACATGTTTTGTCATATTTTACTGCATGCATGTTTTTGTTGGGCCACAACTATATGTTGATGGACTGCTCTAATTTTATTTTATTTTGGGTATGTGCAGG
||||||||||||||||||||||||||||||||||||||||||||||||||||||||||||||||||||||||||||||||||||||||||||||||||||
TACATGTTTTGTCATATTTTACTGCATGCATGTTTTTGTTGGGCCACAACTATATGTTGATGGACTGCTCTAATTTTATTTTATTTTGGGTATGTGCAGG
ATGGTGACTGATGTCCCATCATCAACAAATGATAGCTTTGGTGAGTACATCAAAATTAGTTTTCCTAATGCTCAGGTTTTCATTCAAACTTCTTGCCAAA
||||||||||||||||||||||||||||||||||||||||||||||||||||||||||||||||||||||||||||||||||||||||||||||||||||
ATGGTGACTGATGTCCCATCATCAACAAATGATAGCTTTGGTGAGTACATCAAAATTAGTTTTCCTAATGCTCAGGTTTTCATTCAAACTTCTTGCCAAA
CGGTCCATATTAGGTACTACTATCTCAGGTGATGTTCCATAGTCACGCTCGAGCTTATACTTAGTGCTTAAACTGGGATATTGTATATTATGATATATGA
||||||||||||||||||||||||||||||||||||||||||||||||||||||||||||||||||||||||||||||||||||||||||||||||||||
CGGTCCATATTAGGTACTACTATCTCAGGTGATGTTCCATAGTCACGCTCGAGCTTATACTTAGTGCTTAAACTGGGATATTGTATATTATGATATATGA
GTTTTCACTTCATACCTTAATATGTGATACACCTTACTGTGTTTTTTTGCGAGGGATGATACACCTTAGTTAACAAAGCAGTAGATCTGTATAGTGTGTA
||||||||||||||||||||||||||||||||||||||||||||||||||||||||||||||||||||||||||||||||||||||||||||||||||||
GTTTTCACTTCATACCTTAATATGTGATACACCTTACTGTGTTTTTTTGCGAGGGATGATACACCTTAGTTAACAAAGCAGTAGATCTGTATAGTGTGTA
TAAGACGATGTTAAATCAAAATATTACAGTATGTGGATTAACTAAAAGTGATTTCATACGTTTATTATTAAAACATAAATTTAATGACTTCATCTTTAGG
||||||||||||||||||||||||||||||||||||||||||||||||||||||||||||||||||||||||||||||||||||||||||||||||||||
TAAGACGATGTTAAATCAAAATATTACAGTATGTGGATTAACTAAAAGTGATTTCATACGTTTATTATTAAAACATAAATTTAATGACTTCATCTTTAGG
ATGTATGAATTGTATTGCTTTGTGATCATATTTGTAAAAAAGTATGATGCAAGGAAAATGGTTTCTTATTTACTGCAAAATATTTATCAAACACGGTTAG
||||||||||||||||||||||||||||||||||||||||||||||||||||||||||||||||||||||||||||||||||||||||||||||||||||
ATGTATGAATTGTATTGCTTTGTGATCATATTTGTAAAAAAGTATGATGCAAGGAAAATGGTTTCTTATTTACTGCAAAATATTTATCAAACACGGTTAG
ACATTTCACATCAATTAGTGTGCAATGTTGCAATGGTTGCATAAGGTACTCGCGAAATGCTTCTGAAAAATATATTCTCTGCAAATAATTTGAGAGGTCT
||||||||||||||||||||||||||||||||||||||||||||||||||||||||||||||||||||||||||||||||||||||||||||||||||||
ACATTTCACATCAATTAGTGTGCAATGTTGCAATGGTTGCATAAGGTACTCGCGAAATGCTTCTGAAAAATATATTCTCTGCAAATAATTTGAGAGGTCT
TTATCATGTTGTTCAATTTTTACTTAATTACTCGTCAATATTGTTTTACACCATGTGACTTTGCCAAATTAGCATACATTGGTTTTTCTACATGGCAAAA
||||||||||||||||||||||||||||||||||||||||||||||||||||||||||||||||||||||||||||||||||||||||||||||||||||
TTATCATGTTGTTCAATTTTTACTTAATTACTCGTCAATATTGTTTTACACCATGTGACTTTGCCAAATTAGCATACATTGGTTTTTCTACATGGCAAAA
CACATAATGTTGCCATATAGCAACTGCCATTTTAACGCTCAAATAATACAACATTTTATGAGTGATTACAAGTAACCAACTACCAAGTCGCCCATGTTAA
||||||||||||||||||||||||||||||||||||||||||||||||||||||||||||||||||||||||||||||||||||||||||||||||||||
CACATAATGTTGCCATATAGCAACTGCCATTTTAACGCTCAAATAATACAACATTTTATGAGTGATTACAAGTAACCAACTACCAAGTCGCCCATGTTAA
TTAGGGTAGGAAGAGCAAAATTCTGGCATATACTTCAATAGTAGCTTCAATCACTGAACAAAATAATACATGTACGCCCTTGAACCGCTTTATAGTTGTT
||||||||||||||||||||||||||||||||||||||||||||||||||||||||||||||||||||||||||||||||||||||||||||||||||||
TTAGGGTAGGAAGAGCAAAATTCTGGCATATACTTCAATAGTAGCTTCAATCACTGAACAAAATAATACATGTACGCCCTTGAACCGCTTTATAGTTGTT
ATTTTCCACATATCCCACCTTGCTGCAAAATAGTACAAATTAATGATACCAAGAAAGGACACAAATGCAGTAAAAACCACAAAAGGACATGCTATTTATT
||||||||||||||||||||||||||||||||||||||||||||||||||||||||||||||||||||||||||||||||||||||||||||||||||||
ATTTTCCACATATCCCACCTTGCTGCAAAATAGTACAAATTAATGATACCAAGAAAGGACACAAATGCAGTAAAAACCACAAAAGGACATGCTATTTATT
CCATTTTCCGGGGAAAGCGCACGCTTAACTTATCTGAATTCCCTTGCTCTTTGATGAACTGGCAGGAAAAGAGATCGTGCCATACGAGAGCCCAAGCCCT
||||||||||||||||||||||||||||||||||||||||||||||||||||||||||||||||||||||||||||||||||||||||||||||||||||
CCATTTTCCGGGGAAAGCGCACGCTTAACTTATCTGAATTCCCTTGCTCTTTGATGAACTGGCAGGAAAAGAGATCGTGCCATACGAGAGCCCAAGCCCT
ACCATGGGCATCCACCGCATGGTGCTGGTTCTGTACCAGCAGTTGGGGCGGGGGACGGTGTTCGCGCCGCAGGCACGCCAGAGCTTCAACTCACGCAGCT
||||||||||||||||||||||||||||||||||||||||||||||||||||||||||||||||||||||||||||||||||||||||||||||||||||
ACCATGGGCATCCACCGCATGGTGCTGGTTCTGTACCAGCAGTTGGGGCGGGGGACGGTGTTCGCGCCGCAGGCACGCCAGAGCTTCAACTCACGCAGCT
TCGCACGCCGCTTCAACCTCGGCAAGCCCGTCGCTGCCGTGTACTTCAACTGCCAGCGTCCCACAGGCACTGGTGGGAGGAGGTTCACCTGATCTGTCTA
||||||||||||||||||||||||||||||||||||||||||||||||||||||||||||||||||||||||||||||||||||||||||||||||||||
TCGCACGCCGCTTCAACCTCGGCAAGCCCGTCGCTGCCGTGTACTTCAACTGCCAGCGTCCCACAGGCACTGGTGGGAGGAGGTTCACCTGATCTGTCTA
ACTGATCGATCAGCCATGATCCTCGTCGCCGCCTGAGCTCACCGGTACCTGATACAGATATCTAGAGAAGTTGGAATAAATGTACTGGAAGGTTCATGTC
||||||||||||||||||||||||||||||||||||||||||||||||||||||||||||||||||||||||||||||||||||||||||||||||||||
ACTGATCGATCAGCCATGATCCTCGTCGCCGCCTGAGCTCACCGGTACCTGATACAGATATCTAGAGAAGTTGGAATAAATGTACTGGAAGGTTCATGTC
TGATCTATCTAACTGGTTGATCAGCCATGGCCCAGCTCGAAGGCAGTTAGTTAAGCTCGAGCTCTGCCGGTACTTATGTCTGCGTGCTTACTAATACGTA
||||||||||||||||||||||||||||||||||||||||||||||||||||||||||||||||||||||||||||||||||||||||||||||||||||
TGATCTATCTAACTGGTTGATCAGCCATGGCCCAGCTCGAAGGCAGTTAGTTAAGCTCGAGCTCTGCCGGTACTTATGTCTGCGTGCTTACTAATACGTA
CACCCATGATAGCCGCATCACACGGTACTTAGCTAGAAGAGTTGGAATAAAGTACCTGTGTTTGTACTTGTAGGCTCTTAAGTAAGGAGCCTTTGCAAGG
||||||||||||||||||||||||||||||||||||||||||||||||||||||||||||||||||||||||||||||||||||||||||||||||||||
CACCCATGATAGCCGCATCACACGGTACTTAGCTAGAAGAGTTGGAATAAAGTACCTGTGTTTGTACTTGTAGGCTCTTAAGTAAGGAGCCTTTGCAAGG
ATGCGTTGCTACTGTTCAATAAGGAATGTGACATTATGCCACACCTTGGTAATGAGATATGTGCGGTTGGTTGTTCTGATCTTCAGGTTGAGCAAAATTA
||||||||||||||||||||||||||||||||||||||||||||||||||||||||||||||||||||||||||||||||||||||||||||||||||||
ATGCGTTGCTACTGTTCAATAAGGAATGTGACATTATGCCACACCTTGGTAATGAGATATGTGCGGTTGGTTGTTCTGATCTTCAGGTTGAGCAAAATTA
TTTTGTATTTTTGTGAGTTAGATTAGAACAATCACAAGCGTAATGTTGGCTCATATAGAGATCAAAGTGATGGCACATGCACTATATACCTCTATCGTGC
||||||||||||||||||||||||||||||||||||||||||||||||||||||||||||||||||||||||||||||||||||||||||||||||||||
TTTTGTATTTTTGTGAGTTAGATTAGAACAATCACAAGCGTAATGTTGGCTCATATAGAGATCAAAGTGATGGCACATGCACTATATACCTCTATCGTGC
CACAAAAAATTGAAATCAAATTTTGGTCCAAAGATATAGAACTTCGAATTGTTCGCACCAAATTTGAACTTGGTACGTACGATCTACCAGTACATTTTTC
||||||||||||||||||||||||||||||||||||||||||||||||||||||||||||||||||||||||||||||||||||||||||||||||||||
CACAAAAAATTGAAATCAAATTTTGGTCCAAAGATATAGAACTTCGAATTGTTCGCACCAAATTTGAACTTGGTACGTACGATCTACCAGTACATTTTTC
CTCAATTGTTTCCCATGGTGTGCATTGAATTTATTTCTGGACTCATGCAAACTTCTCGAAGAATCGTGCCTCGAGATGTAGTTTGGTGGATGCGGCTGTA
||||||||||||||||||||||||||||||||||||||||||||||||||||||||||||||||||||||||||||||||||||||||||||||||||||
CTCAATTGTTTCCCATGGTGTGCATTGAATTTATTTCTGGACTCATGCAAACTTCTCGAAGAATCGTGCCTCGAGATGTAGTTTGGTGGATGCGGCTGTA
GAATACCGGTGGGCTGCATGTCGACGGGTTGGGGATCCCAAGGTAGATTTCAGTAAAAACAAACATTTTTATCGTGTTATGGGACAGGTTGAAACATTTA
||||||||||||||||||||||||||||||||||||||||||||||||||||||||||||||||||||||||||||||||||||||||||||||||||||
GAATACCGGTGGGCTGCATGTCGACGGGTTGGGGATCCCAAGGTAGATTTCAGTAAAAACAAACATTTTTATCGTGTTATGGGACAGGTTGAAACATTTA
ACAACAAGATTCCCATGGGCGCCCTTAAATTCCTTGGGAGCCTCTTTTGTGATGTTGTTTTCCCTTCTTTGATTGGACGATGGTTGTGCTTCATGCATCA
||||||||||||||||||||||||||||||||||||||||||||||||||||||||||||||||||||||||||||||||||||||||||||||||||||
ACAACAAGATTCCCATGGGCGCCCTTAAATTCCTTGGGAGCCTCTTTTGTGATGTTGTTTTCCCTTCTTTGATTGGACGATGGTTGTGCTTCATGCATCA
TTCCCTTCATGGGGACATTGTCTTGGAGGTTGGAGACGATGTAGTTCTGACACCGGTGGCTGGCGGCTTGACCGCAACTCGGGTC
|||||||||||||||||||||||||||||||||||||||||||||||||||||||||||||||||||||||||||||||||||||
TTCCCTTCATGGGGACATTGTCTTGGAGGTTGGAGACGATGTAGTTCTGACACCGGTGGCTGGCGGCTTGACCGCAACTCGGGTC

***FT-D4 - TRIAE_CS42_2DS_TGACv1_178082_AA0590500***

CCACCGAGAGCAATATTAAACATGCATAGATAAAAGCAACAAATTGAAAGGCCTAAATATCACATATGTTTTGTCCATTTGTACATGTCTACTCCCTGTC
||||||||||||||||||||||||||||||||||||||||||||||||||||||||||||||||||||||||||||||||||||||||||||||||||||
CCACCGAGAGCAATATTAAACATGCATAGATAAAAGCAACAAATTGAAAGGCCTAAATATCACATATGTTTTGTCCATTTGTACATGTCTACTCCCTGTC
AGAGTTAAATTTAATAAGTATAACAGCACTCAATAATATATATAACCGGTTGTTCGCTGTAAAGAACCAAACAGATCGTAACTTACGTATAAGTACTATA
||||||||||||||||||||||||||||||||||||||||||||||||||||||||||||||||||||||||||||||||||||||||||||||||||||
AGAGTTAAATTTAATAAGTATAACAGCACTCAATAATATATATAACCGGTTGTTCGCTGTAAAGAACCAAACAGATCGTAACTTACGTATAAGTACTATA
TCAAGCACCGGGGCCTGATCGACACCGAGCTAGCCAAGTAATATATCGTCAATTGCCAATGATACCATGTAAAGTCAGAAGTAGAGATCTCCCTCGTGGA
||||||||||||||||||||||||||||||||||||||||||||||||||||||||||||||||||||||||||||||||||||||||||||||||||||
TCAAGCACCGGGGCCTGATCGACACCGAGCTAGCCAAGTAATATATCGTCAATTGCCAATGATACCATGTAAAGTCAGAAGTAGAGATCTCCCTCGTGGA
ATGTGATGCATAGAAGAACAAAAGATATATAATTCCATATAACTGATATGGAAAGGATATATGCGATTCCCAAACACTTGTTACATATGCTCTTATCGCG
||||||||||||||||||||||||||||||||||||||||||||||||||||||||||||||||||||||||||||||||||||||||||||||||||||
ATGTGATGCATAGAAGAACAAAAGATATATAATTCCATATAACTGATATGGAAAGGATATATGCGATTCCCAAACACTTGTTACATATGCTCTTATCGCG
AGAAGGAGAGATGGCGCTGAGAACACTAGCAAGACGAAGGAATAATGTCTAAAATGCTGACAAGTAGAAGGGCATCTTGTCGAAAGTAGATCAACATAGT
||||||||||||||||||||||||||||||||||||||||||||||||||||||||||||||||||||||||||||||||||||||||||||||||||||
AGAAGGAGAGATGGCGCTGAGAACACTAGCAAGACGAAGGAATAATGTCTAAAATGCTGACAAGTAGAAGGGCATCTTGTCGAAAGTAGATCAACATAGT
GATATTCTAATTATTAGAACGCGAGTCACACGTGCATGTGTGTGTCATCTTAGAGCTCTCTCAAAGACTATAAATAGTCCCCAACCCCATGTAATTTCTC
||||||||||||||||||||||||||||||||||||||||||||||||||||||||||||||||||||||||||||||||||||||||||||||||||||
GATATTCTAATTATTAGAACGCGAGTCACACGTGCATGTGTGTGTCATCTTAGAGCTCTCTCAAAGACTATAAATAGTCCCCAACCCCATGTAATTTCTC
AAGCTTTTGTAGTTCAATAGTGTATACACTTGCTCTGAGACTCCAAAATATTATGGCGAATGACTCCTTGACAAGGGCACAGATAGTTGGAGATGTTCTA
||||||||||||||||||||||||||||||||||||||||||||||||||||||||||||||||||||||||||||||||||||||||||||||||||||
AAGCTTTTGTAGTTCAATAGTGTATACACTTGCTCTGAGACTCCAAAATATTATGGCGAATGACTCCTTGACAAGGGCACAGATAGTTGGAGATGTTCTA
GACCCATTTGTTAGCTCGGTGCCTCTAACTGTGATGTATGATGGGAGGCCTGTGTTCAATGGCATGGAGTTTCGCTCGCCAGCAGTCTCTCTGAAACCCA
||||||||||||||||||||||||||||||||||||||||||||||||||||||||||||||||||||||||||||||||||||||||||||||||||||
GACCCATTTGTTAGCTCGGTGCCTCTAACTGTGATGTATGATGGGAGGCCTGTGTTCAATGGCATGGAGTTTCGCTCGCCAGCAGTCTCTCTGAAACCCA
ACGTTGACATAGGCGGTGATGATTTTCGTGTGGCCTATACTCTAGTAAGCCTACCACTTTGAACCCGAAAGAGTACTCCATTGATGGTAGGCCATACGTG
||||||||||||||||||||||||||||||||||||||||||||||||||||||||||||||||||||||||||||||||||||||||||||||||||||
ACGTTGACATAGGCGGTGATGATTTTCGTGTGGCCTATACTCTAGTAAGCCTACCACTTTGAACCCGAAAGAGTACTCCATTGATGGTAGGCCATACGTG
TATGGCCATAGTATGAACTTGAGAGAGTTTGGGAGACTAATGGCTTCACCAAACAAGCTCGTTGTTGCATGTTGCTTGAACTTGTTTGTTGGTAGCCCAA
||||||||||||||||||||||||||||||||||||||||||||||||||||||||||||||||||||||||||||||||||||||||||||||||||||
TATGGCCATAGTATGAACTTGAGAGAGTTTGGGAGACTAATGGCTTCACCAAACAAGCTCGTTGTTGCATGTTGCTTGAACTTGTTTGTTGGTAGCCCAA
TCCAAATTATAGCTAGGTTATTTTTTATCCCCTAACATGTTGACCATGTGCTTGTGTAATTGTAAATCAAATAGGTTATGGTGGATCCTGATGCGCCTAA
||||||||||||||||||||||||||||||||||||||||||||||||||||||||||||||||||||||||||||||||||||||||||||||||||||
TCCAAATTATAGCTAGGTTATTTTTTATCCCCTAACATGTTGACCATGTGCTTGTGTAATTGTAAATCAAATAGGTTATGGTGGATCCTGATGCGCCTAA
TCCCAGCAACCCAACCCTGAGGGAGTACCTGCACTGGTAAGCCATTACTCTTACATGTGTTGTCATATTTTACCGCATGCACGTTTTTGTTGGGCCACAA
||||||||||||||||||||||||||||||||||||||||||||||||||||||||||||||||||||||||||||||||||||||||||||||||||||
TCCCAGCAACCCAACCCTGAGGGAGTACCTGCACTGGTAAGCCATTACTCTTACATGTGTTGTCATATTTTACCGCATGCACGTTTTTGTTGGGCCACAA
CTATATGTTGATGGACTGCTCTAATTTTATTTTATTTTGGGTATGCGCAGGATGGTGACTGATATCCCATCATCAACAAACGATAGCTTTGGTGAGTACA
||||||||||||||||||||||||||||||||||||||||||||||||||||||||||||||||||||||||||||||||||||||||||||||||||||
CTATATGTTGATGGACTGCTCTAATTTTATTTTATTTTGGGTATGCGCAGGATGGTGACTGATATCCCATCATCAACAAACGATAGCTTTGGTGAGTACA
TCAAAATTACTTTTCCTAATGCTCAGGTTTTCATTCAAGCTTCTTGCCAAACGGTCCATATTAGGTACTAGTATCTCAGGTGGTGTTCCATAGTGATGCT
||||||||||||||||||||||||||||||||||||||||||||||||||||||||||||||||||||||||||||||||||||||||||||||||||||
TCAAAATTACTTTTCCTAATGCTCAGGTTTTCATTCAAGCTTCTTGCCAAACGGTCCATATTAGGTACTAGTATCTCAGGTGGTGTTCCATAGTGATGCT
CGAGCTTATACTTAGTGCTTAAACTGGGATATTGTATATTATGATATATGATTTTTTTACTTCTCATCTTAATATGTGATACACCTTAGTTAACAAAGCA
||||||||||||||||||||||||||||||||||||||||||||||||||||||||||||||||||||||||||||||||||||||||||||||||||||
CGAGCTTATACTTAGTGCTTAAACTGGGATATTGTATATTATGATATATGATTTTTTTACTTCTCATCTTAATATGTGATACACCTTAGTTAACAAAGCA
GTAGATCTGTACAGTGTGTATCAGATGATGTTAAATCAAAATATTGCAATATGTGGCTTAACTAAAAGTGAGTTCAAACGCTTATTATTAAAACATAAAT
||||||||||||||||||||||||||||||||||||||||||||||||||||||||||||||||||||||||||||||||||||||||||||||||||||
GTAGATCTGTACAGTGTGTATCAGATGATGTTAAATCAAAATATTGCAATATGTGGCTTAACTAAAAGTGAGTTCAAACGCTTATTATTAAAACATAAAT
TTAATGATTTCATCTTTAGGATGTATGAATTGTATTGCTTTGTGATCATATTTGTAAAAGTATGATGCAAGGAAAATGGTTTCTTATTTACTGCAAAATA
||||||||||||||||||||||||||||||||||||||||||||||||||||||||||||||||||||||||||||||||||||||||||||||||||||
TTAATGATTTCATCTTTAGGATGTATGAATTGTATTGCTTTGTGATCATATTTGTAAAAGTATGATGCAAGGAAAATGGTTTCTTATTTACTGCAAAATA
TTTATCACATCAACTAGTGCAATGTTGCAATGGTTGCATAAGGTACTCGTGAAATGCTGCTGAAAAATATATTCTCTGCAAATAATTTGAGAGGTCTTTG
||||||||||||||||||||||||||||||||||||||||||||||||||||||||||||||||||||||||||||||||||||||||||||||||||||
TTTATCACATCAACTAGTGCAATGTTGCAATGGTTGCATAAGGTACTCGTGAAATGCTGCTGAAAAATATATTCTCTGCAAATAATTTGAGAGGTCTTTG
TCATGTTGTTCAATTTTTCAGTACTTAATTACTCGTCAATATTGTTTTACACATATGTATATTCAGTACCATGTGACTTTGCCAAATTAGCTTACATTGG
||||||||||||||||||||||||||||||||||||||||||||||||||||||||||||||||||||||||||||||||||||||||||||||||||||
TCATGTTGTTCAATTTTTCAGTACTTAATTACTCGTCAATATTGTTTTACACATATGTATATTCAGTACCATGTGACTTTGCCAAATTAGCTTACATTGG
TTTTTCTACATGGCAAAACACACAATGTTGCAATATAGCAACTGCCATTTTAATGCTCAAATAATACAACATTTTATGATAGACCTTACGTACTTGACGT
||||||||||||||||||||||||||||||||||||||||||||||||||||||||||||||||||||||||||||||||||||||||||||||||||||
TTTTTCTACATGGCAAAACACACAATGTTGCAATATAGCAACTGCCATTTTAATGCTCAAATAATACAACATTTTATGATAGACCTTACGTACTTGACGT
TCATTTTCTGTGATTACAAGTAACCAACTACCAACTCGCCCATGTTAATTAGGGTAGGAAGAGAAAAAATCTGGCATGTACTTCAATCAGTGAACAAAAT
||||||||||||||||||||||||||||||||||||||||||||||||||||||||||||||||||||||||||||||||||||||||||||||||||||
TCATTTTCTGTGATTACAAGTAACCAACTACCAACTCGCCCATGTTAATTAGGGTAGGAAGAGAAAAAATCTGGCATGTACTTCAATCAGTGAACAAAAT
AATACATGTACGCCCTTGAACCGCTTTATAGTTGCTATTTTCCACATATCCCACCTTACTGCAAAATAGTACTACAAATTAATGATGCCAAGAAAGGACA
||||||||||||||||||||||||||||||||||||||||||||||||||||||||||||||||||||||||||||||||||||||||||||||||||||
AATACATGTACGCCCTTGAACCGCTTTATAGTTGCTATTTTCCACATATCCCACCTTACTGCAAAATAGTACTACAAATTAATGATGCCAAGAAAGGACA
CAAATACAGTAAAACCCACAAAAGCACATGCTATTTATTCCATTTTCCGGGGAAAGCGCACGCTTAACAGTTAACTTATCTGAATTCCCTTACTCTTTGA
||||||||||||||||||||||||||||||||||||||||||||||||||||||||||||||||||||||||||||||||||||||||||||||||||||
CAAATACAGTAAAACCCACAAAAGCACATGCTATTTATTCCATTTTCCGGGGAAAGCGCACGCTTAACAGTTAACTTATCTGAATTCCCTTACTCTTTGA
TGAACTGGCAGGAAAAGAGGTCGTGCCATACGAGAGCCCAAGCCCTACCATGGGCATCCACCGCATGGTGCTGGTTCTGTACCAGCAGCTGGGGCGGGGG
||||||||||||||||||||||||||||||||||||||||||||||||||||||||||||||||||||||||||||||||||||||||||||||||||||
TGAACTGGCAGGAAAAGAGGTCGTGCCATACGAGAGCCCAAGCCCTACCATGGGCATCCACCGCATGGTGCTGGTTCTGTACCAGCAGCTGGGGCGGGGG
ACGGTGTTCGCGCCGCAGGCACGCCAGAGCTTCAACTCGCGCAGCTTCGCACGCCGCTTCAACCTCGGCAAGCCCGTCGCTGCCGTGTACTTCAACTGCC
||||||||||||||||||||||||||||||||||||||||||||||||||||||||||||||||||||||||||||||||||||||||||||||||||||
ACGGTGTTCGCGCCGCAGGCACGCCAGAGCTTCAACTCGCGCAGCTTCGCACGCCGCTTCAACCTCGGCAAGCCCGTCGCTGCCGTGTACTTCAACTGCC
AGCGTCCCACGGGCACCGGTGGGAGGAGGTTCACCTGATCTGTCAAACTGATCGACCAGCAATGATCCTCGGCCCAGCCGACGGCAATGCTTGAGCTCAC
||||||||||||||||||||||||||||||||||||||||||||||||||||||||||||||||||||||||||||||||||||||||||||||||||||
AGCGTCCCACGGGCACCGGTGGGAGGAGGTTCACCTGATCTGTCAAACTGATCGACCAGCAATGATCCTCGGCCCAGCCGACGGCAATGCTTGAGCTCAC
CGGTACCTGATACATATATCTAGAGAAGCTGGAATAAATGTACCGGAAGGTTCATGTCTGATCTATCTAACTGGTTGATCAGCCATGGCCCAGCTCGAAG
||||||||||||||||||||||||||||||||||||||||||||||||||||||||||||||||||||||||||||||||||||||||||||||||||||
CGGTACCTGATACATATATCTAGAGAAGCTGGAATAAATGTACCGGAAGGTTCATGTCTGATCTATCTAACTGGTTGATCAGCCATGGCCCAGCTCGAAG
GCAGTTAGTTAAGCTCGAGCTTTGCCGGTACTTATGTCTGCGTGCTTACTAATACGTACACACATGATAGCCGCATCACACGGTACTTAGCTAGAAGAGT
||||||||||||||||||||||||||||||||||||||||||||||||||||||||||||||||||||||||||||||||||||||||||||||||||||
GCAGTTAGTTAAGCTCGAGCTTTGCCGGTACTTATGTCTGCGTGCTTACTAATACGTACACACATGATAGCCGCATCACACGGTACTTAGCTAGAAGAGT
TGGAATAAAGTACCTGTGTTTGTACTTGTAGGCTCTTAAGTAAGGTGCCTTTGCAAGGATGCGTTGCTACTGTTCAATAAGGAATGTGACATTATGCCAC
||||||||||||||||||||||||||||||||||||||||||||||||||||||||||||||||||||||||||||||||||||||||||||||||||||
TGGAATAAAGTACCTGTGTTTGTACTTGTAGGCTCTTAAGTAAGGTGCCTTTGCAAGGATGCGTTGCTACTGTTCAATAAGGAATGTGACATTATGCCAC
ACCTTGGTAATGAGATATGTGCGGTTGGTTGTTCTGATCTTCAGGTTGAGCAAAATTATGTTGTATTTTTGTGAGTTAAATTAGAACAACCATGAGCATA
||||||||||||||||||||||||||||||||||||||||||||||||||||||||||||||||||||||||||||||||||||||||||||||||||||
ACCTTGGTAATGAGATATGTGCGGTTGGTTGTTCTGATCTTCAGGTTGAGCAAAATTATGTTGTATTTTTGTGAGTTAAATTAGAACAACCATGAGCATA
ATGTTGGCCGATATAGAGATCAAAGTGATGACAGATGCACATTATACCTCTATCGTGCCACAAAAAATTGAAATCAAATTTTGGTCCAAAGAGTGTGAAT
||||||||||||||||||||||||||||||||||||||||||||||||||||||||||||||||||||||||||||||||||||||||||||||||||||
ATGTTGGCCGATATAGAGATCAAAGTGATGACAGATGCACATTATACCTCTATCGTGCCACAAAAAATTGAAATCAAATTTTGGTCCAAAGAGTGTGAAT
TGTTCACACCAAATATGAACTTGGTACGTACGATCTACCAGTATATTTTTCCTTATTTGTTTCTCATGGTGTGCATTGAATTTATTTCTGGACTCATGCA
||||||||||||||||||||||||||||||||||||||||||||||||||||||||||||||||||||||||||||||||||||||||||||||||||||
TGTTCACACCAAATATGAACTTGGTACGTACGATCTACCAGTATATTTTTCCTTATTTGTTTCTCATGGTGTGCATTGAATTTATTTCTGGACTCATGCA
TGGCAAAACTTCTCGAAGATTCGCGTCTCGAGATGTAGTTTGGCGCTTAAATTCCTTGGGAGGCGCTGATGCGATGTTCTAACAAAAATCCTTGGTCGAA
||||||||||||||||||||||||||||||||||||||||||||||||||||||||||||||||||||||||||||||||||||||||||||||||||||
TGGCAAAACTTCTCGAAGATTCGCGTCTCGAGATGTAGTTTGGCGCTTAAATTCCTTGGGAGGCGCTGATGCGATGTTCTAACAAAAATCCTTGGTCGAA
ATGTTCTTTGATGGGATGATGGTGGTGCTTCATGCATCATTCCCTTCATGGGGGACATTATCTTGGAGCCGATATAGGTGTGAGACCGGTGGCTGGCGGC
||||||||||||||||||||||||||||||||||||||||||||||||||||||||||||||||||||||||||||||||||||||||||||||||||||
ATGTTCTTTGATGGGATGATGGTGGTGCTTCATGCATCATTCCCTTCATGGGGGACATTATCTTGGAGCCGATATAGGTGTGAGACCGGTGGCTGGCGGC
TTGACCATATGGAGGACATATTAGTTTCTGTAGATCCGTTGGCATTGCAACTCGGGTGGCAGAGAAACTGATGTTAGTGGGGCTATGTTGCATTACAAAC
||||||||||||||||||||||||||||||||||||||||||||||||||||||||||||||||||||||||||||||||||||||||||||||||||||
TTGACCATATGGAGGACATATTAGTTTCTGTAGATCCGTTGGCATTGCAACTCGGGTGGCAGAGAAACTGATGTTAGTGGGGCTATGTTGCATTACAAAC
TTCTACGTCGATTATTCGACTTCATTTGGTTCTCTCCGGGCACCCCACT
|||||||||||||||||||||||||||||||||||||||||||||||||
TTCTACGTCGATTATTCGACTTCATTTGGTTCTCTCCGGGCACCCCACT

***FT-A5 -TRIAE_CS42_5AL_TGACv1_376373_AA1235960***

TAATCAGTTAATGTGGCCAGGCTCCTGCCGAGGGCGATTAAGTGGATGCAGTCAATATGGTTCCTATCCCTACTAGTCCATTCCTGCAATCCCTGAATTT
|||||||||||||||||||||||||||||||||| |||||||||||||||||||||||||||||||||||||||||||||||||||||||||||||||||
TAATCAGTTAATGTGGCCAGGCTCCTGCCGAGGGAGATTAAGTGGATGCAGTCAATATGGTTCCTATCCCTACTAGTCCATTCCTGCAATCCCTGAATTT
GAATAAAATAATAACCCTATCATATAAGGAAGATGAGTTTTCAGAAGGGGTAAAATCTGTTGACACTATGTAAATGCATTAAATGCCAGCAATATCAGCA
|||||||||||||||||||||||||||||||||||||||||||||||||||||||||||| |||||||||||||||||||||||||||||||||||||||
GAATAAAATAATAACCCTATCATATAAGGAAGATGAGTTTTCAGAAGGGGTAAAATCTGTGGACACTATGTAAATGCATTAAATGCCAGCAATATCAGCA
GATGTCTTAGAAATTGCAAGAAAAAATACTTAACATCACTAGAAATAGGCAGTTAATGAGAACCCAAACACGTTAGGTACACGTATATGTTCAGTGCCAC
||||||||||||||||||||||||||||||||||||||||||||||| |||||||||||||||||||||||||||||||||||||||||| |||||||||
GATGTCTTAGAAATTGCAAGAAAAAATACTTAACATCACTAGAAATATGCAGTTAATGAGAACCCAAACACGTTAGGTACACGTATATGTCCAGTGCCAC
TACATCATTAGGTTCAGAAATATGGTACTTATTGTTTCCAAACAGATTGTGTCCAAAGGAACAGCTGGTTGAGCCAATGATTACCCACAGTTTGCAGGTT
||| ||||||||||||||||||||||||||||||||||||||||||||||||||||||||||||||||||||||||||||||||||||||||||||||||
TACGTCATTAGGTTCAGAAATATGGTACTTATTGTTTCCAAACAGATTGTGTCCAAAGGAACAGCTGGTTGAGCCAATGATTACCCACAGTTTGCAGGTT
TGTTCCAGTACAGTGCAGCAGTGATAATAGGATGGCTTAAGCATGAACGTCGAAATATCAACAGCCTGAGAGCCTGACAACTCCATAAAGCTCAGGATCT
||||||||||||| ||||||||| ||||||||||||||||||||||||||| ||||||||||||||||||||||||||||||||||||||||||||||||
TGTTCCAGTACAGCGCAGCAGTGCTAATAGGATGGCTTAAGCATGAACGTCAAAATATCAACAGCCTGAGAGCCTGACAACTCCATAAAGCTCAGGATCT
AATCGACAAATATCAAGGACGTCGTGTATATACGGGTGACGCATGGCATGACACAAGTATCAAAACTTTATAAAGTTGAATGAGACAATCCTCATCGCGC
||||||||||||||||||||||||||||||||||||||||||||||||||||||||||||||||||||||||||||||||||||||||||||||||||||
AATCGACAAATATCAAGGACGTCGTGTATATACGGGTGACGCATGGCATGACACAAGTATCAAAACTTTATAAAGTTGAATGAGACAATCCTCATCGCGC
ACTATAAAACTAGTTCATGCACAGACACAATTTCTTCAGGGCCATGGAGAGGTAGGTTAACAGAATAACAGAAAAAGTTCCAGTATATGTCGGCAGTGGA
||||||||||||||||||||||||||||||||||||||||||||||||||||||||||||||||||||||||||| | ||||||||||||||||||||||
ACTATAAAACTAGTTCATGCACAGACACAATTTCTTCAGGGCCATGGAGAGGTAGGTTAACAGAATAACAGAAAAGGGTCCAGTATATGTCGGCAGTGGA
TCCCTTGGTTGCGGCTCATGTTATACATGATGTGTTGGATCCATTTACATCAACTGTTCCACTCACAATAGGATACAACAATAGGCTAGTTCGGCCAGGT
||||||||||||||||||||||||||||||||||||||||||||||||||||||||||||||||||||||||||||||||||||||||||||||||||||
TCCCTTGGTTGCGGCTCATGTTATACATGATGTGTTGGATCCATTTACATCAACTGTTCCACTCACAATAGGATACAACAATAGGCTAGTTCGGCCAGGT
GCGGAGCTAAAACCATCTGCAGTTGTAAGCAAGCCGCGAGTTGATATTGGTGGCAATGACATGAGAGTTCTCTACACCCTGGTAAACTTCTAACTGGAGT
|| ||||||||||||||||||||||||||||||||||||||||||||||||||||||||||||||||||||||||||||||||
GC------------------AGTTGTAAGCAAGCCGCGAGTTGATATTGGTGGCAATGACATGAGAGTTCTCTACACCCTGGTAAACTTCTAACTGGAGT
AGTTGGCAGTATACACCCATTCTTCGGCTATCTAACTCTTTCACATGGTTTATTACAATTAGTGTGAAGAATTTCGTAGAAACAAAAGGTCTTGCAGGTG
||||||||||||||||||||||||||||||||||||||||||||||||||||||||||||||||||||||||||||||||||||||||||||||||||||
AGTTGGCAGTATACACCCATTCTTCGGCTATCTAACTCTTTCACATGGTTTATTACAATTAGTGTGAAGAATTTCGTAGAAACAAAAGGTCTTGCAGGTG
AATAATTTACTCTTAGCATACTCAAACACCATAATTATATGTTAACTTGGTTGAATTTCTCTTTCAATTGAAAAGATGTTGGTGGATCCAGATGCCCCAA
||||||||||||||||||||||||||||||||||||||||||||||||||||||||||||||||||||||||||||||||||||||||||||||||||||
AATAATTTACTCTTAGCATACTCAAACACCATAATTATATGTTAACTTGGTTGAATTTCTCTTTCAATTGAAAAGATGTTGGTGGATCCAGATGCCCCAA
GCCCAAGTCACCCATCTCTAAGGGAGTACTTGCACTGGTAAACTAAACGCGACATGTTCTTATTCTTGTCAATATGACCCCCTACCCATTTCTTTCCTGC
||||||||||||||||||||||||||||||||||||||||||||||||||||||||||||||||||||||||||||||||||||||||||||||||||||
GCCCAAGTCACCCATCTCTAAGGGAGTACTTGCACTGGTAAACTAAACGCGACATGTTCTTATTCTTGTCAATATGACCCCCTACCCATTTCTTTCCTGC
CCCACATTTTTTGTATGTGTTTCTATTTAATTTTATCTCATTTTCATATATCTCCACTCGTACGTAGGATGGTGGCAGGCATCCCTGGAACAACTGGTGT
|||||||||||||||||||||||||||||||||||||||||||||||||||||||||||||||||||||||||||||| |||||||||||||||||||||
CCCACATTTTTTGTATGTGTTTCTATTTAATTTTATCTCATTTTCATATATCTCCACTCGTACGTAGGATGGTGGCAGACATCCCTGGAACAACTGGTGT
CAGCTTTGGTACGATGTTCCTCATAACAATTTGTGCCATTTGATTTTGCGGGTTTACCTGAATTTGTGCATGTGGAAATTATCACTTGTTAAGTTCTTTG
||||||||||||||||||||||||||||||||||||| ||||||||||||||||| |||||||||||||||||||||||||||||||||||||||||||
CAGCTTTGGTACGATGTTCCTCATAACAATTTGTGCCTATTGATTTTGCGGGTTTAGCTGAATTTGTGCATGTGGAAATTATCACTTGTTAAGTTCTTTG
CCTAACATTC------------ACAACAACAACAACAAAGA-TTTTTTTTGCCTCAAACCTGTTGCCTACCATAGAATTAACAGATAGGGCTATCACTTA
|||||||||| ||||||||||||||||||| ||||||||||||||||||||||||||||||||||||||||||||||||||||||||||
CCTAACATTCACAACAACAACAACAACAACAACAACAAAGATTTTTTTTTGCCTCAAACCTGTTGCCTACCATAGAATTAACAGATAGGGCTATCACTTA
AATCAGGTAAAAGTTATTTCAAAGTTGGCTCATCATTAACAATTGGATATCTTAATACTAGAAAAATAATATCTATCAACGTGTAAAATGACATGCACGT
||||||||||||||||||||||||||||||||||||||||||||||||||||||||||||||||||||||||||||||||||||||||||||||||||||
AATCAGGTAAAAGTTATTTCAAAGTTGGCTCATCATTAACAATTGGATATCTTAATACTAGAAAAATAATATCTATCAACGTGTAAAATGACATGCACGT
GTTTTAAAGATTTTGGAAATCATTAAGACAAGTCCATTGTACAGCCTAAAATACAGGGAGATCCTAAAATAGTGGCATACTTCAGAAACACCTTTCCCGA
||||||||||||||||||||||||||||||||||||||||||||||||||||||||||||||||||||||||||||||||||||||||||||||| ||||
GTTTTAAAGATTTTGGAAATCATTAAGACAAGTCCATTGTACAGCCTAAAATACAGGGAGATCCTAAAATAGTGGCATACTTCAGAAACACCTTTTCCGA
TCTAGCTTGAAAAATGATCTTATATTGTGGGACGAAGGGGGTATCATTTATACTTGATTTATTTTTCACATGCACAAAACCAAACTTGCGAAATGAATTA
|||||||||||||||||||||||||||||||||| ||||||||||||||||||||||||||||||| |||||||||||||||||||||||||||||||||
TCTAGCTTGAAAAATGATCTTATATTGTGGGACGGAGGGGGTATCATTTATACTTGATTTATTTTTGACATGCACAAAACCAAACTTGCGAAATGAATTA
TCATAATTCAACAATATAGATAC--GTGACTAGATGTCTCCACCCAGTTGAATTACTCTTTAAATTCTTAAGTGGTTAAAGACTAATTAATGCCATGGTG
||||||||||||||||||||||| |||||||||||||||||||||||||||||||||||||||||||||||||||||||||||||||||||||||||||
TCATAATTCAACAATATAGATACGTGTGACTAGATGTCTCCACCCAGTTGAATTACTCTTTAAATTCTTAAGTGGTTAAAGACTAATTAATGCCATGGTG
CTCTTGCAGGCCAAGAGCTTGTAGTTTATGAAAGACCGGAGCCAAGATCCGGCATCCACCGAATGGTATTTGTGCTGTTCCAGCAACTAGGTAGGGGGAC
||||||||||||||||||||||||||||||||||||||||||||||||||||||||||||||||||||||||||||||||||||||||||||||||||||
CTCTTGCAGGCCAAGAGCTTGTAGTTTATGAAAGACCGGAGCCAAGATCCGGCATCCACCGAATGGTATTTGTGCTGTTCCAGCAACTAGGTAGGGGGAC
GGTTTTTGCACCGGACATGCGGCACAACTTCAGCTCCAGGAACTTCGCACGCCAGTACCACCTCAACATTGCGGCTGCCACATATTTCAACTGTCAAAGG
||||||||||||||||||||||||||||||||||| ||||||||||||||||||||||||||||||||||||||||||||||||||||||||||||||||
GGTTTTTGCACCGGACATGCGGCACAACTTCAGCTTCAGGAACTTCGCACGCCAGTACCACCTCAACATTGCGGCTGCCACATATTTCAACTGTCAAAGG
GAAGGTGGA---GGCGGAAGAAGGTTTAGGCCAGAAAGTTCTCAAGGGTAGTAGAGACTAGACACTACGGAGTACAGACGTGGTGTTGCATCACAATATA
||||||||| |||||||||| |||||||||||||||||||||||||||||||||||||||||||||||||||||||||||||||||||||||||||||
GAAGGTGGATCGGGCGGAAGAATGTTTAGGCCAGAAAGTTCTCAAGGGTAGTAGAGACTAGACACTACGGAGTACAGACGTGGTGTTGCATCACAATATA
GGATGCTCTGTATCATCCATATATGCAGGGTATGTATCTTCACTACATTATCTACAAAAGACTATGTATCTTCAGCTATGACGAGGATGATGGAATATAC
||||||||||||||||||||||||||||||||||||||||||||||||||||||||||||||||||||||||||||||||||||||||||||||||||||
GGATGCTCTGTATCATCCATATATGCAGGGTATGTATCTTCACTACATTATCTACAAAAGACTATGTATCTTCAGCTATGACGAGGATGATGGAATATAC
ATCGGTTATTACTAGTAAAAGAGCCCGTGCGTTGCAACGGGAGAGAAAACATAACACACGCTCTTAACTTAACAACCATCACTCAAGACCATAATAGGTC
 |||||||||||||| |||| |||||||||||||||||||||||||||||||||||||||||||||||||||||||||||||||||||||||||||||||
GTCGGTTATTACTAGCAAAAAAGCCCGTGCGTTGCAACGGGAGAGAAAACATAACACACGCTCTTAACTTAACAACCATCACTCAAGACCATAATAGGTC
CATCTTCTTTATTTTTGCGAGGCATCATATTTGTGTTGCCGCTTATCCTCCTTCTCACCCTCGCCGGTGATGGTCTCGGTGTTCACACAAAACAACAAAA
||||| ||||||||||||||||||||||||||||||||||||||||||||||||||||||||||||| ||||||||||||||||| ||||||||||||||
CATCTCCTTTATTTTTGCGAGGCATCATATTTGTGTTGCCGCTTATCCTCCTTCTCACCCTCGCCGGCGATGGTCTCGGTGTTCATACAAAACAACAAAA
AAACGTGTGTTGAATATGGTTAATTCTAAGACGCCTCTCTCTCTCCCTCTCTCCTCCCTCTCCCACTCTCTCTCTCTCTCTCTCTCTCTCTCTCTCTCTC
|||||||||||||||||||||||| ||||||||| ||||||||||||||||||||||||||| ||||||||||||||||||||||||| || |||||
AAACGTGTGTTGAATATGGTTAATCCTAAGACGC--CTCTCTCTCCCTCTCTCCTCCCTCTCCTTCTCTCTCTCTCTCTCTCTCTCTCTCGCTTTCTCT-
TCTCTCTCTCTCTCTCTCTCTCTCTCTCTCGCGCGCGCGCTTTCTCTCACTCTCGCGATGAGAAATCTGTTGTTTTCCCCTGCGAAATTTTTCAGAGGTA
 |||||||||||||||||||||||||||||||||||||| ||||||||||||||
-----------------------------------------------CACTCTCGCGATGAGAAATCTGTTGTTTTCCCCTGCGACATTTTTCAGAGGTA
TGCTTGTGTAGTTATCGATGTTTTCTTTTCCGTATATGGTTATAGTGGGGTGTTTATTTGCAACCCGGATCGCCGCCGGTATGAAAAAAAAACGAATCTT
||||||||||||||||||||||||||||||| ||||||||||||||||||||||||||||||| ||| ||||||||||||||| ||||||| |||||||
TGCTTGTGTAGTTATCGATGTTTTCTTTTCCATATATGGTTATAGTGGGGTGTTTATTTGCAATCCGAATCGCCGCCGGTATG-GAAAAAAAGGAATCTT
GCGCTATAAATTAT
||||||||||||||
GCGCTATAAATTAT

***FT-B5 - TRIAE_CS42_4BL_TGACv1_321072_AA1054780***

GTGAAAGTTAGGAAGCTTATCGCTCTTGGGTGTTTGGGCACCCTAGAGCTTGTTCCTCTTGGGTATTTTGGTGCACTAGACGGTTGGTGCATTTGGAGCT
|||||||||||||||||||| || ||||||||||| | || ||||||||||||||||||| | |||||||||||||||||||||||| | ||||||
GTGAAAGTTAGGAAGCTTATTACTTTTGGGTGTTTGAGAACACTAGAGCTTGTTCCTCTTGTGC--TTTGGTGCACTAGACGGTTGGTGCGTGCGGAGCT
CAATCGTTGTCGTGTAAAGCTCCGGGCAAGGGTCGGGATCTCCAATTAGGTTGTGGAGATCGCCCCGAGCAATTTGTACGGGTTCCGCTGACCGCCCTCA
||||| |||| |||||||||||| |||||| |||||| ||||||||||||||||| ||||||| ||||||||||||||||||||||| |||||||| |||
CAATCATTGTGGTGTAAAGCTCCAGGCAAGCGTCGGGGTCTCCAATTAGGTTGTGAAGATCGCTCCGAGCAATTTGTACGGGTTCCGATGACCGCCATCA
ATGGTTGCCAAAGTGTATGAGTTCGGTGACCGCCCCCAAGGGTTGCAATTTGTATGGGTTCGGTGAAGGCCCTCAAGGGTCCCTTAGTGAAATCACCGCA
| ||||||||||||||||| ||||||||||| |||| ||||||||| |||| || ||||||||||| | ||||||||||||||||||| |||||| |||
AGGGTTGCCAAAGTGTATGCGTTCGGTGACCACCCCTAAGGGTTGCCATTTTTACGGGTTCGGTGACCGTCCTCAAGGGTCCCTTAGTGGAATCACGGCA
TCTTGCATTGTACGAGGGCCCGAGGAGATTACAGTGGCCCTAGTGGCTTTTGGGGAAGCATTGTGTCTCCACACCGCTCCAAACGGACATTAGCATCCGC
|| |||||||||||||||| |||||||||||| |||||||||||||||||| ||| ||||| |||||||||||| |||||||| | ||||||||||||||
TCATGCATTGTACGAGGGCGCGAGGAGATTACGGTGGCCCTAGTGGCTTTTTGGGGAGCATGGTGTCTCCACACTGCTCCAAATGAACATTAGCATCCGC
AAGGGTGTGAACTTCGGGATACATCATCGTCTCCGCCTGCCTCGGTTATCTCTTACCCGAGCCCTTTACTTATGCACTTTACTTTGTGACATAGTGTTAC
|||||||||||||||| ||||||| ||||||| |||||||||||||| |||||||||||||||||||||| | | || | ||||||||| |
AAGGGTGTGAACTTCGAAATACATCGTCGTCTCTGCCTGCCTCGGTTACCTCTTACCCGAGCCCTTTACTT------TGTGATT---GCCATAGTGTTTC
ATGTTATATATCTTGATATCACTTAGTTGTTTATCCTGCTTAGCATAAGTTTTTGGTGCACATAGGTGAGCCTAATTGTTTTAGGTTTTGTACTTAACAA
||||||||||||||| ||||||||||||||||||| ||||||||||||||| ||||||||| |||||||||||||||||||||||| | ||| ||||
ATGTTATATATCTTGCTATCACTTAGTTGTTTATCTTGCTTAGCATAAGTTGTTGGTGCAC----GTGAGCCTAATTGTTTTAGGTTTTATGCTTGACAA
ACTAAACGCTAGTTTTATTCCACATTTGTTCAAGCCTAAACTGTAATTATTTTAAAGCACCTATCCACCCCCCCCCCCCTCTAGGATGTC-TCCATGATC
| ||||| ||| ||||||||| |||||||| ||||||||||||||||||||||||||||||||| |||||||| |||||| || | |||||||||
ATTAAACACTA-TTTTATTCCGCATTTGTT-AAGCCTAAACTGTAATTATTTTAAAGCACCTATTCACCCCCC------TCTAGG-TGACATCCATGATC
TTTCAGCAGGTCTCATGGGAGCAGATGCAGGCGCACTATGACGTCTCCATGGAGGAGGAGGAGGAGCCTGCGCCGGCTCCTATGTCAGCATTCCAACAAG
|||||||||||||||||| ||||| ||||| ||||||||| |||||||||| |||||||||||||||||||||||||||||| || || |||| ||||
TTTCAGCAGGTCTCATGG-AGCAGGCGCAGGTGCACTATGATGTCTCCATGGTGGAGGAGGAGGAGCCTGCGCCGGCTCCTATATCGGCGTTCCGGCAAG
CACAGGAGGAGCGGAGGTGCAACAAGTTCCTCCTCGTCCAGCACTAGCTGACGGAGGGACAAATCTACGACGAGCGTGCAAGCGAGGCAGCCGTGGCCGC
| |||||||||||||||||||||| ||||||| || || |||||||||||||||||||||||||||||||||||||||||||||||||||||||||||
CGCAGGAGGAGCGGAGGTGCAACATGTTCCTC---GTGCAACACTAGCTGACGGAGGGACAAATCTACGACGAGCGTGCAAGCGAGGCAGCCGTGGCCGC
CATGGCCACGGCGAACCCGAACTTTGTTGCATAGCAGTGGGCGCTCTACGAGGCTGCGCGTGCACAAGGCGCCATTCGCAAAGCAGAGCTGGCACAGCCA
||||||||||||||||||||||||||||||||||||||||||||||||||||||||||||||||||||||||||||||||||||||||||||||||||||
CATGGCCACGGCGAACCCGAACTTTGTTGCATAGCAGTGGGCGCTCTACGAGGCTGCGCGTGCACAAGGCGCCATTCGCAAAGCAGAGCTGGCACAGCCA
GACACGGATGTGGCCGCACACGCGCTGGCGGCGCAGACGGTCCAGGACTCCAACGCAACCAGCTGTGCATCATACGCCCCGCCGTCCAACTTTTGGGGTG
||||||||||||||||||||||||||||||||||||||||||||||||||||||||||||||||||||||||||||||||||||||||||||||||||||
GACACGGATGTGGCCGCACACGCGCTGGCGGCGCAGACGGTCCAGGACTCCAACGCAACCAGCTGTGCATCATACGCCCCGCCGTCCAACTTTTGGGGTG
CACGTGGCAGGTGGACAGTGATGGACCGTCCATGTTCATCGTCGACCTCATGTCCACCAGCATCGGTGACTGACAGGTCACTGACTCCTCCGTGGTTGAG
|||||||||||||||| ||||||||||||||||||||||||||||||||| |||||||||||||||||||||||||||||||||||||||||||||||||
CACGTGGCAGGTGGACGGTGATGGACCGTCCATGTTCATCGTCGACCTCACGTCCACCAGCATCGGTGACTGACAGGTCACTGACTCCTCCGTGGTTGAG
TAGGGCATGGGATGCGGCAGGGCGTTGTGTCCCAAGTGGGCCGGTCTGTCTCCTATGTTCTATTCTTCCTCGCCGGAGATCGCACCTCCACTTCACGGTT
||||||||||||||||||||||||||||||||||||||||||||||||||||||||||||||||||||||||||||||||||||||||||||||||||||
TAGGGCATGGGATGCGGCAGGGCGTTGTGTCCCAAGTGGGCCGGTCTGTCTCCTATGTTCTATTCTTCCTCGCCGGAGATCGCACCTCCACTTCACGGTT
CTTAACCCTGCCGTCGTACATGGAGACGACAGATGGAGGTCGTGCTCGCTGATGCAGAATACAAAGGAAGTGACTGACGGGCCGACGTCGTAGGATAGGT
|||||||||||||||||||||||||||||||||||||||||||||||||||||||||||||||||||||||||||||||||||||||| |||||||||||
CTTAACCCTGCCGTCGTACATGGAGACGACAGATGGAGGTCGTGCTCGCTGATGCAGAATACAAAGGAAGTGACTGACGGGCCGACGTTGTAGGATAGGT
TTAGGGTCGGGTTTCCTTTTTTATCCTAAATATTCGAAATGTATTGAAAATCTGCCGTGTTTCCAAGAATCATGCTCTGGTAGTCAAAGTACACCAGTGC
|||||||||||||||||||||| |||||||||||||||||||||||||||||||||||||||| ||||||||||||||||||||||||||||||| ||||
TTAGGGTCGGGTTTCCTTTTTTGTCCTAAATATTCGAAATGTATTGAAAATCTGCCGTGTTTCTAAGAATCATGCTCTGGTAGTCAAAGTACACCGGTGC
CGCCAAGCCGCCACAAAACCGTGTCCAGGAATTTGCACAAGTTCTCAACCATTTCATCACTGGTACCAATCCAACAGGCGGCCGTACAATAATGATACTA
||||||||||||||||||||||||||||||||||||||||||||||||||||||||||||||||||||||||||||||||||||||||||||||||||||
CGCCAAGCCGCCACAAAACCGTGTCCAGGAATTTGCACAAGTTCTCAACCATTTCATCACTGGTACCAATCCAACAGGCGGCCGTACAATAATGATACTA
TGAGGCCGCAGCGACAGCACTGCTTAACAGCTAAGCCTTTCCTTTTTTATTTTTTATTTTTTTGAAGCTGACCGTAGAACGGTGATTTCCATTAATAAAA
||||||||||||||||||||||||||||||||||||||||||||||||||||||||||||||||||||||||||||||||||||||||||||||||||||
TGAGGCCGCAGCGACAGCACTGCTTAACAGCTAAGCCTTTCCTTTTTTATTTTTTATTTTTTTGAAGCTGACCGTAGAACGGTGATTTCCATTAATAAAA
CAAAGTTATGTACAAAGCCAAGCCTTTCATTTTCTTTTTCTTTTTGAAGCCCTGACCCGTAGAACAATTGCAAAACAAAGGTATGTA----CAAAGCAAA
|||||||||||||||||||||||||||| ||| |||||||||||||||||||||||||||||||||||||||||||||||||||||| |||||||||
CAAAGTTATGTACAAAGCCAAGCCTTTCCTTTCCTTTTTCTTTTTGAAGCCCTGACCCGTAGAACAATTGCAAAACAAAGGTATGTATGTACAAAGCAAA
GCTAAGCCTTTCATCGCAGAGCTTAGCCGGTAAATTTGAGTTTGGCTCCCTTCTGCCTTGCTAGCTCAGGTTACGCATACATCCTTGATATTTTCCGACA
|||||||||||| |||||||||||||||||||||||||||||||||||||||||||||||||||||||||||||||||||||||||||||||||||||||
GCTAAGCCTTTCCTCGCAGAGCTTAGCCGGTAAATTTGAGTTTGGCTCCCTTCTGCCTTGCTAGCTCAGGTTACGCATACATCCTTGATATTTTCCGACA
AACTATCAGCTACTACATCACTTTCTTGTCCAGCCGACAAGCATGCA------TGCAGCATCC---ACCTCTGTTCAAACTCAACTTGAACTTCTTCTCT
||||||||||||||||||||||||||||||| |||||||||||||| ||| || | ||||||||||||||||||||||||||||||||||
AACTATCAGCTACTACATCACTTTCTTGTCCTGCCGACAAGCATGCGGCATCTTGCTGCCTAGCTAACCTCTGTTCAAACTCAACTTGAACTTCTTCTCT
AGTTCCTTGGCTCTTACTTCGTTTCAATAACTAGTAATAGCGGCCTAGAGGTTTCAACAGTGAAAAAAGCATGATGAGCAACGTCTTACTAAAATAAATT
||||||||||||||||||||||||||||||||||||||||||||||||||||||||||||||||||||||||||||||||||||||||||||||||||||
AGTTCCTTGGCTCTTACTTCGTTTCAATAACTAGTAATAGCGGCCTAGAGGTTTCAACAGTGAAAAAAGCATGATGAGCAACGTCTTACTAAAATAAATT
AATGTATAGTCTCGCTAATAACATGCTAATAGTGTATTTTAAGGACCACGCTAATTTGTCAATTTT-TTTCATTGGGTTTCATGCAGCGTGTAGCTCTGT
|||||||||||||||||||||||||||||||||||||||||||||||||||||||||||||| |||||||||||||||||||||||||||||||||
AATGTATAGTCTCGCTAATAACATGCTAATAGTGTATTTTAAGGACCACGCTAATTTGTCAAAAAAATTTCATTGGGTTTCATGCAGCGTGTAGCTCTGT
ACAGCCAGCATACATGCATTAGTCAAAACCAATTCAATTTGACTTCTTCTTCCTTGCACGCATCTCTCAATTTCATTGTTTAACCATAATGGCCAGATTA
||||||||||||||||||||||||||||||||||||||||||||||||||||||||||||||||||||||||||||||||||||||||||||||||||||
ACAGCCAGCATACATGCATTAGTCAAAACCAATTCAATTTGACTTCTTCTTCCTTGCACGCATCTCTCAATTTCATTGTTTAACCATAATGGCCAGATTA
ATTCTAACATGTTCTACTGTTCCAGTAATCATAAAGCTCAGTATCTAATCGGCAAATATCAAGGCCGGCCGTCATGTGTACAAAGGTGACGCATGACATG
||||||||||||||||||||||||||||||||||||||||||||||||||||||||||||||||||||||||||||||||||||||||||||||||||||
ATTCTAACATGTTCTACTGTTCCAGTAATCATAAAGCTCAGTATCTAATCGGCAAATATCAAGGCCGGCCGTCATGTGTACAAAGGTGACGCATGACATG
GCACTAGTATCATGGAGTCGCTTTCGTCCTGGAGATATTCCTTCTCATCACTTGGGAAGGGTCGTGACTTGAATGACTTATAGTTCCAACGAGAACCCAA
||||||||||||||||||||||||||||||||||||||||||||||||||||||||||||||||||||||||||||||||||||||||||||||||||||
GCACTAGTATCATGGAGTCGCTTTCGTCCTGGAGATATTCCTTCTCATCACTTGGGAAGGGTCGTGACTTGAATGACTTATAGTTCCAACGAGAACCCAA
ACATGTCACCTACACTACACTACACCCCATCCCAGGAATGGCGTCGACGTCGACGTCGAAATATCAACAGCCTGGGAGCCTTATCGCACACCATACAAAT
|||||||||||||||||||||||||||||||||||||| |||||||||||||||||||||||||||||||||||||||||||||||||||||||||||||
ACATGTCACCTACACTACACTACACCCCATCCCAGGAACGGCGTCGACGTCGACGTCGAAATATCAACAGCCTGGGAGCCTTATCGCACACCATACAAAT
AGTGGCTTCATAAATCTCAAGATGATATGCCGGCTCAGTCTCTCAAAGGTGCTCATAGAGGTAGGGTGTGCGTGTGTGCGTTCATAGGGATGAATGTATG
|||||||||||||||||||||||||||||||||||||||||||||||||||||||||| |||||||||||||||||||||||||||| | ||| |||||
AGTGGCTTCATAAATCTCAAGATGATATGCCGGCTCAGTCTCTCAAAGGTGCTCATAGGGGTAGGGTGTGCGTGTGTGCGTTCATAGCGGTGAGTGTATA
CGCGTGTATATGAGCTCTTGTGTCTATACTGATGCTCCAAAAAAAAT--TTGGTGCATAGAAACAACAATTCCCCAGCAGGTCCGTGGAGAGCTAGGTCA
|||||||||||||||||||||||||||||||||||||||||||||| |||||||||||||||||||||||||||||||||||||||||||||||||||
CGCGTGTATATGAGCTCTTGTGTCTATACTGATGCTCCAAAAAAAAAAATTGGTGCATAGAAACAACAATTCCCCAGCAGGTCCGTGGAGAGCTAGGTCA
AGAGAATAACCGAAATCCAGCATATATGTCAGCAGTACTTCAAATGTAGCGAATGGAGCTTTGTGGATGACAAATTCATCGATCATGGTGAAAACTGGAT
|||||||||||||||||| ||||||||||||||||||||| |||||||||||| ||||||||||||||||||||||||||||||||||||||||||||||
AGAGAATAACCGAAATCCGGCATATATGTCAGCAGTACTTGAAATGTAGCGAACGGAGCTTTGTGGATGACAAATTCATCGATCATGGTGAAAACTGGAT
TAGTGAGGTGCTGTCTATGGGAGGGAGACTCATTAAAGTGAATACGGTACTGTCACATATACCAACCTTTTACATGTCCATGTTTTTACTGAACAAGACC
||||||||||||||||||||||||||||||||||||||||||| ||||||||||||||||||||||||||||||||||||||||||||||||||||||||
TAGTGAGGTGCTGTCTATGGGAGGGAGACTCATTAAAGTGAATGCGGTACTGTCACATATACCAACCTTTTACATGTCCATGTTTTTACTGAACAAGACC
ACATTAGAGAGGTGGGACAAGCCGAGAAGGAATTTTTTCTGGCATAGAAAAGGAAAGAAAAAAGGATACCATATGGTCAAGTGGGACCGCGTTTGTCGAT
||||||||||||||||||||||||||||||||||||||||||||||| ||||||||||||||||||||||||||||||||||||||||||||||||||||
ACATTAGAGAGGTGGGACAAGCCGAGAAGGAATTTTTTCTGGCATAGGAAAGGAAAGAAAAAAGGATACCATATGGTCAAGTGGGACCGCGTTTGTCGAT
CTAAAAAGAAAGGGGGCCTGGGGGTCAAGGACCTGAGAAAGCAAAATATCAGCTTACTGGTGAAATGGTGGTGGAAACTAGATAAGAATAAAGGCCTCTG
||||||||||||||||||||||||||||||||||||||||||||||||||||||||||||||||||||||||||||||||||||||||||||||||||||
CTAAAAAGAAAGGGGGCCTGGGGGTCAAGGACCTGAGAAAGCAAAATATCAGCTTACTGGTGAAATGGTGGTGGAAACTAGATAAGAATAAAGGCCTCTG
GCAGGATATAGTTAAAGCTAAATACTTGAAGAAAACCTCAGTGGCCATGGTAAAGGCAAAAAACAATGATTCACCATGCTGGAAATCCCTCTTGAAAGTG
|||||||||||||||||||||||||||||||||||||||| |||||||||||||||||||||||||||||||||||||||||||||||||||||||||||
GCAGGATATAGTTAAAGCTAAATACTTGAAGAAAACCTCAATGGCCATGGTAAAGGCAAAAAACAATGATTCACCATGCTGGAAATCCCTCTTGAAAGTG
AAAAACTTATATATGAATGGCAGGGGGGTCAAACTGAATAAAGGAGATGTGGCCAGACTATGGTTCCATCAATTGGAAGGAAGGATCCCATTTAAAGAAA
||||||||||||||||||||||||||||||||||||||||||||||||||||||||||||| |||| |||||||||||||||||||||||||||||||||
AAAAACTTATATATGAATGGCAGGGGGGTCAAACTGAATAAAGGAGATGTGGCCAGACTATAGTTCGATCAATTGGAAGGAAGGATCCCATTTAAAGAAA
AGTTCCCATTACTGTTTGATATCTGTGTAGAACAAAACTGTACTGTTGATAGAATGGACTTGTTGAATCATATTACCTCTTTTAGGAGGAGGATGTCCCC
||||||||||||||||||| ||||||||||||||||||||||||||||||||||||||||||||||||||||||||||||||||||||||||||||||||
AGTTCCCATTACTGTTTGAAATCTGTGTAGAACAAAACTGTACTGTTGATAGAATGGACTTGTTGAATCATATTACCTCTTTTAGGAGGAGGATGTCCCC
TGAAATGATGAAACAATGGGATGAAATGAAAAAGGAGGTATTAACACTTAAACAAAATGATTTTCCTGATGAGATTTATTGGAAATTTGATAACTCTGGA
||||||||||||||||||||||||||||||||||||||||||||||||||||||||||||||||||| ||||||||||||||||||||||||||||||||
TGAAATGATGAAACAATGGGATGAAATGAAAAAGGAGGTATTAACACTTAAACAAAATGATTTTCCT-ATGAGATTTATTGGAAATTTGATAACTCTGGA
AAATATACAACCAACTCGATGTATAGATGGTTGGAAAGAGATATTGCTGGATCCAGCTCAAGTGGATTTGGGATGCTAAGCTCCCCTTAAAAATTCAAAT
||||||||||||||||||||||||||||||||||||||||||||||||||||||||||||||||||||||||||||||||||||||||||||||||||||
AAATATACAACCAACTCGATGTATAGATGGTTGGAAAGAGATATTGCTGGATCCAGCTCAAGTGGATTTGGGATGCTAAGCTCCCCTTAAAAATTCAAAT
CTTCTTATGGCAGGTGGGGCAAAATGCCATCTTGACTAGAGATAATATGAAAAAAAGATTTTGGCCTGGAAACCCCTGTTGCTCCTTCTGTAATCAATTG
||||||||||||||||||||||||||||||||||||||||||||||||||||||||||||||||||||||||||||||||||||||||||||||||||||
CTTCTTATGGCAGGTGGGGCAAAATGCCATCTTGACTAGAGATAATATGAAAAAAAGATTTTGGCCTGGAAACCCCTGTTGCTCCTTCTGTAATCAATTG
GAAACAACCTCGCACTTTCTTTTCTTGTGTCCTGTATCTAGAGTGATTTGGAGAACTGTAGGAGCTCTCCTTGGGACTGATTGTTGTCCTAATTCCATCT
||||||||||||||||| |||||||||||||||||||||||||||||||||||||||||||||||||||||||||||||||||||||||||||| |||||
GAAACAACCTCGCACTTGCTTTTCTTGTGTCCTGTATCTAGAGTGATTTGGAGAACTGTAGGAGCTCTCCTTGGGACTGATTGTTGTCCTAATTTCATCT
GGCAGTATTATACTTGGATGTATAGTTTCTTACCTGGCTTTGAAAAAAATTTATACTATTGGCTTGGCAGCTGTATGCTGGGCCATATGGCTAGCCCGGA
|||||||||||||||||||||||||||||||||||||||||||||||| |||||| | ||||||||||||||||||||||||||||||||||||||||||
GGCAGTATTATACTTGGATGTATAGTTTCTTACCTGGCTTTGAAAAAA-TTTATATTGTTGGCTTGGCAGCTGTATGCTGGGCCATATGGCTAGCCCGGA
ACAGAGCTACTTCCGAATGCAAATGGATTAACTCTCCATTTGAGATCGTGTTCATAGCTTGTGCTTTTCTAATATACTGGGAAATCCTCCATAAACCGGA
||||||||||||||||||||||||||||||||||||||||||||||||||||||||||||||||||||||||||||||||||||||||||||||||||||
ACAGAGCTACTTCCGAATGCAAATGGATTAACTCTCCATTTGAGATCGTGTTCATAGCTTGTGCTTTTCTAATATACTGGGAAATCCTCCATAAACCGGA
GATGGAAGAGATGGTGAAGAAGGGCGCGGAGATGCTGAAAGAGAATACAACTCAGATGATGTTGCTGGTGTGGACCTCCGGCTCCAAACATGGATGGGCA
||||||||||||||||||||||||||||||||||||||||||||||||||||||||||||||||||||||||||||||||||||||||||||||||||||
GATGGAAGAGATGGTGAAGAAGGGCGCGGAGATGCTGAAAGAGAATACAACTCAGATGATGTTGCTGGTGTGGACCTCCGGCTCCAAACATGGATGGGCA
CGACTGAAGCTGAAGATGGATGACAGTTTGTGCTCGACATCGTCTGCTGGGGTGTACTGTTGGGGCTTTGCATGTGGGGGGTGTTTAGTTTATTTTGGTA
|||||||||||||||||||||| ||||||||||||| |||||||||||||||||||||||||||||||||||||||||||||||||||||||||||||||
CGACTGAAGCTGAAGATGGATGGCAGTTTGTGCTCGGCATCGTCTGCTGGGGTGTACTGTTGGGGCTTTGCATGTGGGGGGTGTTTAGTTTATTTTGGTA
ACTGATGATACTTTGGCATGTGCCTGGTGATGTGCGTCTGTAATATGTGTGCTACTGCTCTAGTTCCTGTAGGCGGCTTTGGGTGATAACAGGTTTTCGC
||||||||||||||||||||||||||||||||||||||||||||||||||| ||||||||||||||||||||||||||||||||||||||||||||||||
ACTGATGATACTTTGGCATGTGCCTGGTGATGTGCGTCTGTAATATGTGTGATACTGCTCTAGTTCCTGTAGGCGGCTTTGGGTGATAACAGGTTTTCGC
CCCATGATCTGTTGTTTCTGATGACAGGCGCAGATGGTGGGTTTCCTGTTATTGCCCTGAACTCGCTTTTCTTCTTTCCCTAGCCTTGTTAATTTCAGTT
|||||||||| |||||||||||||||||||||||||||||||||||||||||||||||||||||||||||||||||||||||||||||||||||||||||
CCCATGATCTCTTGTTTCTGATGACAGGCGCAGATGGTGGGTTTCCTGTTATTGCCCTGAACTCGCTTTTCTTCTTTCCCTAGCCTTGTTAATTTCAGTT
CCTGAGACATTGTATTTCCGTTATGAAAGTAATGGAAAGGGGAAAAGCCCGGTTTGAAAATGTCAGCAGTACTGGATCCCTTGGTTGTGGCTCGAGTCAT
|||||||||||||||||||||||||||||||||||||||||||||||||||||||||||||||| |||||||||||||||||||||||| ||||||||||
CCTGAGACATTGTATTTCCGTTATGAAAGTAATGGAAAGGGGAAAAGCCCGGTTTGAAAATGTCGGCAGTACTGGATCCCTTGGTTGTGCCTCGAGTCAT
ACACGATGTGTTGGATCCGTTTACATCAACTGTCCCACTCAGCATAGGCTACAACAACAGGCTACTTCAGCGAGGTGCTGAGCTGAGACCATCTGTCGTC
||||||||||||||||||||||||||||||||||||||||||||||||||||||||||||||||||||||||||||||||||||||||||| |||||||
ACACGATGTGTTGGATCCGTTTACATCAACTGTCCCACTCAGCATAGGCTACAACAACAGGCTACTTCAGCGAGGTGCTGAGCTGAGACCAACTGTCGTA
GTAAGCAAGTCGCGAGTCGATGTCGGTGGCAATGACATGAGACTTCTCTACACCCTGGTAAACTTCTAACTGGACTATAGTAGGGCACTATACACGACTT
||||||||| ||||||||||||||||||||||||||||||||||||||||||||||||||||||||||||||||||| ||||||||||||||||||||||
GTAAGCAAGCCGCGAGTCGATGTCGGTGGCAATGACATGAGACTTCTCTACACCCTGGTAAACTTCTAACTGGACTACAGTAGGGCACTATACACGACTT
CTTCAGTTATCTAACCCTTCCACACGGTTCACCTACTACTATTAGTGTGAAGAATTTCTTAGAAACACAAAAGGTCTCGCACGTGATTAATTTACTCTTA
|||||||||||||||||||||||||||||||||||||||||||||||||||||||||||| |||||||||||||||||||||||||||||||||||||||
CTTCAGTTATCTAACCCTTCCACACGGTTCACCTACTACTATTAGTGTGAAGAATTTCTTGGAAACACAAAAGGTCTCGCACGTGATTAATTTACTCTTA
ACATACTCAAACATCTTAATTATATCTTAACTTGGTTGAGTTCTCTTTTGCTTCAAAAGATGCTGGTGGATCCAGATGCCCCAAGCCCAAGTCACCCAAC
||||||||||||||||||||||||||||||||||||||| ||||||||||||||||||||||||||||||||||||||||||||||||||||||||||||
ACATACTCAAACATCTTAATTATATCTTAACTTGGTTGATTTCTCTTTTGCTTCAAAAGATGCTGGTGGATCCAGATGCCCCAAGCCCAAGTCACCCAAC
ACTAAGGGAGTACTTGCACTAGTAAATTATTATTAATAAATTAAATGCAACATGTTCATATTCTCCACACTATGACTCCCTACCTATTTGTTTTCCGCCC
|||||||||||||||||||| |||||||||||||||| |||||||||||||||||||||||||||| |||||||||||||||||||||||||||||||||
ACTAAGGGAGTACTTGCACTGGTAAATTATTATTAATGAATTAAATGCAACATGTTCATATTCTCCTCACTATGACTCCCTACCTATTTGTTTTCCGCCC
CACATTTTTTACATGTTCTTCCTGTTTAGTTTGATCTCATTATGATATACCTCCACTCATCCTGTGTAGGATGGTGGCAGACATCCCTGGAACAACTGGT
|||||||||||||||||||||||||||||||||||||||||||||||||||||||||||||||||||||||||||||||||||||||||||||||| |||
CACATTTTTTACATGTTCTTCCTGTTTAGTTTGATCTCATTATGATATACCTCCACTCATCCTGTGTAGGATGGTGGCAGACATCCCTGGAACAACCGGT
GTCAGCTTTGGTATGATGTTCTGCATAACAATTGTGCCATTTGATCGTGCGGGTTTAGCTGGATTTGTGGATGTGGAAATTATCACTTGTTAAGTTCTTT
|||||||||||||||||||||||||||||||||||||||||||||||||||||||||||||||||| |||||||||||||||||||||||||||||||||
GTCAGCTTTGGTATGATGTTCTGCATAACAATTGTGCCATTTGATCGTGCGGGTTTAGCTGGATTTATGGATGTGGAAATTATCACTTGTTAAGTTCTTT
GCCTCACATCCCCCCCCCCCCCCCCCCCAAAAAAAACACTTTGCCTCAAACCCATTTTCTAACCCGT
||||||||||||||||||||||||||||||||||| |||||||||||||||| |||||||||||||
GCCTCACATCCCCCCCCCCCCCCCCCCCAAAAAAA--ACTTTGCCTCAAACCCGTTTTCTAACCCGT

***FT-D5 - TRIAE_CS42_4DL_TGACv1_342977_AA1126650***

TAATGTGGCCAGGCTCCTGCCGAGGGCAATTAAGTGAATGCAATCATATGCTTCCTATTCCTACTAATCCATTCCTGCAATCCCTGAATTTGAATAAAAT
||||||||||||||||||||||||||||||||||||||||||||||||||||||||||||||||||||||||||||||||||||||||||||||||||||
TAATGTGGCCAGGCTCCTGCCGAGGGCAATTAAGTGAATGCAATCATATGCTTCCTATTCCTACTAATCCATTCCTGCAATCCCTGAATTTGAATAAAAT
AATGACCCTATCATATAAGGAAGATGAGTTTTGGGACAGGGTAAAATCTGTTAACACTATGCAAATGCATTAAATGCCAGAAATATCAGCAGATGTCTTC
||||||||||||||||||||||||||||||||||||||||||||||||||||||||||||||||||||||||||||||||||||||||||||||||||||
AATGACCCTATCATATAAGGAAGATGAGTTTTGGGACAGGGTAAAATCTGTTAACACTATGCAAATGCATTAAATGCCAGAAATATCAGCAGATGTCTTC
GAAATTACAAGAAAAAATTACTGTACATCACTAGAAATACACAGTTAATGAGAACCCAAACACGTTAGGTACACGTATATATGTCCAGTACCACAACGTC
||||||||||||||||||||||||||||||||||||||||||||||||||||||||||||||||||||||||||||||||||||||||||||||||||||
GAAATTACAAGAAAAAATTACTGTACATCACTAGAAATACACAGTTAATGAGAACCCAAACACGTTAGGTACACGTATATATGTCCAGTACCACAACGTC
ATTAGGTTCAGAGATATGGTACTTATTGTTTCCAAACAGATTGTGTCTAAAGGAACAACTGATTGAGCTAACAATTACCCACAGTTTGCAGGCTTGTTCC
||||||||||||||||||||||||||||||||||||||||||||||||||||||||||||||||||||||||||||||||||||||||||||||||||||
ATTAGGTTCAGAGATATGGTACTTATTGTTTCCAAACAGATTGTGTCTAAAGGAACAACTGATTGAGCTAACAATTACCCACAGTTTGCAGGCTTGTTCC
ATTACACCCCAGCAATGCTAATAATCAGATGGCTTAAGCATAAACGTCGAAATATCAACAGCCTGAGAGCCTGACTACTCCATAAAGCTCCAGATTTAAT
||||||||||||||||||||||||||||||||||||||||||||||||||||||||||||||||||||||||||||||||||||||||||||||||||||
ATTACACCCCAGCAATGCTAATAATCAGATGGCTTAAGCATAAACGTCGAAATATCAACAGCCTGAGAGCCTGACTACTCCATAAAGCTCCAGATTTAAT
CGACAAATATCAAGGACGTCGTGTATATGCGGGTGACGCATGGCATGACAACGGTATCAAAACTTTATAAAGGTGAATAAGACAATCCTCATCGCGCACC
||||||||||||||||||||||||||||||||||||||||||||||||||||||||||||||||||||||||||||||||||||||||||||||||||||
CGACAAATATCAAGGACGTCGTGTATATGCGGGTGACGCATGGCATGACAACGGTATCAAAACTTTATAAAGGTGAATAAGACAATCCTCATCGCGCACC
ATAAAACTAGTTCGTGCATAGACACAATTTCTTCAGGTCTGTAGAGAGGTAGGTTAACAGAATAATAGAAAAGGGTCCAGTATATGTCGGCAGTGGATCC
||||||||||||||||||||||||||||||||||||||||||||||||||||||||||||||||||||||||||||||||||||||||||||||||||||
ATAAAACTAGTTCGTGCATAGACACAATTTCTTCAGGTCTGTAGAGAGGTAGGTTAACAGAATAATAGAAAAGGGTCCAGTATATGTCGGCAGTGGATCC
CTTGGTTGCGGCTCATGTTATACATGATGTGTTGGATCCATTTACATCAACTGTTCCACTCACAATAGGCTACAACAATAGGCTAGTTCGGCCAGGTGCT
||||||||||||||||||||||||||||||||||||||||||||||||||||||||||||||||||||||||||||||||||||||||||||||||||||
CTTGGTTGCGGCTCATGTTATACATGATGTGTTGGATCCATTTACATCAACTGTTCCACTCACAATAGGCTACAACAATAGGCTAGTTCGGCCAGGTGCT
GAGTTAAAACCATCTGCAGTTGTAAGCCAGCCACGAGTTGATATTGGTGGCAATGACATGAGAGTTCTCTACACCCTGGTAAGCTTCTAACCGGATAAGT
||||||||||||||||||||||||||||||||||||||||||||||||||||||||||||||||||||||||||||||||||||||||||||||||||||
GAGTTAAAACCATCTGCAGTTGTAAGCCAGCCACGAGTTGATATTGGTGGCAATGACATGAGAGTTCTCTACACCCTGGTAAGCTTCTAACCGGATAAGT
GGACGGTATACGCCGATTCTTGAGTTATCTAATCTAACTCTTTGACATTGTTTATTAGTATTATTGTAAAGAATTTCGTAGAAACAAAAGTTCTCACGTA
||||||||||||||||||||||||||||||||||||||||||||||||||||||||||||||||||||||||||||||||||||||||||||||||||||
GGACGGTATACGCCGATTCTTGAGTTATCTAATCTAACTCTTTGACATTGTTTATTAGTATTATTGTAAAGAATTTCGTAGAAACAAAAGTTCTCACGTA
CGTTTAATTTTCTCTTAGCAAACTCAAACATAATTATATGTTAACTTGTTTTGAGTTTGTCTTTGACTTGAAAAGATGCTGGTGGATCCAGACGCCCCAA
||||||||||||||||||||||||||||||||||||||||||||||||||||||||||||||||||||||||||||||||||||||||||||||||||||
CGTTTAATTTTCTCTTAGCAAACTCAAACATAATTATATGTTAACTTGTTTTGAGTTTGTCTTTGACTTGAAAAGATGCTGGTGGATCCAGACGCCCCAA
GCCCAAGTCACCCGTCACTAAGGGAGTACTTGCACTGGTAAATGAAATGCAACATATTCTTAATTCTATTCAATTTCTAAACCCTCCACCCATTTGTTTT
||||||||||||||||||||||||||||||||||||||||||||||||||||||||||||||||||||||||||||||||||||||||||||||||||||
GCCCAAGTCACCCGTCACTAAGGGAGTACTTGCACTGGTAAATGAAATGCAACATATTCTTAATTCTATTCAATTTCTAAACCCTCCACCCATTTGTTTT
CTGCCCCGGCAATTTAGATATATGTTTCCTATTTAATTTGATGTCATTATGATATATCTCCACTCCTGTGTAGGATGGTGACAGACATCCCTGGAACAAC
||||||||||||||||||||||||||||||||||||||||||||||||||||||||||||||||||||||||||||||||||||||||||||||||||||
CTGCCCCGGCAATTTAGATATATGTTTCCTATTTAATTTGATGTCATTATGATATATCTCCACTCCTGTGTAGGATGGTGACAGACATCCCTGGAACAAC
TGGTGTCAACTTTGGTATGATGTCATGCATAACAATTTGTTGCATGTGGTTTGCAGTCTTAGCTGAATTTATGGATGTGGAAATTTGCCCATGTTAAGTT
||||||||||||||||||||||||||||||||||||||||||||||||||||||||||||||||||||||||||||||||||||||||||||||||||||
TGGTGTCAACTTTGGTATGATGTCATGCATAACAATTTGTTGCATGTGGTTTGCAGTCTTAGCTGAATTTATGGATGTGGAAATTTGCCCATGTTAAGTT
CTTTACCTCAAAACTGTTGCCTGTCATAGAAATAACAGAGAGGGCTATCACTTAAATCAGGTACAAGTAAATTCAAATTAGCTCATCAATCATCATGAAC
||||||||||||||||||||||||||||||||||||||||||||||||||||||||||||||||||||||||||||||||||||||||||||||||||||
CTTTACCTCAAAACTGTTGCCTGTCATAGAAATAACAGAGAGGGCTATCACTTAAATCAGGTACAAGTAAATTCAAATTAGCTCATCAATCATCATGAAC
AATTGGATATCTTAATACTAGACAAAGAAGCTAGCGATAAATGGCATACATATGTTCTAAACATTTTGGAAATCTTTAAGAAAAATACATTATACGGCCT
||||||||||||||||||||||||||||||||||||||||||||||||||||||||||||||||||||||||||||||||||||||||||||||||||||
AATTGGATATCTTAATACTAGACAAAGAAGCTAGCGATAAATGGCATACATATGTTCTAAACATTTTGGAAATCTTTAAGAAAAATACATTATACGGCCT
AAAGAAACAGGGACTCTAAAATAGTGGTACACTTCAGGAACATATTTTCCTATCTATCAGTTTTCACATGTACAAAACCAAACTCACGCAATGAATTATT
||||||||||||||||||||||||||||||||||||||||||||||||||||||||||||||||||||||||||||||||||||||||||||||||||||
AAAGAAACAGGGACTCTAAAATAGTGGTACACTTCAGGAACATATTTTCCTATCTATCAGTTTTCACATGTACAAAACCAAACTCACGCAATGAATTATT
ATAACTCAACGTGAATTACTCTTTAAATTCTTAACTAGATATCTCCACCCAGTTGAATTACTCTTTTTCAATAATATGGCTGCATGCATCTTCATGATGC
||||||||||||||||||||||||||||||||||||||||||||||||||||||||||||||||||||||||||||||||||||||||||||||||||||
ATAACTCAACGTGAATTACTCTTTAAATTCTTAACTAGATATCTCCACCCAGTTGAATTACTCTTTTTCAATAATATGGCTGCATGCATCTTCATGATGC
AGAGGCCGGGGGCATGCCTCCATTTCCAAAAAAAAAAGTTGAATTACTCTTTAAATTCTTAACTACTCCCTCCGTTCGGAATTATTTGTCGCAGAAATGG
||||||||||||||||||||||||||||||||||||||||||||||||||||||||||||||||||||||||||||||||||||||||||||||||||||
AGAGGCCGGGGGCATGCCTCCATTTCCAAAAAAAAAAGTTGAATTACTCTTTAAATTCTTAACTACTCCCTCCGTTCGGAATTATTTGTCGCAGAAATGG
ATGTATCTAGACGTATTTTAGTTCTAGATACATCAATTTCCGAGACAAGTAATTCCGAACGGAGGGAGTAGCTAATAACTAATGCTCTTGCAGGCCAAGA
||||||||||||||||||||||||||||||||||||||||||||||||||||||||||||||||||||||||||||||||||||||||||||||||||||
ATGTATCTAGACGTATTTTAGTTCTAGATACATCAATTTCCGAGACAAGTAATTCCGAACGGAGGGAGTAGCTAATAACTAATGCTCTTGCAGGCCAAGA
GCTTGTAATTTATGAAAGGCCGGAGCCAAGATCTGGCATCCACCGGATGGTATTTGTGCTGTTCCAGCAACTAGGTAGGGGGACGGTTTTTGCACCGGAC
||||||||||||||||||||||||||||||||||||||||||||||||||||||||||||||||||||||||||||||||||||||||||||||||||||
GCTTGTAATTTATGAAAGGCCGGAGCCAAGATCTGGCATCCACCGGATGGTATTTGTGCTGTTCCAGCAACTAGGTAGGGGGACGGTTTTTGCACCGGAC
ATGCGGCAGAACTTCAGCTCCAGGAACTTCGCACGCCAGTACCACCTCAACATTGCCGCTGCCACATATTTCAACTGTCAAAGGGAAGGTGGATCGGGCG
||||||||||||||||||||||||||||||||||||||||||||||||||||||||||||||||||||||||||||||||||||||||||||||||||||
ATGCGGCAGAACTTCAGCTCCAGGAACTTCGCACGCCAGTACCACCTCAACATTGCCGCTGCCACATATTTCAACTGTCAAAGGGAAGGTGGATCGGGCG
GAAGAAGGTTTAGGCCAGAAAGTTCTCAAGGGTAGTAGAGACTATGCACTACGGAGTACAGACCGTTATATGACTGTACCGTGGTGTTGCATCACCACAT
||||||||||||||||||||||||||||||||||||||||||||||||||||||||||||||||||||||||||||||||||||||||||||||||||||
GAAGAAGGTTTAGGCCAGAAAGTTCTCAAGGGTAGTAGAGACTATGCACTACGGAGTACAGACCGTTATATGACTGTACCGTGGTGTTGCATCACCACAT
AGGATACTGTATCACCCATATATGCAGGGTATGTATCTTCAGCACATTATCTACAAAAACTATGTATCTTCAGCTATGACGAGCATGATGGAATATACCT
||||||||||||||||||||||||||||||||||||||||||||||||||||||||||||||||||||||||||||||||||||||||||||||||||||
AGGATACTGTATCACCCATATATGCAGGGTATGTATCTTCAGCACATTATCTACAAAAACTATGTATCTTCAGCTATGACGAGCATGATGGAATATACCT
CGGTTATTATTTAATCATGCAAAGTATTAGCAATGTTTGTTAACCTCAAATCACTGCTAGTTCTAAGCACCTGCTCTGCATTTAAACATAGTCTCTATCA
||||||||||||||||||||||||||||||||||||||||||||||||||||||||||||||||||||||||||||||||||||||||||||||||||||
CGGTTATTATTTAATCATGCAAAGTATTAGCAATGTTTGTTAACCTCAAATCACTGCTAGTTCTAAGCACCTGCTCTGCATTTAAACATAGTCTCTATCA
ACACATTATCTACAAAAACTATGTATCTTCAGCTATGACGAGCTTGATGGAATATACGTCGATTATTATTTAATCATGCAAAGTATTCACAGTGTTTGCC
||||||||||||||||||||||||||||||||||||||||||||||||||||||||||||||||||||||||||||||||||||||||||||||||||||
ACACATTATCTACAAAAACTATGTATCTTCAGCTATGACGAGCTTGATGGAATATACGTCGATTATTATTTAATCATGCAAAGTATTCACAGTGTTTGCC
AATCTCAAATCACTGCTAGTTCTAAGCACCTGCTCTGCATTTAAACAGGTTGTGCTGAATAAAATCAACACGATCCAAATCATCAAAAATATTTTCGATA
||||||||||||||||||||||||||||||||||||||||||||||||||||||||||||||||||||||||||||||||||||||||||||||||||||
AATCTCAAATCACTGCTAGTTCTAAGCACCTGCTCTGCATTTAAACAGGTTGTGCTGAATAAAATCAACACGATCCAAATCATCAAAAATATTTTCGATA
GCAAACCAAGATTTGTTTAGATGGAAGGTATTATCGACGGATCGACAAACTTTGCATCGTAAGTCCCAGAAGTGGCAAGGTAATTAAACGCAAGGTAGCT
||||||||||||||||||||||||||||||||||||||||||||||||||||||||||||||||||||||||||||||||||||||||||||||||||||
GCAAACCAAGATTTGTTTAGATGGAAGGTATTATCGACGGATCGACAAACTTTGCATCGTAAGTCCCAGAAGTGGCAAGGTAATTAAACGCAAGGTAGCT
CAAATATTTATGCCGGTCAGCTCGCCCGTGGTCCCCGACGACGACTTTACTGGCTCCGACTACGACACGACGCCGGACCCCGCGCCGGTGCATACCGGCC
||||||||||||||||||||||||||||||||||||||||||||||||||||||||||||||||||||||||||||||||||||||||||||||||||||
CAAATATTTATGCCGGTCAGCTCGCCCGTGGTCCCCGACGACGACTTTACTGGCTCCGACTACGACACGACGCCGGACCCCGCGCCGGTGCATACCGGCC
TATGACGCAGGTCTCATGGAGCAGGAGCAGGCGCACTATGACGTCGTCAAGGCGGAGGAGGAGCAGCCTACCCCAGCTCCTATGTCGGCATTTTGGCAAG
||||||||||||||||||||||||||||||||||||||||||||||||||||||||||||||||||||||||||||||||||||||||||||||||||||
TATGACGCAGGTCTCATGGAGCAGGAGCAGGCGCACTATGACGTCGTCAAGGCGGAGGAGGAGCAGCCTACCCCAGCTCCTATGTCGGCATTTTGGCAAG
CGTAGGAGGAGCAGAGGTACAACATGTTCCTCCTCGTGAAGCACTGGCTGGCAGAGGGTCAAATCTACGGCGAGCGCACAAGCGAGGCAGCCGTGTCTGC
||||||||||||||||||||||||||||||||||||||||||||||||||||||||||||||||||||||||||||||||||||||||||||||||||||
CGTAGGAGGAGCAGAGGTACAACATGTTCCTCCTCGTGAAGCACTGGCTGGCAGAGGGTCAAATCTACGGCGAGCGCACAAGCGAGGCAGCCGTGTCTGC
CATGGCCATGGCGAATCCGAACTTCGTTGCGTAGCAGCAGGCGCTCTACGAGGCCGCGCGCACACAAGGCGTCGCTCGCAAAGCAGAGCCGGCGCAGCCA
||||||||||||||||||||||||||||||||||||||||||||||||||||||||||||||||||||||||||||||||||||||||||||||||||||
CATGGCCATGGCGAATCCGAACTTCGTTGCGTAGCAGCAGGCGCTCTACGAGGCCGCGCGCACACAAGGCGTCGCTCGCAAAGCAGAGCCGGCGCAGCCA
GACACGGACGTGGCCGCACACGCGCCGGCGGCACAGATGGTCCAGGACTCCAACGCAACCAATTGCGCGTCATATGCGCCGCCGTCCAACTTTTGGGGGC
||||||||||||||||||||||||||||||||||||||||||||||||||||||||||||||||||||||||||||||||||||||||||||||||||||
GACACGGACGTGGCCGCACACGCGCCGGCGGCACAGATGGTCCAGGACTCCAACGCAACCAATTGCGCGTCATATGCGCCGCCGTCCAACTTTTGGGGGC
GCACGTGGCAGGTGGGCGGTGATGGACCGTCCACGTTCATCATCGACCTCACATCCACCGGCACCGGCGACGGACAAGTCGCATACTCCTCCGTGGCTGA
||||||||||||||||||||||||||||||||||||||||||||||||||||||||||||||||||||||||||||||||||||||||||||||||||||
GCACGTGGCAGGTGGGCGGTGATGGACCGTCCACGTTCATCATCGACCTCACATCCACCGGCACCGGCGACGGACAAGTCGCATACTCCTCCGTGGCTGA
GTAGGGCATGGGATGCGGCAGGGCATTGTGTCCCAAGTGGGCCGGTCTGTCTCCTATGTTCTATTATTCCTCGCTGGAGATCGTACCTCCACTTCACAGG
||||||||||||||||||||||||||||||||||||||||||||||||||||||||||||||||||||||||||||||||||||||||||||||||||||
GTAGGGCATGGGATGCGGCAGGGCATTGTGTCCCAAGTGGGCCGGTCTGTCTCCTATGTTCTATTATTCCTCGCTGGAGATCGTACCTCCACTTCACAGG
TCTTAACCCCGCTGCCGTACATGAAGACGACAGATGGAGGATGTGGTCGCCTATGCAAAATACAAAGGAAGTGAACGACGGGCCGCCGTCGTAGGATAGG
||||||||||||||||||||||||||||||||||||||||||||||||||||||||||||||||||||||||||||||||||||||||||||||||||||
TCTTAACCCCGCTGCCGTACATGAAGACGACAGATGGAGGATGTGGTCGCCTATGCAAAATACAAAGGAAGTGAACGACGGGCCGCCGTCGTAGGATAGG
TTAGGGTTGGATTTATTTTTTTATCCTAAATGTTCGAAATGTATTGAAAATCTGCCTATTTCCATGAATCGTGTCCGATAGTCAAGTACACTGCCAAGCC
||||||||||||||||||||||||||||||||||||||||||||||||||||||||||||||||||||||||||||||||||||||||||||||||||||
TTAGGGTTGGATTTATTTTTTTATCCTAAATGTTCGAAATGTATTGAAAATCTGCCTATTTCCATGAATCGTGTCCGATAGTCAAGTACACTGCCAAGCC
ACCACAAAACCGTGTCCAAGAATTTGCACAAGTTCTCTACCATTTCATCACTGGTACCAATCCAGACCGTACAAGGATGATACTATGACGATTTACCGCA
||||||||||||||||||||||||||||||||||||||||||||||||||||||||||||||||||||||||||||||||||||||||||||||||||||
ACCACAAAACCGTGTCCAAGAATTTGCACAAGTTCTCTACCATTTCATCACTGGTACCAATCCAGACCGTACAAGGATGATACTATGACGATTTACCGCA
GCGACAGCATTGCTTAACAGCTAAGGCTTCCCTTCATATTTTACTTTTTTGAAGCCCTGATCGCAGAACAATTGTTCTACGGTGGTTTTTGTTAATAAAA
||||||||||||||||||||||||||||||||||||||||||||||||||||||||||||||||||||||||||||||||||||||||||||||||||||
GCGACAGCATTGCTTAACAGCTAAGGCTTCCCTTCATATTTTACTTTTTTGAAGCCCTGATCGCAGAACAATTGTTCTACGGTGGTTTTTGTTAATAAAA
CAAAGTTATGTGCGAAGCTAAGCCTTTCCTTTTCTTTTTCTTTTCGAAGCCCTGACCGTAGAACAATTGCAAAACAATGTTATGTAGAAAGCAAAGCTAA
||||||||||||||||||||||||||||||||||||||||||||||||||||||||||||||||||||||||||||||||||||||||||||||||||||
CAAAGTTATGTGCGAAGCTAAGCCTTTCCTTTTCTTTTTCTTTTCGAAGCCCTGACCGTAGAACAATTGCAAAACAATGTTATGTAGAAAGCAAAGCTAA
GCCTTTCCTCACAGAGCTTAGCTAGTAAATTTGAGTTTGGCTCCCTTCTGCCTTGCTAGCTAAGTTATGCATACATCCTCGATATTTTCCGACAAACTAT
||||||||||||||||||||||||||||||||||||||||||||||||||||||||||||||||||||||||||||||||||||||||||||||||||||
GCCTTTCCTCACAGAGCTTAGCTAGTAAATTTGAGTTTGGCTCCCTTCTGCCTTGCTAGCTAAGTTATGCATACATCCTCGATATTTTCCGACAAACTAT
CAGCTACTACAGCACTTTCTTGTCCTGCTGACAAGCATGCAGCATCCAGCTGCCTAGCTTGCCTCTCTTCAAACTCAACTTGAACTTCTTCTCTCGTTCC
||||||||||||||||||||||||||||||||||||||||||||||||||||||||||||||||||||||||||||||||||||||||||||||||||||
CAGCTACTACAGCACTTTCTTGTCCTGCTGACAAGCATGCAGCATCCAGCTGCCTAGCTTGCCTCTCTTCAAACTCAACTTGAACTTCTTCTCTCGTTCC
TTGGCTCTTAATTCATTTCATTAACGAGTAATACCGGCCAAGCCAGCCAGAGGTTTCATGCAGCGTACGTGTAGCTCTTGTACAGCCAGCCAGCCATCAT
||||||||||||||||||||||||||||||||||||||||||||||||||||||||||||||||||||||||||||||||||||||||||||||||||||
TTGGCTCTTAATTCATTTCATTAACGAGTAATACCGGCCAAGCCAGCCAGAGGTTTCATGCAGCGTACGTGTAGCTCTTGTACAGCCAGCCAGCCATCAT
GCATGCCTGCATTAGTCAAAACCAATTCAATTTGACTTCTTCTCTCTTGCATGCATCTCTCAATTGCATGCGTTTAACCATAATGACAGGATTAATTCTA
||||||||||||||||||||||||||||||||||||||||||||||||||||||||||||||||||||||||||||||||||||||||||||||||||||
GCATGCCTGCATTAGTCAAAACCAATTCAATTTGACTTCTTCTCTCTTGCATGCATCTCTCAATTGCATGCGTTTAACCATAATGACAGGATTAATTCTA
ACATGTTCTAGTCTTCCAGTAATCATAAAGCTCAGTATCTAATCGGCAAATATCAAGGCCGGCCGTCATGTGTGCAAAGGTGACGCATGACATGGCACTG
||||||||||||||||||||||||||||||||||||||||||||||||||||||||||||||||||||||||||||||||||||||||||||||||||||
ACATGTTCTAGTCTTCCAGTAATCATAAAGCTCAGTATCTAATCGGCAAATATCAAGGCCGGCCGTCATGTGTGCAAAGGTGACGCATGACATGGCACTG
GTATCATGGAGTCGCTTTCGTCCTGGAGATATTCATTCTCATCACTTGGGATGGGTCATGACTTGAATGACTTGATAGTTCCATTGAGAACCCAAACATG
||||||||||||||||||||||||||||||||||||||||||||||||||||||||||||||||||||||||||||||||||||||||||||||||||||
GTATCATGGAGTCGCTTTCGTCCTGGAGATATTCATTCTCATCACTTGGGATGGGTCATGACTTGAATGACTTGATAGTTCCATTGAGAACCCAAACATG
TTACCACCCGAGCAATGAGAACCCAAACATGTTACCTAAACCCCCATCCCAGGAATAATGGCTTGAGCATCGACGTACGTTCAACAGCCTGGGAGCCTCA
||||||||||||||||||||||||||||||||||||||||||||||||||||||||||||||||||||||||||||||||||||||||||||||||||||
TTACCACCCGAGCAATGAGAACCCAAACATGTTACCTAAACCCCCATCCCAGGAATAATGGCTTGAGCATCGACGTACGTTCAACAGCCTGGGAGCCTCA
TCGCGAGCCATACAAGTAGTTGATGCATAGAAACAACAATCCTTCAGCCGGTCCATGAAGAGCTAGGTCAACAACACAATAACAGAAATCTAGCATGTGG
||||||||||||||||||||||||||||||||||||||||||||||||||||||||||||||||||||||||||||||||||||||||||||||||||||
TCGCGAGCCATACAAGTAGTTGATGCATAGAAACAACAATCCTTCAGCCGGTCCATGAAGAGCTAGGTCAACAACACAATAACAGAAATCTAGCATGTGG
GCGGTGGATCCCTTGGTTGTGGCTCGAGTCATACACGATGTGTTGGATCCGTTTACATCAACTGTCCCACTCAGCATAGGCTACAACAACAGGCTACTTC
||||||||||||||||||||||||||||||||||||||||||||||||||||||||||||||||||||||||||||||||||||||||||||||||||||
GCGGTGGATCCCTTGGTTGTGGCTCGAGTCATACACGATGTGTTGGATCCGTTTACATCAACTGTCCCACTCAGCATAGGCTACAACAACAGGCTACTTC
TGCGAGGCGCTGAGCTGAGACCATCTGCGGTCGTAAGCAAGCCGCGAGTCGACGTCGGTGGCAATGACATGAGAGTTCTCTACATAGGTACGTCGAGTTT
||||||||||||||||||||||||||||||||||||||||||||||||||||||||||||||||||||||||||||||||||||||||||||||||||||
TGCGAGGCGCTGAGCTGAGACCATCTGCGGTCGTAAGCAAGCCGCGAGTCGACGTCGGTGGCAATGACATGAGAGTTCTCTACATAGGTACGTCGAGTTT
TGTTAGGGTTTGTGTCCTGCTCACGAAGACGAGACGACGTCGGTTAAATGAAGACAAGACGATGTCGGTTCCCTGAAGATGGAATAATTAAGGTGATCAC
||||||||||||||||||||||||||||||||||||||||||||||||||||||||||||||||||||||||||||||||||||||||||||||||||||
TGTTAGGGTTTGTGTCCTGCTCACGAAGACGAGACGACGTCGGTTAAATGAAGACAAGACGATGTCGGTTCCCTGAAGATGGAATAATTAAGGTGATCAC
CACCTAGCCCTCGTCCTGGTGGAGGTGTGTTTTCAACGGATTTGTCCTTGGTGGATTTGCTCGGATCTAGTCGTAGTCCGTCTACATTGGTGTGTCTTCA
||||||||||||||||||||||||||||||||||||||||||||||||||||||||||||||||||||||||||||||||||||||||||||||||||||
CACCTAGCCCTCGTCCTGGTGGAGGTGTGTTTTCAACGGATTTGTCCTTGGTGGATTTGCTCGGATCTAGTCGTAGTCCGTCTACATTGGTGTGTCTTCA
GGTTGGATCCTTTCGATCTACGCTACTCTTCATCAGTGGCGGTTGCTGTTCTGCTGCGCTGGTTCTATGGGGCCTTAGCATGACTACTTCTCGACTGTCT
||||||||||||||||||||||||||||||||||||||||||||||||||||||||||||||||||||||||||||||||||||||||||||||||||||
GGTTGGATCCTTTCGATCTACGCTACTCTTCATCAGTGGCGGTTGCTGTTCTGCTGCGCTGGTTCTATGGGGCCTTAGCATGACTACTTCTCGACTGTCT
GCTACAAAAGTTATTGCCCGACTCCGGCGAGTGAGGGGCGATGATGGCGGCGCATCTTCGGCTCGCTTCAGTGCTTGTAGTCATCGCTAGGTGGTCTATG
||||||||||||||||||||||||||||||||||||||||||||||||||||||||||||||||||||||||||||||||||||||||||||||||||||
GCTACAAAAGTTATTGCCCGACTCCGGCGAGTGAGGGGCGATGATGGCGGCGCATCTTCGGCTCGCTTCAGTGCTTGTAGTCATCGCTAGGTGGTCTATG
GATCTAGACTTATTATTATTATTTTTAGTGTTTGTTGCACTGCCATGATTGAAGATGAATAGAAATGAAAGTTTTCTATTCATGAATCATCGGGGTATTT
||||||||||||||||||||||||||||||||||||||||||||||||||||||||||||||||||||||||||||||||||||||||||||||||||||
GATCTAGACTTATTATTATTATTTTTAGTGTTTGTTGCACTGCCATGATTGAAGATGAATAGAAATGAAAGTTTTCTATTCATGAATCATCGGGGTATTT
ATTCGCATAGCCCCAGCTCGAGGCACGATTGGGGAGCAACAATCAACACTTATGCATACACATAGATCTTCTCTCTTCAAAGATTGAATGCCCGAAACTC
||||||||||||||||||||||||||||||||||||||||||||||||||||||||||||||||||||||||||||||||||||||||||||||||||||
ATTCGCATAGCCCCAGCTCGAGGCACGATTGGGGAGCAACAATCAACACTTATGCATACACATAGATCTTCTCTCTTCAAAGATTGAATGCCCGAAACTC
CCTACAAAACATTGAATACCCGAAATGCCACACAAAAATTACACAGATATTTTCGGGCCTAACGTAGATTTAGACCCTGCTTGTCACAAACAATTGACAT
||||||||||||||||||||||||||||||||||||||||||||||||||||||||||||||||||||||||||||||||||||||||||||||||||||
CCTACAAAACATTGAATACCCGAAATGCCACACAAAAATTACACAGATATTTTCGGGCCTAACGTAGATTTAGACCCTGCTTGTCACAAACAATTGACAT
AAGTAATTCCAATTATTATTATTTAAAAAAAACTGGAGTAACTTTGAGATGGCCATAGTATTCTTTATTGTTTCCGTTCCTAATAAAGAGGTAAAGGGTC
||||||||||||||||||||||||||||||||||||||||||||||||||||||||||||||||||||||||||||||||||||||||||||||||||||
AAGTAATTCCAATTATTATTATTTAAAAAAAACTGGAGTAACTTTGAGATGGCCATAGTATTCTTTATTGTTTCCGTTCCTAATAAAGAGGTAAAGGGTC
AAGTTTAAGATGCAAAAGATGATTATTGTCCTCTCAATAAAGATTGATGCAAAAAGATAGATGAGACATGAGATATTCATATTGTTCTCCTATCTAATCT
||||||||||||||||||||||||||||||||||||||||||||||||||||||||||||||||||||||||||||||||||||||||||||||||||||
AAGTTTAAGATGCAAAAGATGATTATTGTCCTCTCAATAAAGATTGATGCAAAAAGATAGATGAGACATGAGATATTCATATTGTTCTCCTATCTAATCT
TATGCCTTCTGCACAAAAGGGAGATGCTCTCCTATCTTCTGGACGAAAGAGATGCTTCTATTTTCATGTTCGAGTCGGCCGAGGAAATGCAAGGAGCACA
||||||||||||||||||||||||||||||||||||||||||||||||||||||||||||||||||||||||||||||||||||||||||||||||||||
TATGCCTTCTGCACAAAAGGGAGATGCTCTCCTATCTTCTGGACGAAAGAGATGCTTCTATTTTCATGTTCGAGTCGGCCGAGGAAATGCAAGGAGCACA
GCTCTTCTTTGGCTGATGAGATGTCTGATAATCAAATCAGATACTTTGCAAAGCATTATCAAGCCCTTTAAAGATTTGCAGCAGTTCATTTCTTCCTGAA
||||||||||||||||||||||||||||||||||||||||||||||||||||||||||||||||||||||||||||||||||||||||||||||||||||
GCTCTTCTTTGGCTGATGAGATGTCTGATAATCAAATCAGATACTTTGCAAAGCATTATCAAGCCCTTTAAAGATTTGCAGCAGTTCATTTCTTCCTGAA
ATATTCGTTCCGATCACTTGGGTCATCTGGACGACCAGGAATGACTTCATGTTTCAAAGAATTCAGCCAAGTGTCTACATGTGCAGGAAGAAATTCAATC
||||||||||||||||||||||||||||||||||||||||||||||||||||||||||||||||||||||||||||||||||||||||||||||||||||
ATATTCGTTCCGATCACTTGGGTCATCTGGACGACCAGGAATGACTTCATGTTTCAAAGAATTCAGCCAAGTGTCTACATGTGCAGGAAGAAATTCAATC
TCCATAGAGCAAAGAGGAAGAATTGTTCCTAGCTTCCTGCCCGGGTTGCAGCATTCCACCAACTGAATGAACGACTGCTACGGCTCTGCGTCGTCGTATC
||||||||||||||||||||||||||||||||||||||||||||||||||||||||||||||||||||||||||||||||||||||||||||||||||||
TCCATAGAGCAAAGAGGAAGAATTGTTCCTAGCTTCCTGCCCGGGTTGCAGCATTCCACCAACTGAATGAACGACTGCTACGGCTCTGCGTCGTCGTATC
CAGAGGGAGAATAAGGATGGATGAGGGGAAGGGGACTTGCTAGATCTTCTCTCTTCAAAGATTGAATGCCCCTAACTCCCTGCAAAACATCAAATACCCA
||||||||||||||||||||||||||||||||||||||||||||||||||||||||||||||||||||||||||||||||||||||||||||||||||||
CAGAGGGAGAATAAGGATGGATGAGGGGAAGGGGACTTGCTAGATCTTCTCTCTTCAAAGATTGAATGCCCCTAACTCCCTGCAAAACATCAAATACCCA
AAACTCTGCACAAAAATTACATAAATATTTTCAAACCTTACAGACTACCTAACATAAATTTAGATCTCCGGCTGGTCACAAACATTTGACATAAGTATTT
||||||||||||||||||||||||||||||||||||||||||||||||||||||||||||||||||||||||||||||||||||||||||||||||||||
AAACTCTGCACAAAAATTACATAAATATTTTCAAACCTTACAGACTACCTAACATAAATTTAGATCTCCGGCTGGTCACAAACATTTGACATAAGTATTT
TGTATAGAAAAATGAATATATATTTGTAATTATGAGATGGTCATGATATTCTATTTTTTTCCCTTCCCCAAAAGTGTAAGGCCAAGGTTAGGATGCAAAA
||||||||||||||||||||||||||||||||||||||||||||||||||||||||||||||||||||||||||||||||||||||||||||||||||||
TGTATAGAAAAATGAATATATATTTGTAATTATGAGATGGTCATGATATTCTATTTTTTTCCCTTCCCCAAAAGTGTAAGGCCAAGGTTAGGATGCAAAA
GATAAATATTGCCCTCTCAATAAAGATCAATGCAAGATAGATGGATCGGATATTCCGATTGTTCTATACTATCTAAATCTTATGCCTTCTGTACAAAAGA
||||||||||||||||||||||||||||||||||||||||||||||||||||||||||||||||||||||||||||||||||||||||||||||||||||
GATAAATATTGCCCTCTCAATAAAGATCAATGCAAGATAGATGGATCGGATATTCCGATTGTTCTATACTATCTAAATCTTATGCCTTCTGTACAAAAGA
GATGCTTCTTTCTTCTGAACACGAGAGATGCTTCTCCTTTCATGTCCGTGTTGCCGAGGATATGTAAGGAGCATGGCTCTTCTTTCGCTGATGGGACATC
||||||||||||||||||||||||||||||||||||||||||||||||||||||||||||||||||||||||||||||||||||||||||||||||||||
GATGCTTCTTTCTTCTGAACACGAGAGATGCTTCTCCTTTCATGTCCGTGTTGCCGAGGATATGTAAGGAGCATGGCTCTTCTTTCGCTGATGGGACATC
TGATAATCAAATCACATATACTTTGCAAAGCATCATTGAGCACTTTAATGATTTGCAGCCAGTTCCTTTCTTCCAGGGGATATTCTTTCTGGTCACTTGG
||||||||||||||||||||||||||||||||||||||||||||||||||||||||||||||||||||||||||||||||||||||||||||||||||||
TGATAATCAAATCACATATACTTTGCAAAGCATCATTGAGCACTTTAATGATTTGCAGCCAGTTCCTTTCTTCCAGGGGATATTCTTTCTGGTCACTTGG
GTCATTTGGAGTTCATATTTCAAAGGATTCAGCCTATGTTGCAGCATTCCACTAACTCAATGAACTGCTGGGGATTTCTTTTTTCTCTTCTTTCAATTTA
||||||||||||||||||||||||||||||||||||||||||||||||||||||||||||||||||||||||||||||||||||||||||||||||||||
GTCATTTGGAGTTCATATTTCAAAGGATTCAGCCTATGTTGCAGCATTCCACTAACTCAATGAACTGCTGGGGATTTCTTTTTTCTCTTCTTTCAATTTA
AGTATATAGCTCCCTTTTCTTCTTTGTACTTCGCTTATTTAAAAATAAATATACCCAGTAGGAGAAATCCTGCTGTTTTCCTGCAAAAAAAAACCCAGAT
||||||||||||||||||||||||||||||||||||||||||||||||||||||||||||||||||||||||||||||||||||||||||||||||||||
AGTATATAGCTCCCTTTTCTTCTTTGTACTTCGCTTATTTAAAAATAAATATACCCAGTAGGAGAAATCCTGCTGTTTTCCTGCAAAAAAAAACCCAGAT
ATTTTGCAGCTACACAAGCACTACAACTCACAGGGCATGTACAAGTCAAATGGTATTTTGTTGATTGGGCTGCTGGTTCTGTATACTTTTTGCTAACATG
||||||||||||||||||||||||||||||||||||||||||||||||||||||||||||||||||||||||||||||||||||||||||||||||||||
ATTTTGCAGCTACACAAGCACTACAACTCACAGGGCATGTACAAGTCAAATGGTATTTTGTTGATTGGGCTGCTGGTTCTGTATACTTTTTGCTAACATG
CATTAAATGATAGGATATTTGCTGTGAAATAGGTACGCAGCATAAAACCATTAAGTACTTGAGATGACACTTAAAAAAATATCATTTTTCTATTCACAAA
||||||||||||||||||||||||||||||||||||||||||||||||||||||||||||||||||||||||||||||||||||||||||||||||||||
CATTAAATGATAGGATATTTGCTGTGAAATAGGTACGCAGCATAAAACCATTAAGTACTTGAGATGACACTTAAAAAAATATCATTTTTCTATTCACAAA
TCTAGGTGACATCGGGGATAAAGCCTTCTAAGGTATATATTCATATTTCACCCCCCATGTGAAGAAGGAACAAATTGGTTCTACTTTGCTATCACAGAGT
||||||||||||||||||||||||||||||||||||||||||||||||||||||||||||||||||||||||||||||||||||||||||||||||||||
TCTAGGTGACATCGGGGATAAAGCCTTCTAAGGTATATATTCATATTTCACCCCCCATGTGAAGAAGGAACAAATTGGTTCTACTTTGCTATCACAGAGT
GGTAAATAGAATACTTATCATTGAGTGAACAAAGTTGCAGCCAACTTTCGAATCTACAGCTGCAAAACATTAACCTCCATGCATATGCCATATTGGTTGG
||||||||||||||||||||||||||||||||||||||||||||||||||||||||||||||||||||||||||||||||||||||||||||||||||||
GGTAAATAGAATACTTATCATTGAGTGAACAAAGTTGCAGCCAACTTTCGAATCTACAGCTGCAAAACATTAACCTCCATGCATATGCCATATTGGTTGG
TTGGACATTGTAGTGATGCCACGTATGGGTAGAAATGTAAGTGACCAACCGAAGATAGATTATGTGAACATATGTTCATATGGAGTTTTTTACCCAAAAC
||||||||||||||||||||||||||||||||||||||||||||||||||||||||||||||||||||||||||||||||||||||||||||||||||||
TTGGACATTGTAGTGATGCCACGTATGGGTAGAAATGTAAGTGACCAACCGAAGATAGATTATGTGAACATATGTTCATATGGAGTTTTTTACCCAAAAC
CACCACACTTGGGGCTAGGGTAACAACTTAGTACCACTTTTCGGACAAGGTACAAAAAACCACCAATTCTGTGCCTAATGACTAACGGGGAGCACTGATT
||||||||||||||||||||||||||||||||||||||||||||||||||||||||||||||||||||||||||||||||||||||||||||||||||||
CACCACACTTGGGGCTAGGGTAACAACTTAGTACCACTTTTCGGACAAGGTACAAAAAACCACCAATTCTGTGCCTAATGACTAACGGGGAGCACTGATT
GTCTGATAAGCACGCAAAAACAGCGAAACTGACAAGTTGGGCCCACCTGTCAGGCTGACATGGCATGCTTGTGTGGACAATATGCTGAGTTGTACAAGGG
||||||||||||||||||||||||||||||||||||||||||||||||||||||||||||||||||||||||||||||||||||||||||||||||||||
GTCTGATAAGCACGCAAAAACAGCGAAACTGACAAGTTGGGCCCACCTGTCAGGCTGACATGGCATGCTTGTGTGGACAATATGCTGAGTTGTACAAGGG
ACCCACGTGTAAGTGACTTATATGTTTGTCTCTTTTAAAATTCTTTTTCTTGTGGGCATTTCAACAAATCGGTAATGGGCGCTTATCAGCGCACTAGACG
||||||||||||||||||||||||||||||||||||||||||||||||||||||||||||||||||||||||||||||||||||||||||||||||||||
ACCCACGTGTAAGTGACTTATATGTTTGTCTCTTTTAAAATTCTTTTTCTTGTGGGCATTTCAACAAATCGGTAATGGGCGCTTATCAGCGCACTAGACG
TCGCCGGCATGGCTCACCGCCCTTGCTGGTCGATGGGCATCCGAGACCTGGCCGCCGCTGTCAAGCTGCTGGAGTTCCCTCGCCGCCCCTCTCTTTTTCT
||||||||||||||||||||||||||||||||||||||||||||||||||||||||||||||||||||||||||||||||||||||||||||||||||||
TCGCCGGCATGGCTCACCGCCCTTGCTGGTCGATGGGCATCCGAGACCTGGCCGCCGCTGTCAAGCTGCTGGAGTTCCCTCGCCGCCCCTCTCTTTTTCT
TCTCCTCCTTCCTCCCTGATTTCCCTCTCTCTCAAAAGGACCAACTGTCATTCCCGCCGCCATGGATGAACTCAACCTCGCCGCGCCGCCCCAACATCGC
||||||||||||||||||||||||||||||||||||||||||||||||||||||||||||||||||||||||||||||||||||||||||||||||||||
TCTCCTCCTTCCTCCCTGATTTCCCTCTCTCTCAAAAGGACCAACTGTCATTCCCGCCGCCATGGATGAACTCAACCTCGCCGCGCCGCCCCAACATCGC
CGAGCTACCCCAGCCCTCTGCTGCGCCTGGATCCAACGAGTTTCGGCCGGGAACTCGTGCTCGCGGCAACCGCCAGAGGCCCTGGTCGTGACCGTACAGA
||||||||||||||||||||||||||||||||||||||||||||||||||||||||||||||||||||||||||||||||||||||||||||||||||||
CGAGCTACCCCAGCCCTCTGCTGCGCCTGGATCCAACGAGTTTCGGCCGGGAACTCGTGCTCGCGGCAACCGCCAGAGGCCCTGGTCGTGACCGTACAGA
GGGGGGAGGGGTGTCGTGGTGGAGTGCCCTAGTCGCGCCCATGCGCACGGTGCCCGCCGAGAGCCCCTGGCCGCGGGTGCCGCCGTGGAGCGCTCTGGCC
||||||||||||||||||||||||||||||||||||||||||||||||||||||||||||||||||||||||||||||||||||||||||||||||||||
GGGGGGAGGGGTGTCGTGGTGGAGTGCCCTAGTCGCGCCCATGCGCACGGTGCCCGCCGAGAGCCCCTGGCCGCGGGTGCCGCCGTGGAGCGCTCTGGCC
ACGCCCGTGCTAGAGGCTCACGTAGGGAGGCCTTGGCCGAGCGCGCCTTCGTGAAGCGCCGTGGCCATGCCCCATGCTCGCCGCGCCCGACGGGAGGCCC
||||||||||||||||||||||||||||||||||||||||||||||||||||||||||||||||||||||||||||||||||||||||||||||||||||
ACGCCCGTGCTAGAGGCTCACGTAGGGAGGCCTTGGCCGAGCGCGCCTTCGTGAAGCGCCGTGGCCATGCCCCATGCTCGCCGCGCCCGACGGGAGGCCC
TGGCCACCCTCGTGCTCTGCTCGCCCGCCGGGAGGGTCTGGTCGCGCCCATGCTCAAGTAAGCAGCAAGTGGCGGCGGAAGCAAAGCACACACGGGGCGA
||||||||||||||||||||||||||||||||||||||||||||||||||||||||||||||||||||||||||||||||||||||||||||||||||||
TGGCCACCCTCGTGCTCTGCTCGCCCGCCGGGAGGGTCTGGTCGCGCCCATGCTCAAGTAAGCAGCAAGTGGCGGCGGAAGCAAAGCACACACGGGGCGA
GGCCTGTTGTAGTCGTGCTCCTAGTCGACTCGGCACTGGCGTCAGGCAGCGGTCTGGGCGGCGAGGCGGATCCTCGCCGGATCCGGGCTGGACAAGGGTT
||||||||||||||||||||||||||||||||||||||||||||||||||||||||||||||||||||||||||||||||||||||||||||||||||||
GGCCTGTTGTAGTCGTGCTCCTAGTCGACTCGGCACTGGCGTCAGGCAGCGGTCTGGGCGGCGAGGCGGATCCTCGCCGGATCCGGGCTGGACAAGGGTT
GAATCCGCGCGGGGCCAGTGGCTTGCTGCAGCGGATGGTAGGTGGTTGGTACGACGGGGAGGATCGCCGGGGTGGGGTGGCTCGCTGGGGTAAGGCTCTG
||||||||||||||||||||||||||||||||||||||||||||||||||||||||||||||||||||||||||||||||||||||||||||||||||||
GAATCCGCGCGGGGCCAGTGGCTTGCTGCAGCGGATGGTAGGTGGTTGGTACGACGGGGAGGATCGCCGGGGTGGGGTGGCTCGCTGGGGTAAGGCTCTG
GCGGCGGCGCTGGATATGCCCGACTATGTCGGGTGGGTGGGGAAGAAGGTGGCTTGGGGAGAGAGA-GGGGGGGTGAATAAGACAGAGGCTGACAGGTGG
|||||||||||||||||||||||||||||||||||||||||||||||||||||||||||||||||| |||||||||||||||||||||||||||||||||
GCGGCGGCGCTGGATATGCCCGACTATGTCGGGTGGGTGGGGAAGAAGGTGGCTTGGGGAGAGAGAGGGGGGGGTGAATAAGACAGAGGCTGACAGGTGG
GGCCTTTGGTCACCTCAGCAGATTATCCAGGTAGGCATGCCACATCGGCCGGACAGGTGGGCCCAACCAGTTAGATTCGCTGTCTTTGCGACCAAATTCA
||||||||||||||||||||||||||||||||||||||||||||||||||||||||||||||||||||||||||||||||||||||||||||||||||||
GGCCTTTGGTCACCTCAGCAGATTATCCAGGTAGGCATGCCACATCGGCCGGACAGGTGGGCCCAACCAGTTAGATTCGCTGTCTTTGCGACCAAATTCA
AGCATCAGTGCTCCGCGTTACAGATTAGGCGCAATTTTGGTGGTTTTTTGTGCCCTGGCTTGAATGTGGTATCAAGTTATTACCCTAGCCTCAAGTGTGG
||||||||||||||||||||||||||||||||||||||||||||||||||||||||||||||||||||||||||||||||||||||||||||||||||||
AGCATCAGTGCTCCGCGTTACAGATTAGGCGCAATTTTGGTGGTTTTTTGTGCCCTGGCTTGAATGTGGTATCAAGTTATTACCCTAGCCTCAAGTGTGG
TGGTTTTGGGTAAATAACTCGTTCATATGAACCCCTTGCGACTTCGTCCCCTCGTGGGTTTTGAAAAATTCTTACATAAGCCATTGAACTACTAAGTGTT
||||||||||||||||||||||||||||||||||||||||||||||||||||||||||||||||||||||||||||||||||||||||||||||||||||
TGGTTTTGGGTAAATAACTCGTTCATATGAACCCCTTGCGACTTCGTCCCCTCGTGGGTTTTGAAAAATTCTTACATAAGCCATTGAACTACTAAGTGTT
CATTTAAGGACATATTCAAAAGTGTATGAAGAAAACAATCATGCGTATTGTACCCCAAAAAAAAGAAATTTTGAGTTCTGCAACAAAGAGATGTTTTTGT
||||||||||||||||||||||||||||||||||||||||||||||||||||||||||||||||||||||||||||||||||||||||||||||||||||
CATTTAAGGACATATTCAAAAGTGTATGAAGAAAACAATCATGCGTATTGTACCCCAAAAAAAAGAAATTTTGAGTTCTGCAACAAAGAGATGTTTTTGT
ATTTTCTCTTTATACTATATAATAGACATATTACCATATTTTTGCTCAAGTTTCTTTGTGTATGTACCAAATTAGAATTTGGTACTACCATGATTTTTTT
||||||||||||||||||||||||||||||||||||||||||||||||||||||||||||||||||||||||||||||||||||||||||||||||||||
ATTTTCTCTTTATACTATATAATAGACATATTACCATATTTTTGCTCAAGTTTCTTTGTGTATGTACCAAATTAGAATTTGGTACTACCATGATTTTTTT
AGAAACTTTTAATGTGTTCAAGTGTACACAGTTGGAATGAGAACCCAAACACGCTAAGTACACGTACATGTCCAGTAACCACTACGTCGCGCGTTAGGTT
||||||||||||||||||||||||||||||||||||||||||||||||||||||||||||||||||||||||||||||||||||||||||||||||||||
AGAAACTTTTAATGTGTTCAAGTGTACACAGTTGGAATGAGAACCCAAACACGCTAAGTACACGTACATGTCCAGTAACCACTACGTCGCGCGTTAGGTT
CAAAAATATGTTACTTATTGTTTGCAAACATATTGTGACCAATGGAACATCTGTTGAGCCAGTTCAGAAGCTTGTTCCAGTACACCCCAGCAATGCTAAT
||||||||||||||||||||||||||||||||||||||||||||||||||||||||||||||||||||||||||||||||||||||||||||||||||||
CAAAAATATGTTACTTATTGTTTGCAAACATATTGTGACCAATGGAACATCTGTTGAGCCAGTTCAGAAGCTTGTTCCAGTACACCCCAGCAATGCTAAT
AATCAGATCGCTTAAGCATGAACGTCAAAATATCAACAGCCTGAGAGCTGCCTACTCCAGAAAGCTCAGAAAATAATTGACGAATATAAAGAATGAATGT
||||||||||||||||||||||||||||||||||||||||||||||||||||||||||||||||||||||||||||||||||||||||||||||||||||
AATCAGATCGCTTAAGCATGAACGTCAAAATATCAACAGCCTGAGAGCTGCCTACTCCAGAAAGCTCAGAAAATAATTGACGAATATAAAGAATGAATGT
CATGTGTATACAGGTGACACATGACATGACACTAGTATCAAAACTTTTAAAAGGTGAATGAGACAATCCTCATCGCGCACCATATAACTAGCTAGTTCGT
||||||||||||||||||||||||||||||||||||||||||||||||||||||||||||||||||||||||||||||||||||||||||||||||||||
CATGTGTATACAGGTGACACATGACATGACACTAGTATCAAAACTTTTAAAAGGTGAATGAGACAATCCTCATCGCGCACCATATAACTAGCTAGTTCGT
GCATAGACACAATTTATTCAAGACGAGGTAGGTTAACAGAATAACAGAAAAGGGAGATAACTGATTGCTCTCGTCTAGTATATGTTGGCAATGGATTCTT
||||||||||||||||||||||||||||||||||||||||||||||||||||||||||||||||||||||||||||||||||||||||||||||||||||
GCATAGACACAATTTATTCAAGACGAGGTAGGTTAACAGAATAACAGAAAAGGGAGATAACTGATTGCTCTCGTCTAGTATATGTTGGCAATGGATTCTT
TGGTTATGGCTCGTGTTATACATGATGTGTTGGATCCATTTACATCAACTGTTCCACTCCGAATAGGCTACAACAATAGGCTGTTTCTGCCAGGTGCTGA
||||||||||||||||||||||||||||||||||||||||||||||||||||||||||||||||||||||||||||||||||||||||||||||||||||
TGGTTATGGCTCGTGTTATACATGATGTGTTGGATCCATTTACATCAACTGTTCCACTCCGAATAGGCTACAACAATAGGCTGTTTCTGCCAGGTGCTGA
TCTAAGACCATCTGCAGTTGCAAGCAAGCCGCGAGTTCATGTTGGTGGCAATTACATGAGAGTTCTCTACACCCTGGTATACACAAGTTCTTCAGTTATC
||||||||||||||||||||||||||||||||||||||||||||||||||||||||||||||||||||||||||||||||||||||||||||||||||||
TCTAAGACCATCTGCAGTTGCAAGCAAGCCGCGAGTTCATGTTGGTGGCAATTACATGAGAGTTCTCTACACCCTGGTATACACAAGTTCTTCAGTTATC
TAACTCTTCCGCATGGTTTGATAGTATTACCATGAAGAATTCTATAGAAACAAAAGGTCTCGCAGGTGATTAATTTACTCTTAACAAACTCGAACATAAT
||||||||||||||||||||||||||||||||||||||||||||||||||||||||||||||||||||||||||||||||||||||||||||||||||||
TAACTCTTCCGCATGGTTTGATAGTATTACCATGAAGAATTCTATAGAAACAAAAGGTCTCGCAGGTGATTAATTTACTCTTAACAAACTCGAACATAAT
TATATGTTAATTTATTTGAGTTTCTCTTTGACTTAAAAAGATGCTGGTGGATCCAGATGCCCCAAGCCCAAGTCACCCAACACTAAGGGAGTACTTGCAC
||||||||||||||||||||||||||||||||||||||||||||||||||||||||||||||||||||||||||||||||||||||||||||||||||||
TATATGTTAATTTATTTGAGTTTCTCTTTGACTTAAAAAGATGCTGGTGGATCCAGATGCCCCAAGCCCAAGTCACCCAACACTAAGGGAGTACTTGCAC
TGGTAAATTAGATGCAACATGTTCATATTCTCGTCACTATGACTACAATCCCTCCACCCATTTTTTTCCACCCTGTGTAGGATGGTGGTAGACATCCCTG
||||||||||||||||||||||||||||||||||||||||||||||||||||||||||||||||||||||||||||||||||||||||||||||||||||
TGGTAAATTAGATGCAACATGTTCATATTCTCGTCACTATGACTACAATCCCTCCACCCATTTTTTTCCACCCTGTGTAGGATGGTGGTAGACATCCCTG
GAACAACTGGTGCCAGCTTTGGTATGATGTCCTGCATAACAATTTATTCCATGTGATTTGCAGTTTTAGCTGAATTTGCGGGTGTGGAAATTGACCCATG
||||||||||||||||||||||||||||||||||||||||||||||||||||||||||||||||||||||||||||||||||||||||||||||||||||
GAACAACTGGTGCCAGCTTTGGTATGATGTCCTGCATAACAATTTATTCCATGTGATTTGCAGTTTTAGCTGAATTTGCGGGTGTGGAAATTGACCCATG
TTAATTTATTTGCCGCAAACATGGTGCCTACCATAGAATTGACAGATAGGGCTATGACTTAACTAAGGTAAAACTTAACTCAAAGTTAGCTCATCTCATC
||||||||||||||||||||||||||||||||||||||||||||||||||||||||||||||||||||||||||||||||||||||||||||||||||||
TTAATTTATTTGCCGCAAACATGGTGCCTACCATAGAATTGACAGATAGGGCTATGACTTAACTAAGGTAAAACTTAACTCAAAGTTAGCTCATCTCATC
GGGTTTTTTTTTTTTTGCAGTTGAAGTTAGCTGATCGTTAACAATTGTATATTTTAATACTAGACAAAGAATAACTATCAATGGCATAAAATGACATTCA
||||||||||||||||||||||||||||||||||||||||||||||||||||||||||||||||||||||||||||||||||||||||||||||||||||
GGGTTTTTTTTTTTTTGCAGTTGAAGTTAGCTGATCGTTAACAATTGTATATTTTAATACTAGACAAAGAATAACTATCAATGGCATAAAATGACATTCA
TATCTTTTAAAGTTCTTTAAGAAAGTCGATTGAACAACTTAATAAACTAAAGGACTCTTAGAAAAGTGCTATTCAGAAACACCTTTCCATGTGTATCATT
||||||||||||||||||||||||||||||||||||||||||||||||||||||||||||||||||||||||||||||||||||||||||||||||||||
TATCTTTTAAAGTTCTTTAAGAAAGTCGATTGAACAACTTAATAAACTAAAGGACTCTTAGAAAAGTGCTATTCAGAAACACCTTTCCATGTGTATCATT
TCTACTTAGTTTATTTTTCACATGGACAAAACCAAACTCACGCAATGAATTAGGGAAATTTTACGGTGCATTGCAATACATCAGAGCATGAGTATTATAT
||||||||||||||||||||||||||||||||||||||||||||||||||||||||||||||||||||||||||||||||||||||||||||||||||||
TCTACTTAGTTTATTTTTCACATGGACAAAACCAAACTCACGCAATGAATTAGGGAAATTTTACGGTGCATTGCAATACATCAGAGCATGAGTATTATAT
GGGTGATCCAACAGATGAGACTAACTGGGCCTTATTAAAGCCCAAGGGTATTTTCGAACTAAAAATTATCTAAGTTTAATTGGCCCAATTAAGTCTAGGG
||||||||||||||||||||||||||||||||||||||||||||||||||||||||||||||||||||||||||||||||||||||||||||||||||||
GGGTGATCCAACAGATGAGACTAACTGGGCCTTATTAAAGCCCAAGGGTATTTTCGAACTAAAAATTATCTAAGTTTAATTGGCCCAATTAAGTCTAGGG
GTATTATCAACCTAAAATTTTGAATTAACTTAATTGCATTAACATTGCATTTAATTATAACTAAACGTCATCGTGTGTGGTCATTTGAAAAGTTTGAGAG
||||||||||||||||||||||||||||||||||||||||||||||||||||||||||||||||||||||||||||||||||||||||||||||||||||
GTATTATCAACCTAAAATTTTGAATTAACTTAATTGCATTAACATTGCATTTAATTATAACTAAACGTCATCGTGTGTGGTCATTTGAAAAGTTTGAGAG
GAACGCCTGATCTGTTTGAATTTTTTTTCTGAAAATGCCCCTTGGCGTTCACATAATACACGTGACGTCAGTGTATTGCTTTGGATCTGGAACATCCAAT
||||||||||||||||||||||||||||||||||||||||||||||||||||||||||||||||||||||||||||||||||||||||||||||||||||
GAACGCCTGATCTGTTTGAATTTTTTTTCTGAAAATGCCCCTTGGCGTTCACATAATACACGTGACGTCAGTGTATTGCTTTGGATCTGGAACATCCAAT
CCGGTCCGACGGACGGTCCGTATCAACCGGATGCACTGTTGAATTACCAATTGTAAAATAGTGACAGGATGTCTCCACCCAGTTTAATTACTCTTTAAAT
||||||||||||||||||||||||||||||||||||||||||||||||||||||||||||||||||||||||||||||||||||||||||||||||||||
CCGGTCCGACGGACGGTCCGTATCAACCGGATGCACTGTTGAATTACCAATTGTAAAATAGTGACAGGATGTCTCCACCCAGTTTAATTACTCTTTAAAT
TCCTAACTAGTTAATAACTAATGCCATGGTGCTCTTGCAGGCCAAGAGCTTGTAGTTTATGAAAGACCGGAGCCAAGATCTGGCATCCACCGAATGGTAT
||||||||||||||||||||||||||||||||||||||||||||||||||||||||||||||||||||||||||||||||||||||||||||||||||||
TCCTAACTAGTTAATAACTAATGCCATGGTGCTCTTGCAGGCCAAGAGCTTGTAGTTTATGAAAGACCGGAGCCAAGATCTGGCATCCACCGAATGGTAT
TTGTGCTGTTCCAGCAACTAGGTAGGGGGACGGTTTTTGCACCGCACATGCGGCACAACTTCAGCTCCAGGAACTTCGCATGCCAGTACCACCTCAACAT
||||||||||||||||||||||||||||||||||||||||||||||||||||||||||||||||||||||||||||||||||||||||||||||||||||
TTGTGCTGTTCCAGCAACTAGGTAGGGGGACGGTTTTTGCACCGCACATGCGGCACAACTTCAGCTCCAGGAACTTCGCATGCCAGTACCACCTCAACAT
TGTGGCTGCCACATATTTCAACTGTCAAAGGGAAGGTGGATCGGGCGGAAGAAGGTTTAAACCAGAAAGTACTCAAAGGGGTAGATACTAGATAGTATAG
||||||||||||||||||||||||||||||||||||||||||||||||||||||||||||||||||||||||||||||||||||||||||||||||||||
TGTGGCTGCCACATATTTCAACTGTCAAAGGGAAGGTGGATCGGGCGGAAGAAGGTTTAAACCAGAAAGTACTCAAAGGGGTAGATACTAGATAGTATAG
AGCACAACCCATTATATGTTTGTACTGTAGTGTTGCATCACAATCAATGGGCGCTATATAGGATGCTCTGTATCTCTGTTGGATCGAAGGTGGTGCGACA
||||||||||||||||||||||||||||||||||||||||||||||||||||||||||||||||||||||||||||||||||||||||||||||||||||
AGCACAACCCATTATATGTTTGTACTGTAGTGTTGCATCACAATCAATGGGCGCTATATAGGATGCTCTGTATCTCTGTTGGATCGAAGGTGGTGCGACA
GTAGCGGGAGGTAGGCGGTAAGGGACGTCTTTGTCTTTCGTTGTCTTCTCCAGAGGTATCCGGCTATTGAGGCGCCGGTAGACGGATAAGGGCGGATGGT
||||||||||||||||||||||||||||||||||||||||||||||||||||||||||||||||||||||||||||||||||||||||||||||||||||
GTAGCGGGAGGTAGGCGGTAAGGGACGTCTTTGTCTTTCGTTGTCTTCTCCAGAGGTATCCGGCTATTGAGGCGCCGGTAGACGGATAAGGGCGGATGGT
ACAGACGGCTTCAGTGGCAAGTTTATGCACTCCGGTGGAAACACAAGATCTCTGGTTGGCCTATGTCGACCCGCGCCTGCATAGTGTCTTTGTTGTAGGT
||||||||||||||||||||||||||||||||||||||||||||||||||||||||||||||||||||||||||||||||||||||||||||||||||||
ACAGACGGCTTCAGTGGCAAGTTTATGCACTCCGGTGGAAACACAAGATCTCTGGTTGGCCTATGTCGACCCGCGCCTGCATAGTGTCTTTGTTGTAGGT
GGTGGACTGAAGCTTGACTTTGGGGATGAGAATTCAGAGTTCAACATTGTGGTGGACTTCCATCATCGGCGCACATGCAGCGTTTTCTTCTTGAAGGTGT
||||||||||||||||||||||||||||||||||||||||||||||||||||||||||||||||||||||||||||||||||||||||||||||||||||
GGTGGACTGAAGCTTGACTTTGGGGATGAGAATTCAGAGTTCAACATTGTGGTGGACTTCCATCATCGGCGCACATGCAGCGTTTTCTTCTTGAAGGTGT
GGCCTAGGAGTTGTGTATTTTTTGTTTCTGATGTGTCTTATAACATAGGGTTAGTGACTATTTGGGGAGTTACTGTCGTGAGGTATACGACCTCAGGATT
||||||||||||||||||||||||||||||||||||||||||||||||||||||||||||||||||||||||||||||||||||||||||||||||||||
GGCCTAGGAGTTGTGTATTTTTTGTTTCTGATGTGTCTTATAACATAGGGTTAGTGACTATTTGGGGAGTTACTGTCGTGAGGTATACGACCTCAGGATT
TGTTTTTCGCCTTATTTCCGGGTCGAGATTGTTCGCCTGCTGGATTATGCATTATCAATAATATGTGGTTGCATGCATGGTTCTGATGCAGAGGCCGGGG
||||||||||||||||||||||||||||||||||||||||||||||||||||||||||||||||||||||||||||||||||||||||||||||||||||
TGTTTTTCGCCTTATTTCCGGGTCGAGATTGTTCGCCTGCTGGATTATGCATTATCAATAATATGTGGTTGCATGCATGGTTCTGATGCAGAGGCCGGGG
ATTATCCTCCTTTTCCAAAAAAAAATCATCACTGATATCAATCCAACAGGCGGCCATACAAGGATAATACTATGCACTAGAAAAAATAGCATGCTATACC
||||||||||||||||||||||||||||||||||||||||||||||||||||||| ||||||||||||||||||||||||||||||||||||||||||||
ATTATCCTCCTTTTCCAAAAAAAAATCATCACTGATATCAATCCAACAGGCGGCCGTACAAGGATAATACTATGCACTAGAAAAAATAGCATGCTATACC
GTCGCTAATAACATGTTATAGTTTGTAGAGAATTGTCTCGTTAAATTTATCGGTGTACAATGTTTAACTAATAGTACGCTATAGCACGCTAATAACATAT
||||||||||||||||||||||||||||||||||||||||||||||||||||||||||||||||||||||||||||||||||||||||||||||||||||
GTCGCTAATAACATGTTATAGTTTGTAGAGAATTGTCTCGTTAAATTTATCGGTGTACAATGTTTAACTAATAGTACGCTATAGCACGCTAATAACATAT
TTTCAAGGGCGGCGCTATTTTGTATAGCGCATTATTTTTTTCCTTAGTTACTATGACGGCTTACCGCAGCGACAGCATTGCTTAACAGGTAAGCCATTCC
||||||||||||||||||||||||||||||||||||||||||||||||||||||||||||||||||||||||||||||||||||||||||||||||||||
TTTCAAGGGCGGCGCTATTTTGTATAGCGCATTATTTTTTTCCTTAGTTACTATGACGGCTTACCGCAGCGACAGCATTGCTTAACAGGTAAGCCATTCC
TCTTATTTCCTGCGGTGATTTTCGTTAATAAAACAAACTTATCTACAAAGGTAGGCCTTTCTCTTTTATTGAAGCCCTGACAGTAGAACATTGTTCTTCT
||||||||||||||||||||||||||||||||||||||||||||||||||||||||||||||||||||||||||||||||||||||||||||||||||||
TCTTATTTCCTGCGGTGATTTTCGTTAATAAAACAAACTTATCTACAAAGGTAGGCCTTTCTCTTTTATTGAAGCCCTGACAGTAGAACATTGTTCTTCT
GTGATTTTCATTAATAAAACAAAGTCATGTGCAAAGCTATGCCTTTCCTCACATAGCTTAACCAGTGAATTTGACTTTGGCTCCCTTCTGCCTTGCTAGC
||||||||||||||||||||||||||||||||||||||||||||||||||||||||||||||||||||||||||||||||||||||||||||||||||||
GTGATTTTCATTAATAAAACAAAGTCATGTGCAAAGCTATGCCTTTCCTCACATAGCTTAACCAGTGAATTTGACTTTGGCTCCCTTCTGCCTTGCTAGC
TAAGGTTATACATACATCCTTGATATTTTCCGACAAACTATCAGCTGCTACAGCACTTTCTTGTCCTGCCGACAAGCATGCAGCATCCAGCTGCCTAGCT
||||||||||||||||||||||||||||||||||||||||||||||||||||||||||||||||||||||||||||||||||||||||||||||||||||
TAAGGTTATACATACATCCTTGATATTTTCCGACAAACTATCAGCTGCTACAGCACTTTCTTGTCCTGCCGACAAGCATGCAGCATCCAGCTGCCTAGCT
AACCTCTGTTCAAACTCAACTTGAACTTCTTCTCTCGTTCCTTGGCTCTTACTTCATTGCATTAACTAGTAATACCGGCCTAGAGGTTTCAACAGTGAAA
||||||||||||||||||||||||||||||||||||||||||||||||||||||||||||||||||||||||||||||||||||||||||||||||||||
AACCTCTGTTCAAACTCAACTTGAACTTCTTCTCTCGTTCCTTGGCTCTTACTTCATTGCATTAACTAGTAATACCGGCCTAGAGGTTTCAACAGTGAAA
AAAGCATTACGAGCAATGTCTCGCTAGATAAATTAATGTATAGTATTTCATTAATAGCATGCTAATGGCGTATTCTAACGAACACCCTATTTTGTCAACT
||||||||||||||||||||||||||||||||||||||||||||||||||||||||||||||||||||||||||||||||||||||||||||||||||||
AAAGCATTACGAGCAATGTCTCGCTAGATAAATTAATGTATAGTATTTCATTAATAGCATGCTAATGGCGTATTCTAACGAACACCCTATTTTGTCAACT
TTTTTCATTGGGTTTCATGCAGCGTGTAGCTCTGTACAGCCAGCATGCATGCATTAGTCAAAACCAATTCAATCTGACTTCTTCTTCCTTGCATGCATCT
||||||||||||||||||||||||||||||||||||||||||||||||||||||||||||||||||||||||||||||||||||||||||||||||||||
TTTTTCATTGGGTTTCATGCAGCGTGTAGCTCTGTACAGCCAGCATGCATGCATTAGTCAAAACCAATTCAATCTGACTTCTTCTTCCTTGCATGCATCT
CTCAATTTCATTGTTTAACCATAATGGCAGGATTAATTCTAACATGGTCTAGTCTTCCAGTAATCATAAAGCTCAGTATCTAATCGGCAAATATCAAGGC
||||||||||||||||||||||||||||||||||||||||||||||||||||||||||||||||||||||||||||||||||||||||||||||||||||
CTCAATTTCATTGTTTAACCATAATGGCAGGATTAATTCTAACATGGTCTAGTCTTCCAGTAATCATAAAGCTCAGTATCTAATCGGCAAATATCAAGGC
CGGCCGTCATGTGTACAAAGGTGACGCATGACATGGCACTGGTATCATGGAGTCGCTTTCGTCCTGGAGATATTCATTCTCATCACTTGGGATGGGTCAT
||||||||||||||||||||||||||||||||||||||||||||||||||||||||||||||||||||||||||||||||||||||||||||||||||||
CGGCCGTCATGTGTACAAAGGTGACGCATGACATGGCACTGGTATCATGGAGTCGCTTTCGTCCTGGAGATATTCATTCTCATCACTTGGGATGGGTCAT
AACTTGAATGACTTGATAGTTCGAGTGAGAACCCAAACATGTTACCTACACCCCATCCCAGGAATAATGGCTTGAGCATCGACGTACGTCGAAATATCAA
||||||||||||||||||||||||||||||||||||||||||||||||||||||||||||||||||||||||||||||||||||||||||||||||||||
AACTTGAATGACTTGATAGTTCGAGTGAGAACCCAAACATGTTACCTACACCCCATCCCAGGAATAATGGCTTGAGCATCGACGTACGTCGAAATATCAA
CAGCCTGGGTGCCTCATCGCAAGCCATACAAGTAGTCCATGCATAGAAACAACAATTCTTCAGCTGGTCCGTGGAGAGCTAGGTCAAGAGAAAAACAGAA
||||||||||||||||||||||||||||||||||||||||||||||||||||||||||||||||||||||||||||||||||||||||||||||||||||
CAGCCTGGGTGCCTCATCGCAAGCCATACAAGTAGTCCATGCATAGAAACAACAATTCTTCAGCTGGTCCGTGGAGAGCTAGGTCAAGAGAAAAACAGAA
ATGTCGGCGGTGGATCCCTTGGTTGTGGCTCGTGTTATACATGATGTGTTGGATCCGTTTACATCAACTGTCCTACTCAGCATAGGCTACAACAACAGGC
||||||||||||||||||||||||||||||||||||||||||||||||||||||||||||||||||||||||||||||||||||||||||||||||||||
ATGTCGGCGGTGGATCCCTTGGTTGTGGCTCGTGTTATACATGATGTGTTGGATCCGTTTACATCAACTGTCCTACTCAGCATAGGCTACAACAACAGGC
TACTTCTGCGAGGTGCTGAGCTGAGACCATCTGCAGTCGTAAGCAAGCCGCGAGTCGATGTCGGTGGCAATGACATGAGAGTTCTCTACACCCTGGTAAA
||||||||||||||||||||||||||||||||||||||||||||||||||||||||||||||||||||||||||||||||||||||||||||||||||||
TACTTCTGCGAGGTGCTGAGCTGAGACCATCTGCAGTCGTAAGCAAGCCGCGAGTCGATGTCGGTGGCAATGACATGAGAGTTCTCTACACCCTGGTAAA
CTTCTAACTGGACTGCAGTGGGCAGTATACGCGACTTCTTCAGTTATCTAACTCTTCCACAAGGTTTACCTACTACTATTAGTGTGAAGAATTTCTTAGA
||||||||||||||||||||||||||||||||||||||||||||||||||||||||||||||||||||||||||||||||||||||||||||||||||||
CTTCTAACTGGACTGCAGTGGGCAGTATACGCGACTTCTTCAGTTATCTAACTCTTCCACAAGGTTTACCTACTACTATTAGTGTGAAGAATTTCTTAGA
AACGAAAGGTCTCGCAGGTGATTAATTTACTCCTAGCATACTCAAACATCTTAATTATATGTTAACTTGTTTGAGTTTTTATTTCACTTCAAAAGATGCT
||||||||||||||||||||||||||||||||||||||||||||||||||||||||||||||||||||||||||||||||||||||||||||||||||||
AACGAAAGGTCTCGCAGGTGATTAATTTACTCCTAGCATACTCAAACATCTTAATTATATGTTAACTTGTTTGAGTTTTTATTTCACTTCAAAAGATGCT
GGTGGATCCAGACGCCCCAAGCCCAAGTCACCCAACACTGAGGGAGTACTTGCACTGGTAAATCAATTAATTAAATGCAACATGTTCATATTCTCCAGCC
||||||||||||||||||||||||||||||||||||||||||||||||||||||||||||||||||||||||||||||||||||||||||||||||||||
GGTGGATCCAGACGCCCCAAGCCCAAGTCACCCAACACTGAGGGAGTACTTGCACTGGTAAATCAATTAATTAAATGCAACATGTTCATATTCTCCAGCC
ATTTTGTTTCGGCTCCGACAATGTTGAAATACGTTTCTTGTTTAGTTTGATCTCATTATGATATACCTCCACTCGTCCTGTGTAGGATGGTGGCAGACAT
||||||||||||||||||||||||||||||||||||||||||||||||||||||||||||||||||||||||||||||||||||||||||||||||||||
ATTTTGTTTCGGCTCCGACAATGTTGAAATACGTTTCTTGTTTAGTTTGATCTCATTATGATATACCTCCACTCGTCCTGTGTAGGATGGTGGCAGACAT
CCCTGGAACAACTGGTGTCAGCTTTGGTATGATGTTCTGCATAACAATTTGTGCCATTTGATTTTGCGGGTTTAGCTCAATTTGTGGATGTGGAAATTAT
||||||||||||||||||||||||||||||||||||||||||||||||||||||||||||||||||||||||||||||||||||||||||||||||||||
CCCTGGAACAACTGGTGTCAGCTTTGGTATGATGTTCTGCATAACAATTTGTGCCATTTGATTTTGCGGGTTTAGCTCAATTTGTGGATGTGGAAATTAT
CACTTGTTAAGTTCTTTGCCTCATATTCCCAAAAAAAAAAGGAAGAAGAAAAACTTTGCCTCAAACCTGTTGTCTACCCAGTTGAATTACTCTTTAAATT
||||||||||||||||||||||||||||||||||||||||||||||||||||||||||||||||||||||||||||||||||||||||||||||||||||
CACTTGTTAAGTTCTTTGCCTCATATTCCCAAAAAAAAAAGGAAGAAGAAAAACTTTGCCTCAAACCTGTTGTCTACCCAGTTGAATTACTCTTTAAATT
CTTATTAAGTGGTTGATGACTAACTAATGCCATGGTGCTCTTGCAGGCCAAGAGCTTGTGGTTTATGAAAGACCGGAGCCAAGATCCGGCATCCACCGGA
||||||||||||||||||||||||||||||||||||||||||||||||||||||||||||||||||||||||||||||||||||||||||||||||||||
CTTATTAAGTGGTTGATGACTAACTAATGCCATGGTGCTCTTGCAGGCCAAGAGCTTGTGGTTTATGAAAGACCGGAGCCAAGATCCGGCATCCACCGGA
TGGTATTTGTGCTGTTCCAGCAACTAGGTAGGGGGACGGTTTTCGCGCCGGACATGCGGCACAACTTCAGCTGCAGGAGCCTCGCACGCCAGTACCACCT
||||||||||||||||||||||||||||||||||||||||||||||||||||||||||||||||||||||||||||||||||||||||||||||||||||
TGGTATTTGTGCTGTTCCAGCAACTAGGTAGGGGGACGGTTTTCGCGCCGGACATGCGGCACAACTTCAGCTGCAGGAGCCTCGCACGCCAGTACCACCT
CAGCATTGTGGCCGCGACATATTTCAACTGTCAAATCGAAGGTGGATGGGGCGGGAGAAGGTTTAGGCCAGAAAGTTCTCAAGGGGAGTAGATGCTAGAT
||||||||||||||||||||||||||||||||||||||||||||||||||||||||||||||||||||||||||||||||||||||||||||||||||||
CAGCATTGTGGCCGCGACATATTTCAACTGTCAAATCGAAGGTGGATGGGGCGGGAGAAGGTTTAGGCCAGAAAGTTCTCAAGGGGAGTAGATGCTAGAT
AATACAGAGTATAGGCCATTGTATGCCTGAACTGAACTGTAGTGTTGCATCACAATATACGATGCTCGGTATCACCCATATATGCAGGGTATGTATCTTG
||||||||||||||||||||||||||||||||||||||||||||||||||||||||||||||||||||||||||||||||||||||||||||||||||||
AATACAGAGTATAGGCCATTGTATGCCTGAACTGAACTGTAGTGTTGCATCACAATATACGATGCTCGGTATCACCCATATATGCAGGGTATGTATCTTG
AGTACATTATATTCTACAAAAAAACTACTCCCTCCGTCCCATAATATAAGAACGTTTTTGACACTCAAAAACGTTCTTATATTATGGGACGGAGGGAGTA
||||||||||||||||||||||||||||||||||||||||||||||||||||||||||||||||||||||||||||||||||||||||||||||||||||
AGTACATTATATTCTACAAAAAAACTACTCCCTCCGTCCCATAATATAAGAACGTTTTTGACACTCAAAAACGTTCTTATATTATGGGACGGAGGGAGTA
TGTATCTTCAGCTAGGACGAACATGATGGAATATACCTCTGTTATTATTTAGTCATGCAAAGTATTAGCAATCTGAATGCATTGCTGGTTCTAAGCACCT
||||||||||||||||||||||||||||||||||||||||||||||||||||||||||||||||||||||||||||||||||||||||||||||||||||
TGTATCTTCAGCTAGGACGAACATGATGGAATATACCTCTGTTATTATTTAGTCATGCAAAGTATTAGCAATCTGAATGCATTGCTGGTTCTAAGCACCT
GCTCTGCATTTCTAACAGGTTGTGCTGAATAAAATCAACACGGTCTAAATCGTCAAAAATAATTTCGACAGCAAATTAAACCCAAGATTTGTTTAGTCGA
||||||||||||||||||||||||||||||||||||||||||||||||||||||||||||||||||||||||||||||||||||||||||||||||||||
GCTCTGCATTTCTAACAGGTTGTGCTGAATAAAATCAACACGGTCTAAATCGTCAAAAATAATTTCGACAGCAAATTAAACCCAAGATTTGTTTAGTCGA
AAGGAAGGAATTATTGACGGATCGACCAACTTTGCATTGTAAATCCCAGAAGTGGCAAGGTAATTAAACCCAAGGTAGGTCGAATATTTACATCATGCCT
||||||||||||||||||||||||||||||||||||||||||||||||||||||||||||||||||||||||||||||||||||||||||||||||||||
AAGGAAGGAATTATTGACGGATCGACCAACTTTGCATTGTAAATCCCAGAAGTGGCAAGGTAATTAAACCCAAGGTAGGTCGAATATTTACATCATGCCT
AGCCTATTCAAAGCAAAAGGGGCTACTGGAGGTGCAAACCAAAAACTTAGCAGTATATATTACAGAGCAGCAAAATGAAGCATCATACGGATGAAACTGA
||||||||||||||||||||||||||||||||||||||||||||||||||||||||||||||||||||||||||||||||||||||||||||||||||||
AGCCTATTCAAAGCAAAAGGGGCTACTGGAGGTGCAAACCAAAAACTTAGCAGTATATATTACAGAGCAGCAAAATGAAGCATCATACGGATGAAACTGA
TTACTACTTTACTTCACAACGAGCTCCCAGGACTCCAAGCTTTTGTACATGGCATCCGTTATATACAGTAAAATGCAAGTTCAGAGATCTCAAACACTCA
||||||||||||||||||||||||||||||||||||||||||||||||||||||||||||||||||||||||||||||||||||||||||||||||||||
TTACTACTTTACTTCACAACGAGCTCCCAGGACTCCAAGCTTTTGTACATGGCATCCGTTATATACAGTAAAATGCAAGTTCAGAGATCTCAAACACTCA
ATGGTTGGTTGATCCATGACACCCAGAGTGAGCACACAAGTGACCGATTGCCTGCAAAATTCGGATAACCTGGAACAACCAGAGCAAATAACAGGGGAAT
||||||||||||||||||||||||||||||||||||||||||||||||||||||||||||||||||||||||||||||||||||||||||||||||||||
ATGGTTGGTTGATCCATGACACCCAGAGTGAGCACACAAGTGACCGATTGCCTGCAAAATTCGGATAACCTGGAACAACCAGAGCAAATAACAGGGGAAT
AACCGAAGAAATTCTCTGGCAATCATGAATTCTCTCCGGGGGCGCCAATCACAAATGCAACTCCTGTCCTGATCAAACACACCTCAGCACCCACCGGCCA
||||||||||||||||||||||||||||||||||||||||||||||||||||||||||||||||||||||||||||||||||||||||||||||||||||
AACCGAAGAAATTCTCTGGCAATCATGAATTCTCTCCGGGGGCGCCAATCACAAATGCAACTCCTGTCCTGATCAAACACACCTCAGCACCCACCGGCCA
CTCGTCGTCTCGCACTTAGTACCAAAGTAGGAAATCGCGTGGAGGAGCACAGGGGGTGGGATCCAGACGGAGGCAGATGATGTACCTGTACAACGGTTGA
||||||||||||||||||||||||||||||||||||||||||||||||||||||||||||||||||||||||||||||||||||||||||||||||||||
CTCGTCGTCTCGCACTTAGTACCAAAGTAGGAAATCGCGTGGAGGAGCACAGGGGGTGGGATCCAGACGGAGGCAGATGATGTACCTGTACAACGGTTGA
CGCCGGTGGCTTGGGCGGTGCAGGGGGCGGCGCGGCGTGGCCGCTGAGAGGGTCGAACACCGGGCGGCCGTGTGCGGCACGGCGCGGCCGCTGAGAGGCG
||||||||||||||||||||||||||||||||||||||||||||||||||||||||||||||||||||||||||||||||||||||||||||||||||||
CGCCGGTGGCTTGGGCGGTGCAGGGGGCGGCGCGGCGTGGCCGCTGAGAGGGTCGAACACCGGGCGGCCGTGTGCGGCACGGCGCGGCCGCTGAGAGGCG
GGCACGGATGGGCTCCGGCGAGAGGATCAACACGGAGCGCACCTCCTCCGGCAACCGGTCCGCCGGGTGTCAACGGACCTCACCTCTGAATTCATTCTGA
||||||||||||||||||||||||||||||||||||||||||||||||||||||||||||||||||||||||||||||||||||||||||||||||||||
GGCACGGATGGGCTCCGGCGAGAGGATCAACACGGAGCGCACCTCCTCCGGCAACCGGTCCGCCGGGTGTCAACGGACCTCACCTCTGAATTCATTCTGA
AATATAACTGAATGCAGGCGCATTAACAGACTAAGTAAGTTCTGCACATATTCTGTTCACCAAGAGACACTTTGGCCGAGTGGTTAAGGCGTGTGCCTGC
||||||||||||||||||||||||||||||||||||||||||||||||||||||||||||||||||||||||||||||||||||||||||||||||||||
AATATAACTGAATGCAGGCGCATTAACAGACTAAGTAAGTTCTGCACATATTCTGTTCACCAAGAGACACTTTGGCCGAGTGGTTAAGGCGTGTGCCTGC
TAAGTACATGGGGTTTCCCCGCGAGAGTTCGAATCTCTCAGGTGTCGATTTTCTTTTTCTTTTTTACCTCTTTTTGCTTCGCTGTTCTTTTTTGAATTAT
||||||||||||||||||||||||||||||||||||||||||||||||||||||||||||||||||||||||||||||||||||||||||||||||||||
TAAGTACATGGGGTTTCCCCGCGAGAGTTCGAATCTCTCAGGTGTCGATTTTCTTTTTCTTTTTTACCTCTTTTTGCTTCGCTGTTCTTTTTTGAATTAT
TTATATTTTTTCGATGA
|||||||||||||||||
TTATATTTTTTCGATGA

***FT-A6 - TRIAE_CS42_6AS_TGACv1_485558_AA1547550***

ATATTGTTATATGACAACTTTCCTTGTGCATATTTGTATCTATATGTGTGACCATATATATTTGCGTATATATTGGTTGGCGAGAGGAAAGTTTTACGTA
||||||||||||||||||||||||||||||||||||||||||||||||||||||||||||||||||||||||||||||||||||||||||||||||||||
ATATTGTTATATGACAACTTTCCTTGTGCATATTTGTATCTATATGTGTGACCATATATATTTGCGTATATATTGGTTGGCGAGAGGAAAGTTTTACGTA
TTTTCAATAAATGTTGAGGAAAGTAAGCCGGTATGCAAATATTTGTGTAGCCTGTAAACAATACTCCATGACAGTTCAGAGCTGAGAAACAATGCCAAGC
||||||||||||||||||||||||||||||||||||||||||||||||||||||||||||||||||||||||||||||||||||||||||||||||||||
TTTTCAATAAATGTTGAGGAAAGTAAGCCGGTATGCAAATATTTGTGTAGCCTGTAAACAATACTCCATGACAGTTCAGAGCTGAGAAACAATGCCAAGC
TAGCGATCACCAAGCTTCGTCAAAAAGGGTCGGTCCTCCACGGACGCATCTATTCCGATCCCGAGATCATCTGAGCTCGGATGAACGGTAAAATCAAATT
||||||||||||||||||||||||||||||||||||||||||||||||||||||||||||||||||||||||||||||||||||||||||||||||||||
TAGCGATCACCAAGCTTCGTCAAAAAGGGTCGGTCCTCCACGGACGCATCTATTCCGATCCCGAGATCATCTGAGCTCGGATGAACGGTAAAATCAAATT
AAATAAAAAATAACAAAAAATCTGAAAAAATATTTGTGTCAATGTTTGGCACAAAAAAATTCACATTTTTTGAATTTTTATTTTATTTTACTGTGCACCC
||||||||||||||||||||||||||||||||||||||||||||||||||||||||||||||||||||||||||||||||||||||||||||||||||||
AAATAAAAAATAACAAAAAATCTGAAAAAATATTTGTGTCAATGTTTGGCACAAAAAAATTCACATTTTTTGAATTTTTATTTTATTTTACTGTGCACCC
GAGCTCAGAACAACAATTTGGATCCTCCACGTGTTTAATCATATCATACAAATCTCGCACAATATGTTGGCCCGTACACACATCAAATTGTAGATGTGTA
||||||||||||||||||||||||||||||||||||||||||||||||||||||||||||||||||||||||||||||||||||||||||||||||||||
GAGCTCAGAACAACAATTTGGATCCTCCACGTGTTTAATCATATCATACAAATCTCGCACAATATGTTGGCCCGTACACACATCAAATTGTAGATGTGTA
TATGCCCCTCAATAATTGTGCAAAGAGATGCTAGTTAGCTTTACTACATACGTACGTCATTCGAGCTATAAAGACATGCCTGGAATCAGCATGCACGACC
||||||||||||||||||||||||||||||||||||||||||||||||||||||||||||||||||||||||||||||||||||||||||||||||||||
TATGCCCCTCAATAATTGTGCAAAGAGATGCTAGTTAGCTTTACTACATACGTACGTCATTCGAGCTATAAAGACATGCCTGGAATCAGCATGCACGACC
GGTCAAATCTAGTGGAGCGAAAATTACTATCAAGGGCATACAAGGAACGATGACAACGATTGGAGCTGCATGTGATATGCATGGCGGTTTCTCGATCTTC
||||||||||||||||||||||||||||||||||||||||||||||||||||||||||||||||||||||||||||||||||||||||||||||||||||
GGTCAAATCTAGTGGAGCGAAAATTACTATCAAGGGCATACAAGGAACGATGACAACGATTGGAGCTGCATGTGATATGCATGGCGGTTTCTCGATCTTC
TCATGACTAGCTACTATAAATATGACGCCTAAGTACCTCTAGCTCTGTAGTATCTTTATACCGAGCCATTACACTGAAGCTAGACATCAACACGCATACA
||||||||||||||||||||||||||||||||||||||||||||||||||||||||||||||||||||||||||||||||||||||||||||||||||||
TCATGACTAGCTACTATAAATATGACGCCTAAGTACCTCTAGCTCTGTAGTATCTTTATACCGAGCCATTACACTGAAGCTAGACATCAACACGCATACA
TCTGCGTATACTATACACATCATATATACACTAGAAATGTCGAACGACTCCTTGGTTACATCACGAATAGTAGGTGATGTGTTGGACCCCTTCCGTAGCA
||||||||||||||||||||||||||||||||||||||||||||||||||||||||||||||||||||||||||||||||||||||||||||||||||||
TCTGCGTATACTATACACATCATATATACACTAGAAATGTCGAACGACTCCTTGGTTACATCACGAATAGTAGGTGATGTGTTGGACCCCTTCCGTAGCA
CAGTTGATCTGACAGTGCTCTATGACGGTAGGTTCGTCATTAACGGCATGGAGTTCCGCTCACCGGCGGTATCGGGCAAGCCGAGCGTCGAGATCGGCGG
||||||||||||||||||||||||||||||||||||||||||||||||||||||||||||||||||||||||||||||||||||||||||||||||||||
CAGTTGATCTGACAGTGCTCTATGACGGTAGGTTCGTCATTAACGGCATGGAGTTCCGCTCACCGGCGGTATCGGGCAAGCCGAGCGTCGAGATCGGCGG
TGATGATATTAGCGTGACATACACCCTTGTAAGCCCATGATTTATTAGTTATTTATGTTTTCAGCACTGTGGTTTGTTCAAGTTAACCATGTAATATGTG
||||||||||||||||||||||||||||||||||||||||||||||||||||||||||||||||||||||||||||||||||||||||||||||||||||
TGATGATATTAGCGTGACATACACCCTTGTAAGCCCATGATTTATTAGTTATTTATGTTTTCAGCACTGTGGTTTGTTCAAGTTAACCATGTAATATGTG
AATGTGATACATGCAGGTCATGGTGGATCCTGATGCTCCTAACCCCAGCAATCCGACCTTGAGGGAATATCTTCACTGGTTAGAGACTAAGAGCAACCCT
||||||||||||||||||||||||||||||||||||||||||||||||||||||||||||||||||||||||||||||||||||||||||||||||||||
AATGTGATACATGCAGGTCATGGTGGATCCTGATGCTCCTAACCCCAGCAATCCGACCTTGAGGGAATATCTTCACTGGTTAGAGACTAAGAGCAACCCT
ACCTATAGCTTATTAGGTCTTGCATGCTGTCCTGCGAGCAGGAAATATACTCTTTTTTTTTCGGGAAATATACTCTATACATGCATCACATCTAACGGTG
||||||||||||||||||||||||||||||||||||||||||||||||||||||||||||||||||||||||||||||||||||||||||||||||||||
ACCTATAGCTTATTAGGTCTTGCATGCTGTCCTGCGAGCAGGAAATATACTCTTTTTTTTTCGGGAAATATACTCTATACATGCATCACATCTAACGGTG
GATATATGTATTGGTGCATGCATGATAAGCTATATGATGCTTTAGGTCGTTAGATAAAAAACAAAATCCAAGAAAAGATAATGATAATATATATTCTTTC
||||||||||||||||||||||||||||||||||||||||||||||||||||||||||||||||||||||||||||||||||||||||||||||||||||
GATATATGTATTGGTGCATGCATGATAAGCTATATGATGCTTTAGGTCGTTAGATAAAAAACAAAATCCAAGAAAAGATAATGATAATATATATTCTTTC
ACTCATGTATCATGCACGTACATAACAACAAATATGCTTATGGCAAGCGTGTCGACGGACATGACATACTATAAATATGTATGGATAATTTCAAGATAGG
||||||||||||||||||||||||||||||||||||||||||||||||||||||||||||||||||||||||||||||||||||||||||||||||||||
ACTCATGTATCATGCACGTACATAACAACAAATATGCTTATGGCAAGCGTGTCGACGGACATGACATACTATAAATATGTATGGATAATTTCAAGATAGG
TTCACTGTGAGTACATCCATTTTTATTGTGAGTACAGCTAGATCTATATCCAAGATCCCCACTAACTATCGACCGACTGATTCATGTCCATGCTCCATGC
||||||||||||||||||||||||||||||||||||||||||||||||||||||||||||||||||||||||||||||||||||||||||||||||||||
TTCACTGTGAGTACATCCATTTTTATTGTGAGTACAGCTAGATCTATATCCAAGATCCCCACTAACTATCGACCGACTGATTCATGTCCATGCTCCATGC
AGGATGGTAACCGATATCCCAGGATCAATGGATGACACCTACGGTGAGCACTCGTGAATGCAATAATAGTACATGACTTGGCATCTCTGAACAGTTTGGA
||||||||||||||||||||||||||||||||||||||||||||||||||||||||||||||||||||||||||||||||||||||||||||||||||||
AGGATGGTAACCGATATCCCAGGATCAATGGATGACACCTACGGTGAGCACTCGTGAATGCAATAATAGTACATGACTTGGCATCTCTGAACAGTTTGGA
TGTAAATTTTTCTTCATTGTTTGAAGGGCGGGAGGTGGTGTGCTACGAGAGTCCGACGCCGACGACGGGGATCCACCGCATGGTGCTGGTGCTGTTCCGG
||||||||||||||||||||||||||||||||||||||||||||||||||||||||||||||||||||||||||||||||||||||||||||||||||||
TGTAAATTTTTCTTCATTGTTTGAAGGGCGGGAGGTGGTGTGCTACGAGAGTCCGACGCCGACGACGGGGATCCACCGCATGGTGCTGGTGCTGTTCCGG
CAGCTCGGGCGGAACACGGTGTACGCGGCGTCCATGCGCCACAACTTCAACACCCGCAGCTTCGCCCGCCGCTACAACCTTGGCGCGCCCGTCGCCGCAA
||||||||||||||||||||||||||||||||||||||||||||||||||||||||||||||||||||||||||||||||||||||||||||||||||||
CAGCTCGGGCGGAACACGGTGTACGCGGCGTCCATGCGCCACAACTTCAACACCCGCAGCTTCGCCCGCCGCTACAACCTTGGCGCGCCCGTCGCCGCAA
AGTACTTCAACTGCCAGCGCCAGGCCGGCTCCGGCGGCCGGAAGTTCACCGGGCCCTATACCAGCCACCGCCAGCAAATCTAATCCCTAGCTAACAAAAT
||||||||||||||||||||||||||||||||||||||||||||||||||||||||||||||||||||||||||||||||||||||||||||||||||||
AGTACTTCAACTGCCAGCGCCAGGCCGGCTCCGGCGGCCGGAAGTTCACCGGGCCCTATACCAGCCACCGCCAGCAAATCTAATCCCTAGCTAACAAAAT
AGCTACCACACACGGGAGCACGTGGCGCACGCATGGGCATGGCAACGTCATGGATCACATGAGAATAAGACTGGCTACAATTACCGGTAGAAAAAGACTA
||||||||||||||||||||||||||||||||||||||||||||||||||||||||||||||||||||||||||||||||||||||||||||||||||||
AGCTACCACACACGGGAGCACGTGGCGCACGCATGGGCATGGCAACGTCATGGATCACATGAGAATAAGACTGGCTACAATTACCGGTAGAAAAAGACTA
GCTACAAATAAATGAATAAAAGTAGTTCCTTCCAAAAGACCCCGGCCGGATGAATAATACTAGTGTACGTAGCTTCTTCCCTTTTTGGGCGGAGAAAAAG
||||||||||||||||||||||||||||||||||||||||||||||||||||||||||||||||||||||||||||||||||||||||||||||||||||
GCTACAAATAAATGAATAAAAGTAGTTCCTTCCAAAAGACCCCGGCCGGATGAATAATACTAGTGTACGTAGCTTCTTCCCTTTTTGGGCGGAGAAAAAG
CACATAGCTTCACATAGATGTTGCATCATATATGTGCTCGATATAAATCTCTTTTCTGAAATAATATCTAGCTCCGCCTTCTATATACTCTCAAGACTCG
||||||||||||||||||||||||||||||||||||||||||||||||||||||||||||||||||||||||||||||||||||||||||||||||||||
CACATAGCTTCACATAGATGTTGCATCATATATGTGCTCGATATAAATCTCTTTTCTGAAATAATATCTAGCTCCGCCTTCTATATACTCTCAAGACTCG
ACAAGGTGATTCTGACAAGTTGTTATTCCTGGTCTCCTGGATGATCTCAAGCATTGCTGCTCCACCTGCCCTGCCTTTGTTTGGTCGGCGGTGATTTTAA
||||||||||||||||||||||||||||||||||||||||||||||||||||||||||||||||||||||||||||||||||||||||||||||||||||
ACAAGGTGATTCTGACAAGTTGTTATTCCTGGTCTCCTGGATGATCTCAAGCATTGCTGCTCCACCTGCCCTGCCTTTGTTTGGTCGGCGGTGATTTTAA
TTTGATTGCTAGCGCTGCTGACAAAAGCAACATCAATCTGAACCGGCGCACCATGGCTGCGTTCCGGCGTTTCATAAACGAGATGGAGCTCAAGGACATG
||||||||||||||||||||||||||||||||||||||||||||||||||||||||||||||||||||||||||||||||||||||||||||||||||||
TTTGATTGCTAGCGCTGCTGACAAAAGCAACATCAATCTGAACCGGCGCACCATGGCTGCGTTCCGGCGTTTCATAAACGAGATGGAGCTCAAGGACATG
TATCTCCATGGTCGGAGATGCATTTGGTTCAATAAGCAAGGAAGGTCGATACGCTGTTGTTTTAAGCGATAAGAGCAACTCCAACGGGCCGATCGAAACG
||||||||||||||||||||||||||||||||||||||||||||||||||||||||||||||||||||||||||||||||||||||||||||||||||||
TATCTCCATGGTCGGAGATGCATTTGGTTCAATAAGCAAGGAAGGTCGATACGCTGTTGTTTTAAGCGATAAGAGCAACTCCAACGGGCCGATCGAAACG
GTCGGCGATTTTGTCCATTTGAGTCGACCAGGCGGACACGGATGTCCGCTTTCATATTTGGGTCGGCGTGTGCGCCCAAGACT
|||||||||||||||||||||||||||||||||||||||||||||||||||||||||||||||||||||||||||||||||||
GTCGGCGATTTTGTCCATTTGAGTCGACCAGGCGGACACGGATGTCCGCTTTCATATTTGGGTCGGCGTGTGCGCCCAAGACT

***FT-B6 - TRIAE_CS42_6BS_TGACv1_513941_AA1652570***

ATGCGAAGCTAGCGATCACCAAGCTTCGTCAAAAGTGGTCGGTCCTCCACGGACGCATCTGTTCCGATCCCGAGCTCATCTGAGCTCGGATGAACGGTAC
||||||||||||||||||||||||||||||||||||||||||||||||||||||||||||||||||||||||||||||||||||||||||||||||||||
ATGCGAAGCTAGCGATCACCAAGCTTCGTCAAAAGTGGTCGGTCCTCCACGGACGCATCTGTTCCGATCCCGAGCTCATCTGAGCTCGGATGAACGGTAC
AATCAAATAAAATGAAAAAAATCAAAAAAAATCTGATTTTTTTGTGCCAAACATTGACAAATGTTTGATTGCTTGCAAAGTTTCATCACCTGGCACAAGA
||||||||||||||||||||||||||||||||||||||||||||||||||||||||||||||||||||||||||||||||||||||||||||||||||||
AATCAAATAAAATGAAAAAAATCAAAAAAAATCTGATTTTTTTGTGCCAAACATTGACAAATGTTTGATTGCTTGCAAAGTTTCATCACCTGGCACAAGA
AAATCATATTTTTTTTTATTTTATTTTTACTGTCCACCTGAGCTCACATGAGTTGGGAATCATAACAACACTTTTGGTCCTCCACGTGTTTCGTCATATC
||||||||||| |||| ||||||||||||||||||||||||||||||||||||| |||||||||||||||||||||||||||||||||||||||||||||
AAATCATATTTATTTT-ATTTTATTTTTACTGTCCACCTGAGCTCACATGAGTTCGGAATCATAACAACACTTTTGGTCCTCCACGTGTTTCGTCATATC
ATACAAATGTCGCACAATATGTTGGCCCGTACACACATCAAATTGTAGATGTGTATATGCCCCCCAATAATTGTGCAAAGAGATGCTAGTTAGCTTTACT
||||||||||||||||||||||||||||||||||||||||||||||||||||||||||||||||||||||||||||||||||||||||||||||||||||
ATACAAATGTCGCACAATATGTTGGCCCGTACACACATCAAATTGTAGATGTGTATATGCCCCCCAATAATTGTGCAAAGAGATGCTAGTTAGCTTTACT
ACATACGTCATTCTAGCTAGCTAGAAAGACATGCCTGGAATCAGCATGCACGACCGGTCAAATTTAGTGGAGCGAAAATTACTATCAAGGGCATACAAGG
||||||||||||||||||||||||||||||||||||||||||||||||||||||||||||||||||||||||||||||||||||||||||||||||||||
ACATACGTCATTCTAGCTAGCTAGAAAGACATGCCTGGAATCAGCATGCACGACCGGTCAAATTTAGTGGAGCGAAAATTACTATCAAGGGCATACAAGG
AACGATGACAAGGATTGGAGCTGCATGTGATATGCATGGCGGTTTCTCGATCTTCTCATGGGTAGCTACTATAAATATGACGCCTAAGTACCTCTAGCCC
||||||||||||||||||||||||||||||||||||||||||||||||||||||||||||||||||||||||||||||||||||||||||||||||||||
AACGATGACAAGGATTGGAGCTGCATGTGATATGCATGGCGGTTTCTCGATCTTCTCATGGGTAGCTACTATAAATATGACGCCTAAGTACCTCTAGCCC
TGTAGTATCTTTATACCGAGCCATCACACTGAAGCTAGACATCAACACACATACATCTGCGTATACTATACTACAACATCATATATACACTAGAAATGTC
||||||||||||||||||||||||||||||||||||||||||||||||||||||||||||||||||||||||||||||||||||||||||||||||||||
TGTAGTATCTTTATACCGAGCCATCACACTGAAGCTAGACATCAACACACATACATCTGCGTATACTATACTACAACATCATATATACACTAGAAATGTC
GAATGACTCCTTGGTTACATCACGAGTAGTAGGAGATGTGTTGGACCCCTTCCGTAGCACAGTTGATCTGACAGTGCTCTATGACGGTAGGTTTGTCATT
||||||||||||||||||||||||||||||||||||||||||||||||||||||||||||||||||||||||||||||||||||||||||||||||||||
GAATGACTCCTTGGTTACATCACGAGTAGTAGGAGATGTGTTGGACCCCTTCCGTAGCACAGTTGATCTGACAGTGCTCTATGACGGTAGGTTTGTCATT
AACGGCATGGAGTTCCGCTCACCGGCGGTATCGGGCAAGCCGAGCGTCGAGATTGGCGGCGACGATATCAGCGTGACATACACCCTTGTAAGCCCATGAT
||||||||||||||||||||||||||||||||||||||||||||||||||||||||||||||||||||||||||||||||||||||||||||||||||||
AACGGCATGGAGTTCCGCTCACCGGCGGTATCGGGCAAGCCGAGCGTCGAGATTGGCGGCGACGATATCAGCGTGACATACACCCTTGTAAGCCCATGAT
TTATTAGTCATTTATGTTTACAGCACTGTGGTTTGTCCAAGTTAACTGTGTAATCTGTGAATGTGATACATGCAGGTCATGGTGGATCCTGATGCTCCTA
||||||||||||||||||||||||||||||||||||||||||||||||||||||||||||||||||||||||||||||||||||||||||||||||||||
TTATTAGTCATTTATGTTTACAGCACTGTGGTTTGTCCAAGTTAACTGTGTAATCTGTGAATGTGATACATGCAGGTCATGGTGGATCCTGATGCTCCTA
ACCCCAGCAATCCGACCTTGAGGGAATATCTTCACTGGTGAGAGGCTGAGTGCAACCCTAGCTAGCTTGCTCTTGCATGCCGTGGATTAGGAAATATAAT
||||||||||||||||||||||||||||||||||||||||||||||||||||||||||||||||||||||||||||||||||||||||||||||||||||
ACCCCAGCAATCCGACCTTGAGGGAATATCTTCACTGGTGAGAGGCTGAGTGCAACCCTAGCTAGCTTGCTCTTGCATGCCGTGGATTAGGAAATATAAT
CTATACATGCATCACATGTAACGGTGGATATATGTATTGGTGCATGCATGATCATAATAATAAGATACTCATGGATCATGCACGTACGTACATGACCACA
||||||||||||||||||||||||||||||||||||||||||||||||||||||||||||||||||||||||||||||||||||||||||||||||||||
CTATACATGCATCACATGTAACGGTGGATATATGTATTGGTGCATGCATGATCATAATAATAAGATACTCATGGATCATGCACGTACGTACATGACCACA
AATATGCTTGTGGCAGGCGTGTCAATGGACATGACATACTATAAATATATGTGCATTATTCAGGATAGCTAGGTTCATTGTGAGTACAGCTAGATATATA
||||||||||||||||||||||||||||||||||||||||||||||||||||||||||||||||||||||||||||||||||||||||||||| |||||
AATATGCTTGTGGCAGGCGTGTCAATGGACATGACATACTATAAATATATGTGCATTATTCAGGATAGCTAGGTTCATTGTGAGTACAGCTAG--ATATA
GATCCAATATATCCTAACTAACTATCGACCGACTGATTCATGTCCATGCTCCATGCAGGATGGTAACCGATATCCCAGGATCAATGGATGACACCTACGG
||||||||||||||||||||||||||||||||||||||||||||||||||||||||||||||||||||||||||||||||||||||||||||||||||||
GATCCAATATATCCTAACTAACTATCGACCGACTGATTCATGTCCATGCTCCATGCAGGATGGTAACCGATATCCCAGGATCAATGGATGACACCTACGG
TGAGCACTGCCCCTCGTGAATGCAATAATAGTACATGACTTGGCATCTCTGGACAGTTTGGATGTAAATTTTTCTTCATTGTCTGAAGGGCGGGAGGTGG
||||||||||||||||||||||||||||||||||||||||||||||||||||||||||||||||||||||||||||||||||||||||||||||||||||
TGAGCACTGCCCCTCGTGAATGCAATAATAGTACATGACTTGGCATCTCTGGACAGTTTGGATGTAAATTTTTCTTCATTGTCTGAAGGGCGGGAGGTGG
TGTGCTACGAGAGCCCGACGCCGGCGACGGGGATCCATCGCATGGTGCTGGTGCTGTTCCGGCAGCTCGGGCGGAACACGGTGTACGCACCGTCGATGCG
||||||||||||||||||||||||||||||||||||| ||||||||||||||||||||||||||||||||||||||||||||||||||||||||||||||
TGTGCTACGAGAGCCCGACGCCGGCGACGGGGATCCACCGCATGGTGCTGGTGCTGTTCCGGCAGCTCGGGCGGAACACGGTGTACGCACCGTCGATGCG
CCACAACTTCAACACCCGCAACTTCGCCCGCCGCTACAACCTTGGTGCGCCCGTCGCCGCAAAGTACTTCAACTGCCAGCGCCAGGCCGGCTCCGGCGGC
||||||||||||||||||||||||||||||||||||||||||||||||||||||||||||||||||||||||||||||||||||||||||||||||||||
CCACAACTTCAACACCCGCAACTTCGCCCGCCGCTACAACCTTGGTGCGCCCGTCGCCGCAAAGTACTTCAACTGCCAGCGCCAGGCCGGCTCCGGCGGC
CGGAAGTTTACCGGGGCCTACACCAGCCGCCGCCAGCAAATTTAATCCTTCGAGCTCAAATACCCACGTACCTAGCTACATAAATAGCTGCCACATACGG
|||||||| |||||||||||||||||||||||||||||||||||||||||||||||||||||||||||||||||||||||||||||||||||||||||||
CGGAAGTTCACCGGGGCCTACACCAGCCGCCGCCAGCAAATTTAATCCTTCGAGCTCAAATACCCACGTACCTAGCTACATAAATAGCTGCCACATACGG
GAGCACGTGGCGCACGCATGGGCATGGCAACGTTATCGATCACATGGGTATAAAGACAAGCTACAAATAGCTGTAGAAAAAGACCAGCTACAAATAAATG
||||||||||||||||||||||||||||||||||||||||||||||||||||||||||||||||||||||||||||||||||||||||||||||||||||
GAGCACGTGGCGCACGCATGGGCATGGCAACGTTATCGATCACATGGGTATAAAGACAAGCTACAAATAGCTGTAGAAAAAGACCAGCTACAAATAAATG
AATAAAGAGTACTTAGTCTTCCCAAAGGAGGATAGACCCCGGCTGGATGAATAAAAGTATGTAGCTTCTTCTCTCTTTTTTGCGGGAAAAGAGTACTCTA
||||||||||||||||||||||||||||||||||||||||||||||||||||||||||||||||||||||||||||||||||||||||||||||||||||
AATAAAGAGTACTTAGTCTTCCCAAAGGAGGATAGACCCCGGCTGGATGAATAAAAGTATGTAGCTTCTTCTCTCTTTTTTGCGGGAAAAGAGTACTCTA
CATAGCTTTTGAAGGCTT-------------CATCATATATGTGATCTATATAAATCTCTTTTCTGAAATAATATCTAGCCCCGCCTTCTATATACTCTC
|||||||||||||||||| |||||||||||||||||||||||||||||||||||||||||||||||||||||||||||||||||||||
CATAGCTTTTGAAGGCTTCACACAGATGTTGCATCATATATGTGATCTATATAAATCTCTTTTCTGAAATAATATCTAGCCCCGCCTTCTATATACTCTC
AAGACTCGACAAACTGATTTTCACCAGTTGTTATTCCTGGATGATCTCAAGCATTGCTGCTCCACTGCCCTGCCTTTGTTTGGTCGGCGCTGCTGACAAA
||||||||||||||||||||||||||||||||||||||||||||||||||||||||||||||||||||||||||||||||||||||||||||||||||||
AAGACTCGACAAACTGATTTTCACCAGTTGTTATTCCTGGATGATCTCAAGCATTGCTGCTCCACTGCCCTGCCTTTGTTTGGTCGGCGCTGCTGACAAA
AGCAACACCAACCTGAACCGGCGCACCATGGCTGCGTTCCGGCGTCTCATAAACGGGATGGAGCTTAAGGACATGTATCTCCATGCCGGAGATGCAC--T
||||||||||||||||||||||||||||||||||||||||||||||||||||||||||||||||||||||||||||||||||||||||||||||||| |
AGCAACACCAACCTGAACCGGCGCACCATGGCTGCGTTCCGGCGTCTCATAAACGGGATGGAGCTTAAGGACATGTATCTCCATGCCGGAGATGCACTTT
TTTTTTTCCTGAAAAAGGGGTATAACTCCGGCCTCTGCATCAGAACGATGCATACGGCCACCTTTATTATAAAGCAAATAAGTTTAACAAAGGTCATGAA
||||||||||||||||||| |||||||||||||||||||||||||||||||||||||||||||||||||||||||||||||||| |||||||||||| ||
TTTTTTTCCTGAAAAAGGGATATAACTCCGGCCTCTGCATCAGAACGATGCATACGGCCACCTTTATTATAAAGCAAATAAGTTCAACAAAGGTCATAAA
GTCTTAAACAAACGAACCACAAAAAGGCTCTTGCCTATCCTATTACATGACCGTCATCCAAACCGGTTGAATATAGCCCGAGCTACCATCTCCCATCGGG
||||||||||||||||||||||||||||||||||||||||||||||||||||||||||||||||||||||||||||||||||||| ||||||||||||||
GTCTTAAACAAACGAACCACAAAAAGGCTCTTGCCTATCCTATTACATGACCGTCATCCAAACCGGTTGAATATAGCCCGAGCTATCATCTCCCATCGGG
TAGATCCGGTAACCAAACGCTCCCTGGCCTCCGCC
|||||||||||||||||||||||||||||||||||
TAGATCCGGTAACCAAACGCTCCCTGGCCTCCGCC

***FT-D6 - TRIAE_CS42_6DS_TGACv1_543080_AA1735170***

AGGCTGTAACAATACTCCATGACAGTTTGAGCTGGAGAAATAATGCCAAGCCAACAATCACCAAGCTTCGTCAAAAGGGGTCGGTTCTCCATGGACGCAT
||||||||||||||||||||||||||||||||||||||||||||||||||||||||||||||||||||||||||||||||||||||||||||||||||||
AGGCTGTAACAATACTCCATGACAGTTTGAGCTGGAGAAATAATGCCAAGCCAACAATCACCAAGCTTCGTCAAAAGGGGTCGGTTCTCCATGGACGCAT
CTGTTCTAATCCCGCATTCATGTGAGCTCGGGTGAACAATAAAAACAAATAAAATGAAAAAAAAAACAATATGAATTTTTCTGACAATGTTTGGCATAAA
||||||||||||||||||||||||||||||||||||||||||||||||||||||||||||||||| ||||||||||||||||||||||||||||||||||
CTGTTCTAATCCCGCATTCATGTGAGCTCGGGTGAACAATAAAAACAAATAAAATGAAAAAAAAA-CAATATGAATTTTTCTGACAATGTTTGGCATAAA
AAAATCTGATTTTTTTGAATTTTTATTTTATTTTACTGTTCACTCAAGCTCGGGATCAGGACAGCAATTTGGGTCCTCCACGTGTTCCGTCATATCATAC
||||||||||||||||||||||||||||||||||||||||||||||||||||||||||||||||||||||||||||||||||||||||||||||||||||
AAAATCTGATTTTTTTGAATTTTTATTTTATTTTACTGTTCACTCAAGCTCGGGATCAGGACAGCAATTTGGGTCCTCCACGTGTTCCGTCATATCATAC
AAATGTCGCACAATATGTTGGCCCGTACACACATCAAATTGTGGATGTGTATATGCCCTCCAATAATTGTGCAAAGAGATGCTAGTTAGCTTTACTACAT
||||||||||||||||||||||||||||||||||||||||||||||||||||||||||||||||||||||||||||||||||||||||||||||||||||
AAATGTCGCACAATATGTTGGCCCGTACACACATCAAATTGTGGATGTGTATATGCCCTCCAATAATTGTGCAAAGAGATGCTAGTTAGCTTTACTACAT
ACGTCATTCTAGCTAGAAAGACATGCCTGGAATCAGCATGCACAACCGGTCAAATTTAGTGGAGCGAAAATTACTATCAAGCGCATACAACGAACGATGA
||||||||||||||||||||||||||||||||||||||||||||||||||||||||||||||||||||||||||||||||||||||||||||||||||||
ACGTCATTCTAGCTAGAAAGACATGCCTGGAATCAGCATGCACAACCGGTCAAATTTAGTGGAGCGAAAATTACTATCAAGCGCATACAACGAACGATGA
CAAGGATTGGAGCTGCATGTGATATGCATGGCGGTTTCTCGATCTTCTCATGGCTAGCTACTATACTATAAATATGACGCCTAACTACCTCTAGCTCTGT
||||||||||||||||||||||||||||||||||||||||||||||||||||||||||||||||||||||||||||||||||||||||||||||||||||
CAAGGATTGGAGCTGCATGTGATATGCATGGCGGTTTCTCGATCTTCTCATGGCTAGCTACTATACTATAAATATGACGCCTAACTACCTCTAGCTCTGT
AGTATCTTTATACCAAGCCATTACACTGAAGCTAGACATCAACACGCATACATCTGCGTATACTATACACATCATATATATACACTAGAAATGTCGAACG
||||||||||||||||||||||||||||||||||||||||||||||||||||||||||||||||||||||||||||||||||||||||||||||||||||
AGTATCTTTATACCAAGCCATTACACTGAAGCTAGACATCAACACGCATACATCTGCGTATACTATACACATCATATATATACACTAGAAATGTCGAACG
ACTCCTTGGTTACATCACGAATAGTAGGTGATGTGTTGGACCCCTTCCGTAGCACAGTTGATCTGACAGTGCTCTATGACGGTAGGTTCGTCATTAACGG
||||||||||||||||||||||||||||||||||||||||||||||||||||||||||||||||||||||||||||||||||||||||||||||||||||
ACTCCTTGGTTACATCACGAATAGTAGGTGATGTGTTGGACCCCTTCCGTAGCACAGTTGATCTGACAGTGCTCTATGACGGTAGGTTCGTCATTAACGG
CATGGAGTTCCGCTCACCGGCGGTGTCGGGCAAGCCTAGCGTCGAGATTGGCGGTGACGATATTAGCGTGACATACACCCTTGTAAGCCCATGATTTATT
||||||||||||||||||||||||||||||||||||||||||||||||||||||||||||||||||||||||||||||||||||||||||||||||||||
CATGGAGTTCCGCTCACCGGCGGTGTCGGGCAAGCCTAGCGTCGAGATTGGCGGTGACGATATTAGCGTGACATACACCCTTGTAAGCCCATGATTTATT
AGTTATTTATGTTTTCAGCACTGTGGTTTGCTCAAGTTAACTGTGTAATCTGTCAATGTGATATATGCAGGTCATGGTGGATCCTGATGCTCCTAACCCC
||||||||||||||||||||||||||||||||||||||||||||||||||||||||||||||||||||||||||||||||||||||||||||||||||||
AGTTATTTATGTTTTCAGCACTGTGGTTTGCTCAAGTTAACTGTGTAATCTGTCAATGTGATATATGCAGGTCATGGTGGATCCTGATGCTCCTAACCCC
AGCAATCCGACCTTGAGGGAATATCTTCACTGGTGAGAGGCTGAGTGCAACCCTACCTTGCTTGCTCTTGCATGCTGTCGATTAGGAACTATAATCTATA
||||||||||||||||||||||||||||||||||||||||||||||||||||||||||||||||||||||||||||||||||||||||||||||||||||
AGCAATCCGACCTTGAGGGAATATCTTCACTGGTGAGAGGCTGAGTGCAACCCTACCTTGCTTGCTCTTGCATGCTGTCGATTAGGAACTATAATCTATA
CATGCATCACATGTAACGGTGGATGTATGTATTGGTGCATGCATGATAATGATATAATATACTCATGGATCATGCACGTACGTACATAACCACAAATATG
||||||||||||||||||||||||||||||||||||||||||||||||||||||||||||||||||||||||||||||||||||||||||||||||||||
CATGCATCACATGTAACGGTGGATGTATGTATTGGTGCATGCATGATAATGATATAATATACTCATGGATCATGCACGTACGTACATAACCACAAATATG
CTTGTGGCAAGCGTGTCGATGGACATGACATACTATAAATATATGTGCATTATTTCAGGATAGCTAGGTTCATTGTGAGTACAGCTAGATATAGATCCAA
||||||||||||||||||||||||||||||||||||||||||||||||||||||||||||||||||||||||||||||||||||||||||||||||||||
CTTGTGGCAAGCGTGTCGATGGACATGACATACTATAAATATATGTGCATTATTTCAGGATAGCTAGGTTCATTGTGAGTACAGCTAGATATAGATCCAA
TATATCCTAACTAACTATCGACCGACTGATTCATGTCCATGCTCCATGCAGGATGGTAACCGATATCCCAGGATCAATGGATGACACCTACGGTGAGCAC
||||||||||||||||||||||||||||||||||||||||||||||||||||||||||||||||||||||||||||||||||||||||||||||||||||
TATATCCTAACTAACTATCGACCGACTGATTCATGTCCATGCTCCATGCAGGATGGTAACCGATATCCCAGGATCAATGGATGACACCTACGGTGAGCAC
TGCCCCTCGTGAATGCAATAATTATAATAGTACATGACTTGGCATCTCTGGACAGTTTGGATGTAAATTTTTCTTCGTTGTTTGAAGGGCGGGAGGTGGT
||||||||||||||||||||||||||||||||||||||||||||||||||||||||||||||||||||||||||||||||||||||||||||||||||||
TGCCCCTCGTGAATGCAATAATTATAATAGTACATGACTTGGCATCTCTGGACAGTTTGGATGTAAATTTTTCTTCGTTGTTTGAAGGGCGGGAGGTGGT
GTGCTACGAGAGCCCGACGCCGGCGACGGGGATCCACCGCATGGTGCTGGTGCTGTTCCGGCAGCTCGGGCGGAACACGGTGTACGCGCCGTCGATGCGC
||||||||||||||||||||||||||||||||||||||||||||||||||||||||||||||||||||||||||||||||||||||||||||||||||||
GTGCTACGAGAGCCCGACGCCGGCGACGGGGATCCACCGCATGGTGCTGGTGCTGTTCCGGCAGCTCGGGCGGAACACGGTGTACGCGCCGTCGATGCGC
CACAACTTCAACACCCGCAACTTCGCCCGCCGCTACAACCTTGGCGCGCCCGTCGCCGCAAAGTACTTCAACTGCCAGCGCCAGGCCGGCTCCGGCGGCC
||||||||||||||||||||||||||||||||||||||||||||||||||||||||||||||||||||||||||||||||||||||||||||||||||||
CACAACTTCAACACCCGCAACTTCGCCCGCCGCTACAACCTTGGCGCGCCCGTCGCCGCAAAGTACTTCAACTGCCAGCGCCAGGCCGGCTCCGGCGGCC
GGAAGTTCACCGGGCCCTACACCAGCCGCCGCCAGCAAATCTAATCCTTCGAGCTCAAATACCCACGTACCTAGCTAAATAAATAGCTGCCACATACGGG
||||||||||||||||||||||||||||||||||||||||||||||||||||||||||||||||||||||||||||||||||||||||||||||||||||
GGAAGTTCACCGGGCCCTACACCAGCCGCCGCCAGCAAATCTAATCCTTCGAGCTCAAATACCCACGTACCTAGCTAAATAAATAGCTGCCACATACGGG
AGCACGTGGCGCACGCATGGGCATGGCAACGTCATCGATCACATGGGTATAAGACTAGCTACAAATACCTGTAGAAAAAGACTAGCTACAAATAAATGAA
||||||||||||||||||||||||||||||||||||||||||||||||||||||||||||||||||||||||||||||||||||||||||||||||||||
AGCACGTGGCGCACGCATGGGCATGGCAACGTCATCGATCACATGGGTATAAGACTAGCTACAAATACCTGTAGAAAAAGACTAGCTACAAATAAATGAA
TAAAAGTACTTCTCTTCCCAAAGGAAGATAGACCCCGGCTGGATGAATAAAAGTACGTAGCTTATTCTCTCCTTTTTGCGGGAAAAGAGTACTCTACATA
||||||||||||||||||||||||||||||||||||||||||||||||||||||||||||||||||||||||||||||||||||||||||||||||||||
TAAAAGTACTTCTCTTCCCAAAGGAAGATAGACCCCGGCTGGATGAATAAAAGTACGTAGCTTATTCTCTCCTTTTTGCGGGAAAAGAGTACTCTACATA
GCTTTTGAAAGCTTCACATAGATGTTGCATCATACATGTGATCTATATAAATCTCTTTCTGAAATAATATCTAGCTTCGCCTTCTATATACTCTCAAGAC
||||||||||||||||||||||||||||||||||||||||||||||||||||||||||||||||||||||||||||||||||||||||||||||||||||
GCTTTTGAAAGCTTCACATAGATGTTGCATCATACATGTGATCTATATAAATCTCTTTCTGAAATAATATCTAGCTTCGCCTTCTATATACTCTCAAGAC
TCGACAAACTGATTTTCACCTGTTGTTATTCCTGGATGATCTCAAGCATTGCTGCTCCACTGCCCTGCCTTTGTTTGGTCGGCGCTGCTGACAGAAGCAA
||||||||||||||||||||||||||||||||||||||||||||||||||||||||||||||||||||||||||||||||||||||||||||||||||||
TCGACAAACTGATTTTCACCTGTTGTTATTCCTGGATGATCTCAAGCATTGCTGCTCCACTGCCCTGCCTTTGTTTGGTCGGCGCTGCTGACAGAAGCAA
CACCAATCTGAACCGGCGCACCATGGCTGCGTTCTGGCGTCTCATAAACGGGATGGAGCTCAAGGACATGTATCTCCATGGCCGGAGATGCACTTGGTCC
||||||||||||||||||||||||||||||||||||||||||||||||||||||||||||||||||||||||||||||||||||||||||||||||||||
CACCAATCTGAACCGGCGCACCATGGCTGCGTTCTGGCGTCTCATAAACGGGATGGAGCTCAAGGACATGTATCTCCATGGCCGGAGATGCACTTGGTCC
GATAAGCAGCAGGCTGCGAGCAAGGAAGCTCGACAGGCTGTTGCTTAAGTCATAAATTCACTGAAGGTCACTGGACTCGTCCCAAATTTTATTTCAGTCA
||||||||||||||||||||||||||||||||||||||||||||||||||||||||||||||||||||||||||||||||||||||||||||||||||||
GATAAGCAGCAGGCTGCGAGCAAGGAAGCTCGACAGGCTGTTGCTTAAGTCATAAATTCACTGAAGGTCACTGGACTCGTCCCAAATTTTATTTCAGTCA
CTGGACTTAGAAATGCCTGAAATATGGTGAAGAAAGTCATATCCGTGAACGCCTACGGTCATCGTGTACGCTTTGGTTATGTATTCTGTCTACGTGCCAT
||||||||||||||||||||||||||||||||||||||||||||||||||||||||||||||||||||||||||||||||||||||||||||||||||||
CTGGACTTAGAAATGCCTGAAATATGGTGAAGAAAGTCATATCCGTGAACGCCTACGGTCATCGTGTACGCTTTGGTTATGTATTCTGTCTACGTGCCAT
ACTATTTAACCGATTGAC
||||||||||||||||||
ACTATTTAACCGATTGAC
