# Supplementary material for: Developmental responses of bread wheat to changes in ambient temperature following deletion of a locus that includes FLOWERING LOCUS T1
Source: Plant Cell Environ. 2018 Feb 7;41(7):1715–25. doi: 10.1111/pce.13130 (PMC6033019; doi:10.1111/pce.13130)
Supplement: Supplementary file 1 — Figure S1: Flowering time under different ambient temperatures Figure S2: Gene expression of activators in flowering regulation Figure S3: Gene expression of FT genes in FT‐B1 NILs Figure S4: FT alleles in the Paragon cultivar Table S1: Oligonucleotides used for determining the extent of the FT‐B1 deletion Table S2: List of all predicted genes and gene models within the deleted region of 7BS from the IWGSC alignment Table S3: Oligonucleotides used in Q‐PCR analysis [file PCE-41-1715-s001.zip › Supplementary information.docx]

**Supplementary Information**

*
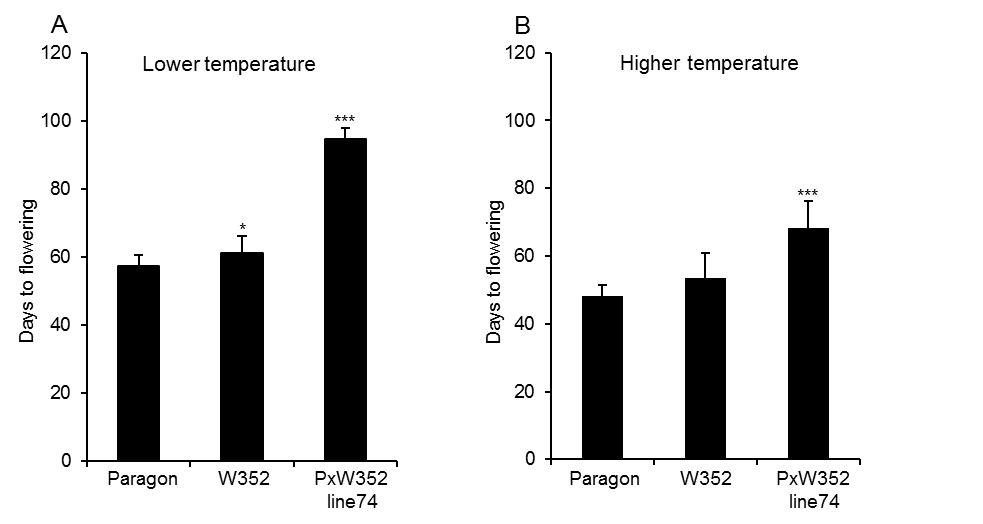
*

**Supplementary Figure 1:** **Flowering time under different ambient temperatures.** Flowering time was measured as half-ear emergence for N=5 plants from two independent experiments. Parent lines Paragon and W352 and progeny PxW352 line74 were grown from germination under either (A) lower ambient temperature (18 °C light: 13 °C dark) LD conditions or (B) higher ambient temperature (24 °C light: 19 °C dark) LD conditions. Value is taken as an average from the two independent experiments with SEM error bars. Significance determined by Student’s t-test between Paragon and either W352 or PxW352 line74 (* p<0.05, *** p<0.001).

*
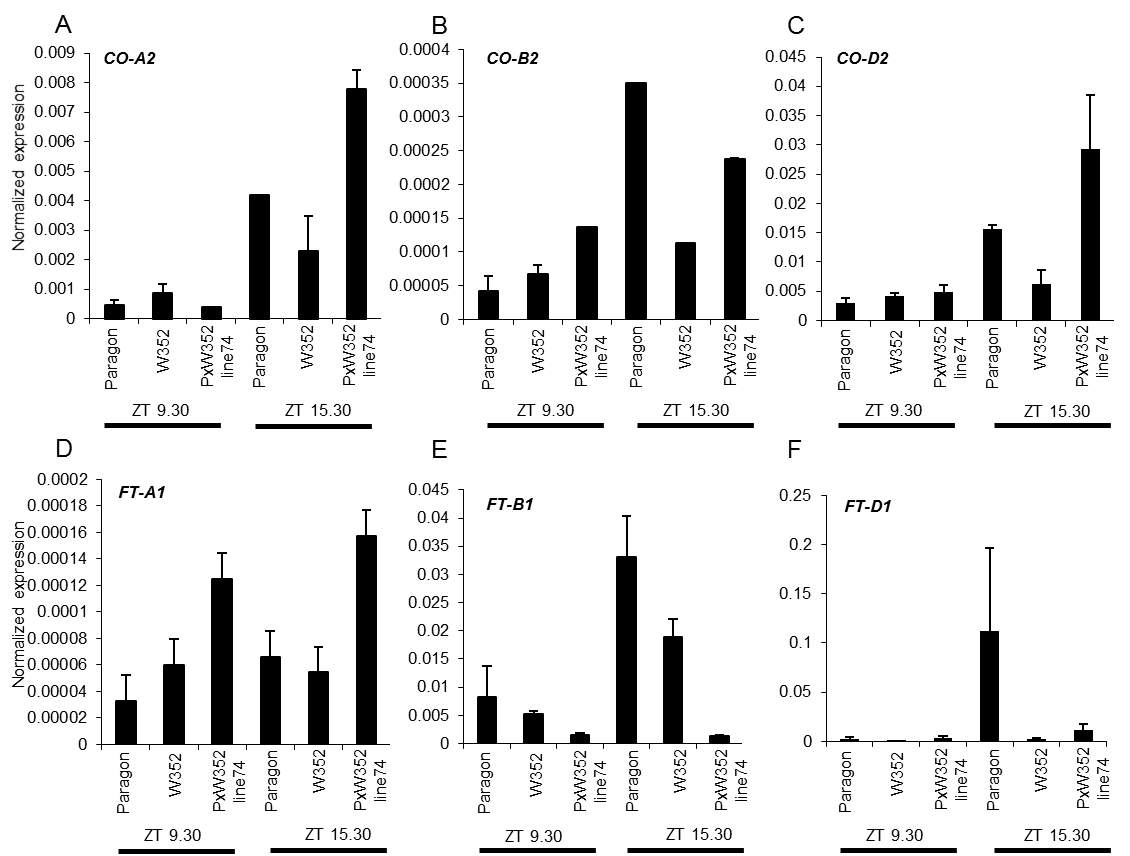
*

**Supplementary Figure 2:** **Gene expression of activators in flowering regulation**

From the same gene expression experiment shown in Figure 1, gene expression of A) *CO-A2*, B) *CO-B2*, C) *CO-D2*, D) *FT-A1,* E) *FT-B1* (as shown in Figure 1B and included here for completeness) F) *FT-D1* normalized to *GAPDH* expression at ZT9.30 and ZT15.30.

*
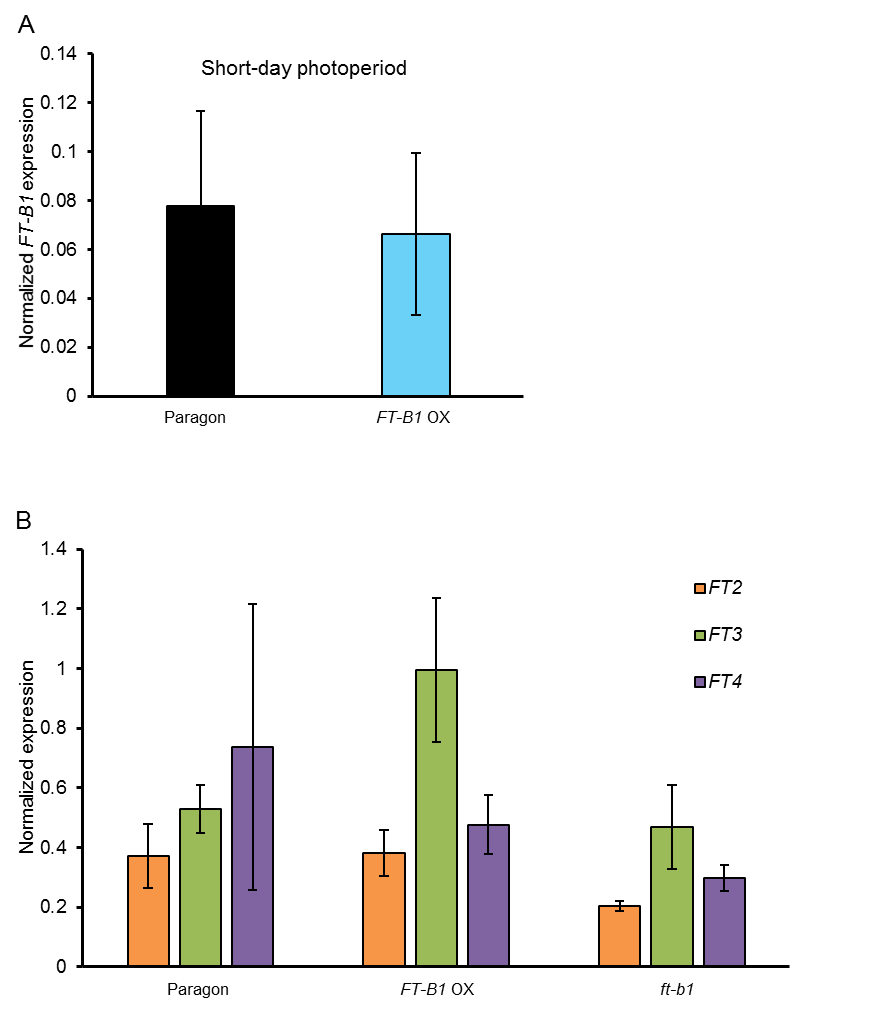
*

**Supplementary Figure 3: Gene expression of *FT* genes in *FT-B1* NILs**

A) *FT-B1* expression was measured under SD photoperiod for Paragon and *FT-B1* OX NIL in third-leaf tissue at ZT7, 1h before lights off in the SD photoperiod. Expression is normalized to TRIAE_CS42_5AS_TGACv1_392916_AA1266200, n=4 and error is SEM. B) *FT2*, *FT3* and *FT4* expression under LD photoperiod from third-leaf tissue at ZT15, 1 h before lights off in the LD photoperiod in Paragon, *FT-B1* OX and *ft-b1* NIL. Expression is normalized to TRIAE_CS42_6DS_TGACv1_544038_AA1746170, n=4 and error is SEM.

**Supplementary Figure 4:** ***FT* alleles in the Paragon cultivar**

Allelic sequence for all of the currently identified Paragon *FT* genes (*FT-A1*, *FT-B1*, *FT- D1*, *FT-A2*, *FT-B2*, *FT-D2*, *FT-A3*, *FT-B3*, *FT-D3*, *FT-A4*, *FT-B4*, *FT-D4*, *FT-A5*, *FT-B5*, *FT-D5,* *FT-A6*, *FT-B6* and *FT-D6*) from exome capture data available through Earlham Institute Grassroots ([http://www.earlham.ac.uk/ grassroots-genomics/](http://www.earlham.ac.uk/%20grassroots-genomics/)) aligned to reference sequence from [http://plants.ensembl.org/ Triticum_aestivum/Info/Index/](http://plants.ensembl.org/%20Triticum_aestivum/Info/Index/).

**Supplementary Table 1:** **Oligonucleotides used for determining the extent of the *FT-B1* deletion**

**Supplementary Table 2:** **List of all predicted genes and gene models within the deleted region of 7BS from the IWGSC alignment**

**Supplementary Table 3: Oligonucleotides used in Q-PCR analysis**
